# Iron oxide-promoted photochemical oxygen reduction to hydrogen peroxide (H_2_O_2_)†

**DOI:** 10.1039/d3ey00256j

**Published:** 2023-11-24

**Authors:** Thomas Freese, Jelmer T. Meijer, Maria B. Brands, Georgios Alachouzos, Marc C. A. Stuart, Rafael Tarozo, Dominic Gerlach, Joost Smits, Petra Rudolf, Joost N. H. Reek, Ben L. Feringa

**Affiliations:** a Stratingh Institute for Chemistry, University of Groningen Nijenborgh 4 9747 AG Groningen The Netherlands b.l.feringa@rug.nl; b van’t Hoff Institute for Molecular Sciences, University of Amsterdam Science Park 904 1098 XH Amsterdam The Netherlands; c Electron Microscopy, Groningen Biomolecular Sciences and Biotechnology Institute, University of Groningen Nijenborgh 7 9747AG Groningen The Netherlands; d Zernike Institute for Advanced Materials, University of Groningen Nijenborgh 4 9747AG Groningen The Netherlands; e Shell Global Solutions International BV Grasweg 31 1031 HW Amsterdam The Netherlands

## Abstract

Hydrogen peroxide (H_2_O_2_) is a valuable green oxidant with a wide range of applications. Furthermore, it is recognized as a possible future energy carrier achieving safe operation, storage and transportation. The photochemical production of H_2_O_2_ serves as a promising alternative to the waste- and energy-intensive anthraquinone process. Following the 12 principles of Green Chemistry, we demonstrate a facile and general approach to sustainable catalyst development utilizing earth-abundant iron and biobased sources only. We developed several iron oxide (FeO_*x*_) nanoparticles (NPs) for successful photochemical oxygen reduction to H_2_O_2_ under visible light illumination (445 nm). Achieving a selectivity for H_2_O_2_ of >99%, the catalyst material could be recycled for up to four consecutive rounds. An apparent quantum yield (AQY) of 0.11% was achieved for the photochemical oxygen reduction to H_2_O_2_ with visible light (445 nm) at ambient temperatures and pressures (9.4–14.8 mmol g^−1^ L^−1^). Reaching productivities of H_2_O_2_ of at least 1.7 ± 0.3 mmol g^−1^ L^−1^ h^−1^, production of H_2_O_2_ was further possible *via* sunlight irradiation and in seawater. Finally, a detailed mechanism has been proposed on the basis of experimental investigation of the catalyst's properties and computational results.

## Abstract 2

A sustainable catalyst development utilizing earth-abundant iron oxide (FeO_*x*_) nanoparticles for photochemical oxygen reduction to hydrogen peroxide (H_2_O_2_) was achieved, generating a valuable green oxidant and future energy carrier.

## Graphical abstract

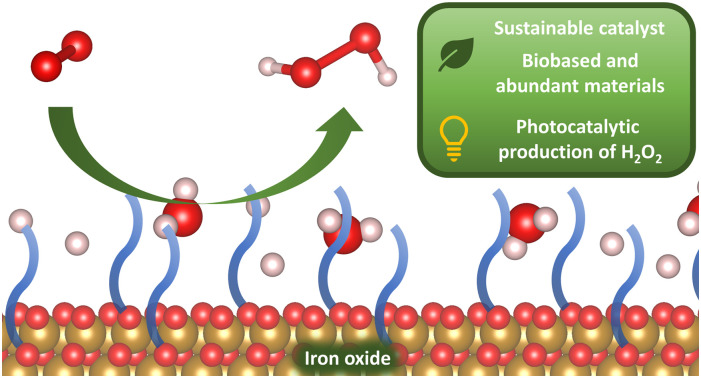

**Broader context**

Facing the environmental crisis, the energy transition to renewables (wind and solar) increases global demand for future energy carriers. Next to its applications as an eco-friendly oxidant, H_2_O_2_ offers great potential as an energy carrier as it is fully soluble in water. Hence it offers an easy-to-handle liquid fuel alternative achieving safer operation, storage and transportation. The solar-driven reduction of molecular oxygen to produce H_2_O_2_ is an ecologically viable route, especially when sustainable and biobased photocatalysts are utilized. We established a strategy for the photochemical production of H_2_O_2_ catalysed by iron. The sustainable synthesis of iron oxide (FeO_*x*_) nanoparticles terminated with different surfactants was demonstrated, where specifically FeO_*x*_ NPs with *cis* double bonds possess photoactivity for oxygen reduction to hydrogen peroxide. An apparent quantum yield (AQY) of 0.11% was achieved for the photochemical oxygen reduction to H_2_O_2_ with visible light (445 nm) at ambient temperatures and pressures (9.4–14.8 mmol g^−1^ L^−1^), corresponding to 1.7 ± 0.3 mmol g^−1^ L^−1^ h^−1^. The H_2_O_2_ yield could be increased by decreasing the pH, addition of cation exchangers and production in biphasic systems (heptane/DCM with Milli-Q water) (up to 19.5 ± 2.7 mmol g^−1^ L^−1^). The FeO_*x*_ nanoparticles with oleic acid (2 : 1) as a surfactant were successfully utilized in applications like wastewater treatment, polymerizations and *in situ* oxidations. Production of H_2_O_2_ was possible *via* sunlight irradiation and in seawater. Utilizing earth-abundant metals and biobased (co-)catalysts offers great potential for the photocatalytic production of hydrogen peroxide as a solar fuel.

## Introduction

Hydrogen peroxide (H_2_O_2_) is a versatile green oxidant with large scale applications in the chemical industry, pulp and paper bleaching, wastewater treatment and disinfectants.^1–6^ H_2_O_2_ is also utilized in fuel cells as an advantageous energy carrier over hydrogen (H_2_).^7–14^ Despite major advances in the generation of H_2_ from water, its storage and low energy density (per volume) are still a major bottleneck.^15–18^ Being far from practical with regards to the energy density of H_2_ per volume at atmospheric pressure, stationary fuel storage of H_2_ requires chemical transformation into transportable liquids (*e.g.* ammonia or formic acid).^19–29^ Pure H_2_ stored in a tank at 35 MPa (room temperature) delivers 2.8 MJ L^−1^ when operated in a fuel cell, which is very similar to the energy density of aqueous H_2_O_2_ (70 wt%) with 3.1 MJ L^−1^.^30,31^ Thus H_2_O_2_, being fully soluble in water, offers an easy-to-handle liquid fuel alternative achieving safer operation, storage and transportation.^3,4^ The global market for H_2_O_2_ is estimated to grow at a compound annual growth rate (CAGR) of 4.6% increasing to 5.7 million tons annual demand by 2028.^32–34^ Its characteristics as a high-energy fuel and as an ecological oxidant, generating water (H_2_O) and oxygen (O_2_) as the only by-products, constitute H_2_O_2_ being listed as one of the 100 most important chemicals on earth.^35,36^

Currently more than 95% of H_2_O_2_ is produced *via* the anthraquinone process, comprising Pd-catalysed hydrogenation of an alkyl-anthraquinone and consecutive oxidation in organic solvents.^1,37^ However, this synthetic strategy involves high energy input and generates a substantial volume of wastewater and solid waste.^1,36^ With increasing demand for a sustainable alternative, the electrocatalytic oxygen reduction reaction (ORR) to form H_2_O_2_,^38–40^ as well as the direct synthesis of H_2_O_2_ from H_2_ and O_2_, offer apparent solutions to these problems.^41–48^ Nevertheless high energy consumption and inevitable high explosion risks of O_2_ and H_2_ gas mixtures hamper the industrial scale up of these systems.^7,9^

Consequently, utilizing green energy sources such as solar energy for the photochemical production of H_2_O_2_ directly from water serves as an alternative, cleaner method to meet the global demand for H_2_O_2_.^4,9,33^ By combining the photocatalytic two-electron reduction of O_2_ (ORR, +0.68 V_NHE_) and the catalytic four-electron oxidation of H_2_O (WOR, +1.23 V_NHE_), the overall photosynthesis of H_2_O_2_ from water can be achieved (eqn (1)).^3,49^

1 2H_2_O + O_2_ → 2H_2_O_2_ (Δ*G*° = 204 kJ mol^−1^)

Over the past decade, remarkable progress towards the photocatalytic production of H_2_O_2_ has been accomplished, where initial homogeneous catalysts^50^ were further developed towards heterogeneous systems predominantly.^20,51–53^ Catalyst systems range from metals,^20,54,55^ metal nanoparticles (NP)^56–59^ or metal–organic frameworks (MOFs)^60–62^ to nonmetal variants^63,64^ such as graphitic carbon nitride (g-C_3_N_4_),^65,66^ resorcinol-formaldehyde resins,^19,67^ conjugated polymers^68,69^ or covalent organic frameworks (COFs).^70–73^

**Fig. 1:**
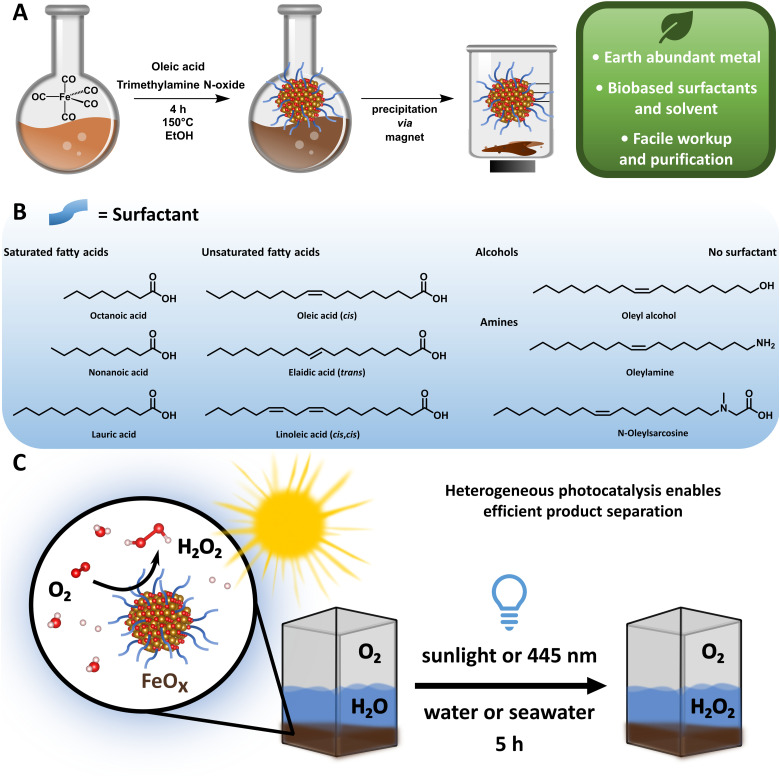
(A) Schematic representation of the synthesis of FeO_*x*_ NPs in ethanol (EtOH), magnetic precipitation and its sustainability advantages. (B) Scope of different biobased surfactants assessed as ligands in FeO_*x*_ NPs for H_2_O_2_ production. (C) Photochemical oxygen reduction to H_2_O_2_*via* FeO_*x*_ NPs in water (1 mg mL^−1^) at 445 nm (5 h).

Despite these advances, the metal-based photocatalysts often rely on noble and scarce metals, the starting materials and solvents are not biobased and the catalyst synthesis requires special equipment or high temperatures.^74^ Thus, legitimate H_2_O_2_ production as a future energy carrier can only be achieved by a green photocatalyst complying with the Sustainable Development Goals and the 12 principles of Green Chemistry (Fig. 1A).^75,76^ Indeed, the development of sustainable materials such as catalysts,^20,77–79^ polymers,^80,81^ coatings^82,83^ and molecular motors^84^ from renewable and earth abundant sources has received high priority in the past, avoiding dependency on fossil resources. Considering element scarcity and climate change, iron is the fourth most abundant element in the earth's crust and the most abundant metal, and thus an ideal candidate as an environmental photocatalyst material.^85,86^

Based on these considerations, we envisioned a possibility to adapt previously reported iron oxide (Fe_3_O_4_) nanoparticles (NPs) for our approach,^87^ as these particles, next to other iron oxides,^88^ already demonstrated promising electrocatalytic activity for the two-electron ORR to H_2_O_2_.^89,90^ We hypothesized that these might also have potential for the photocatalytic reduction of oxygen. Also, the proven electrochemical water splitting capability of iron oxide materials makes such catalysts interesting for photochemical H_2_O_2_ production including possible four-electron WOR.^91,92^ To the best of our knowledge, no pure iron oxide photocatalyst system has been reported to date, since iron oxide (Fe_2_O_3_) was only utilized as a support material before.^55^

Here we report an improved preparation method compared to the original synthesis of Fe_3_O_4_ NPs regarding solvent, energy consumption, workup and overall sustainability,^93,94^ and provide a scope of different nanoparticles, which can be synthesized and purified *via* a sustainable and facile route within six hours (Fig. 1A and B). The synthesized iron oxide nanoparticles (FeO_*x*_ NPs) with oleic acid (OA, 2 : 1 ratio) as a surface ligand possess photochemical activity for oxygen reduction to selectively form H_2_O_2_ (up to 19.5 ± 2.7 mmol g^−1^ L^−1^, Fig. 1C) and could be recycled several times (up to four rounds). The production of H_2_O_2_ was achieved at ambient temperatures and pressures upon irradiation of the NPs with 445 nm light (5 h), and a detailed study of the oxygen reduction mechanism was conducted. Considering the high abundance of seawater and sunlight, the successful H_2_O_2_ production under these conditions *via* FeO_*x*_ NPs provides indeed a basis for a sustainable solution in the H_2_O_2_ market.

## Results and discussion

We investigated the ORR aiming at modifying the synthesis of Fe_3_O_4_ towards a more sustainable route and tuning the properties of the material towards a photocatalyst.^87,95^ This was especially of interest as similar iron-based materials possess electrochemical water splitting capability, which if tuned properly could lead to photochemical four-electron WOR and subsequent two-electron ORR properties.^91^

The original solvent dioctyl ether (BP: 292 °C) was replaced with ethanol (BP: 78 °C), which not only allowed for less energy intensive reflux conditions, but also a greener solvent (Table 1).^96–98^ Instead of separation by centrifugation, utilizing the magnetic properties of the nanoparticles and precipitation on a magnet allowed for facile purification. An extensive investigation of the synthesis conditions was conducted yielding photoactive FeO_*x*_ NP material eventually. As heterogeneous catalyst performance can strongly be impacted by changes in parameters such as temperature, particle size, pore dimensions and reactor configuration, we envisioned the material to be sensitive to changes in mass and heat transport.^99^ A thorough optimization of glassware (round bottom flask size, beaker size for magnetic precipitation), stirring bar size and shape, magnetic stirring *vs.* mechanical stirring, reflux temperature (78–150 °C), atmosphere (air, N_2_) and storage conditions was conducted (ESI-3.2 and ESI-3.3,†). The consistent synthesis of photoactive heterogeneous FeO_*x*_ catalyst material could successfully be reproduced by several researchers in labs at different locations (ESI,† Fig. S110). As the reproducibility of heterogeneous catalyst materials is an often-overlooked aspect, achieving synthesis independent of iron(0) pentacarbonyl (FeCO_5_) suppliers (Sigma Aldrich, Acros Organics) from different Lot-numbers/continents was essential. The overall sustainability of the whole process has been assessed in Table 1.

**Table 1:** Justification of the principles of Green Chemistry. Relevant principles of Green Chemistry and analysis for the photochemical production of H_2_O_2_^76^

Principle	Justification
(1) Prevention of waste	As the synthesis of the Fe NPs is conducted in one step and purified *via* precipitation, stoichiometric amounts of waste could be minimized. Purification techniques such as column chromatography or centrifugation could be replaced by precipitation *via* magnetic properties of the NPs. As for the production of H_2_O_2_ no side products are obtained and the catalyst is not leached into the water.
(3) Less hazardous chemical synthesis	The synthetic method for the nanoparticles is designed for relatively low temperatures (150 °C), relies on biobased surfactants such as oleic acid, which can be obtained from olive oil, and utilizes non-harmful solvents such as ethanol in synthesis and workup.^96,101^ As for the metal, only earth-abundant iron (Fe) is utilized for the synthesis of the nanoparticles.^85^ In future optimizations the iron source (Fe(CO)_5_) will be replaced with other materials such as FeCl_2_ or FeCl_3_. The photochemical production of hydrogen peroxide is conducted with visible light in water, generating less harmful diluted H_2_O_2_ for application.^9^
(5) Benign solvents & auxiliaries	Environmentally benign, biobased and only non-halogenated solvents (water, ethanol) were used throughout the synthesis and purification of the nanoparticles, as well as during the production of H_2_O_2_. Abundant seawater and lake water can also be utilized for the production.^20^
(6) Energy efficiency	The original solvent dioctyl ether (BP: 292 °C) was replaced with ethanol (BP: 78 °C), which allowed for less energy intensive reflux conditions during catalyst synthesis. The photochemical production of H_2_O_2_ was fuelled by visible light (445 nm) and even by sunlight (September 2022) at room temperature. Thus, avoiding energy intensive high-pressure/temperature conditions.
(7) Renewable feedstocks	Considering element scarcity and CO_2_ footprint, iron is the fourth most abundant element in the earth's crust and the most abundant metal, and thus an ideal candidate as an environmental photocatalyst material.^86,93^ All surfactants utilized for the NP scope are biobased and renewable.^100–102^
(8) No derivatives	Derivatization is avoided and the whole synthesis towards the catalyst material or H_2_O_2_ was conducted without the use of protecting groups.
(9) Catalysis	An environmentally benign heterogeneous photocatalyst has been developed for the production of H_2_O_2_ in water. Utilizing heterogeneous systems over homogeneous ones allows for facile product separation and purification. Replacing the Pd-catalysed anthraquinone process *via* heterogeneous photocatalysts is less energy intensive and generates less waste.^3^
(10) Design for degradation	H_2_O_2_ as a product decomposes naturally on iron surfaces, which in fact can be used for degradation and wastewater treatment.^111^ The successful decomposition of an organic dye (methylene blue) *via* FeO_*x*_ NPs has been confirmed (ESI-10.4).

**Fig. 2:**
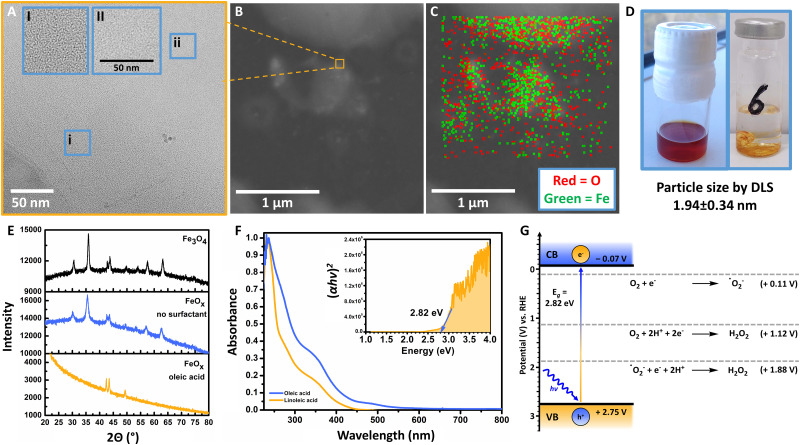
(A) Transmission electron microscopy (TEM) of FeO_*x*_ (batch 131, oleic acid 2 : 1, Acros Organics, 1 mg mL^−1^ in THF), at a magnification of 100 000× (inlet: zoomed); particle size by DLS 1.94 ± 0.34 nm. (B) Scanning transmission electron microscopy of FeO_*x*_ (batch 131, oleic acid 2 : 1, 1 mg mL^−1^ in THF), inlet: zoom towards A. (C) EDX of FeO_*x*_ (batch 131, oleic acid 2 : 1, 1 mg mL^−1^ in THF), drying spots of solvents contain more FeO_*x*_ NPs and concentration decreases towards the edges of the droplets; oxygen is depicted in red – iron in green. (D) FeO_*x*_ NPs stored in DCM and N_2_ atmosphere after synthesis (left); heterogeneous FeO_*x*_ catalyst material after drying, insoluble in water for photoirradiation studies (1 mg mL^−1^). (E) XRD comparison between FeO_*x*_ NPs with oleic acid (2 : 1, batch 135, Acros Organics) surfactant, without surfactant (batch 143, Acros Organics) and Fe_3_O_4_. The FeO_*x*_ photocatalyst resembles Fe_3_O_4_ as indicated by the peaks in the region 40–55 degrees; peak broadening is observed due to small size and/or amorphous properties of the FeO_*x*_ NP material. (F) Normalized UV-Vis absorption spectra for FeO_*x*_ species possessing photochemical activity for oxygen reduction towards H_2_O_2_ (oleic acid: 0.125 mg mL^−1^, linoleic acid: 0.02 mg mL^−1^ in DCM); inlet: UV-Vis Tauc plot indicating the optical bandgap of the FeO_*x*_ catalyst, where *h* = Planck's constant, *ν* = frequency of the radiation and *α* = absorption coefficient. The Tauc plots of different batches, solvents (THF and DCM) and heterogeneous deposition on the side of the cuvettes (AQY measurements) were compared. Conditions: batch 188 of FeO_*x*_ NPs measured in DCM (*c* = 1 mg mL^−1^). (G) Energy band position of the FeO_*x*_ catalyst material obtained *via* UV-Vis Tauc plot and electrochemical Mott-Schottky analysis *via* FeO_*x*_@FTO.

The general synthesis of FeO_*x*_ NPs allowed for a wide variety of biobased capping agents (Fig. 1B), which were fully characterized (ESI-4,†).^100–102^ Analyses by transmission electron microscopy (TEM), scanning transmission electron microscopy (STEM), energy-dispersive X-ray spectroscopy (EDX), dynamic light scattering measurements (DLS), powder X-ray diffraction (XRD), UV-visible spectroscopy (UV-Vis) and elemental analysis were conducted to assess the properties for photochemical ORR activity. Specifically, FeO_*x*_ with oleic acid and linoleic acid possessed photochemical activity for the production of H_2_O_2_. Additional immobilization of FeO_*x*_ with oleic acid on graphene and activated carbon (C) was achieved (Fig. S47–S56, ESI†), where lower but still present activity for photochemical ORR was observed when FeO_*x*_@C was used. Through DLS measurements we correlated the particle size to the photochemical activity: FeO_*x*_ NPs with oleic acid and linoleic acid were smaller (1.94 ± 0.34 nm and 1.54 ± 0.26 nm, respectively) than nanoparticles with other ligands (2.5–7.5 nm, Fig. S37, ESI†). While the synthesis offered great selectivity for consistent size below 10 nm, capping with oleyl alcohol or without surfactant led to larger particles (377 ± 166 nm and 1299 ± 100 nm, respectively, Table S1, ESI†).^103^ The monodisperse small nanoparticles were further visualized *via* TEM, STEM and EDX indicating successful incorporation of oxygen (O) and iron (Fe) (Fig. 2A–C). UV-Vis spectroscopy revealed that all catalyst materials possess similar absorption spectra (ESI,† Fig. S82). Interestingly, no significant difference between active FeO_*x*_ NPs with oleic acid (*cis*)/linoleic acid (*cis*,*cis*) and inactive NPs with elaidic acid (*trans*) was observed (ESI,† Fig. S83). As FeO_*x*_ NPs with oleic acid provided the highest photochemical production of H_2_O_2_ while also being the most abundant and cost-efficient surfactant,^100,101^ we analysed those in detail and will refer to those as standard when mentioning FeO_*x*_ in upcoming sections.

### Catalyst properties and performance

The synthesis of the FeO_*x*_ NPs offered a consistent particle size of 1.94 ± 0.34 nm (DLS), which was also confirmed by TEM (Fig. 2A and B). Energy-dispersive X-ray spectroscopy allowed for the visualization of oxygen (O) and iron (Fe) on the images, indicating a higher concentration of NPs at the drying spots of the solvent (1 mg mL^−1^, THF, Fig. 2A and C). As previously confirmed by Xiao *et al.*, comparing the FeO_*x*_ NPs with Fe_2_O_3_ and Fe_3_O_4_*via* X-ray powder diffraction (XRD) revealed that the photocatalyst indeed resembles Fe_3_O_4_ (Fig. 2E). Possessing distinct peaks at 42.4°, 43.4° and 49.4° the FeO_*x*_ does not resemble Fe_2_O_3_ but Fe_3_O_4_.^104–106^ Here, peak broadening was mainly correlated to the small size (1.94 nm) of the nanoparticles, but additionally could indicate the material being more amorphous than commercial Fe_3_O_4_ or FeO_*x*_ synthesized without surfactant (Fig. 2E), which could also be observed *via* TEM.^107,108^ As depicted in Fig. 2D, the brown FeO_*x*_ NPs are stable in hydrophobic solvents (DCM). This colour was retained when the heterogeneous catalyst was dried on glass for photoirradiation studies in water (1 mg mL^−1^, hydrophilic). Thus, the surfactant properties allow for facile catalyst and product separation from the product solution. The brown colour (absorbance up to 600 nm) of FeO_*x*_ is also depicted in the UV-Vis spectrum in Fig. 2F. The Tauc plot analysis of the photocatalyst exhibits an adequate band gap in the visible-light region of 2.82 eV (Fig. 2F inlet). Mott-Schottky measurements (ESI-4.7,†) revealed that the flat-band potentials or conduction band (CB) of the FeO_*x*_ NPs are positioned at −0.07 V *vs.* RHE. These results suggest that the catalyst material is able to facilitate indirect 2e^−^ oxygen reduction (ORR: +0.11 V *vs.* RHE) *via* superoxide ˙O_2_^−^ towards H_2_O_2_ without significant overpotential (Fig. 2G).

Photoirradiation studies were generally conducted in a batch irradiation setup allowing for temperature controlled high-throughput screening of 24 conditions simultaneously (ESI-5,†).^109^ We opted for an Oslon SSL 80 royal blue LED (500 mW, *λ* = 445 nm, 180 mW cm^−2^) as the FeO_*x*_ catalyst showed adequate absorbance in the visible-light region. After storage of the nanoparticles in DCM the catalyst was added to a vial (10 mL) to obtain a concentration of 4 mg per 4 mL solvent (generally H_2_O) after evaporation of DCM (Fig. 2D). The photoreactions were carried out in triplicate for 5 h in an oxygen atmosphere (20 °C) with irradiation from the bottom, where several blank reactions (including blanks in darkness) were performed in triplicate.

**Fig. 3:**
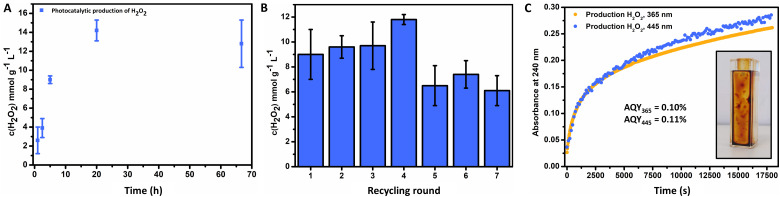
(A) Kinetics for the photochemical ORR of FeO_*x*_ with oleic acid (2 : 1) surfactant (1 mg mL^−1^), obtained by irradiation with 445 nm in Milli-Q water at 20 °C. (B) Recycling of FeO_*x*_ with oleic acid (2 : 1) surfactant (1 mg mL^−1^), obtained by irradiation for 5 h at 20 °C repeatedly with 445 nm in fresh Milli-Q water (30 min oxygenated) every round. (C) Production of hydrogen peroxide over time (∼5 h) upon irradiation with 445 nm and 365 nm, followed at 240 nm for apparent quantum yield (AQY) measurements; AQY_365_ = 0.10%, AQY_445_ = 0.11%. Inlet: immobilization.

The general catalyst performance is depicted in Fig. 3, where successful photochemical production of H_2_O_2_ was achieved *via* the FeO_*x*_ catalyst. Initially, an investigation of optimal catalyst loading was conducted, where 1 mg mL^−1^ offered the highest production of H_2_O_2_ (ESI,† Fig. S108) over 2 mg mL^−1^ and 0.5 mg mL^−1^. Thus, production is not limited to catalyst concentration, since more catalyst resulted in less production due to Fenton decomposition of hydrogen peroxide.^110–112^ The influence of the temperature on the photoactivity of the catalyst was investigated, where higher temperatures resulted in more production (ESI,† Fig. S113). Fig. 3A depicts the kinetics for the photochemical production of H_2_O_2_*via* FeO_*x*_ NPs (1 h to 3 d), where production increased for the first 24 h and then stagnated for longer irradiation times (9.0 ± 0.4 mmol g^−1^ L^−1^ (5 h), 14.2 ± 1.1 mmol g^−1^ L^−1^ (20 h)). For future screenings, we opted for 5 h irradiation time at room temperature, as this yielded significant production of H_2_O_2_, which enabled quantification with peroxide test strips and titration. Furthermore, catalyst stability and recycling were also investigated, where the product solution was decanted off after each round of irradiation. We found that the catalyst could be recycled for up to four consecutive rounds (Fig. 3B, 20 h total). The catalyst material was stable for at least 6 months (including mixing of different batches) without loss of activity.

The apparent quantum yield (AQY) of the nanoparticles was measured *via* UV-Vis. After immobilization of the FeO_*x*_ photocatalyst on the side of a cuvette (Fig. 3C, inlet), illumination with certain wavelengths was performed. The absorption of 240 nm was followed over time (5 h), corresponding to formed hydrogen peroxide (Fig. 3C) with an AQY_365_ = 0.10% and AQY_445_ = 0.11%. The catalyst stability towards hydrogen peroxide was explored (ESI,† Fig. S112), where subjection to a solution of H_2_O_2_ (1 mM) and irradiation (445 nm) for 5 h had no effect on the absorbance of the UV-Vis absorption spectrum, nor led to leaching of the material from the glass surface into solution. Hence the photocatalytic production of hydrogen peroxide or stronger oxidizing conditions do not have a deleterious effect on the photochemical catalyst properties. Previous studies suggested to analyse the catalyst stability for 24 h, as earlier decomposition greatly reduces practical applications, which as indicated is not the case for the FeO_*x*_ NPs producing H_2_O_2_ for 3 d.^68^

Next, an extensive study on the quantification of H_2_O_2_ was conducted to cross-validate different quantification techniques,^113–117^ but also to confirm that solely H_2_O_2_ was formed, instead of other peroxides (*e.g.* autoxidation of unsaturated fatty acids). A method using high performance liquid chromatography-mass spectrometry (HPLC-MS) was developed, where H_2_O_2_ was found to leave the column at a retention time of 1.5 min, while amounts of oleic acid and its hydroperoxide could be separated and observed at 15–18 min (ESI-6.3,†). Utilizing cross-detection techniques of UV-DAD, MS and peroxide test strips, we confirmed a selectivity of >99% for the formation of H_2_O_2_. Thus, the formation of the allylic hydroperoxide of oleic acid and free (unligated) oleic acid was limited to trace amounts. Knowing that the photochemical oxygen reduction indeed led to H_2_O_2_ only, we further cross-validated UV-Vis iodometric quantification (ESI-6.4,†), UV-Vis of Ampliflu red in the presence of horseradish peroxidase towards resorufin (ESI-6.4,†), GC-MS (ESI-6.6,†), NMR (ESI-6.5,†), peroxide test strips (ESI-6.1,†) and iodometric titration (ESI-6.2,†).

### Condition screening and mechanism studies

**Fig. 4:**
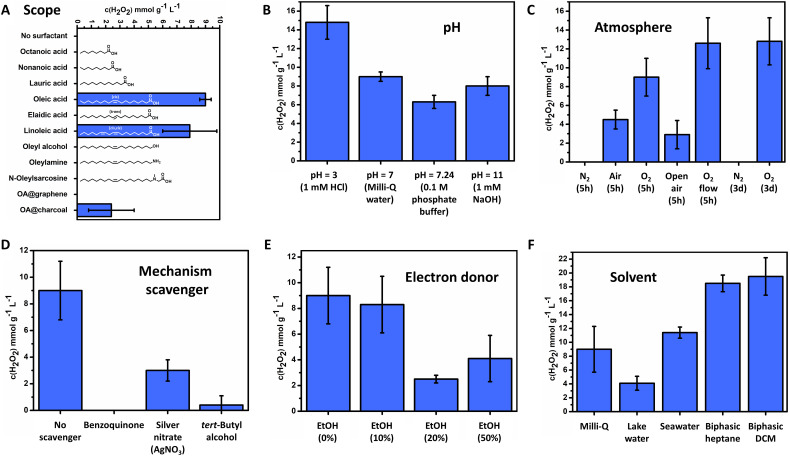
Production of H_2_O_2_ by FeO_*x*_ NPs, depending on (A) surfactant type; (B) pH value; (C) different atmospheres; (D) presence of active species scavengers (12.5 mol L^−1^); (E) presence of electron donor ethanol; (F) solvent. General reactions conditions: FeO_*x*_ NPs (1 mg mL^−1^), 4 mL solvent, O_2_ atmosphere (1 bar in 10 mL vial), LED OSRAM Oslon SSL 80 royal blue (500 mW, *λ* = 445 nm, 180 mW cm^−2^), 5 h, 293.15 K. H_2_O_2_ concentration values were obtained by iodometric redox titration. All yields are average values obtained from triplicate.

Only FeO_*x*_ NPs with oleic acid and linoleic acid ligands were able to catalyse the photoproduction of H_2_O_2_ (Fig. 4A, 9.0 ± 0.4 mmol g^−1^ L^−1^ for oleic acid, 7.9 ± 1.9 mmol g^−1^ L^−1^ for linoleic acid, 5 h irradiation). Thus, the synthesis of a broad surfactant scope could indicate properties and allowed for predictions towards the reaction mechanism for oxygen reduction. Immobilization of the catalyst (FeO_*x*_ NPs with oleic acid (2 : 1)) on different carbon materials (graphene, activated carbon) resulted in lowered activity, where accelerated recombination of charge carriers by enhanced conductivity could be an explanation. Utilizing oleyl alcohol or no surfactant resulted in microparticles instead of nanoparticles (ESI,† Fig. S36), indicating that small sizes of the nanoparticles (below 2 nm) enhance photoactivity through exciton transfer and quantum dot behavior.^107,118–122^ Nanoparticles with amines and alcohols were also found not to produce H_2_O_2_ (Fig. 4A). It is proposed that the NPs with amines or alcohols as a capping agent have different connectivity to the iron oxide surface than NPs with carboxylic acid^123^ and hence proper electron transfer to the active site is not warranted. Saturated fatty acids and unsaturated fatty acids with a *trans* double bond were all found to be inactive, suggesting a crucial role of the double bonds and their geometrical configuration (see oleic and linoleic acid) in the mechanism (Fig. 4A). No differences in the UV-Vis absorption spectrum between nanoparticles with *trans*-double bond elaidic and *cis*-double bond oleic acid could be observed, indicating no differences in its photochemical behaviour (ESI,† Fig. S83). However, elaidic acid NPs were not able to promote photocatalytic production towards hydrogen peroxide. Thus, the *cis* double bond of the nanoparticle bound surfactant (oleic acid and linoleic acid) is a crucial component to enable photocatalytic activity.

Elucidating the influence of pH on the FeO_*x*_-catalyzed H_2_O_2_ photoproduction revealed that production in Milli-Q water, phosphate buffer and 1 mM NaOH resulted in similar amounts of H_2_O_2_ production (6.3–9.0 mmol g^−1^ L^−1^). Hence basic conditions or buffer solutions do not enhance or lower production. An increased production of H_2_O_2_ was obtained in an acidic environment (14.8 ± 1.8 mmol g^−1^ L^−1^ (pH = 3)). The low pH ensures more protons in solution, stabilizing H_2_O_2_ and decreasing the Fenton process, thus improving the balance between H_2_O_2_ formation and decomposition (Fig. 4B and 2G).^124^

The FeO_*x*_ catalyst was then investigated in different atmospheres. As shown in Fig. 4C the H_2_O_2_ concentration decreased when switching from O_2_ (100%, 5 h, standard conditions) to air (21% O_2_). A nitrogen atmosphere (5 h and 3 d) resulted in no production of H_2_O_2_, which suggests that a water oxidation reaction (WOR) is not part of the FeO_*x*_ catalyst system. Knowing that the FeO_*x*_ material acts solely as an ORR catalyst, we envisioned open air and constant oxygen bubbling to increase the productivity. As only a slight increase in production was observed, the previous preparation method already ensured sufficient oxygen saturation and concentration. Long-term irradiation was conducted for three days, which only increased the production of H_2_O_2_ for about 1.4 times, while the irradiation time was about 13 times as long. This fact stresses the mismatch between the WOR and ORR, also suggesting that long-term irradiation studies do not necessarily lead to complete catalyst deactivation towards zero production of H_2_O_2_. The influence of stirring bars was examined by performing the reaction with Teflon and glass stirring bars added as well as without; no significant difference was observed as production was not affected by stirring or trace metals as new stirring bars were utilized (ESI,† Fig. S115). Active species trapping experiments of superoxide radicals (˙O_2_^−^), electrons (e^−^) and hydroxyl radicals (˙OH) by *p*-benzoquinone (BQ), silver nitrate (AgNO_3_) and *tert*-butyl alcohol, respectively, were conducted (Fig. 4D and ESI,† Schemes S2, S3)).^70^ These experiments should always be performed to check which active intermediates are participating in the H_2_O_2_ production mechanism. When AgNO_3_ and *tert*-butyl alcohol were added, H_2_O_2_ production dropped significantly, indicating the presence of electrons (e^−^) and hydroxyl radicals (˙OH) in the reaction pathway (3.0 ± 0.8 mmol g^−1^ L^−1^ (AgNO_3_), 0.4 ± 0.7 mmol g^−1^ L^−1^ (TBA)). Zero production of H_2_O_2_ was observed when BQ was present, stressing the importance of superoxide (˙O_2_^−^) and thus an indirect ORR pathway for the mechanism. These results confirm again the adequate band gap and its reaction pathway (Fig. 2G). Hence, superoxide radicals, electrons and hydroxyl radicals are all actively taking part in the mechanism of FeO_*x*_-catalysed photochemical H_2_O_2_ production.

In the literature, higher productions/productivities are often reported with the addition of sacrificial agents through filling of excess holes, produced by a photochemical mismatch in the WOR and ORR.^98^ Since the FeO_*x*_ NPs nanoparticles were lacking the WOR, we opted for the addition of an electron donor (EtOH) to improve production and mismatch. However, as depicted in Fig. 4E, no significant increase was obtained. Other sacrificial agents investigated (methanol, isopropanol, benzyl alcohol) were also not compatible (ESI-8.2,†). An extensive evaluation of sacrificial agents was conducted by exposing sacrificial agents to air overnight, where it was found that benzyl alcohol and isopropanol produced peroxides.^125–128^ These findings are in line with previously reported non-innocent auto-photocatalytic oxidation of benzyl alcohol to benzaldehyde, where large quantities of H_2_O_2_ are produced upon irradiation.^2,129,130^ Therefore, we opted for only methanol and ethanol as trustworthy and biobased sacrificial agents. Methanol and ethanol did not inherently produce hydrogen peroxide in contact with oxygen by autoxidation.

A solvent screening was performed on biphasic systems and naturally occurring water resources, as shown in Fig. 4F. To our delight we obtained the production of H_2_O_2_ both in lake water and seawater, without any purification other than filtration (4.1 ± 1.0 mmol g^−1^ L^−1^ (lake water), 11.4 ± 0.8 mmol g^−1^ L^−1^ (seawater)). The FeO_*x*_ NPs were thus able to perform the ORR in the presence of salts and other impurities present in lake and seawater.

We purposely decided against benzyl alcohol as a hole scavenger and ‘advantageous system’ for biphasic product (H_2_O_2_) separation from the active site of the catalyst, due to its inherent capability to absorb photons and undergo autoxidation (ESI-8.2,†). Heptane (0.6795 g cm^−3^) and DCM (1.3266 g cm^−3^) were chosen for biphasic systems as these do not undergo autoxidation and are immiscible with Milli-Q water (0.99705 g cm^−3^) (ESI-9.1,†). Significantly higher production was achieved (18.5 ± 1.2 mmol g^−1^ L^−1^ for heptane, 19.5 ± 2.7 mmol g^−1^ L^−1^ for DCM), the reason being circumvention of Fenton degradation through separation of the produced H_2_O_2_ and ˙O_2_^−^ from the catalyst surface, as the catalyst dissolved in heptane and DCM while H_2_O_2_ migrated to the water layer.

Furthermore, the possibility of a cation-enhancement effect using various metals (Zn^2+^, Al^3+^, Ni^2+^, Fe^2+^, Fe^3+^) was investigated. Several iron salts and oxides (iron(ii) sulfate heptahydrate, iron(ii) chloride, iron(iii) sulfate hydrate, iron(iii) chloride and iron(iii) nitrate nonahydrate and iron oxide (Fe_2_O_3_ and Fe_3_O_4_)) were investigated as additives resulting in zero production of H_2_O_2_ as Fenton chemistry was enhanced.^131^ The addition of zinc, aluminium and nickel salts to the reaction mixture was found to be tolerated for performing the ORR by the FeO_*x*_ photocatalyst (5.2–8.3 mmol g^−1^ L^−1^). Interestingly, the addition of aluminium oxide (Al_2_O_3_) resulted in significantly increased production (15.5 ± 4.3 mmol g^−1^ L^−1^). Addition of pure sodium salts (in contrast to the salt mixtures in seawater) was found to significantly reduce production, probably through poisoning of active sites (ESI-9,†).^132^ In direct synthesis, these sodium salts are usually added in order to improve selectivity presumably by blocking of sites for O–O cleavage.^133^ This effect was however not found for the iron oxide nanoparticles.

### Proposed photochemical oxygen reduction mechanism

After having established that the FeO_*x*_ photocatalyst exclusively promotes oxygen reduction towards H_2_O_2_, we confirmed those findings further *via* Headspace GC-TCD (thermal conductivity detector). Neither O_2_ nor H_2_ formation was observed, indicating the absence of proton reduction as well as water oxidation for our catalyst system (ESI-8.1,†).

**Fig. 5:**
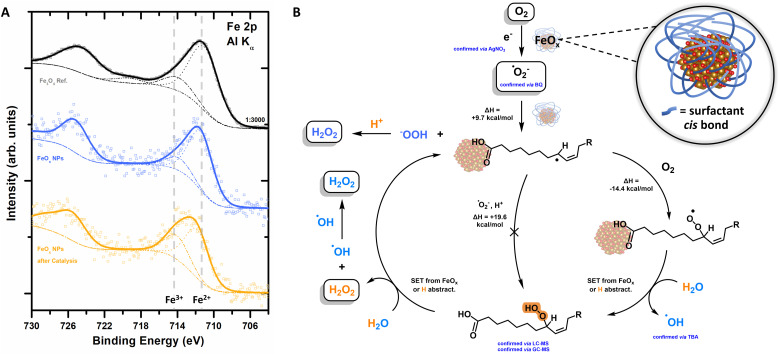
(A) XPS spectra of the Fe 2p core level region of the as-synthesised nanoparticles, black: FeO_*x*_ without surfactant, blue: FeO_*x*_ NPs before photochemical production of H_2_O_2_, orange: inactive FeO_*x*_ NPs after 8 consecutive rounds of catalysis. (B) Proposed mechanism for FeO_*x*_ promoted photochemical oxygen reduction to H_2_O_2_; inset: proposed active site. Energy barriers are calculated without the effect of the FeO_*x*_ core.

XPS analysis revealed that the electrons for the photochemical oxygen reduction are provided by the FeO_*x*_ catalyst material, as depicted in Table S10 (ESI†) and Fig. 5A. Fe^2+^ (2p photoemission line peaked at binding energy (BE) of 711.7 eV) species are oxidized to Fe^3+^ (BE = 714.3 eV) over time, transforming the Fe_3_O_4_ resembling catalyst (BE = 709.9 eV) into Fe_2_O_3_ (BE = 711.1 eV) (Fig. 5A).^134–137^ These observations are also corroborated by the loss of magnetism over consecutive rounds of catalyst recycling until deactivation (Fig. 3B). The *cis* double bond, playing a role as a co-catalyst, is also oxidized over time (C 1s peak at a BE of 286.5 eV, ESI,† Fig. S120). The general oxygen ratio is increasing due to oxidative conditions (ESI,† Fig. S119).^134,138,139^ Thus, the catalyst becomes inactive after four rounds of catalysis, *i.e.* ORR, as holes generated are not filled by WOR or sacrificial agents (Fig. 4C and E). Catalyst reactivation was attempted by reattaching new surfactant molecules but proved to be ineffective. Future research will focus on the implementation of the FeO_*x*_ photocatalyst as a photoelectrodic material to not only achieve higher oxygen reduction rates to H_2_O_2_ but also avoid oxidation.^89,140–142^

The extensive studies of the process led to the proposed mechanism depicted in Fig. 5B, which exhibits similar characteristics as the peroxidase and cyclooxygenase reaction of arachidonic acid (AA, *cis*) to Prostaglandin G_2_.^143–146^ H_2_O_2_ is produced photochemically with a selectivity of >99% (LC-MS) *via* the oxygen reduction reaction (ORR, atmosphere experiments).

Active species trapping experiments with AgNO_3_ indicate the presence e^−^ (Fig. 4D), which are formed by photoexcitation of the photocatalyst (Fig. 2G). With these electrons, oxygen undergoes an indirect reduction mechanism towards superoxide ˙O_2_^−^ (trapping experiments with BQ, Fig. 4D). The superoxide is able to attack the surfactant at the double bond forming first an allylic radical, followed by trapping of this allylic radical with molecular triplet oxygen to form an allylic peroxyl radical (Fig. 5B). A (i) subsequent single electron transfer (SET) event with either the FeO_*x*_ core or with the solvated electrons; or (ii) subsequent hydrogen abstraction reaction of this peroxyl radical with water, both yield the hydroperoxide (intermediate trace amounts, confirmed by LC-MS and GC-MS) and hydroxyl radicals whose presence was confirmed by active species trapping with *tert*-butyl alcohol (Fig. 4D). The final cleavage of the hydroperoxide from the surfactant and regeneration of the catalytically active fatty acid allyl radical is also thought to proceed *via* either (i) SET event or (ii) hydrogen abstraction from the water solvent (calculated by density functional theory (DFT), ESI-8.5 and ESI-8.6,†). The presence of iron is crucial for the catalytic cycle proposed in Fig. 5B, indicating an active role of iron for adsorption and subsequent desorption from the catalyst surface.^147,148^ The active site (Fig. 5B inset) therefore seems to consist of iron oxide (Fe_3_O_4_, Fe_oct_^2+^) connected to a carboxylic acid, which in proximity of the *cis* double bond forms a hydrophobic pocket favourable for oxygen affinity.^87,149,150^ Here, protons could possibly be supplied by (carboxylic) acids or water (Fig. 4B), while electron transfer is feasible from iron *via* its connectivity with the surfactant.^151^

From XRD and DLS measurements (ESI,† Table S1) it was found that particle sizes smaller than 2 nm (below the exciton Bohr radius) with a certain crystallinity were necessary for photoactivity through exciton transfer, while also TEM confirmed these small and round particles.^118–120,152^ This interplay between factors seems to be crucial for photochemical oxygen reduction activity towards hydrogen peroxide *via* FeO_*x*_.

### Applications and *in situ* oxidations

After elucidating the catalytic properties, the oxygen reduction mechanism and the conditions for optimal photochemical production of hydrogen peroxide, we applied the FeO_*x*_ NPs in several organic transformations and real-life conditions.

**Scheme 1:**
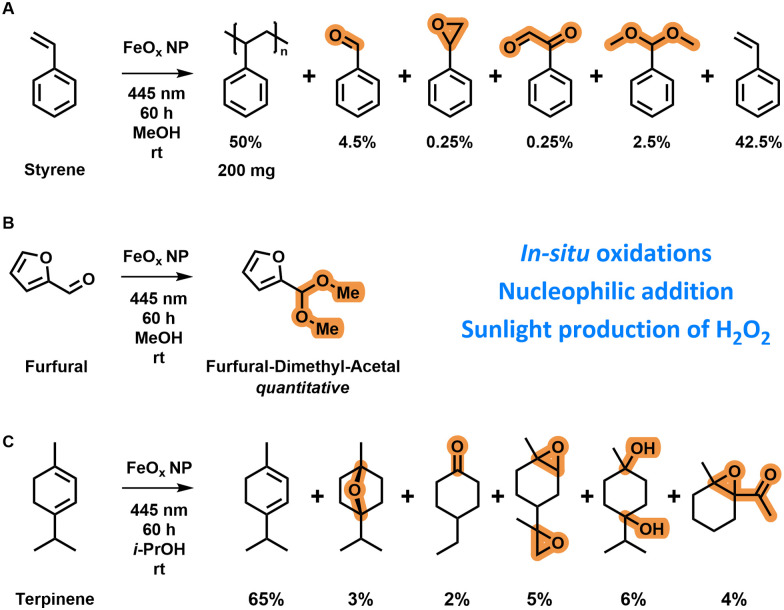
(A) Oxidation and polymerization of styrene (0.4 g, distilled) in methanol (2 mL) after 60 h of light irradiation (445 nm) in the presence of FeO_*x*_ NPs (4 mg). (B) Nucleophilic addition reaction on furfural (30 μL, 362 μmol) to its dimethyl acetal after 60 h in oxygenated methanol (4 mL) by irradiation of FeO_*x*_ NPs (4 mg) at 445 nm. (C) *In situ* oxidation of α-terpinene (0.15 mL) towards a mixture of products after 60 h in oxygenated isopropanol (5 mL) by irradiation of FeO_*x*_ NPs (5 mg) at 445 nm. Product ratios based on GC-MS and ^1^H-NMR.

Styrene is normally polymerized *via* free radical polymerization techniques initiated by the addition of a radical initiator (*e.g.* benzoyloxy peroxide).^153^ The O–O bond is cleaved at elevated temperatures when a peroxide initiator is used to form radicals for initiation (ESI-10.1,†). *In situ* polymerization of styrene (0.4 g, distilled) in methanol (2 mL) with iron oxide nanoparticles (4 mg) was possible after three days of irradiation by blue light (445 nm) (Scheme 1A) as indicated by the formation of a polystyrene solid in the reaction vial (ESI,† Scheme S5). This observation shows that hydrogen peroxide was formed and subsequently decomposed *via* photo-Fenton reactions towards radical species; these formed radical species were finally able to polymerize styrene. The polymerization reaction was slow as the polymer was only observed after three days. Oxygen present in the vial is known to inhibit free radical polymerization by reaction with active radicals.^154^ Other products formed were benzaldehyde, styrene oxide, benzene acetaldehyde and a dimethyl acetal as confirmed by GC-MS. A nucleophilic addition reaction was conducted on furfural (30 μL, 362 μmol) in oxygenated methanol (4 mL) by irradiation with 445 nm light catalysed by FeO_*x*_ NPs (4 mg) (Scheme 1B). After 60 h, quantitative production of the dimethyl acetal of furfural was observed *via*^1^H-NMR (ESI,† Fig. S124). This reaction confirmed the absence of singlet oxygen in the mechanism as O=O would have led to the formation of hydroxybutenolide *via* [4+2] cycloaddition.^82^ Interestingly, with 17 046 mmol g^−1^ L^−1^ of dimethyl acetal formed, a 1812× increase of H_2_O_2_ production was achieved (ESI-10.2,†), demonstrating that performing *in situ* reactions results in higher productivities because Fenton-decomposition is circumvented through direct reaction with organic substrates. An *in situ* oxidation reaction of α-terpinene (0.15 mL) in oxygenated isopropanol (5 mL) was catalysed by FeO_*x*_ NPs (5 mg) when irradiating with 445 nm light (Scheme 1C). Also here, no singlet oxygen was formed, as there were no [4+2] cycloaddition products identified.^80^ The products of the *in situ* oxidations catalysed by FeO_*x*_ NPs consisted of epoxides, ketones, ethers and alcohols. In total 7377 mmol g^−1^ L^−1^ of products were formed, which compared to previously investigated hydrogen peroxide production (5 h, 9.4 ± 1.3 mmol g^−1^ L^−1^) is a 784× increase concentration-wise. Again, in all these application reactions only benign solvents, room temperature and photoirradiation were utilized as sustainable conditions.^155^*In situ* oxidation of *o*-tolidene was also attempted as a quantification method *via*^1^H-NMR; however too little hydrogen peroxide was produced, not reaching the detection limit (ESI,† Fig. S107).

A long-term irradiation experiment in real sunlight was conducted for 1 week. This was performed by putting a sample in a window, where it had approximately 8 h of sunlight daily (performed in September facing south in Groningen, NL, ESI-5.3,†). Photochemical production of H_2_O_2_ was also possible by 1 week irradiation by sunlight. Thus, ultimately a very sustainable and interesting process was achieved: iron oxide promoted photochemical oxygen reduction to hydrogen peroxide from sunlight in seawater.

## Conclusion

Here, we established a strategy for the photochemical production of H_2_O_2_ catalysed by iron. Following the 12 principles of Green Chemistry we developed a sustainable synthesis for an entire scope of earth-abundant iron oxide nanoparticles terminated with biobased fatty acid surfactants. These materials were fully characterized and investigated for the photochemical production of H_2_O_2_. Specifically, FeO_*x*_ NPs with fatty acid ligands possessing *cis* double bonds confer photoactivity for oxygen reduction to hydrogen peroxide. An extensive study for peroxide quantification revealed a selectivity of >99% for H_2_O_2_. Acting as an oxygen reduction material, the synthesized nanoparticles could be recycled for up to four consecutive rounds of 5 h irradiation. Through detailed experimental investigation of the catalyst properties and computational results, a mechanism and an active site were proposed for photochemical hydrogen peroxide production (Fig. 5). The H_2_O_2_ yield could be increased by decreasing the pH, addition of cation exchangers and by production in biphasic systems (heptane/DCM with Milli-Q water) (up to 19.5 ± 2.7 mmol g^−1^ L^−1^). Here a lower pH supplied protons, aluminium oxide facilitated electron transfer and biphasic systems circumvented Fenton-decomposition by separation of the product from the catalyst. The iron oxide nanoparticles with oleic acid (2 : 1) as a surfactant were also successfully utilized in other applications like wastewater treatment, polymerizations and *in situ* oxidations.

Crucially, the FeO_*x*_ NPs could be synthesized from biobased and abundant materials. Oleic acid is the main component in olive oils, ethanol is obtained from biomass and iron is the most abundant metal in the earth's crust. An apparent quantum yield (AQY) of 0.11% was achieved for photochemical oxygen reduction to H_2_O_2_ at ambient temperatures and pressures upon irradiation of the FeO_*x*_ catalyst with visible light (445 nm; 9.4–14.8 mmol g^−1^ L^−1^). Productivities to H_2_O_2_ of at least 1.7 ± 0.3 mmol g^−1^ L^−1^ h^−1^ were obtained. Production of H_2_O_2_ was possible *via* sunlight irradiation and in seawater.

This study demonstrates the importance of the development of sustainable catalyst materials if the replacement of outdated industrial processes is the ultimate goal. Utilizing earth-abundant metals and biobased (co-)catalysts offers great potential for the photocatalytic production of hydrogen peroxide as a solar fuel. The results presented here may open a new avenue for designing suitable and green photocatalysts for efficient H_2_O_2_ production from solar energy.

## Author contributions

B. L. F. conceptualized the research project and coordinated it with the help of J. S.. T. F. and J. T. M. conducted the research and its validation. M. B. B., M. C. A. S., R. T. and D. G. conducted validation experiments with input from P. R. and J. N. H. R.. G. A. performed the DFT studies. T. F. and B. L. F. prepared the manuscript with input from J. T. M., M. B. B., G. A.. All authors reviewed the manuscript.

## Conflicts of interest

There are no conflicts to declare.

## Supplementary Material

EY-002-D3EY00256J-s001
