# Supplementary material for: Iron oxide-promoted photochemical oxygen reduction to hydrogen peroxide (H2O2)
Source: EES Catal. 2023 Nov 24;2(1):262–75. doi: 10.1039/d3ey00256j (PMC10782808; doi:10.1039/d3ey00256j)
Supplement: EY-002-D3EY00256J-s001 [file EY-002-D3EY00256J-s001.pdf]

Electronic Supplementary Material (ESI) for EES Catalysis.
This journal is © The Royal Society of Chemistry 2023

Supplementary Information

for

Iron oxide promoted photochemical oxygen reduction to hydrogen peroxide

Thomas Freese,[a] Jelmer T. Meijer,[a] Marie Brands,[b] Georgios Alachouzos,[a] Marc C. A. Stuart,[c] Rafael Tarozo,[a] Dominic Gerlach,[d] Joost Smits,[e] Petra Rudolf,[d] Joost N. H. Reek,[b] and Ben L. Feringa*[a]

a. Stratingh Institute for Chemistry, University of Groningen, Nijenborgh 4, 9747AG Groningen, The Netherlands

b. van’t Hoff Institute for Molecular Sciences, University of Amsterdam, Science Park 904, 1098XH Amsterdam, The Netherlands

c. Electron Microscopy, Groningen Biomolecular Sciences and Biotechnology Institute, University of Groningen, Nijenborgh 7, 9747AG Groningen, The Netherlands

d. Zernike Institute for Advanced Materials, University of Groningen, Nijenborgh 4, 9747AG Groningen, The Netherlands

e. Shell Global Solutions International BV, Grasweg 31, 1031HW Amsterdam, The Netherlands

E-Mail: b.l.feringa@rug.nl

Table of Contents

1. Materials and methods 5

1.1 Reagents and solvents 5

2. Equipment and general analytical information 7

3. Experimental procedures 9

3.1 Catalyst synthesis in dibutyl ether and optimization 9

3.2 Catalyst synthesis optimization 10

3.3 Catalyst synthesis in ethanol 11

3.3.1 Synthesis of iron oxide nanoparticles without surfactant 12

3.3.2 Synthesis of iron oxide nanoparticles with oleic acid 12

3.3.3 Synthesis of iron oxide nanoparticles with oleic acid: troubleshooting 15

3.3.4 Synthesis of iron oxide nanoparticles with elaidic acid 17

3.3.5 Synthesis of iron oxide nanoparticles with linoleic acid 18

3.3.6 Synthesis of iron oxide nanoparticles with octanoic acid 19

3.3.7 Synthesis of iron oxide nanoparticles with nonanoic acid 20

3.3.8 Synthesis of iron oxide nanoparticles with lauric acid 21

3.3.9 Synthesis of iron oxide nanoparticles with stearic acid 22

3.3.10 Synthesis of iron oxide nanoparticles with oleyl alcohol 23

3.3.11 Synthesis of iron oxide nanoparticles with oleylamine 23

3.3.12 Synthesis of iron oxide nanoparticles with N-oleylsarcosine 25

3.3.13 Synthesis of FeO_x NPs with oleic acid supported on active charcoal (mesh 20-40) 26

3.3.14 Synthesis of FeO_x NPs with oleic acid supported on active charcoal (mesh 100) 27

3.3.15 Synthesis of FeO_x NPs with oleic acid supported on graphene nanoplatelets 28

3.3.16 Immobilization on TiO_2, lignin, cellulose and chitosan 29

4. Catalyst properties 30

4.1 DLS results 30

4.1.1 FeO_x scope comparison 30

4.2 Transmission electron microscopy (TEM), scanning transmission electron microscopy (STEM) and energy-dispersive X-ray spectroscopy (EDX) of FeO_x NPs 32

4.2.1 FeO_x NP with oleic acid (*cis*) 33

4.2.2 FeO_x NP with linoleic acid (*cis*,*cis*) 35

4.2.1.2 FeO_x NPs with oleic acid immobilized on active charcoal mesh 20-40 37

4.2.1.3 FeO_x NPs with oleic acid immobilized on active charcoal mesh 100 39

4.2.1.4 FeO_x NPs with oleic acid immobilized on graphene nanoplatelets 41

4.2.5 FeO_x without surfactant 42
4.2.6 FeO_x NPs with octanoic acid 43
4.2.7 FeO_x NPs with nonanoic acid 44
4.2.8 FeO_x NPs with lauric acid 45
4.2.10 FeO_x NPs with oleyl alcohol 47
4.2.11 FeO_x NPs with oleylamine (1:1) 48
4.2.12 FeO_x NPs with oleylamine (2:1) 49
4.2.13 FeO_x NPs with oleylamine (3:1) 50
4.2.14 FeO_x NPs with N-oleyl sarcosine 51
4.3 XRD 52
4.4 Elemental analysis 56
4.5 UV-Vis 57
4.5.1 FeO_x scope comparison 57
4.5.2 Comparison FeO_x active materials: oleic acid (*cis*), linoleic acid (*cis*,*cis*) with inactive elaidic acid (*trans*) 57
4.5.3 FeO_x NP scope 58
4.6 Tauc plot 60
4.7 Electrochemical Mott-Schottky measurements 61
5. Irradiation studies 63
5.1 Batch photocatalytic oxygen reduction 63
5.2 Rotary photoreactor (PhotoVap) 64
5.3 Real sunlight 64
5.4 Screening and batch irradiation of NPs synthesized in dibutyl ether 65
5.5 Screening and batch irradiation of NPs synthesized in ethanol 65
5.6 Experimental procedure for blank reactions and results 67
6. Quantification of hydrogen peroxide 72
6.1 Peroxide test strips 72
6.2 Iodometric titration 73
6.2.1 Stock solution preparation 73
6.2.2 Quantification procedure 74
6.2.3 Troubleshooting iodometry 74
6.3 HPLC-MS 75
6.4 Qualitative validation *via* UV-Vis (H_2O_2, resorufin and iodometric assay) 80
6.5 ^{1}H-NMR spectroscopy 81
6.6 GC-MS 82
6.6.1 Derivatization procedure (esterification and silylation) 82

7. Catalyst performance 83
7.1 List of photochemical H_2O_2 production values 84
7.2 Catalyst recycling 87
7.3 Synthesis and production consistency 88
7.4 Apparent quantum yield (AQY) measurements 89
7.5 Catalyst stability 90
7.6 Temperature dependency 90
8. Mechanism studies 91
8.1 Headspace GC analysis 95
8.2 Sacrificial electron donors (hole scavengers) 97
8.3 Catalyst reactivation 97
8.4 XPS Analysis 98
8.5 DFT calculations 103
8.6 Proposed mechanism 103
9. Additives (cation exchangers, salts, acids, biphasic systems) 104
9.1 Solvents and biphasic systems 104
10. Applications 105
10.1 Styrene polymerization and oxidation 105
10.1.1 Experimental procedure styrene oxidation 106
10.2 Furfural – nucleophilic addition 106
10.2.1 Experimental procedure furfural oxidation 106
10.3 α-terpinene oxidation 106
10.3.1 Experimental procedure α-terpinene oxidation 106
10.4 Wastewater treatment for degradation of methylene blue (MB) 107
10.5.1 Experimental procedure wastewater treatment 107
11. DFT Data 108
12. ^{1}H-NMR spectra 116
14.1 Furfural-dimethyl-acetal 116
14.2 Surfactants and reactivity with oxygen 117
13. GC-MS 122
15.1 Styrene oxidation in ethyl acetate 122
15.2 Styrene oxidation in methanol 124
15.3 Furfural nucleophilic addition 129
15.4 α-terpinene oxidation 130
15.5 GC-MS analysis for confirmation of oleic acid hydroperoxide 137
14. References 139

1. Materials and methods

1.1 Reagents and solvents

Commercial reagents and solvents: Unless stated otherwise, all reagents and solvents were obtained from the commercial sources: Sigma–Aldrich, TCI, Boom and Linde-gas and were used as received. For aqueous solutions, Milli-Q water was used.

The following chemicals were purchased from *Sigma Aldrich*:

Iron (0) pentacarbonyl (Sigma Aldrich, 99.99%, SHBN5572, 09-05-22), linolenic acid (Sigma Aldrich, 70%, BCCD7126, 19-08-22), oleic acid (Sigma Aldrich, 90%, MKCL2492, 21-07-20), trimethylamine N-oxide dihydrate (Sigma Aldrich, 98%, BCCF8795, 08-07-22), silver nitrate (Sigma Aldrich, 99%, MKCR0415, 15-09-22), *p*-benzoquinone (Sigma Aldrich, 98%, BCCG7061, 15-09-22), acetic acid (Sigma Aldrich, 100%, K52550263 025, 24-09-21), hydrochloric acid (Sigma Aldrich, 37%, STBK5583, 20-06-22) stearic acid (Sigma Aldrich, 95%, SHBN3646, 05-07-22), lauric acid (Sigma Aldrich, 98%, SHBN9628, 23-11-22), octanoic acid (Sigma Aldrich, 99%, MKCQ5948, 15-07-22), linoleic acid (Sigma Aldrich, 95%, SHBP0710, 06-07-22), graphene nanoplatelets (25 µm particle size, surface area 150 m^2 g^{-1}, Sigma Aldrich, MKCM5180, 11-02-22), activated charcoal (100 mesh particle size, Sigma Aldrich, SHBN4347, 22-02-22), dibutyl ether (Sigma Aldrich, 99.3%, STBK3168, 09-06-22), nonanoic acid (Sigma Aldrich, 97%, 055H0302V, 15-07-22), iron(II) chloride (Sigma Aldrich, 0000124564, 11-02-22), iron(III) chloride (Sigma Aldrich, 97%, STBK3467, 15-09-22), oleylamine (Sigma Aldrich, 70%, STBK0863, 11-02-22), activated charcoal 20-40 mesh particle size (Sigma Aldrich, SHBL6345, 11-02-22), iron(III) oxide (Sigma Aldrich, MKCH7591, 27-01-21), aluminum oxide (Sigma Aldrich, BCCG6349, 11-02-22), iron(III) nitrate (Sigma Aldrich, 98%, MKCN0770, 11-02-22), sodium bromide (Sigma Aldrich, 99%, MKCP4918, 11-02-22), iron (III) sulfate hydrate (Sigma Aldrich, 97%, STBK2602, 11-02-22), zinc nitrate hexahydrate (Sigma Aldrich, 98%, BCCG2165, 11-02-22), aluminum nitrate nonahydrate (Sigma Aldrich, 98%, MKCN0704, 15-02-22), starch (Sigma Aldrich, SLCC5527, 21-01-21), sodium thiosulfate (Sigma Aldrich, 99%, BCCC5894, 21-01-21), benzyl alcohol (Sigma Aldrich, 99%, STBK7155, 05-07-22), iron (II,III) oxide (Sigma Aldrich, 97%, MKCL0694, 27-01-21), iron (II) sulfate heptahydrate (Sigma Aldrich, 99%, MKCN7636, 15-02-22), nickel(II) chloride hexahydrate (Sigma Aldrich, BCCG4504, 11-02-22), copper(II) chloride dihydrate (Sigma Aldrich, MKCL5412, 11-02-22), oleyl alcohol (Sigma Aldrich, 85%, MKCQ9938, 23-08-22), potassium Iodide (Sigma Aldrich, 99.5%, STBJ7197, 21-01-21), ammonium molybdate tetrahydrate (Sigma Aldrich, 81-83%, SLCH3187, 21-01-21), sodium hydroxide (Sigma Aldrich, 98%, SLCC5278, 07-01-20), phosphate buffered saline (Sigma Aldrich, SLCF2176, 23-12-20), α-terpinene (Sigma Aldrich, 85%, SHBL8464, 13-10-22), furfuryl alcohol (Sigma Aldrich, 98%, MKCJ0857, 01-07-22), styrene (distilled before use, Sigma Aldrich, 99%, STBH7369, 18-02-19), mesitylene (Sigma Aldrich, 98%, BCCN2445, 27-11-19), potassium hydroxide (Sigma Aldrich, 85%, MKCJ6734, 18-12-19), methylene blue (173820).

The following chemicals are purchased from *Boom B.V.*:

Sulfuric acid (Boom B.V., 95-97%, 26-10-22), n-heptane (Boom B.V., 99%, UN1206, 14-09-22), ethanol (Boom B.V., 100%, EA99-4422-10SD, 16-12-22), sodium chloride (Boom B.V., 03-05-21), ammonia solution (Boom B.V., 25%, 14-09-21),

The following chemicals are purchased from *Acros Organics*: Iron (0) pentacarbonyl (Acros Organics, A0425102, 24-12-20), purchased from *TCI*: Elaidic acid (TCI, 97%, J6PVI-OK, 15-11-22), purchased from *BLD Pharmatech ltd.*: N-oleylsarcosine (BLD Pharmatech Ltd., Total Nitrogen 3.2%, CMC314, 08-09-22), purchased from *Macron*: Dichloromethane (Macron, UN1593, 13-07-22), methyl alcohol anhydrous (Macron, 2217805858, 28-11-22), isopropyl alcohol (Macron, 13-05-22), purchased from *Merck*: Potassium dichromat (Merck, 09-01-13), purchased from *Linde-gas*: Oxygen compressed 5,0 (technical grade, SOL SpA, S161280921X01247DI, 01-09-22).

Real lake water (2 L) was collected physically from Wippinger Kolk, 52°54'53.6"N 7°23'45.6"E (52.914897, 7.395993), 26892 Wippingen, Lower Saxony, Germany on 6th March 2022 at 17:47. Remaining sand and animals were filtered off and the water used without further purification. It was stored in darkness at room temperature.

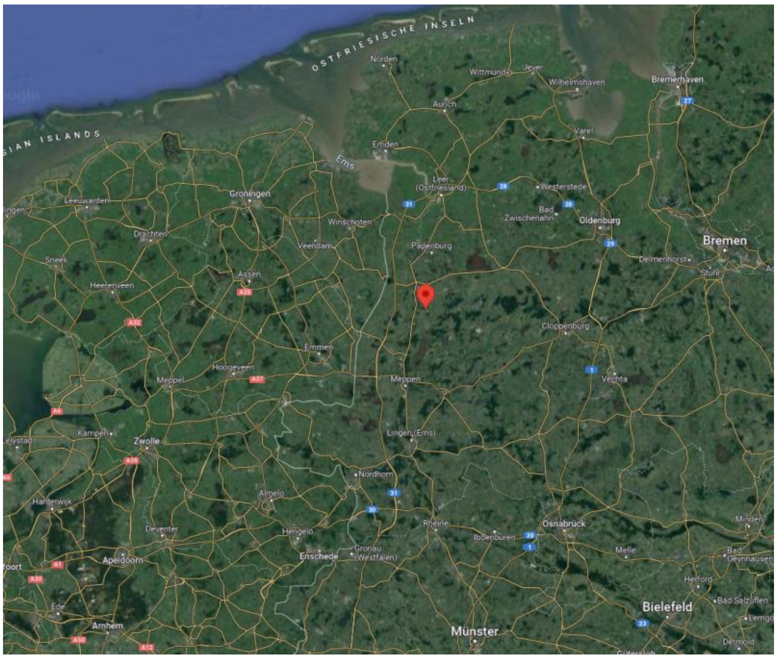

Figure S1: Location of Wippinger Kolk, 52°54'53.6"N 7°23'45.6"E (52.914897, 7.395993), 26892 Wippingen, Lower Saxony, Germany for the collection of lake water.

Real seawater (2 L) was collected physically from the North Sea, 52°46'20.2"N 4°39'16.0"E (N52.772285, E4.654436) 1755 Petten, Noord Holland, The Netherlands on 4th March 2022 at 20:08. Remaining sand and animals were filtered off and the water used without further purification. It was stored in darkness at room temperature.

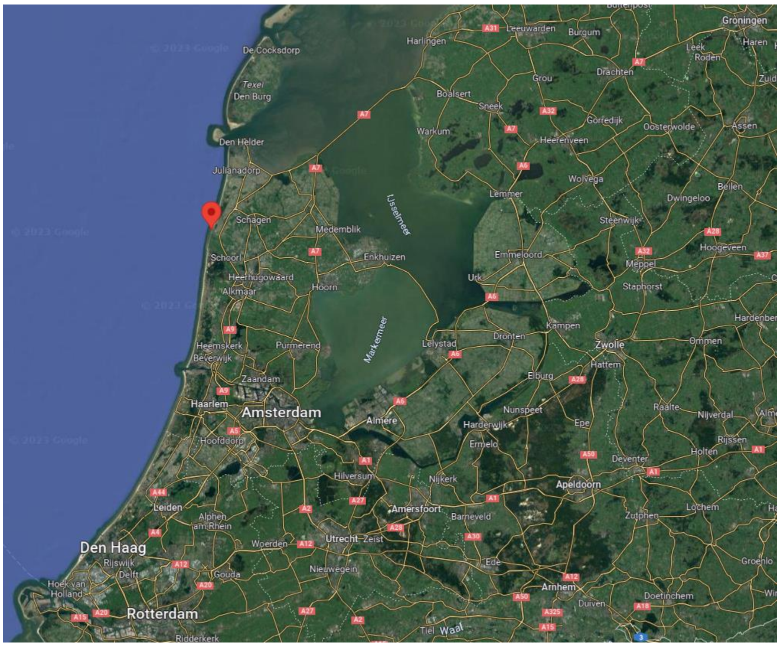

Figure S2: Location of North Sea, 52°46'20.2"N 4°39'16.0"E (N52.772285, E4.654436) 1755 Petten, Noord Holland, The Netherlands for the collection of seawater.

2. Equipment and general analytical information

Photochemical equipment:

- white light LED lamps (575 lm, 8 W each; 5750 lm, 80 W total) as light source for the rotary photoreactor.
- LEDs (OSRAM Oslon SSL 80 royal blue, LDCQ7P-2U3U, 500 mW, λ = 445 nm, 180 mW/cm^2) as light source for batch and flow production of hydrogen peroxide.

General Analytical Information: Nuclear Magnetic Resonance spectra were measured with an Agilent Technologies 400-MR (400/54 Premium Shielded) spectrometer (400 MHz). All spectra were measured at room temperature (22–24 °C). Chemical shifts for the specific NMR spectra were reported relative to the residual solvent peak [in ppm; $CDCl_3$: δH = 7.26; $CDCl_3$: δC = 77.16]. The multiplicities of the signals are denoted by s (singlet), d (doublet), t (triplet), q (quartet), m (multiplet), br s (broad signal), app (apparent). All ^{13}C-NMR spectra are ^{1}H-broadband decoupled.

High-resolution mass spectrometric measurements were performed using a Thermo scientific LTQ OrbitrapXL spectrometer with electrospray ionization. The molecular ion (M+, [M + H]+ and [M–X]+) is given in m/z-units.

UV-vis spectra were recorded with an Agilent 8543 spectrophotometer. The Agilent 8453 UV-Visible spectrometer was equipped with a custom-built (Prizmatix/Mountain Photonics) multi-wavelength fiber coupled LED-system (FC6-LED-WL) including the following LEDs: 365A, 390B, 420Z, 445B, 535R, 630CA. A detailed description of the setup was published earlier by our group (see Figure S1 in reference 1).[1] A Quantum Northwest TC1 temperature controller was used to maintain the temperature at 20 °C during photochemical studies.

Electrochemical measurements were carried out on a Zahner Zennium & PP211 workstation (Zahner, Germany). More detailed information on the Mott-Schottky measurements can be found in Section SI4.6.

Dynamic light scattering (DLS) experiments were performed with a Zetasizer Ultra Red (Malvern Panalytical, ZSU3305).

A Tecnai T20 cryo-electron microscope with 200 keV was used to take the Transmission electron microscopy (TEM) and scanning transmission electron (STEM) images. Energy-dispersive X-ray spectroscopy (EDX) was performed with a silicon drift energy dispersive X-ray (SDD EDX) detector X-max from Oxford Instruments. The elemental ratio was calculated *via* INCA software.

A Bruker D8 Advance diffractometer was used for X-ray Powder Diffraction (XRD) pattern measurements. Here Cu Kα radiation (λ = 1.5418 Å) employing a 0.25° divergent slit and a 0.125° anti-scattering slit was utilized. The patterns were recorded in the 2θ range from 10° to 80° with a step of 0.017365 ° and a counting time of 10 s per step.

Liquid-chromatography (LC) mass-spectrometry (MS) (LC-MS) measurements were performed on a Agilent InfinityLab LC/MSD (G6125C SG2215N102) with an Agilent 1290 Infinity II. A non-polar column by Waters (BEH-C4, 2.1x150, 1.7 micron) was utilized with 100.0% Water as eluent at a flow rate of 0.300 mL min^{-1} and 600.00 bar pressure (26 min acquisition time). The injection volume was set to 1.00 µL. The UV-DAD detector followed products at wavelengths of 200 nm, 210 nm and 250 nm and full spectra were recorded from 190 nm to 350 nm. The SQ Mass Spectrometer was set to follow mass values ranging from 40-600 m/z. Simultaneously SIM scans at masses of 312 m/z, 313 m/z and 281 m/z were conducted.

Gas-chromatography (GC) mass-spectrometry (MS) (GC-MS) measurements were performed on a Shimadzu GC-2010 (Japan) gas chromatograph with a GCMS-QP2010 mass-spectrometer. A non-polar column ((5%-phenyl)-methylpolysiloxane) by Agilent (dimensions 30 m · 0.25 mm · 0.25 µm) was utilized.

Headspace GC-TCD (thermal conductivity detector) measurements taken *via* measurement from the headspace of the 10-mL pressure vial using a VICI Precision Sampling PRESSURE-LOK® syringe equipped with a push-button valve. The evolved gases in the headspace of the reaction were analyzed with a Shimadzu Nexis GC-2030 (Japan) gas chromatograph (GC), equipped with a thermal conductivity detector (TCD). More sampling details and equipment specifics are described in **Section 8.1**. 5-mL samples of the headspace were injected in the 50 µL sample loop of a SHIMADZU Nexis GC-2030 (Japan) gas chromatograph, equipped with two columns connected to a 6-way valve.

X-ray photoelectron spectroscopy (XPS) was performed using a Surface Science Instruments SSX-100 ESCA spectrometer, equipped with a monochromatic Al Kα X-ray source (hv =1486.6 eV). The pressure in the measurement chamber was maintained below $5*x10^{-9}$ mbar during data acquisition. The photoelectron take-off angle was 37° with respect to the surface normal. The diameter of the analyzed area was 1000 µm; the energy resolution was 1.26 eV (or 1.67 eV for a broad survey scan). A more detailed description can be found in **Section 8.3**.

Computational methods: All computational input files were prepared in GaussView 6.0 on a local Windows 10 terminal. Input files were then transferred to the University of Groningen Peregrine HPC cluster where DFT or TD-DFT calculations were carried out using the Gaussian 16 (g16) suite of programs. A more detailed description can be found in **Section 8.4 & 8.5**.

3. Experimental procedures

3.1 Catalyst synthesis in dibutyl ether and optimization

Iron(0) pentacarbonyl (0.4 g, 3.04 mmol, 1 eq.), oleic acid (1.1 g, 3.5 mmol, 1.15 eq, 90%) and dibutyl ether (12 mL) were heated to 150°C for an hour. After cooling to room temperature with an ice bath, trimethylamine N-oxide dihydrate (1 g (98%), 8.82 mmol, 2.90 eq.) was added while flushing the system with nitrogen. The temperature was subsequently increased to 130°C for two hours and after to 150°C for one hour. After cooling to room temperature with an ice bath the particles were magnetically precipitated in ethanol on a magnet. After washing once with ethanol, the particles were dried and dissolved in DCM for storage in the fridge.

The addition of trimethylamine N-oxide was necessary to enhance the substitution of carbon monoxide ligands and their disconnection to the iron(0) core. Thereby oleic acid is able to coordinate/connect to iron.

$$\mathbf{M(CO)_n + Me_3NO + L \rightarrow M(CO)_{n-1}L + Me_3N + CO_2}$$

Scheme S1: Production of carbon dioxide by addition of trimethylamine N-oxide to iron(0) pentacarbonyl.

A screening was subsequently performed on the ratio of oleic acid: iron(0) pentacarbonyl (usually 2:1). It was found that only 2:1 molar ratios gave best results, whereas 1:1 and 3:1 ratios were proven inactive by peroxide test strip and titration. A molar ratio of 4:1 was also attempted; however, these particles were not magnetically precipitating on the magnet in the workup. Nanoparticles shrink in size with increasing amounts of surfactant added, which could explain the particles to not magnetically fall down as they were too well dispersed.

3.2 Catalyst synthesis optimization

Since the above-mentioned nanoparticles were found to perform well, we opted for further optimization of the synthesis of these iron oxide nanoparticles with molar oleic acid ratio of 2:1. It was found that glassware used in the synthesis and workup had influence on the performance of the photocatalyst for hydrogen peroxide production. Heterogeneous catalysts are sensitive to changes in mass and heat transport. Thus, the performance of heterogeneous catalysts can strongly be impacted by changes in parameters like temperature, particle size, pore dimensions and reactor configuration.[2] After thoroughly screening glassware in the lab (50 mL, 100 mL, 250 mL, 500 mL), it was found that a 100 mL two-neck flask yielded the most active nanoparticles in the synthesis. A 100 mL three-neck flask, a 50 mL two-neck flask and 250 mL 2-neck flask were also tested, where the particles produced in the 50 mL two-neck flask were inactive. Reasons for dependence on the glassware could be the different rates of heating at reflux provided by each piece of glassware or differences in the sheer rate from the magnet (egg shaped, 2 cm). An attempt with overhead stirring during the synthesis was made, but not continued further as it led to inactive particles. Upon precipitation on a magnet, it was found that also here the choice of glassware had an effect on the nanoparticles. 250 mL, 500 mL and 1000 mL beakers were tested, where 500 mL beakers produced the most active nanoparticles. Each beaker size has a different diameter, which influences the way the magnet pulls the magnetic nanoparticles out of solution.

The effect of temperature and atmosphere on the synthesis were also tested. Dibutyl ether has a boiling point of 141 °C, therefore the synthesis was conducted at a temperature of 150 °C to obtain reflux. The synthesis was conducted at 100 °C once, which gave less active nanoparticles. Similarly, synthesis in air instead of nitrogen atmosphere resulted in less active nanoparticles. These findings emphasize the importance of reflux conditions and nitrogen atmosphere in the synthesis for active iron oxide nanoparticles.

Initially, the nanoparticles were stored in the refrigerator in an air atmosphere, which contained enough oxygen to initiate self-autoxidation. This resulted in blank positive results in darkness, if the nanoparticles had been stored for some time already. This could be prevented by storing the nanoparticles in DCM in nitrogen atmosphere in the fridge. Ten active batches of nanoparticles were combined with the intention to make a large batch for condition screening; however, this deactivated the particles. Therefore, it was opted to always store each separate batch of nanoparticles under a nitrogen atmosphere in the fridge in a 20 mL vial. Additionally, parafilm was wrapped around the cap to ensure that each vial remained sealed and its nitrogen atmosphere maintained.

Finally, ethanol and methanol were tested as solvent to replace dibutyl ether in the synthesis, since these are more sustainable following green chemistry principles. Both of these solvents in the synthesis were found to give positive results, where synthesis in ethanol was found to be more active. Synthesis in ethanol as a solvent resulted in higher yields and more consistent results from batch to batch compared to dibutyl ether as solvent in synthesis. Hence, we opted for ethanol as the standard solvent.

3.3 Catalyst synthesis in ethanol

Ethanol as solvent in the synthesis instead of dibutyl ether yielded more active and reproduceable nanoparticles. Synthesis in ethanol is more sustainable than dibutyl ether since ethanol can be obtained from biomass.[3] Also, oleic acid can be regarded sustainable as it is the main component in olive oil.[4] Photocatalytic hydrogen peroxide production *via* these iron oxide nanoparticles compared to the already existing anthraquinone process is more sustainable according to the principles of green chemistry.[5,6] The described photocatalytic process is safer, creates less waste, uses more renewable materials and can be performed at ambient temperature and pressure. Building upon the synthesis in ethanol, we prepared nanoparticles with a number of different surfactants in order to gain a better understanding of the hydrogen peroxide production mechanism. Conditions were kept as optimized in the previous section, where reflux in ethanol is conducted at 150 °C (boiling point: 78 °C). This temperature is significantly above the boiling point of ethanol but was found to be crucial as synthesis at lower temperatures yielded inactive nanoparticles. The rate of reflux is thus important for activity of the NPs. It should be highlighted, that synthesis in ethanol effectively resembles a synthesis directly in the anti-solvent, which usually would be used to enhance precipitation of nanoparticles in the workup step. Utilizing the described technique, a scope of surfactants was synthesized (**Figure S3**).

Immobilization on activated charcoal (mesh 20-40 and 100) as well as graphene nanoplatelets was achieved and resulted in magnetic activated charcoal and graphene nanoplatelet particles as shown in **Figure S30, Figure S32** and **Figure S34**. Nanoparticles using stearic acid and linolenic acid could not be synthesized (**Figure S3)**. Stearic acid is a solid at room temperature and did not dissolve in ethanol at room temperature. At elevated temperatures the surfactant was miscible, however, when cooled all surfactant precipitated as shown in **Figure S20**. Stearic acid does not dissolve in oleic acid, so synthesis with a 1:1 mixture of these surfactants was also not an option. NPs with linolenic acid did not magnetically precipitate on the magnet, possibly because particles were too small and too well dispersed for precipitation. After synthesis the nanoparticles were stored in DCM under nitrogen atmosphere in darkness in the fridge. Successful surfactant incorporation was tested by their stability in DCM overnight; a batch was regarded stable if still in solution overnight.[7]

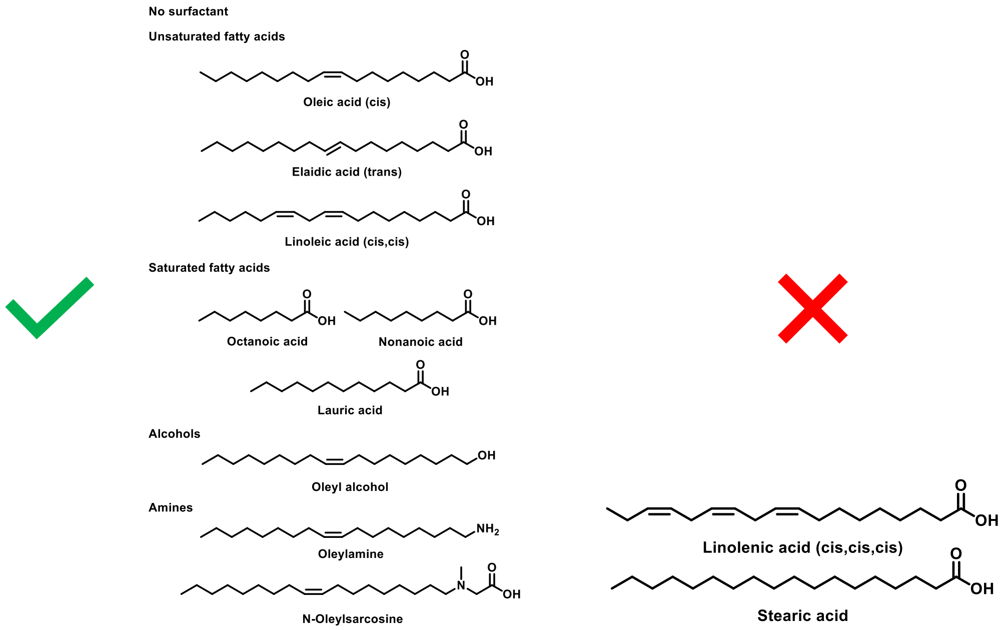

Figure S3: Scope of surfactants employed for successful iron oxide nanoparticle synthesis (left); surfactants not successful in nanoparticle synthesis (right).

3.3.1 Synthesis of iron oxide nanoparticles without surfactant

Ethanol (12 mL) and iron pentacarbonyl (0.4 mL, 3.04 mmol, 1 eq.) were added to a 100 mL two-neck round bottom flask and the mixture heated at reflux (150°C Allihn condenser) for 1h at a stirring speed of 660 rpm (**Figure S7**). After 1h solid precipitated out of solution (**Figure S4 (A)**). The mixture was initially cooled using an ice bath and then with a 20°C water bath to *room temperature* (20°C) **(B)**, while filling the decreasing volume of the headspace with nitrogen. To the cooled mixture trimethyl amine N-oxide dihydrate (1 g (98%), 8.82 mmol, 2.90 eq.) was added while flushing the system with nitrogen, which resulted in bubbling and a dark solution **(C)**. The mixture was then heated to 130°C for 2h under nitrogen atmosphere **(D)**, where overpressure of gas (CO) was allowed to leave the system in the first few minutes. After 2h the temperature was increased to 150°C for 1h. The reaction was stopped by cooling with an ice bath and subsequently with a water bath until but *not further* than 20°C (whole cooling process: 1 min), while flushing with nitrogen. The nanoparticles were decanted into a beaker (500 mL) and precipitated from ethanol (200 mL). The magnetic nanoparticles were pulled out of solution (212 mL) by a magnet under the beaker for 1h (**Figure S5**). The ethanol was decanted off and the particles were washed once with ethanol (50 mL). Dried *via* constant air flow, the particles were dissolved in dichloromethane (10 mL) and stored in nitrogen atmosphere and darkness at 5°C. The particles were not stable in DCM overnight, as shown in **Figure S6**. The approximate yield was 65.5 mg per batch.

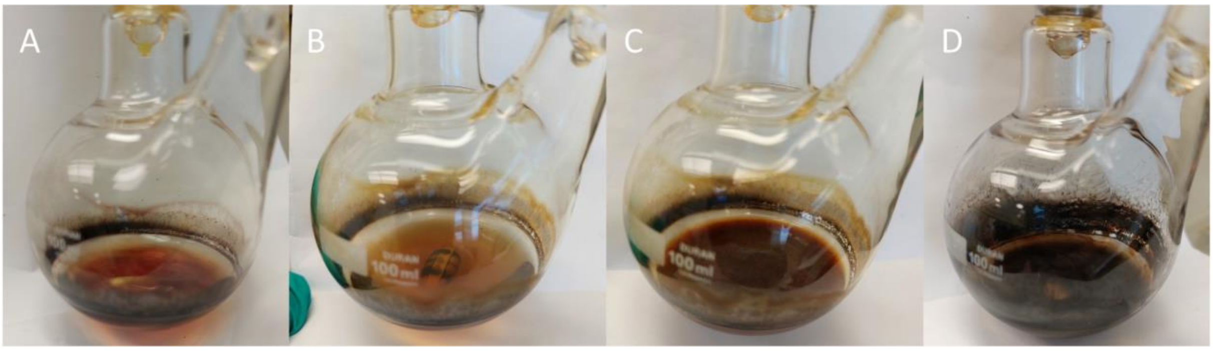

Figure S4: FeO_x NPs (batch 143) without surfactant. A) t=50 min, B) t=60 min cooled, C) t=60 min Me_3N added, D) t=100 min.

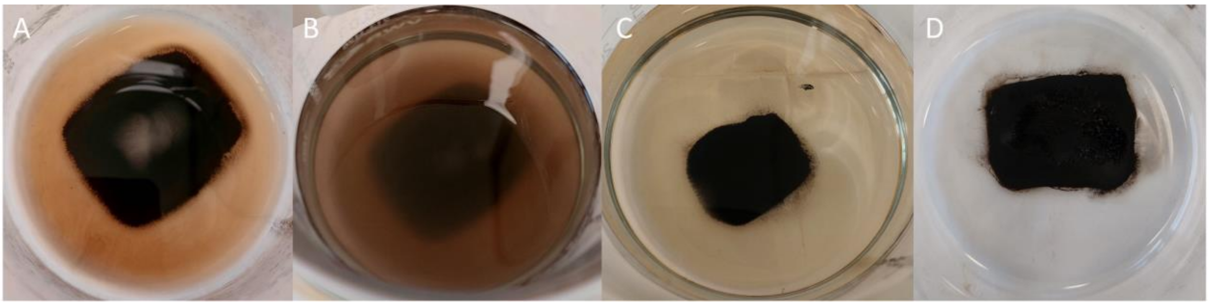

Figure S5: Precipitation on magnet of FeO_x NPs (batch 143) without surfactant.

Figure S6: Photograph of FeO_x NPs (batch 143) without surfactant in DCM after 12h - it is clear that the dispersion is not stable in solution.

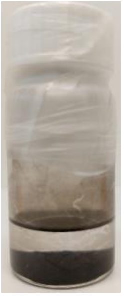

3.3.2 Synthesis of iron oxide nanoparticles with oleic acid

Oleic acid (2.20 g (90%), 7.01 mmol, 2.31 eq.), ethanol (12 mL) and iron pentacarbonyl (0.4 mL, 3.04 mmol, 1 eq.) were added to a 100 mL two-neck round bottom flask and the mixture heated at reflux (150°C Allihn condenser) for 1h at a stirring speed of 660 rpm (**Figure S7**). After 1h the mixture had turned from yellow to orange/brown (**Figure S8 (A)**). The mixture was initially cooled using an ice bath and then with a 20°C water bath to *room temperature* (20°C) **(B)**, while filling the decreasing volume of the headspace with nitrogen. To the cooled mixture trimethyl amine N-oxide dihydrate (1 g (98%), 8.82 mmol, 2.90 eq.) was added while flushing the system with nitrogen, which resulted in bubbling and a dark solution **(C)**. The mixture was then heated to 130°C for 2h under nitrogen atmosphere resulting in a colour change to yellow **(E)**, where overpressure of gas (CO) was allowed to leave the system in the first few minutes **(D)**. After 2h the temperature was increased to 150°C and kept for 1h. The reaction was stopped by cooling with an ice bath and subsequently with a water bath until but *not further* than 20°C (whole cooling process: 1 min, **F**), while flushing the solution with nitrogen. The nanoparticles were decanted into a beaker (500 mL, **G**) and precipitated from ethanol (200 mL). While rinsing the round bottom flask with the ethanol the magnetic nanoparticle droplets were already formed inside the flask **(H)**. The magnetic nanoparticles were pulled out of solution (212 mL) by a magnet under the beaker for 1h (**Figure S9 I, J**). The ethanol was decanted off and the particles were washed once with ethanol (50 mL) **(K, L)**. Dried *via* constant air flow, the particles were dissolved in dichloromethane (10 mL) and stored in nitrogen atmosphere and darkness at 5°C. The particles were stable in DCM overnight, as shown in **Figure S9**. The approximate yield was 7-34% corresponding to 100-500 mg per batch.

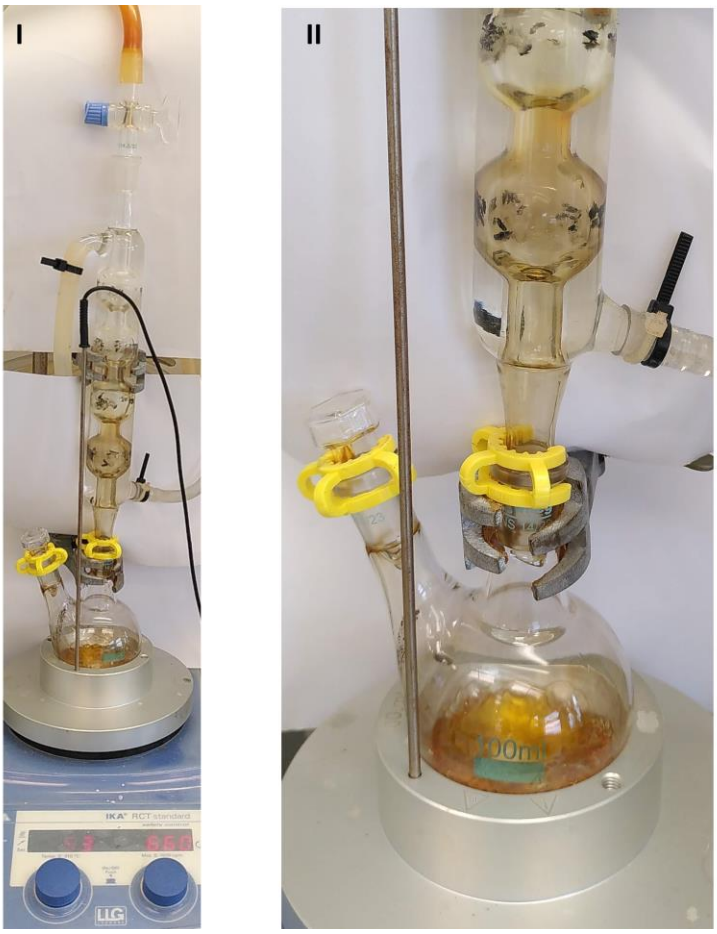

Figure S7: Synthesis equipment used for the FeO_x nanoparticle synthesis. Two-neck round bottom flask equipped with an Allihn condenser and heated *via* metal heating mantle.

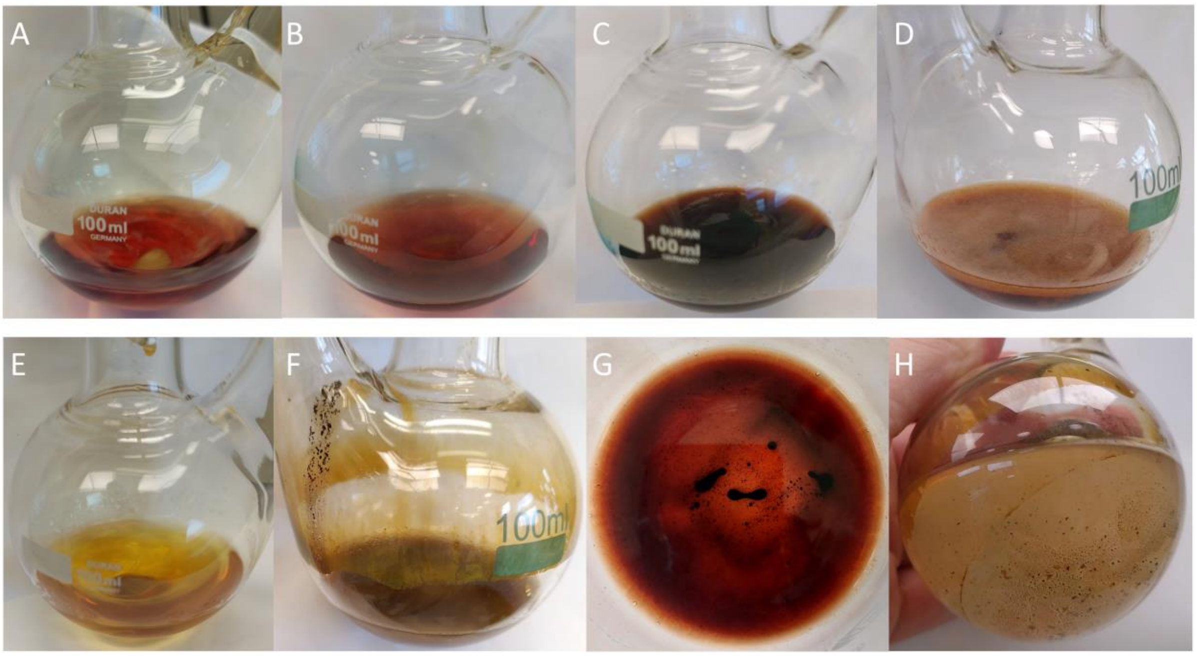

Figure S8: Photographs of FeO_x NPs (batch 141) with oleic acid surfactant 2:1. A) t=50 min, B) t=60 min cooled, C) cooled at t=60 min Me_3N added, D) heating up at t=63 min and gas formation E) t=100 min, F) cooled solution after synthesis, G) precipitation on magnet, purely decanted, H) washing and rinsing of round bottom flask for droplet formation.

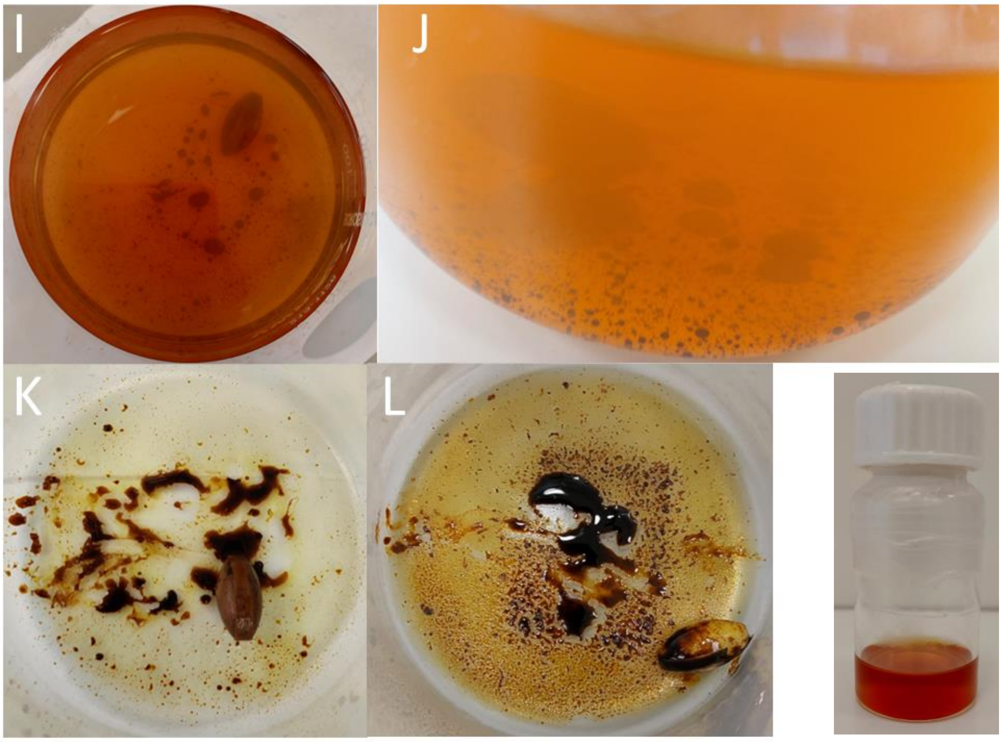

Figure S9: Precipitation on top of a magnet of FeO_x NPs (batch 141) with oleic acid 2:1 as surfactant. I,J) 1h washing with 200 mL EtOH and droplet formation, K,L) washed FeO_x NPs with ethanol (50 mL). Photograph of FeO_x NPs (batch 153) with oleic acid 2:1 in DCM after 12h - it is clear that the dispersion is stable and no precipitates can be identified (bottom right).

3.3.3 Synthesis of iron oxide nanoparticles with oleic acid: troubleshooting

The synthesis was found to be consistent (>210 batches) and could successfully be reproduced by three different researchers in different labs at different locations (a bachelor student at Linnaeusborg (University of Groningen), a master student at Linnaeusborg (University of Groningen), a PhD candidate at Nijenborgh & Linnaeusborg (University of Groningen) and at University of Amsterdam). Especially for the synthesis of heterogeneous catalyst materials, reproducibility is crucial and often an overlooked aspect.[2] Successful synthesis was independent of iron(0) pentacarbonyl suppliers (Sigma Aldrich, Acros Organics) with different Lot-numbers, coming from different continents.

Unsuccessful syntheses of a few batches led to an extensive troubleshooting. The importance of dry conditions was discovered when once 96% ethanol was used instead of the usual 100% ethanol: big pieces were floating around in the flask after addition of trimethylamine N-oxide dihydrate and heating to reflux, where usually with 100% ethanol a completely dissolved and homogeneous solution is obtained. A similar phenomenon was observed when water was once utilized as 1:1 cosolvent with ethanol, suggesting that water in the synthesis leads to undesired precipitation of nanoparticles out of solution. Wet nitrogen gas from the Schlenk-line also led to unsuccessful nanoparticle synthesis; here the nanoparticles did not magnetically precipitate from the solution during workup. Over time stirring bars became yellow/brown after repeatedly being used for synthesis and cleaning using hydrochloric acid solution (37%), which led to particles not magnetically precipitating during workup. By using new stirring bars, we were able to overcome this problem. Over time iron(0) pentacarbonyl was found to precipitate as a solid in the normally yellow liquid, which led to differently looking pieces magnetically precipitating in the workup. We suspect that precipitation was caused by the septum on the bottle being punctured too often, therefore not sufficiently sealing the nitrogen atmosphere in the bottle anymore. Iron(0) pentacarbonyl is a pyrophoric compound, meaning that it could react with air to burn to iron(III) oxide.[8] By switching to a new bottle of iron(0) pentacarbonyl these problems could be avoided. When the trimethylamine N-oxide dihydrate was too dry (dry powder instead of hygroscopic white solid) inconsistent syntheses were observed. A 'wet' hygroscopic solid is recommended over a dry white powder. During the cooling steps of the reaction mixture nitrogen has to be flushed into the decreasing volume of the headspace. Furthermore, the cooling should be performed rapidly with an ice bath for up to 1 min, but not longer and the temperature should not drop below 20°C; this can be monitored by replacing the initial ice cooling with an additional water bath. Letting the solution drop below 16°C leads to precipitation of frozen oleic acid. These crystals will trap the 2 nm FeO_x onto 400-600 nm crystals of oleic acid, which is not desired. Extensive cooling also leads to gel/droplet/oil formation at the bottom of the round bottom flask, resulting in tedious workup and transfer to the beaker.

Below a list of requirements is given as guide, which should be consulted if there are difficulties in synthesis.

List of requirements for successful nanoparticle synthesis

- 100% ethanol as solvent
- clean and dry glassware (100 mL 2-neck flask, Allihn condenser, adapter to Schlenk-line)
- clean stirring bar (egg shaped, 2 cm)
- clean Schlenk-line tubing
- dry nitrogen (make sure phosphorus pentoxide is still dry by moisture indicator)
- clean oil in Schlenk-line
- 660 rpm stirring speed
- sufficient reflux
- 500 mL beaker during workup on magnet
- cooling with the ice bath not below 20 °C to avoid precipitation of oleic acid (16 °C)
- hygroscopic trimethylamine N-oxide dihydrate

3.3.4 Synthesis of iron oxide nanoparticles with elaidic acid

Elaidic acid (2.20 g (97%), 7.56 mmol, 2.49 eq.), ethanol (12 mL) and iron pentacarbonyl (0.4 mL, 3.04 mmol, 1 eq.) were added to a 100 mL two-neck round bottom flask and the mixture heated at reflux (150°C Allihn condenser) for 1h at a stirring speed of 660 rpm (**Figure S7**). After 1h the mixture had turned from yellow to slightly orange (**Figure S10 (A)**). The mixture was initially cooled using an ice bath and then with a 20°C water bath to *room temperature* (20°C) **(B)**, while filling the decreasing volume of the headspace with nitrogen. To the cooled mixture trimethyl amine N-oxide dihydrate (1 g (98%), 8.82 mmol, 2.90 eq.) was added while flushing the system with nitrogen, which resulted in bubbling and a dark solution **(C)**. The mixture was then heated to 130°C for 2h under nitrogen atmosphere resulting in a colour change to yellow/orange **(D)**, where overpressure of gas (CO) was allowed to leave the system in the first few minutes. After 2h the temperature was increased to 150°C for 1h. The reaction was stopped by cooling with an ice bath and subsequently with a water bath until but *not further* than 20°C (whole cooling process: 1 min, **(E)**), while flushing with nitrogen. The nanoparticles were decanted into a beaker (500 mL) and precipitated from ethanol (200 mL). The magnetic nanoparticles were pulled out of solution (212 mL) by a magnet under the beaker for 1h (**Figure S11**). The ethanol was decanted off and the particles were washed once with ethanol (50 mL). Dried *via* constant air flow, the particles were dissolved in dichloromethane (10 mL) and stored in nitrogen atmosphere and darkness at 5°C. The nanoparticles were stable in DCM overnight, as shown in **Figure S11**. The approximate yield was 218 mg per batch.

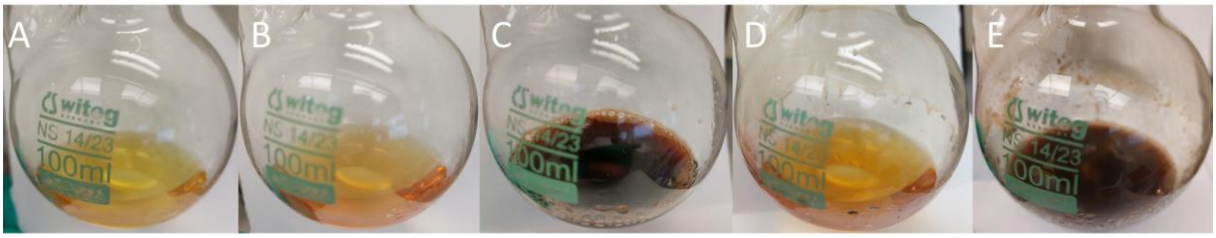

Figure S10: Photographs of FeO_x NPs (batch 172) with elaidic acid surfactant 2:1. A) t=50 min, B) t=60 min cooled, C) t=60 min Me_3N added, D) t=100 min, E) t=240 min cooled.

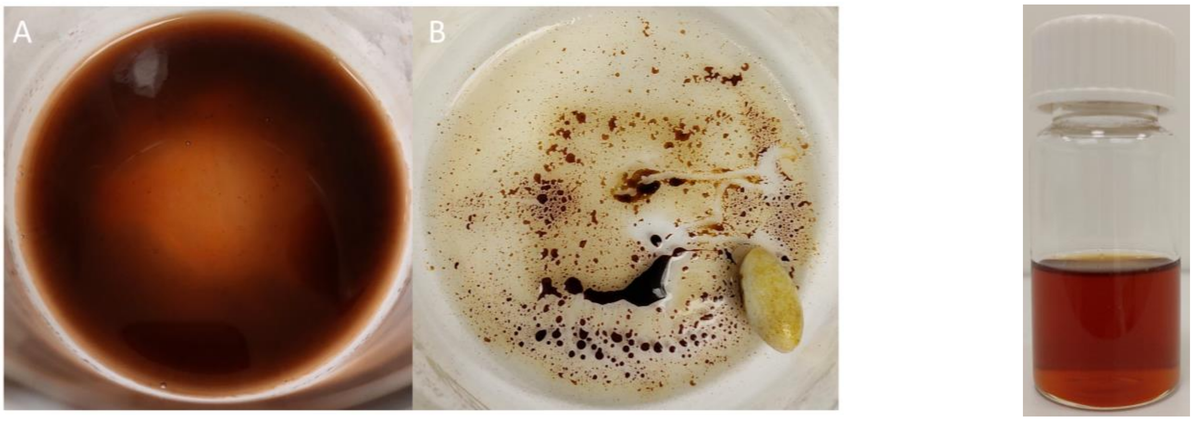

Figure S11: A,B) Precipitation on top of a magnet of FeO_x NPs (batch 172) with elaidic acid 2:1 as surfactant. Photograph of FeO_x NPs (batch 172) with elaidic acid 2:1 in DCM after 12h - it is clear that the dispersion is stable and no precipitates can be identified (bottom right).

3.3.5 Synthesis of iron oxide nanoparticles with linoleic acid

Linoleic acid (2.10 g (95%), 7.11 mmol, 2.34 eq.), ethanol (12 mL) and iron pentacarbonyl (0.4 mL, 3.04 mmol, 1 eq.) were added to a 100 mL two-neck round bottom flask and the mixture heated at reflux (150°C Allihn condenser) for 1h at a stirring speed of 660 rpm (**Figure S7**). After 1h the mixture colour of the mixture was yellow (**Figure S12 (A)**). The mixture was initially cooled using an ice bath and then with a 20°C water bath to *room temperature* (20°C) **(B)**, while filling the decreasing volume of the headspace with nitrogen. To the cooled mixture trimethyl amine N-oxide dihydrate (1 g (98%), 8.82 mmol, 2.90 eq.) was added while flushing the system with nitrogen, which resulted in bubbling and a dark solution **(C)**. The mixture was then heated to 130°C for 2h under nitrogen atmosphere resulting in a colour change to yellow/orange **(D)**, where overpressure of gas (CO) was allowed to leave the system in the first few minutes. After 2h the temperature was increased to 150°C and kept for 1h. The reaction was stopped by cooling with an ice bath and subsequently with a water bath until but *not further* than 20°C (whole cooling process: 1 min), while flushing with nitrogen. The nanoparticles were decanted into a beaker (500 mL) and precipitated from ethanol (200 mL). The magnetic nanoparticles were pulled out of solution (212 mL) by a magnet under the beaker for 1h (**Figure S13**). The ethanol was decanted off and the particles were washed once with ethanol (50 mL). Dried *via* constant air flow, the particles were dissolved in dichloromethane (10 mL) and stored in nitrogen atmosphere and darkness at 5°C. The nanoparticles were stable in DCM overnight, as shown in **Figure S13**. The approximate yield was 50 mg per batch.

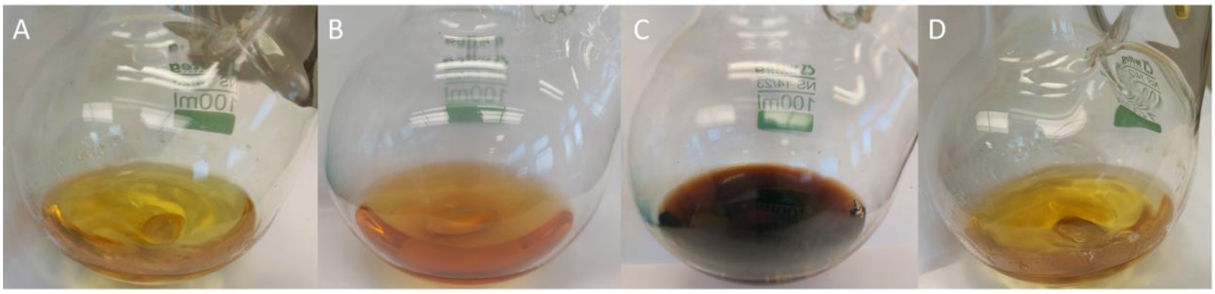

Figure S12: Photographs of FeO_x NPs (batch 144) with linoleic acid surfactant 2:1. A) t=50 min, B) t=60 min cooled, C) t=60 min Me_3N added, D) t=100 min.

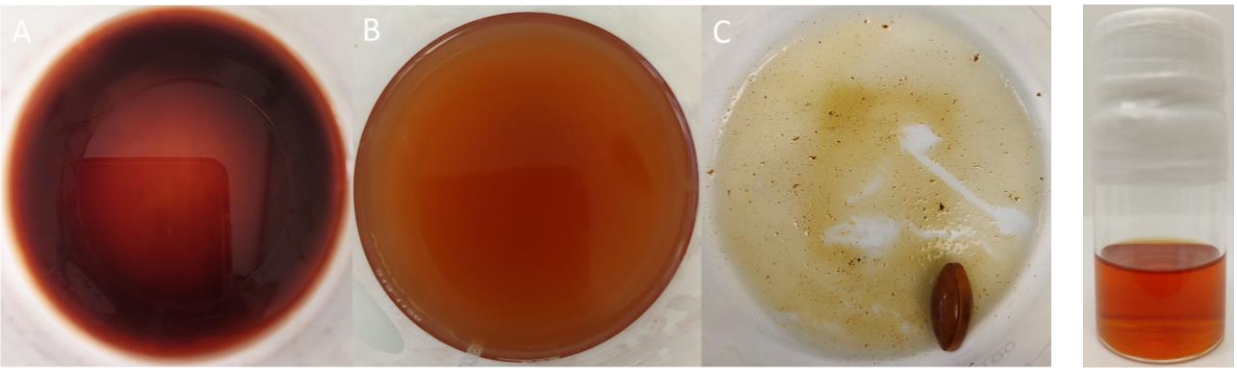

Figure S13: A,B,C) Precipitation on top of a magnet of FeO_x NPs (batch 144) with linoleic acid 2:1 as surfactant. Photograph of FeO_x NPs (batch 144) with linoleic acid 2:1 in DCM after 12h - it is clear that the dispersion is stable and no precipitates can be identified (bottom right).

3.3.6 Synthesis of iron oxide nanoparticles with octanoic acid

Octanoic acid (0.52 g (99%), 3.57 mmol, 1.17 eq.), ethanol (12 mL) and iron pentacarbonyl (0.4 mL, 3.04 mmol, 1 eq.) were added to a 100 mL two-neck round bottom flask and the mixture heated at reflux (150°C Allihn condenser) for 1h at a stirring speed of 660 rpm (**Figure S7**). After 1h the mixture had turned from yellow to brown (**Figure S14 (A)**). The mixture was initially cooled using an ice bath and then with a 20°C water bath to *room temperature* (20°C) **(B)**, while filling the decreasing volume of the headspace with nitrogen. To the cooled mixture trimethyl amine N-oxide dihydrate (1 g (98%), 8.82 mmol, 2.90 eq.) was added while flushing the system with nitrogen, which resulted in bubbling and a dark solution **(C)**. The mixture was then heated to 130°C for 2h under nitrogen atmosphere resulting in formation of a precipitate on the side of the flask **(D)**, where overpressure of gas (CO) was allowed to leave the system in the first few minutes. After 2h the temperature was increased to 150°C for 1h. The reaction was stopped by cooling with an ice bath and subsequently with a water bath until but *not further* than 20°C (whole cooling process: 1 min **(E)**), while flushing with nitrogen. The nanoparticles were decanted into a beaker (500 mL) and precipitated from ethanol (200 mL). The magnetic nanoparticles were pulled out of solution (212 mL) by a magnet under the beaker for 1h (**Figure S15**). The ethanol was decanted off and the particles were washed once with ethanol (50 mL). Dried *via* constant air flow, the particles were dissolved in dichloromethane (10 mL) and stored in nitrogen atmosphere and darkness at 5°C. The nanoparticles were stable in DCM overnight, as shown in **Figure S15**. The approximate yield was 105 mg per batch.

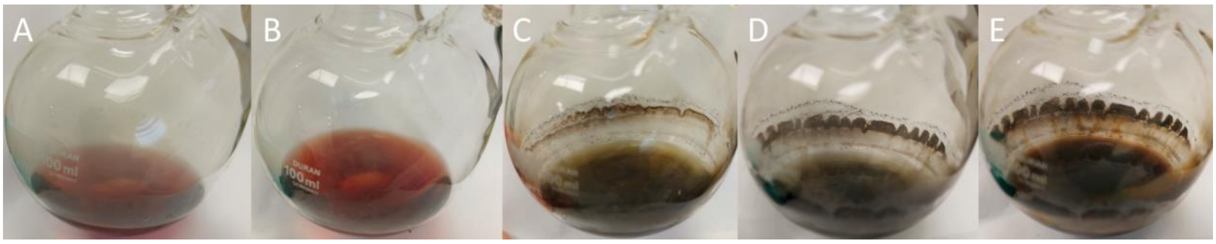

Figure S14: Photographs of FeO_x NPs (batch 161) with octanoic acid surfactant 1:1. A) t=50 min, B) t=60 min cooled, C) t=60 min Me_3N added, D) t=100 min, E) t=240 min cooled.

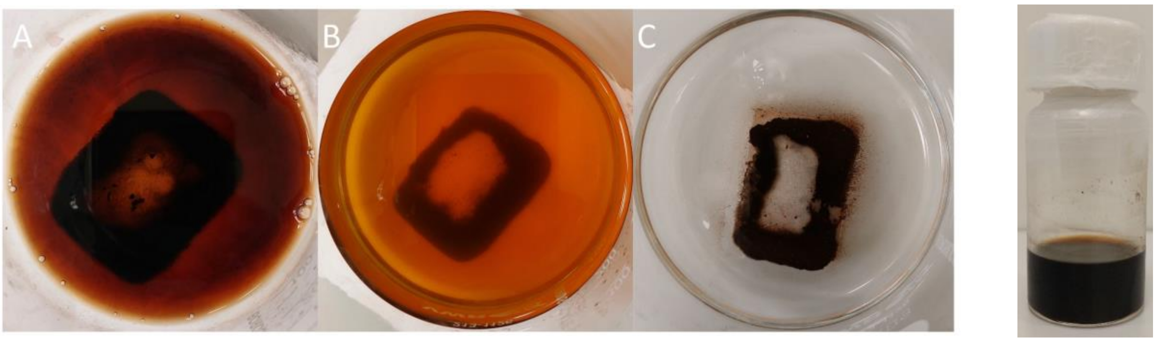

Figure S15: A,B,C) Precipitation on top of a magnet of iron oxide nanoparticles (batch 161) with octanoic acid 1:1 as surfactant. Photograph of FeO_x NPs (batch 161) with octanoic acid 1:1 in DCM after 12h - it is clear that the dispersion is stable and no precipitates can be identified (bottom right).

3.3.7 Synthesis of iron oxide nanoparticles with nonanoic acid

Nonanoic acid (0.56 g (97%), 3.43 mmol, 1.13 eq.), ethanol (12 mL) and iron pentacarbonyl (0.4 mL, 3.04 mmol, 1 eq.) were added to a 100 mL two-neck round bottom flask and the mixture heated at reflux (150°C Allihn condenser) for 1h at a stirring speed of 660 rpm (**Figure S7**). After 1h the mixture had turned from yellow to brown (**Figure S16 (A)**). The mixture was initially cooled using an ice bath and then with a 20°C water bath to *room temperature* (20°C) **(B)**, while filling the decreasing volume of the headspace with nitrogen. To the cooled mixture trimethyl amine N-oxide dihydrate (1 g (98%), 8.82 mmol, 2.90 eq.) was added while flushing the system with nitrogen, which resulted in bubbling and a dark solution **(C)**. The mixture was then heated to 130°C for 2h under nitrogen atmosphere, resulting in formation of a precipitate on the side of the flask **(D)**, where overpressure of gas (CO) was allowed to leave the system in the first few minutes. After 2h the temperature was increased to 150°C for 1h. The reaction was stopped by cooling with an ice bath and subsequently with a water bath until but *not further* than 20°C (whole cooling process: 1 min **(E)**), while flushing with nitrogen. The nanoparticles were decanted into a beaker (500 mL) and precipitated from ethanol (200 mL). The magnetic nanoparticles were pulled out of solution (212 mL) by a magnet under the beaker for 1h (**Figure S17**). The ethanol was decanted off and the particles were washed once with ethanol (50 mL). Dried *via* constant air flow, the particles were dissolved in dichloromethane (10 mL) and stored in nitrogen atmosphere and darkness at 5°C. The particles were stable in DCM overnight, as shown in **Figure S17**. The approximate yield was 125 mg per batch.

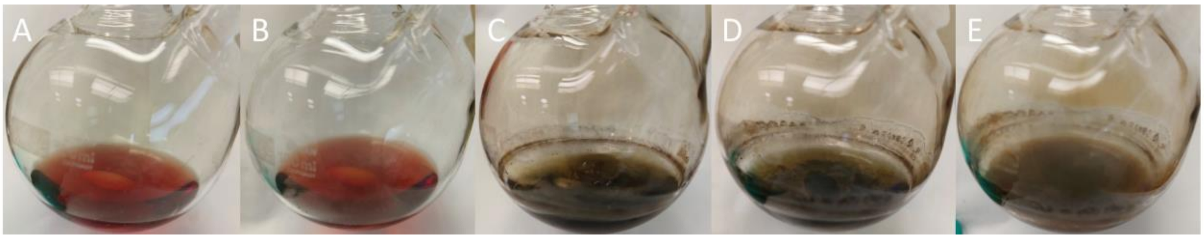

Figure S16: Photographs of FeO_x NPs (batch 162) with nonanoic acid surfactant 1:1. A) t=50 min, B) t=60 min cooled, C) t=60 min Me_3N added, D) t=100 min, E) t=240 min cooled.

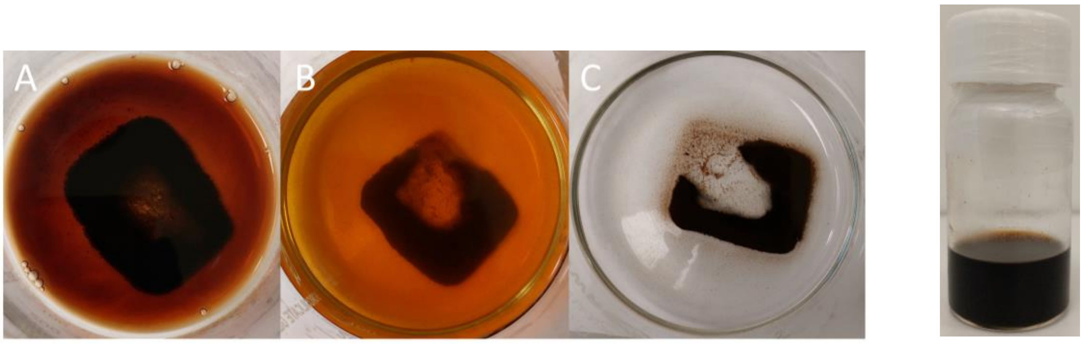

Figure S17: A,B,C) Precipitation on top of a magnet of FeO_x NPs (batch 162) with nonanoic acid 1:1 as surfactant. Photograph of FeO_x NPs (batch 162) with nonanoic acid 1:1 in DCM after 12h - it is clear that the dispersion is stable and no precipitates can be identified (bottom right).

3.3.8 Synthesis of iron oxide nanoparticles with lauric acid

Lauric acid (1.05 g (98%), 5.14 mmol, 1.69 eq.), ethanol (12 mL) and iron pentacarbonyl (0.4 mL, 3.04 mmol, 1 eq.) were added to a 100 mL two-neck round bottom flask and the mixture heated at reflux (150°C Allihn condenser) for 1h at a stirring speed of 660 rpm (**Figure S7**). After 1h the mixture had turned from yellow to red/brown (**Figure S18 (A)**). The mixture was initially cooled using an ice bath and then with a 20°C water bath to *room temperature* (20°C) **(B)**, while filling the decreasing volume of the headspace with nitrogen. To the cooled mixture trimethyl amine N-oxide dihydrate (1 g (98%), 8.82 mmol, 2.90 eq.) was added while flushing the system with nitrogen, which resulted in bubbling and a dark solution **(C)**. The mixture was then heated to 130°C for 2h under nitrogen atmosphere resulting in formation of a precipitate on the Teflon stirring bar **(D)**, where overpressure of gas (CO) was allowed to leave the system in the first few minutes. After 2h the temperature was increased to 150°C for 1h. The reaction was stopped by cooling with an ice bath and subsequently with a water bath until but *not further* than 20°C (whole cooling process: 1 min **(E)**), while flushing with nitrogen. The nanoparticles were decanted into a beaker (500 mL) and precipitated from ethanol (200 mL). The magnetic nanoparticles were pulled out of solution (212 mL) by a magnet under the beaker for 1h (**Figure S19**). The ethanol was decanted off and the particles were washed once with ethanol (50 mL). Dried *via* constant air flow, the particles were dissolved in dichloromethane (10 mL) and stored in nitrogen atmosphere and darkness at 5°C. The nanoparticles were stable in DCM overnight, as shown in **Figure S19**. The approximate yield was 242 mg per batch.

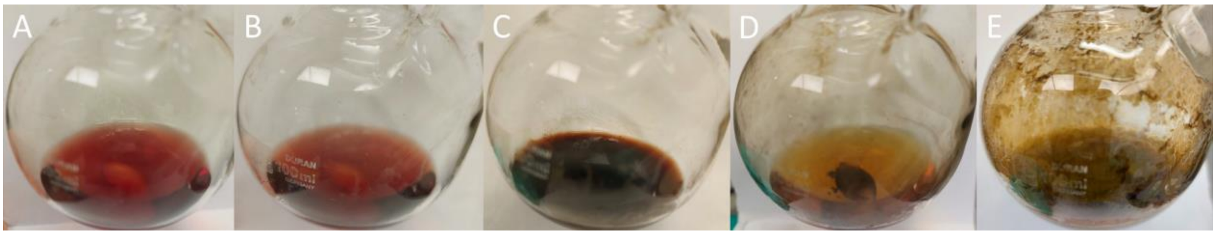

Figure S18: Photographs of FeO_x NPs (batch 171) with lauric acid surfactant 1.5:1. A) t=50 min, B) t=60 min cooled, C) t=60 min Me_3N added, D) t=100 min, E) t=240 min cooled.

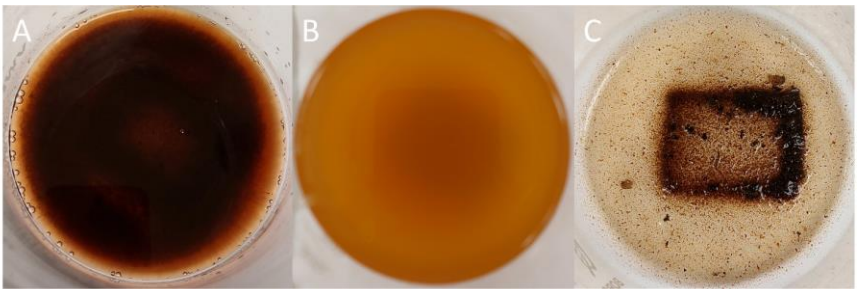

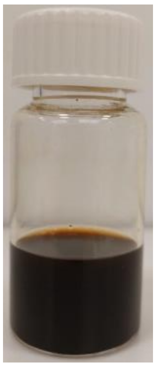

Figure S19: A,B,C) Precipitation on top of a magnet of FeO_x NPs (batch 171) with lauric acid 1.5:1 as surfactant. Photograph of FeO_x NPs (batch 171) with lauric acid 1.5:1 in DCM after 12h - it is clear that the dispersion is stable and no precipitates can be identified (bottom right).

3.3.9 Synthesis of iron oxide nanoparticles with stearic acid

Stearic acid (2.11 g (95%), 7.05 mmol, 3.34 eq.), ethanol (12 mL) and iron pentacarbonyl (0.4 mL, 3.04 mmol, 1 eq.) were added to a 100 mL two-neck round bottom flask and the mixture heated at reflux (150°C Allihn condenser) for 1h at a stirring speed of 660 rpm (**Figure S7**). After 1h the mixture had turned from yellow to red/brown (**Figure S20 (A)**). The mixture was initially cooled using an ice bath and then with a 20°C water bath to *room temperature* (20°C), while filling the decreasing volume of the headspace with nitrogen. This resulted in a white colour and solidification of the mixture **(B)**. To the cooled mixture trimethyl amine N-oxide dihydrate (1 g (98%), 8.82 mmol, 2.90 eq.) was added while flushing the system with nitrogen, which resulted in bubbling and a darker solution upon adding heat **(C)**. For the first few minutes of heating overpressure of gas (CO) was allowed to leave the system. The mixture was then heated to 130°C for 2h under nitrogen atmosphere resulting in a white mixture **(D)**. After 2h the temperature was increased to 150°C for 1h. The reaction was stopped by cooling with an ice bath and subsequently with a water bath until but *not further* than 20°C (whole cooling process: 1 min), while flushing with nitrogen. The nanoparticles were decanted into a beaker (500 mL) and precipitated from ethanol (200 mL). The magnetic nanoparticles were pulled out of solution (212 mL) by a magnet under the beaker for 1h (**Figure S21**). The ethanol was decanted off and the particles were washed once with ethanol (50 mL). Dried *via* constant air flow, the particles were dissolved in dichloromethane (10 mL) and stored in nitrogen atmosphere and darkness at 5°C. The nanoparticles were not stable in DCM overnight, as shown in **Figure S21**, where pieces floating around in the vial can be identified. This synthesis was regarded unsuccessful due to stearic acid being a solid during stages in the synthesis.

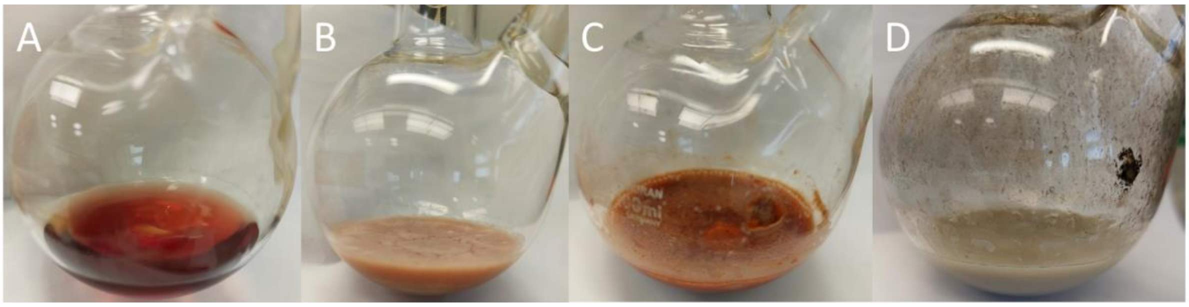

Figure S20: Photographs of FeO_x NPs (batch 146) with stearic acid 2:1. A) t=50 min, B) t=60 min cooled, C) t=60 min Me_3N added, D) t=100 min.

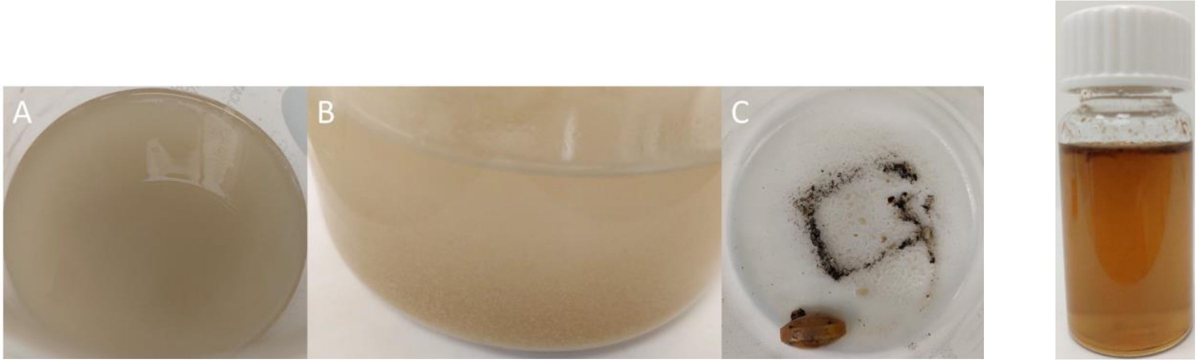

Figure S21: Precipitation on top of a magnet of FeO_x NPs (batch 146) with stearic acid 2:1 as surfactant. Photograph of FeO_x NPs (batch 146) with stearic acid 2:1 in DCM after 12h - it is clear that the dispersion is not stable and precipitates can be identified (bottom right).

3.3.10 Synthesis of iron oxide nanoparticles with oleyl alcohol

Oleyl alcohol (2.21 g (85%), 7.00 mmol, 2.30 eq.), ethanol (12 mL) and iron pentacarbonyl (0.4 mL, 3.04 mmol, 1 eq.) were added to a 100 mL two-neck round bottom flask and the mixture heated at reflux (150°C Allihn condenser) for 1h at a stirring speed of 660 rpm (**Figure S7**). After 1h a solid precipitated out of solution (**Figure S22 (A)**). The mixture was initially cooled using an ice bath and then with a 20°C water bath to *room temperature* (20°C) **(B)**, while filling the decreasing volume of the headspace with nitrogen. To the cooled mixture trimethyl amine N-oxide dihydrate (1 g (98%), 8.82 mmol, 2.90 eq.) was added while flushing the system with nitrogen, which resulted in bubbling and a dark solution **(C)**. The mixture was then heated to 130°C for 2h under nitrogen atmosphere resulting in precipitate on the side of the glass **(D)**, where overpressure of gas (CO) was allowed to leave the system in the first few minutes. After 2h the temperature was increased to 150°C for 1h. The reaction was stopped by cooling with an ice bath and subsequently with a water bath until but *not further* than 20°C (whole cooling process: 1 min), while flushing with nitrogen. The nanoparticles were decanted into a beaker (500 mL) and precipitated from ethanol (200 mL). The magnetic nanoparticles were pulled out of solution (212 mL) by a magnet under the beaker for 1h (**Figure S23**). The ethanol was decanted off and the particles were washed once with ethanol (50 mL). Dried *via* constant air flow, the particles were dissolved in dichloromethane (10 mL) and stored in nitrogen atmosphere and darkness at 5°C. The nanoparticles were not stable in DCM overnight, as shown in **Figure S24**. The approximate yield was 62 mg per batch.

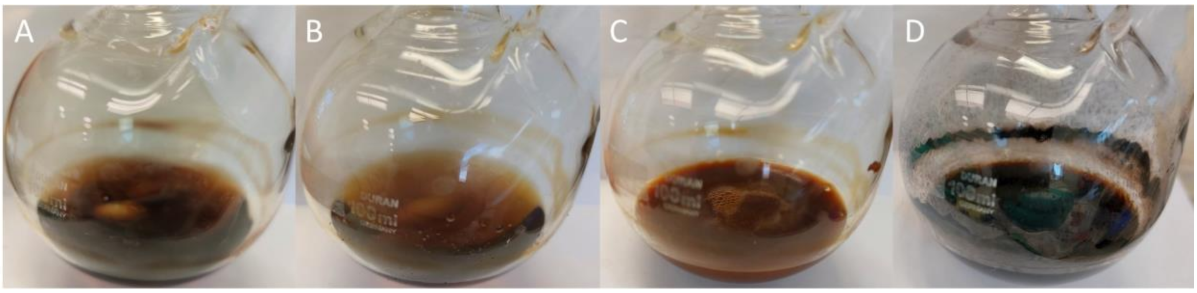

Figure S22: Photographs of FeO_x NPs (batch 156) with oleyl alcohol surfactant 2:1. A) t=50 min, B) t=60 min cooled, C) t=60 min Me_3N added, D) t=100 min.

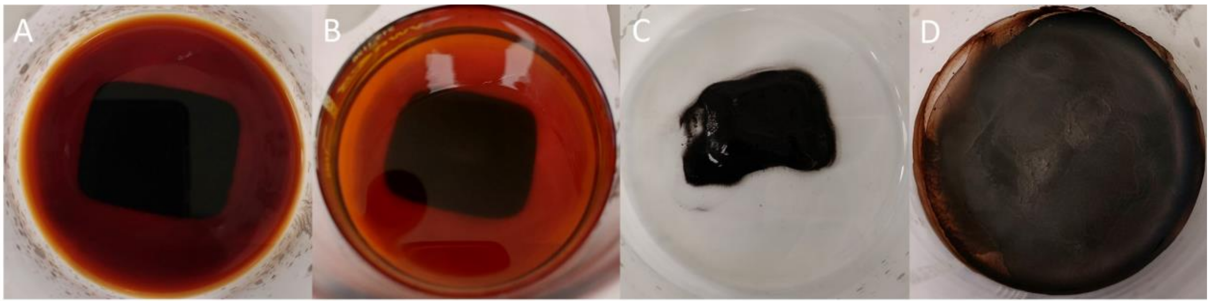

Figure S23: Precipitation on top of a magnet of FeO_x NPs (batch 156) with oleyl alcohol 2:1 as surfactant.

Figure S24: Photograph of FeO_x NPs (batch 156) with oleyl alcohol 2:1 in DCM after 12h - it is clear that the dispersion is not stable and precipitates can be identified.

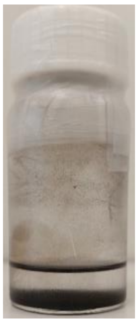

3.3.11 Synthesis of iron oxide nanoparticles with oleylamine

Oleylamine (2.86 g (70%), 7.48 mmol, 2.46 eq.), ethanol (12 mL) and iron pentacarbonyl (0.4 mL, 3.04 mmol, 1 eq.) were added to a 100 mL two-neck round bottom flask and the mixture heated at reflux (150°C Allihn condenser) for 1h at a stirring speed of 660 rpm (**Figure S7**). After 1h the mixture had turned from yellow to dark red. The mixture was initially cooled using an ice bath and then with a 20°C water bath to *room temperature* (20°C), while filling the decreasing volume of the headspace with nitrogen. To the cooled mixture trimethyl amine N-oxide dihydrate (1 g (98%), 8.82 mmol, 2.90 eq.) was added while flushing the system with nitrogen. The mixture was then heated to 130°C for 2h under nitrogen atmosphere resulting in bubbling and a dark mixture, where overpressure of gas (CO) was allowed to leave the system in the first few minutes. After 2h the temperature was increased to 150°C for 1h, where a precipitation on the side of the flask was observed. The reaction was stopped by cooling with an ice bath and subsequently with a water bath until but *not further* than 20°C (whole cooling process: 1 min), while flushing with nitrogen. The nanoparticles were decanted into a beaker (500 mL) and precipitated from ethanol (200 mL). The magnetic nanoparticles were pulled out of solution (212 mL) by a magnet under the beaker for 1h. The ethanol was decanted off and the particles were washed once with ethanol (50 mL). Dried *via* constant air flow, the particles were dissolved in dichloromethane (10 mL) and stored in nitrogen atmosphere and darkness at 5°C. The nanoparticles were stable in DCM overnight, as shown in **Figure S25** and **Figure S26**. The approximate yields for 1:1, 2:1 and 3:1 molar ratios of surfactants were 459 mg, 779 mg and 865 mg, respectively.

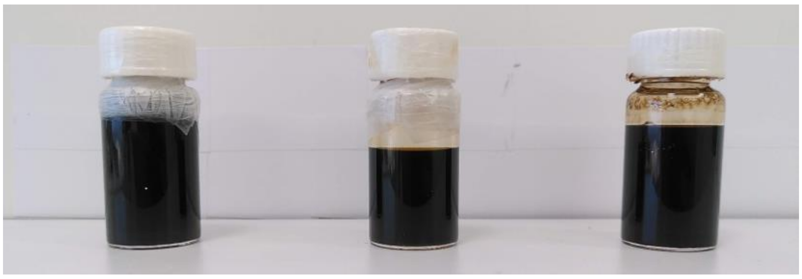

Figure S25: Photograph of FeO_x NPs with oleylamine in DCM after 12h - it is clear that the dispersions are stable and no precipitates can be identified; ratio 1:1 (batch 113) left, ratio 2:1 (batch 114) middle and ratio 3:1 (batch 115) right.

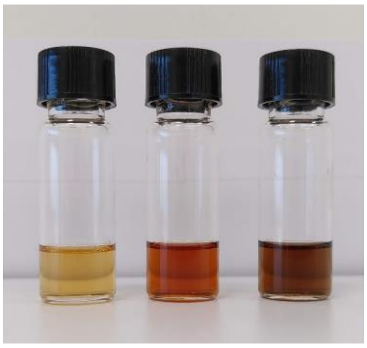

Figure S26: Oleylamine surfactant batches diluted to 1 mg mL^{-1}; ratio 1:1 (batch 113) left, ratio 2:1 (batch 114) middle and ratio 3:1 (batch 115) right.

3.3.12 Synthesis of iron oxide nanoparticles with N-oleylsarcosine

N-oleylsarcosine (2.48 g, 7.01 mmol, 2.31 eq.), ethanol (12 mL) and iron pentacarbonyl (0.4 mL, 3.04 mmol, 1 eq.) were added to a 100 mL two-neck round bottom flask and the mixture heated at reflux (150°C Allihn condenser) for 1h at a stirring speed of 660 rpm (**Figure S7**). After 1h the mixture had turned from yellow to an emulsion of yellow/white (**Figure S27 (A)**). The mixture was initially cooled using an ice bath and then with a 20°C water bath to *room temperature* (20°C) **(B)**, while filling the decreasing volume of the headspace with nitrogen. To the cooled mixture trimethyl amine N-oxide dihydrate (1 g (98%), 8.82 mmol, 2.90 eq.) was added while flushing the system with nitrogen, which resulted in bubbling and a dark solution **(C)**. The mixture was then heated to 130°C for 2h under nitrogen atmosphere resulting in a yellow/green mixture **(D),** where overpressure of gas (CO) was allowed to leave the system in the first few minutes. After 2h the temperature was increased to 150°C for 1h. The reaction was stopped by cooling with an ice bath and subsequently with a water bath until but *not further* than 20°C (whole cooling process: 1 min, **(E)**), while flushing with nitrogen. The nanoparticles were decanted into a beaker (500 mL) and precipitated from ethanol (200 mL). The magnetic nanoparticles were pulled out of solution (212 mL) by a magnet under the beaker for 1h (**Figure S*28***). The ethanol was decanted off and the particles were washed once with ethanol (50 mL). Dried *via* constant air flow, the particles were dissolved in dichloromethane (10 mL) and stored in nitrogen atmosphere and darkness at 5°C. The nanoparticles were stable in DCM overnight, as shown in **Figure S*28***. The approximate yield was 97.5 mg per batch.

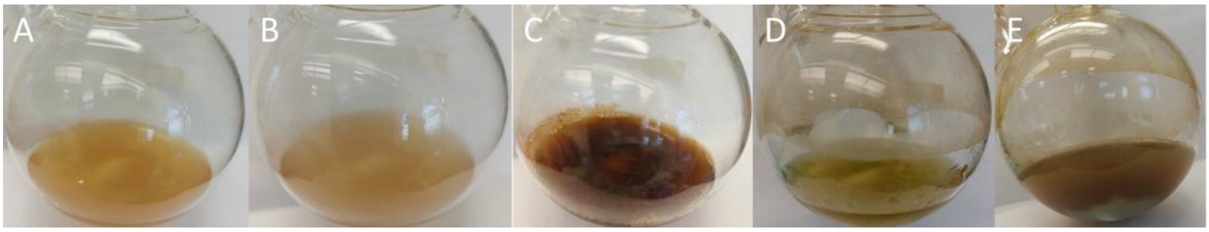

Figure S27: Photographs of FeO_x NPs (batch 158) with N-oleylsarcosine surfactant 1.5:1. A) t=50 min, B) t=60 min cooled, C) t=60 min Me_3N added, D) t=100 min, E) t=240 min cooled.

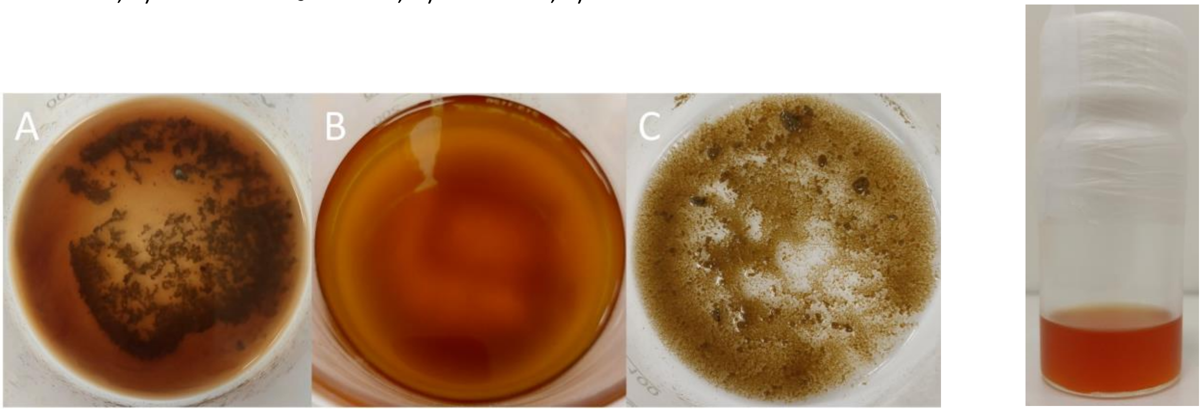

Figure S28: Precipitation on top of a magnet of FeO_x NPs (batch 158) with N-oleylsarcosine 2:1 as surfactant. Photograph of FeO_x NPs (batch 158) with N-oleylsarcosine 2:1 in DCM after 12h - it is clear that the dispersion is stable and no precipitates can be identified (bottom right).

3.3.13 Synthesis of FeO_x NPs with oleic acid supported on active charcoal (mesh 20-40)

Active charcoal (1 g, 20-40 mesh size), oleic acid (2.2 g (90%), 7.01 mmol, 2.31 eq.) and ethanol (12 mL) were added to a 100 mL two-neck round bottom and heated to 70°C for 20 min at a stirring speed of 660 rpm (**Figure S29 (A)**). Iron pentacarbonyl (0.4 mL, 3.04 mmol, 1 eq.) was added and the mixture heated at reflux (150°C Allihn condenser) for 1h at a stirring speed of 660 rpm **(B)**. The mixture was initially cooled using an ice bath and then with a 20°C water bath to *room temperature* (20°C) **(C)**, while filling the decreasing volume of the headspace with nitrogen. To the cooled mixture trimethyl amine N-oxide dihydrate (1 g, 8.82 mmol, 98%) was added while flushing the system with nitrogen, which resulted in bubbling **(D)**. The mixture was then heated to 130°C for 2h under nitrogen atmosphere **(E)**, where overpressure of gas (CO) was allowed to leave the system in the first few minutes. After 2h the temperature was increased to 150°C for 1h. The reaction was stopped by cooling with an ice bath and subsequently with a water bath until but *not further* than 20°C (whole cooling process: 1 min, **(F)**), while flushing with nitrogen. The nanoparticles were decanted into a beaker (500 mL) and precipitated from ethanol (200 mL). The magnetic nanoparticles were pulled out of solution (212 mL) by a magnet under the beaker for 1h (**Figure S30**). The ethanol was decanted off and the particles were washed once with ethanol (50 mL). Dried *via* constant air flow, the particles were dissolved in dichloromethane (10 mL) and stored in nitrogen atmosphere and darkness at 5°C. Magnetic properties due to immobilization of FeO_x NPs on the surface of the active charcoal (mesh 20-40) were confirmed (**Figure S30**). The approximate yield was 1542 mg per batch.

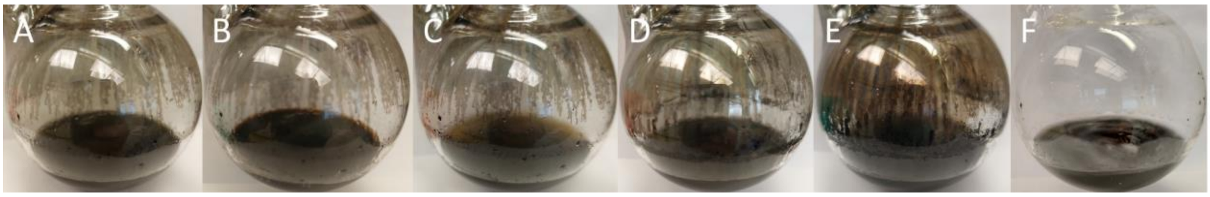

Figure S29: Photographs of FeO_x NPs (batch 159) with oleic acid surfactant 2:1, immobilized on active charcoal (mesh 20-40). A) t=15, B) t=70 min, C) t=80 min cooled, D) t=80 min Me_3N added, E) t=120 min, F) t=260 min cooled.

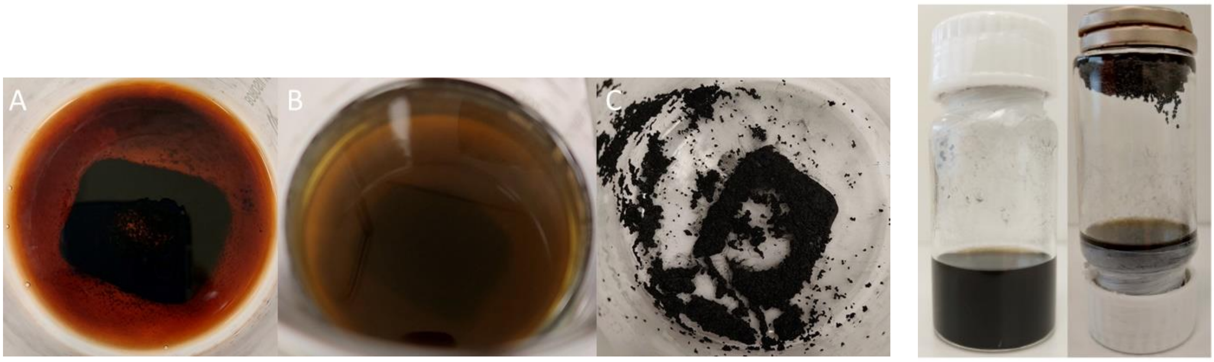

Figure S30: Precipitation on top of a magnet of FeO_x NPs (batch 159) with oleic acid surfactant 2:1, immobilized on active charcoal (mesh 20-40). Magnetic properties of the active charcoal (mesh 20-40, right) due to immobilization of FeO_x NPs on the surface, DCM used as solvent.

3.3.14 Synthesis of FeO_x NPs with oleic acid supported on active charcoal (mesh 100)

Active charcoal (1 g, 100 mesh size), oleic acid (2.2 g (90%), 7.01 mmol, 2.31 eq.) and ethanol (12 mL) were added to a 100 mL two-neck round bottom and heated to 70°C for 20 min at a stirring speed of 660 rpm (**Figure S31 (A)**). Iron pentacarbonyl (0.4 mL, 3.04 mmol, 1 eq.) was added and the mixture heated at reflux (150°C Allihn condenser) for 1h at a stirring speed of 660 rpm **(B)**. The mixture was initially cooled using an ice bath and then with a 20°C water bath to *room temperature* (20°C) **(C)**, while filling the decreasing volume of the headspace with nitrogen. To the cooled mixture trimethyl amine N-oxide dihydrate (1 g, 8.82 mmol, 98%) was added while flushing the system with nitrogen, which resulted in bubbling **(D)**. The mixture was then heated to 130°C for 2h under nitrogen atmosphere **(E)**, where overpressure of gas (CO) was allowed to leave the system in the first few minutes. After 2h the temperature was increased to 150°C for 1h. The reaction was stopped by cooling with an ice bath and subsequently with a water bath until but *not further* than 20°C (whole cooling process: 1 min, **(F)**), while flushing with nitrogen. The nanoparticles were decanted into a beaker (500 mL) and precipitated from ethanol (200 mL). The magnetic nanoparticles were pulled out of solution (212 mL) by a magnet under the beaker for 1h (**Figure S32**). The ethanol was decanted off and the particles were washed once with ethanol (50 mL). Dried *via* constant air flow, the particles were dissolved in dichloromethane (10 mL) and stored in nitrogen atmosphere and darkness at 5°C. Magnetic properties due to immobilization of FeO_x NPs on the surface of the active charcoal (mesh 100) were confirmed (**Figure S32**).

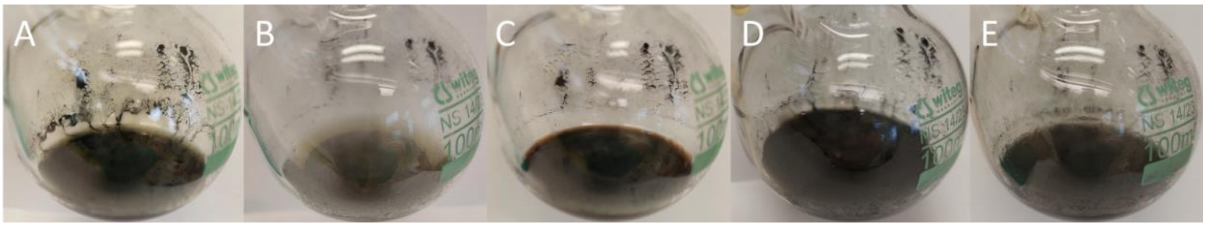

Figure S31: Photographs of FeO_x NPs (batch 183) with oleic acid surfactant 2:1, immobilized on active charcoal (mesh 100). A) t=15, B) t=80 min cooled, C) t=80 min Me_3N added, D) t=120 min, E) t=260 min cooled.

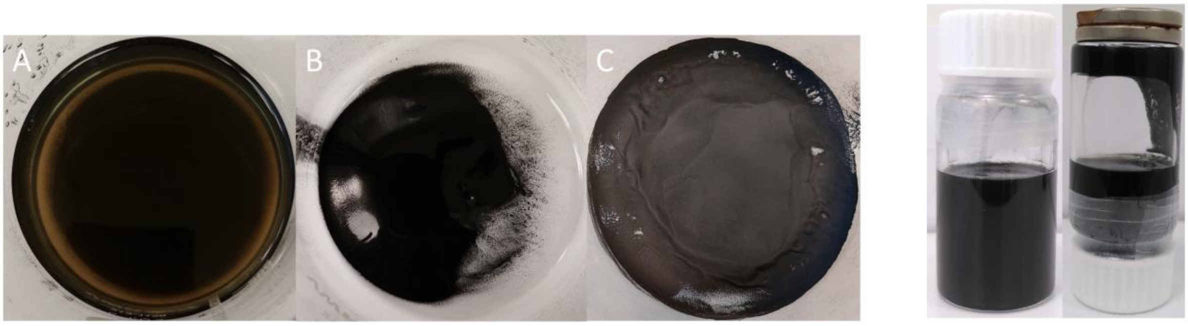

Figure S32: Precipitation on top of a magnet of FeO_x NPs (batch 183) with oleic acid surfactant 2:1, immobilized on active charcoal (mesh 100). Magnetic properties of the active charcoal (mesh 100, right) due to immobilization of FeO_x NPs on the surface, DCM used as solvent.

3.3.15 Synthesis of FeO_x NPs with oleic acid supported on graphene nanoplatelets

Graphene nanoplatelets (1 g, 25 µm particle size), oleic acid (2.2 g (90%), 7.01 mmol, 2.31 eq.) and ethanol (12 mL) were added to a 100 mL two-neck round bottom and heated to 70°C for 20 min at a stirring speed of 660 rpm (**Figure S33 (A)**). Iron pentacarbonyl (0.4 mL, 3.04 mmol, 1 eq.) was added and the mixture heated at reflux (150°C Allihn condenser) for 1h at a stirring speed of 660 rpm **(B)**. The mixture was initially cooled using an ice bath and then with a 20°C water bath to *room temperature* (20°C) **(C)**, while filling the decreasing volume of the headspace with nitrogen. To the cooled mixture trimethyl amine N-oxide dihydrate (1 g (98%), 8.82 mmol, 2.90 eq.) was added while flushing the system with nitrogen, which resulted in bubbling **(D)**. The mixture was then heated to 130°C for 2h under nitrogen atmosphere **(E)**, where overpressure of gas (CO) was allowed to leave the system in the first few minutes. After 2h the temperature was increased to 150°C for 1h. The reaction was stopped by cooling with an ice bath and subsequently with a water bath until but *not further* than 20°C (whole cooling process: 1 min, **(F)**), while flushing with nitrogen. The nanoparticles were decanted into a beaker (500 mL) and precipitated from ethanol (200 mL). The magnetic nanoparticles were pulled out of solution (212 mL) by a magnet under the beaker for 1h (**Figure S34**). The ethanol was decanted off and the particles were washed once with ethanol (50 mL). Dried *via* constant air flow, the particles were dissolved in dichloromethane (10 mL) and stored in nitrogen atmosphere and darkness at 5°C. Magnetic properties due to immobilization of FeO_x NPs on the surface of the graphene nanoplatelets were confirmed (**Figure S34**). The approximate yield was 2098 mg per batch.

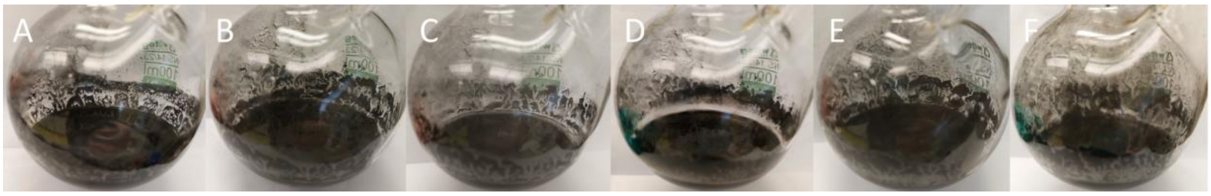

Figure S33: Photographs of FeO_x NPs (batch 160) with oleic acid surfactant 2:1, immobilized on graphene nanoplatelets. A) t=15, B) t=70 min, C) t=80 min cooled, D) t=80 min Me_3N added, E) t=120 min, E) t=260 min cooled.

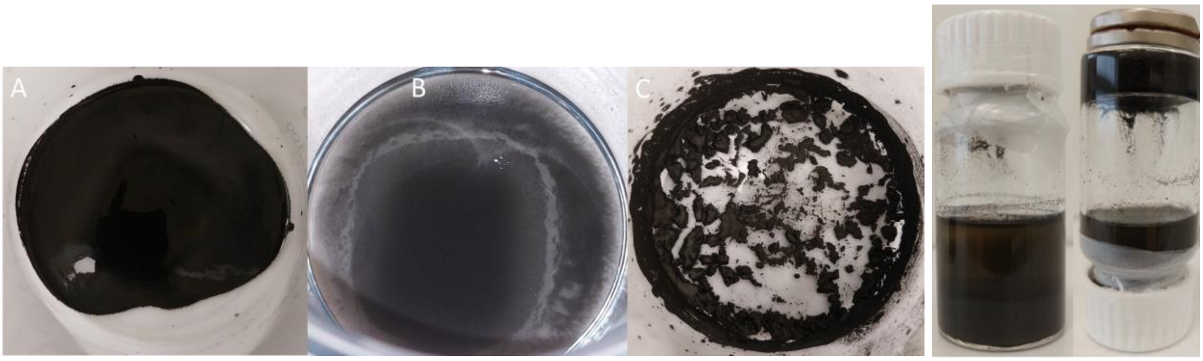

Figure S34: Precipitation on top of a magnet of FeO_x NPS (batch 160) with oleic acid surfactant 2:1, immobilized on graphene nanoplatelets. Magnetic properties of the graphene (right) due to immobilization of FeO_x NPs on the surface, DCM used as solvent.

3.3.16 Immobilization on TiO_2, lignin, cellulose and chitosan

Immobilization of the nanoparticles was attempted on TiO_2, which itself is also photocatalyst for hydrogen peroxide production.[9] The synthesis was unsuccessful as the nanoparticles could not properly attach to TiO_2. As the obtained FeO_x NP are capped with oleic acid surfactant, their hydrophobic properties are suspected to hinder attachment to hydrophilic TiO_2. Accordingly, it was opted to immobilize the FeO_x material on carbon-based materials: Cellulose, chitosan and lignin were chosen as naturally occurring polymeric materials. Successful immobilization was obtained, but FeO_x@cellulose and FeO_x@chitosan did not lead to active NPs for photochemical production of H_2O_2.

Immobilization on lignin (FeO_x@lignin) led to enhanced activity, more than FeO_x NP produced without immobilization. However, the blank of pure lignin in water yielded higher production of peroxides as shown in **Figure S35**. This suggests that iron-immobilization on lignin induced Fenton chemistry (decomposition of H_2O_2) rather than enhancing the production, while hydrogen peroxide and other peroxides were formed stoichiometrically from pure lignin as previously reported.[10-13]

Successful immobilization on graphene nanoplatelets was achieved, but no enhancement compared to non-immobilized nanoparticles was obtained. Interestingly, photochemical production by immobilization of FeO_x NPs on activated charcoal (mesh 20-40) was achieved. Immobilization on 250 mg, 500 mg, 1000 mg and 1250 mg activated charcoal (mesh 20-40) were tested, where 1250 mg activated charcoal (mesh 20-40) resulted in best results.

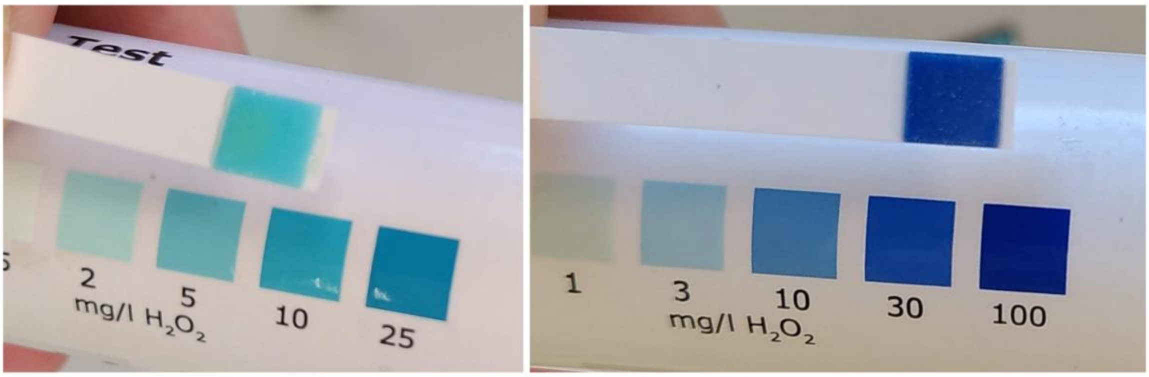

Figure S35: H_2O_2 production of FeO_x NPs immobilized on lignin (left) and lignin Blank (right).

4. Catalyst properties

4.1 DLS results

A Zetasizer Ultra Red (ZSU3305) from Malvern Panalytical was used for dynamic light scattering experiments. All samples (1 mL with a concentration of 1 mg mL^{-1}) were measured at 298.15 K.

4.1.1 FeO_x scope comparison

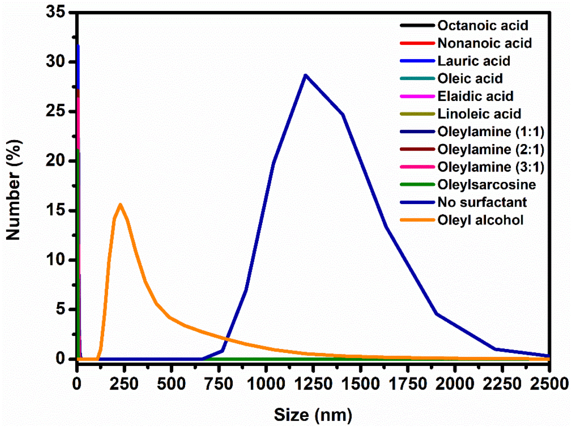

Figure S36: Particle size distribution of all synthesized batches, measured by DLS at concentration of 1 mg mL^{-1}.

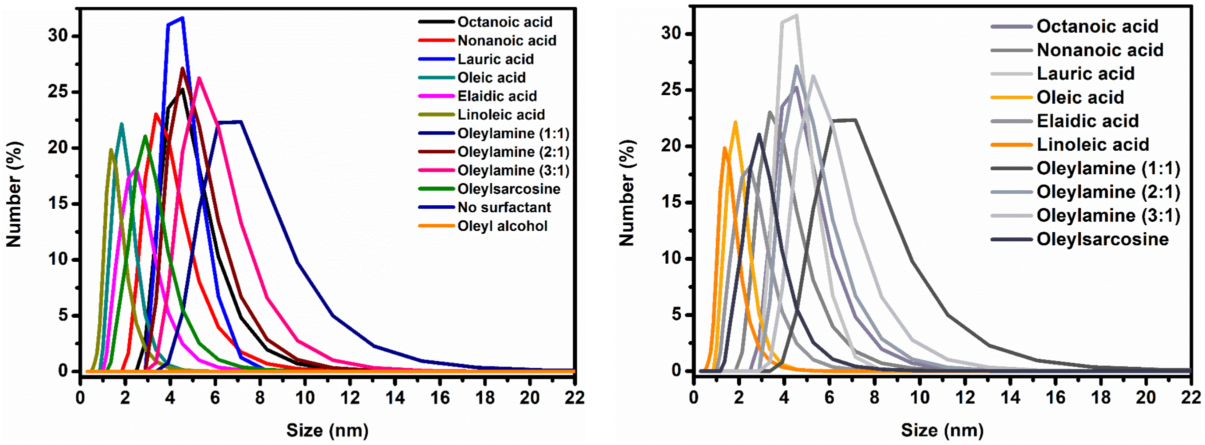

Figure S37: Comparison of particle size for the whole scope of the synthesized FeO_x NPs. Active catalyst materials (oleic acid and linoleic acid) have been depicted in orange/yellow, while the rest is depicted in greyscale.

Table S1: Particle size and diffusion coefficient of FeO_x NP scope with concentration 1 mg mL^{-1}, obtained *via* DLS measurements.

Surfactant (Batch #)	Measured in solvent	Particle size (nm) by number %	Diffusion coefficient ($\mu m^2\ s^{-1}$)
No surfactant (143)	DCM	1299±100	0.33±0.09
Oleyl alcohol (156)	DCM	377.35±166	1.14±0.05
Octanoic acid (161)	DCM	4.75±0.27	109.9±8.81
Nonanoic acid (162)	DCM	3.86±0.43	112.4±3.68
Lauric acid (171)	DCM	3.85±1.19	0.39±0.05
Oleic acid (173), Acros organics	DCM	1.88±0.37	8.13±1.49
Oleic acid (174), Sigma aldrich	DCM	1.97±0.31	7.27±1.29
Oleic acid (131)	THF	3.82±0.42	1.20±0.67
Elaidic acid (172)	DCM	2.49±0.55	18.64±14.61
Linoleic acid (144)	DCM	1.54±0.26	35.28±13.83
Oleylamine (113, 1:1)	DCM	7.41±0.71	52.14±0.36
Oleylamine (114, 2:1)	DCM	5.09±0.01	108.7±0.26
Oleylamine (115, 3:1)	DCM	5.87±0.23	45.66±2.89
Oleylsarcosine (158)	DCM	3.00±0.56	47.24±2.81

4.2 Transmission electron microscopy (TEM), scanning transmission electron microscopy (STEM) and energy-dispersive X-ray spectroscopy (EDX) of FeO_x NPs

TEM characterization:

A PHILIPS CM 120 Cryo electron microscope with 120 keV was used to take the TEM images. The sample grid was prepared by dropping 5 µL of the solution (1 mg/mL in THF or DCM) onto an ultrathin carbon film coated copper grid (or graphene grid in the case of oleic acid and linoleic acid). After 30 seconds of drying the grid was washed with 5 µL ethanol (EtOH) and any surplus solvent was dried on a filter paper. In the case of oleic acid and linoleic acid (1 mg/mL THF), the graphene grids were placed in a desiccator at full vacuum over-night.

STEM characterization:

A Tecnai T20 cryo-electron microscope with 200 keV was used to take the Transmission electron microscopy (TEM) and scanning transmission electron (STEM) images. EDX analysis was performed with a SDD EDX detector from Oxford xmax instruments, and the elemental ratio was calculated *via* INCA software.

4.2.1 FeO_x NP with oleic acid (*cis*)

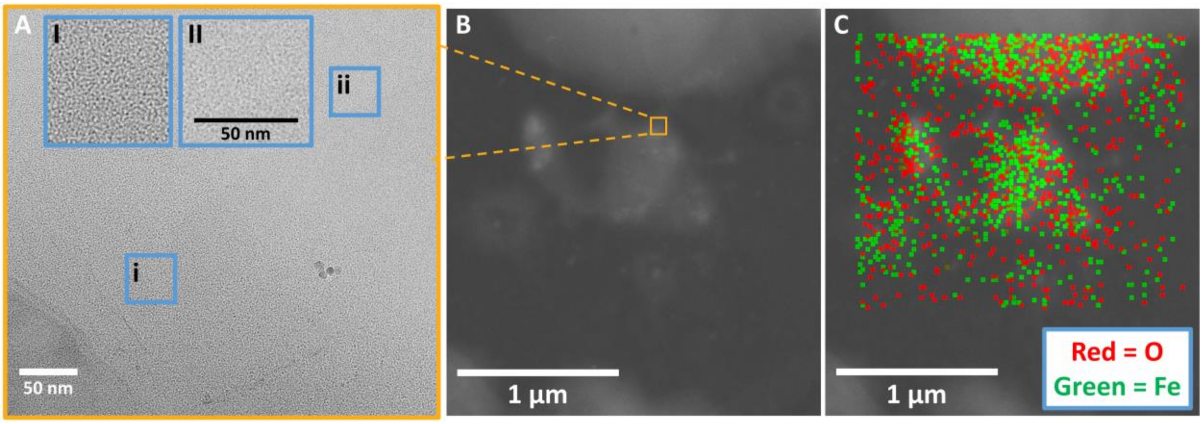

Figure S38: A) Transmission electron microscopy of FeO_x (batch 131, oleic acid 2:1, Acros Organics, 1 mg mL^{-1} in THF), at a magnification of 100000x (inlet: zoomed); particle size by DLS 1.94±0.34 nm. B) Scanning transmission electron microscopy of FeO_x (batch 131, oleic acid 2:1, 1 mg mL^{-1} in THF), inlet: zoom towards A. C) EDX of FeO_x (batch 131, oleic acid 2:1, 1 mg mL^{-1} in THF), drying spots of solvents contain more FeO_x NP and concentration decreases towards the edges of the droplets; oxygen is depicted in red – iron in green.

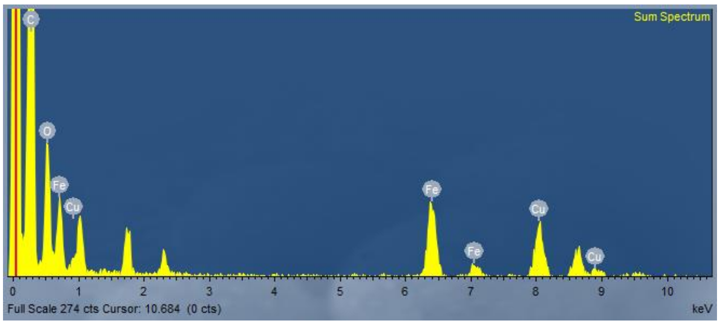

Figure S39: Full spectrum of the elemental analysis (EDX) of FeO_x (batch 131, oleic acid 2:1, Acros Organics, 1 mg mL^{-1} in THF).

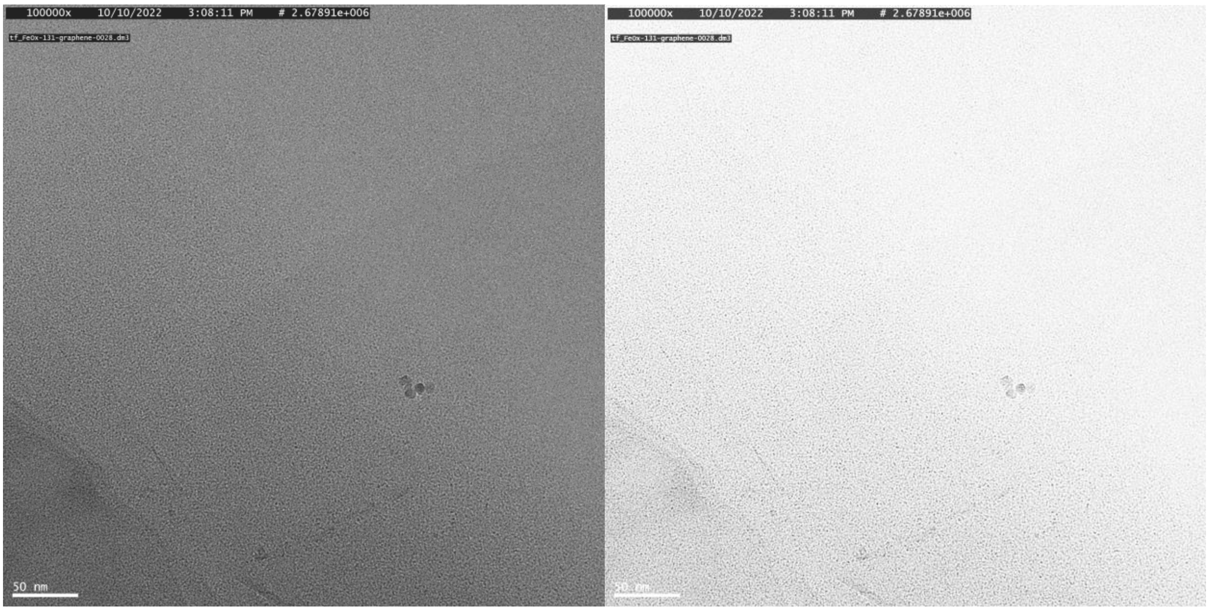

Figure S40: Transmission electron microscopy of FeO_x (batch 131, oleic acid 2:1, Acros Organics, 1 mg mL^{-1} in THF), at a magnification of 100000x; particle size by DLS 1.94±0.34 nm, higher contrast for better visibility (right).

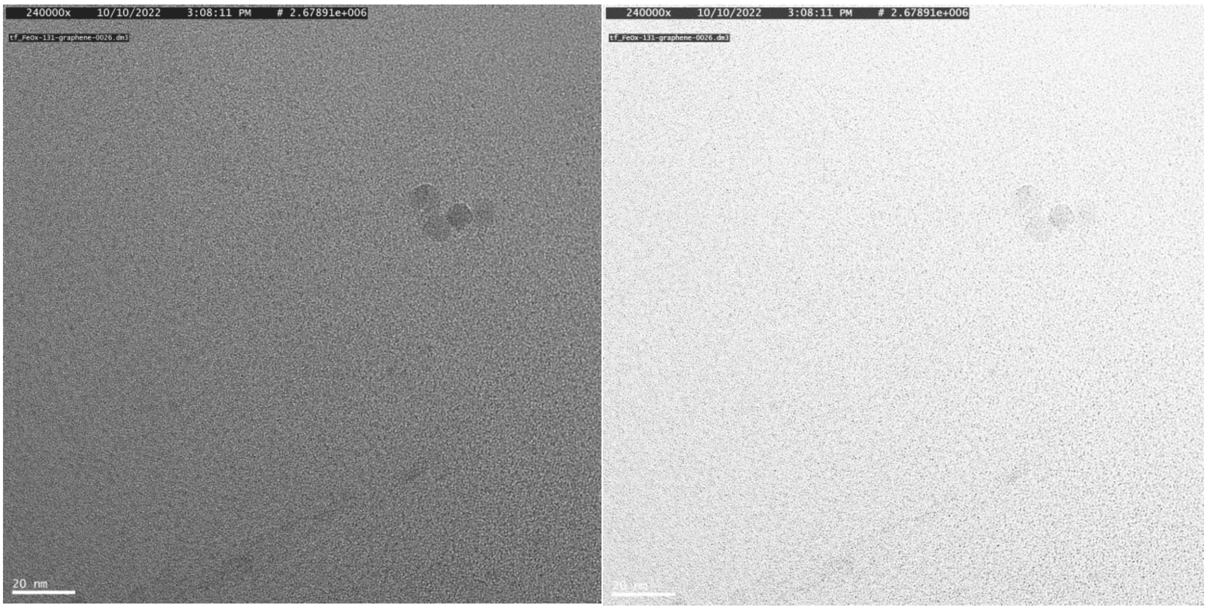

Figure S41: Transmission electron microscopy of FeO_x (batch 131, oleic acid 2:1, Acros Organics, 1 mg mL^{-1} in THF), at a magnification of 240000x; particle size by DLS 1.94±0.34 nm, higher contrast for better visibility (right).

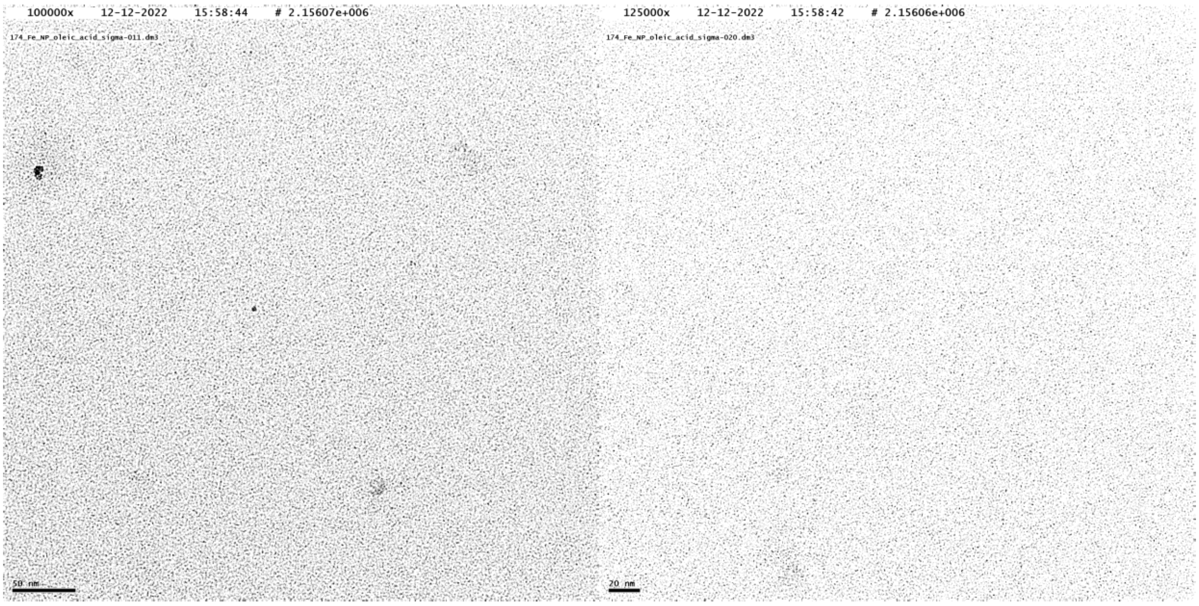

Figure S42: left) Transmission electron microscopy of FeO_x (batch 174, oleic acid 2:1, Sigma Aldrich, 1 mg mL^{-1} in THF), at a magnification of 100000x; particle size by DLS 1.94±0.34 nm, higher contrast for better visibility. right) Transmission electron microscopy of FeO_x (batch 174, oleic acid 2:1, Sigma Aldrich, 1 mg mL^{-1} in THF), at a magnification of 125000x; particle size by DLS 1.94±0.34 nm, higher contrast for better visibility.

4.2.2 FeO_x NP with linoleic acid (*cis*,*cis*)

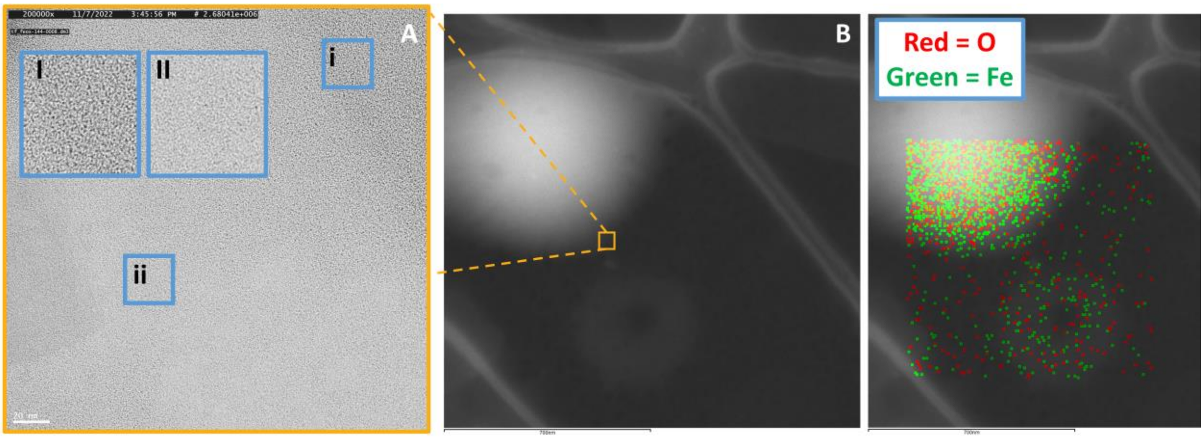

Figure S43: A) Transmission electron microscopy of FeO_x (batch 144, linoleic acid 2:1, 1 mg mL^{-1} in DCM), at a magnification of 200000x (inlet: zoomed); particle size by DLS 1.54±0.26 nm. B) Scanning transmission electron microscopy of FeO_x (batch 144, linoleic acid 2:1, 1 mg mL^{-1} in DCM), inlet: zoom towards A. C) EDX of FeO_x (batch 144, linoleic acid 2:1, 1 mg mL^{-1} in DCM), drying spots of solvents contain more FeO_x NP and concentration decreases towards the edges of the droplets; oxygen is depicted in red – iron in green.

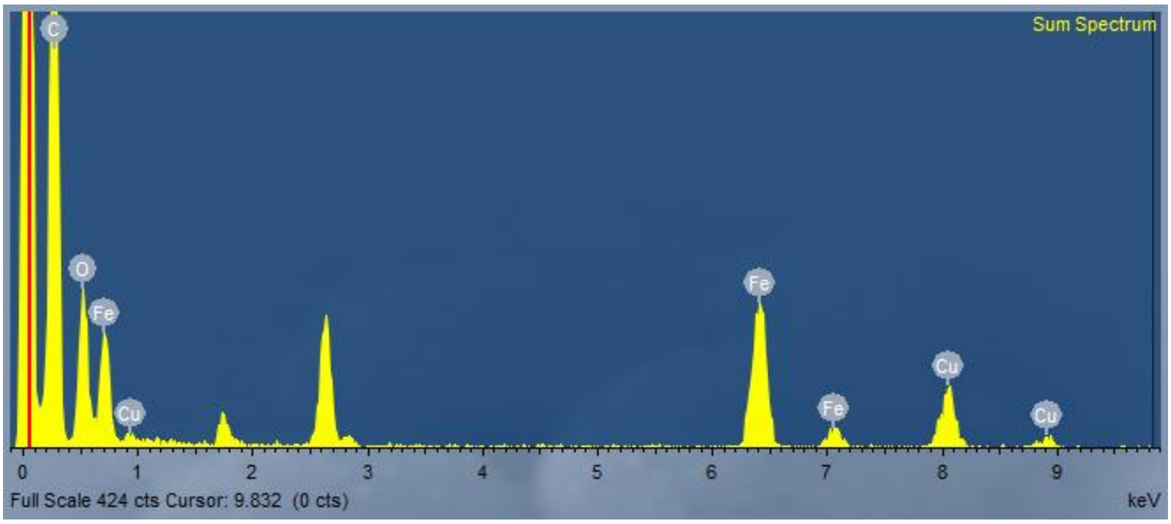

Figure S44: Full spectrum of the elemental analysis (EDX) of FeO_x (batch 144, linoleic acid 2:1, 1 mg mL^{-1} in DCM).

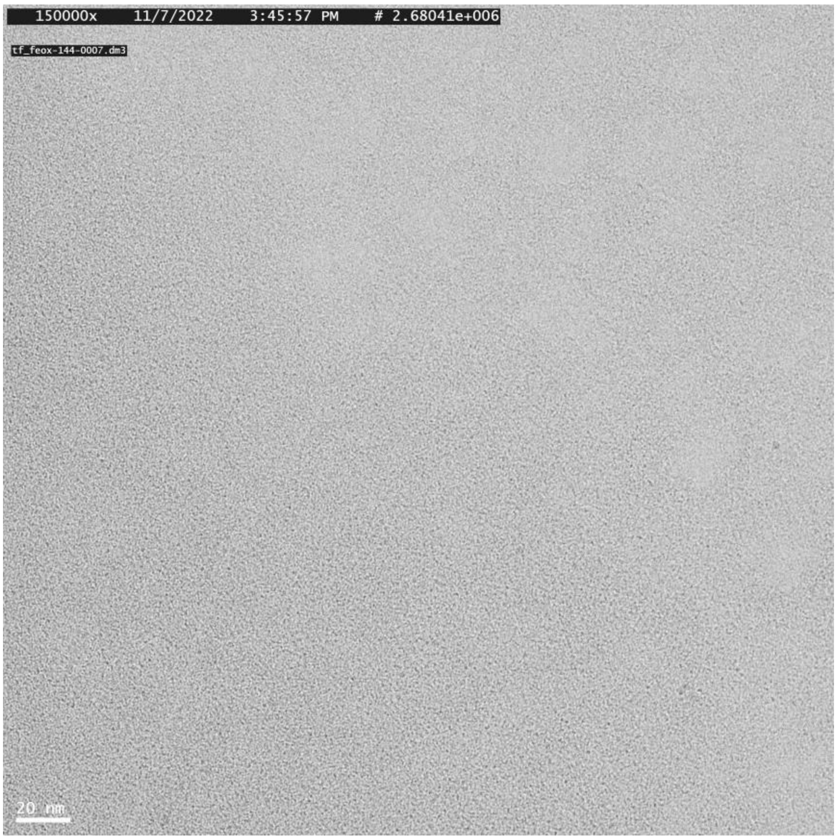

Figure S45: Transmission electron microscopy of FeO_x (batch 144, linoleic acid 2:1, 1 mg mL^{-1} in DCM), at a magnification of 150000x; particle size by DLS 1.54±0.26 nm, higher contrast for better visibility.

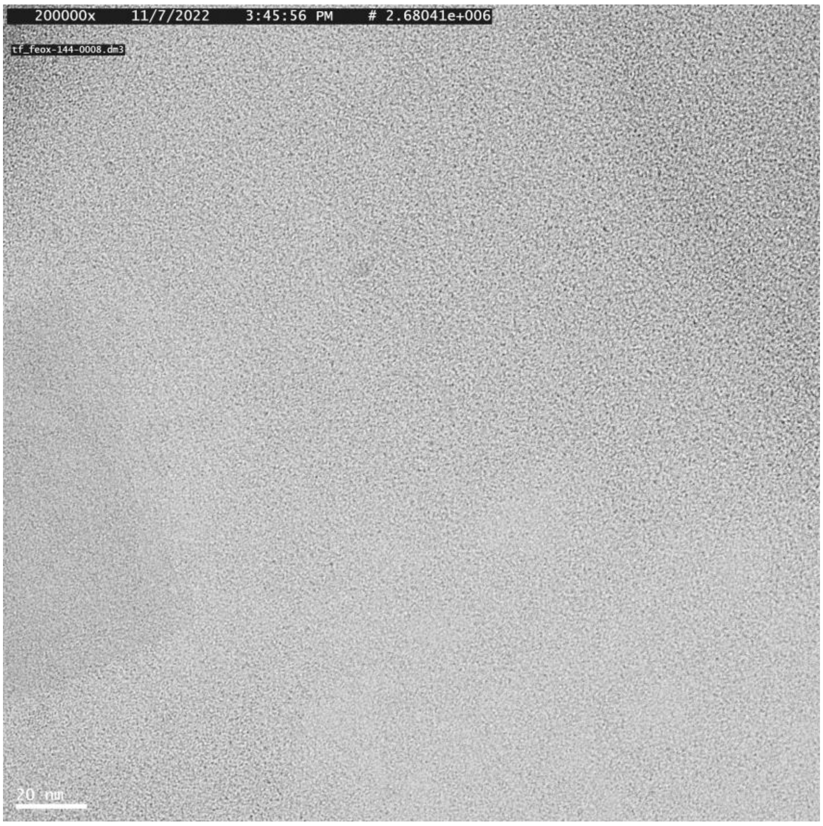

Figure S46: Transmission electron microscopy of FeO_x (batch 144, linoleic acid 2:1, 1 mg mL^{-1} in DCM), at a magnification of 200000x; particle size by DLS 1.54±0.26 nm, higher contrast for better visibility.

4.2.1.2 FeO_x NPs with oleic acid immobilized on active charcoal mesh 20-40

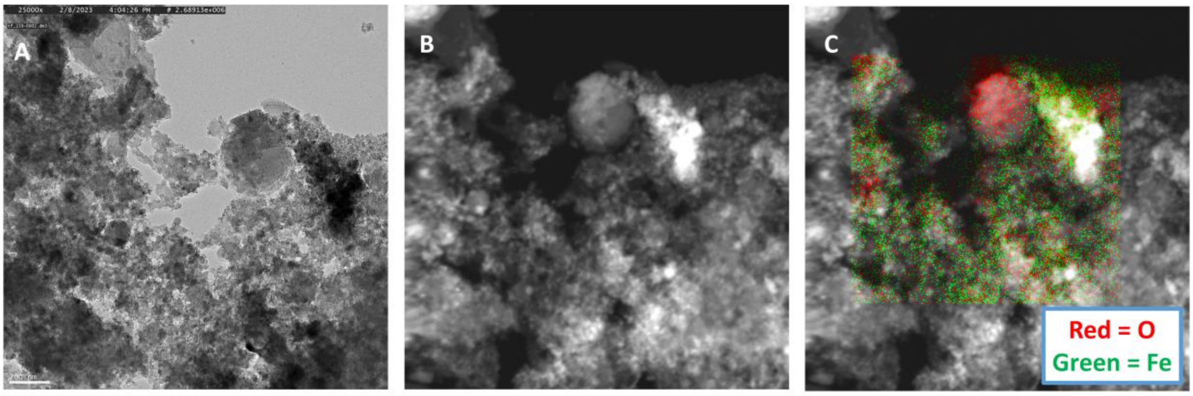

Figure S47: A) Transmission electron microscopy of FeO_x NPs immobilized on active charcoal (batch 159, oleic acid 2:1, active charcoal mesh 20-40, 1 mg mL^{-1} in DCM), at a magnification of 25000x. B) Scanning transmission electron microscopy FeO_x NPs immobilized on active charcoal C) EDX of FeO_x NPs immobilized on active charcoal (batch 159), successful immobilization of FeO_x NPs on the support was achieved; oxygen is depicted in red – iron in green.

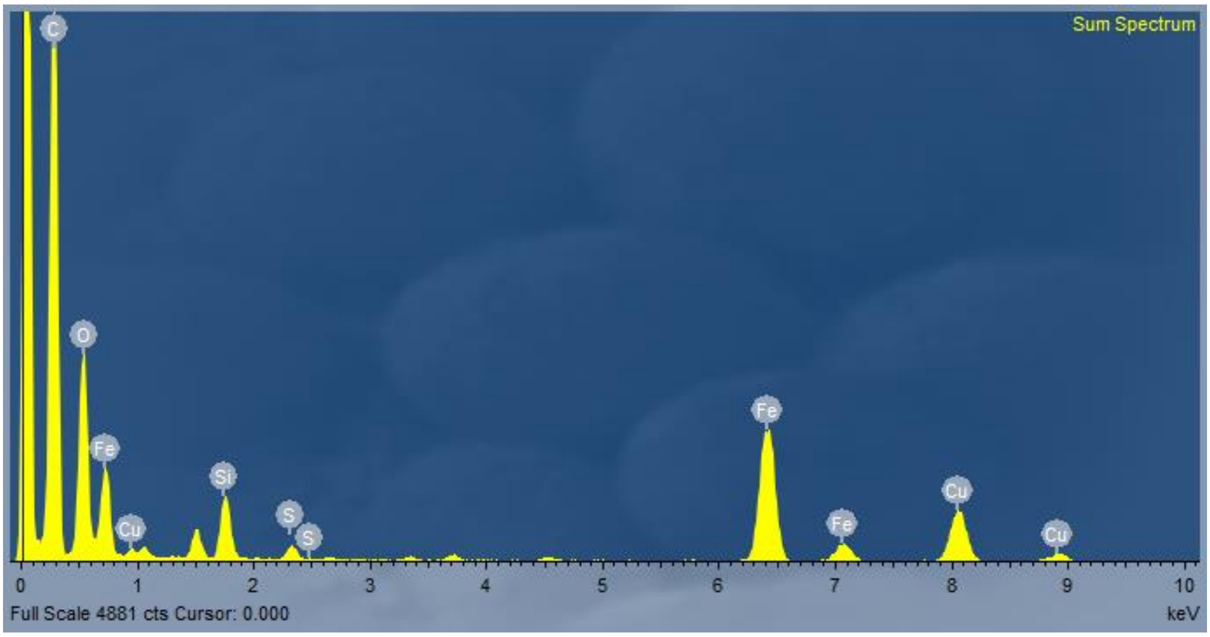

Figure S48: Full spectrum of the elemental analysis (EDX) of FeO_x NPs immobilized on active charcoal (batch 159, oleic acid 2:1, active charcoal mesh 20-40, 1 mg mL^{-1} in DCM).

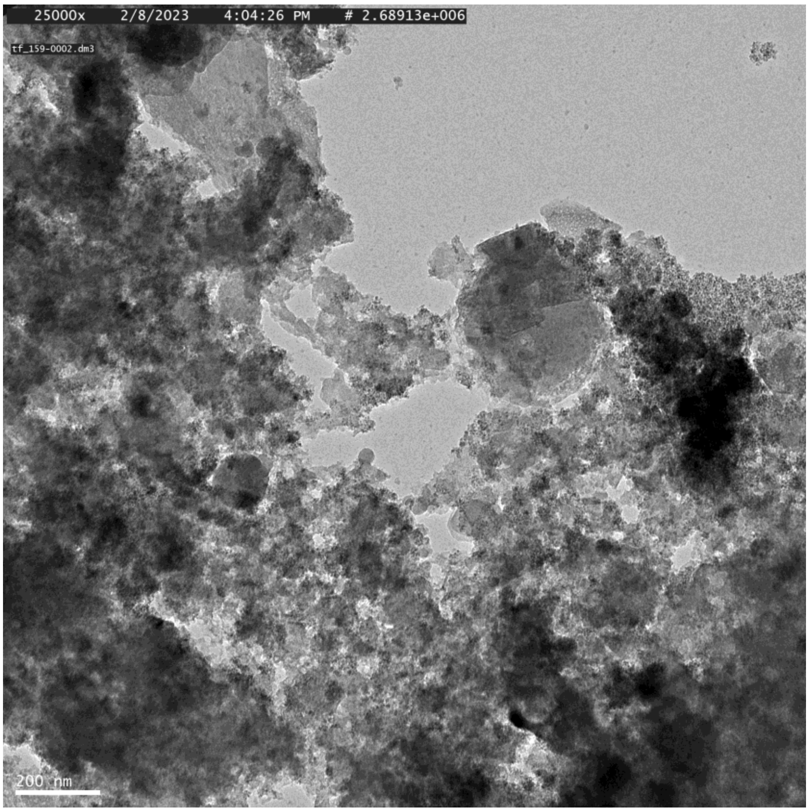

Figure S49: Transmission electron microscopy of FeO_x NPs immobilized on active charcoal (batch 159, oleic acid 2:1, active charcoal mesh 20-40, 1 mg mL^{-1} in DCM), at a magnification of 25000x.

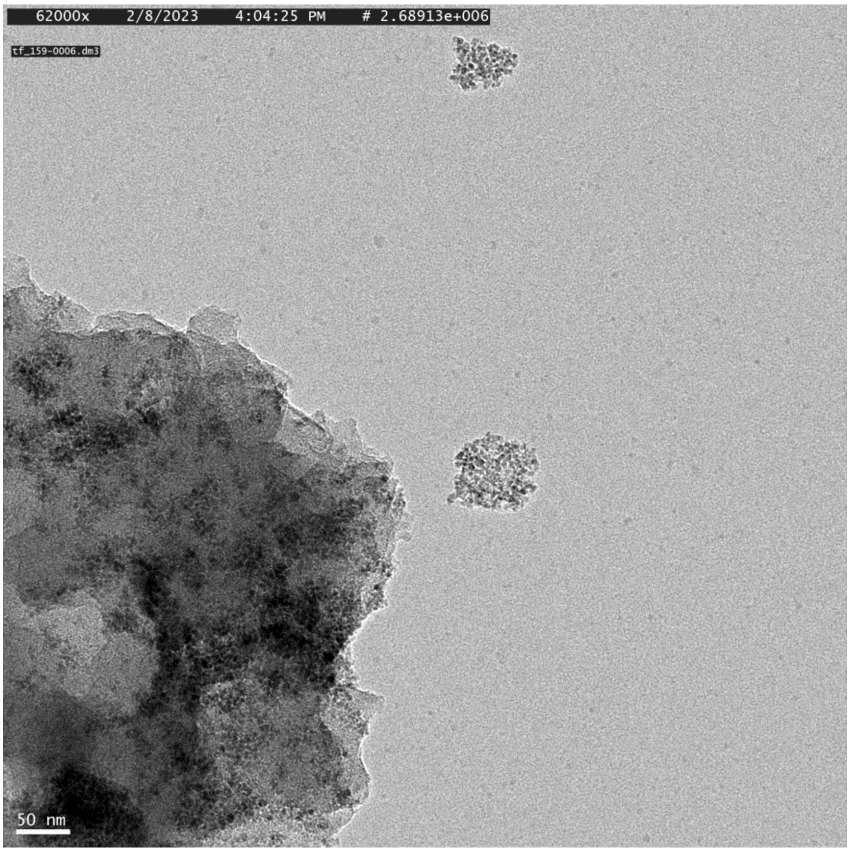

Figure S50: Transmission electron microscopy of FeO_x NPs immobilized on active charcoal (batch 159, oleic acid 2:1, active charcoal mesh 20-40, 1 mg mL^{-1} in DCM), at a magnification of 62000x.

4.2.1.3 FeO_x NPs with oleic acid immobilized on active charcoal mesh 100

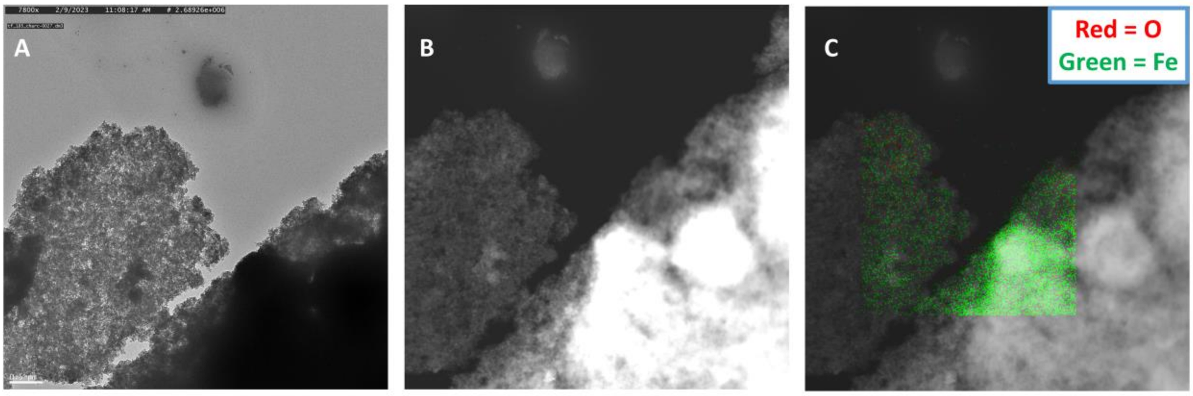

Figure S51: A) Transmission electron microscopy of FeO_x NPs immobilized on active charcoal (batch 183, oleic acid 2:1, active charcoal mesh 100, 1 mg mL^{-1} in DCM), at a magnification of 19000x. B) Scanning transmission electron microscopy FeO_x NPs immobilized on active charcoal C) EDX of FeO_x NPs immobilized on active charcoal (batch 183), successful immobilization of FeO_x NPs on the support was achieved; oxygen is depicted in red – iron in green.

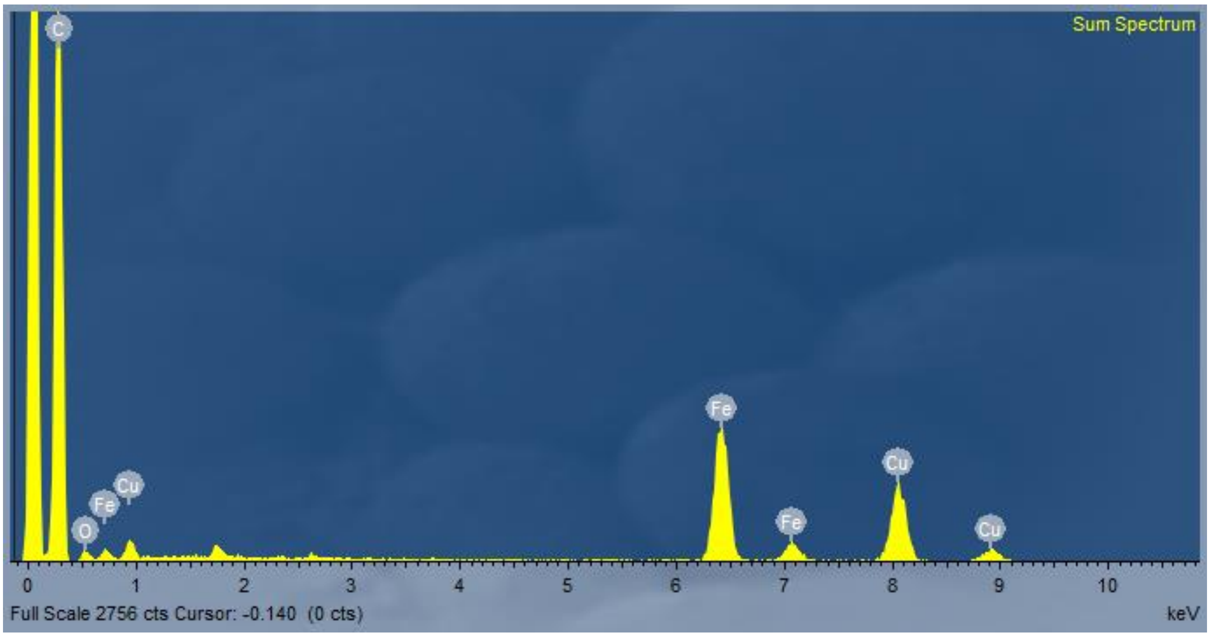

Figure S52: Full spectrum of the elemental analysis (EDX) of FeO_x NPs immobilized on active charcoal (batch 183, oleic acid 2:1, active charcoal mesh 100, 1 mg mL^{-1} in DCM).

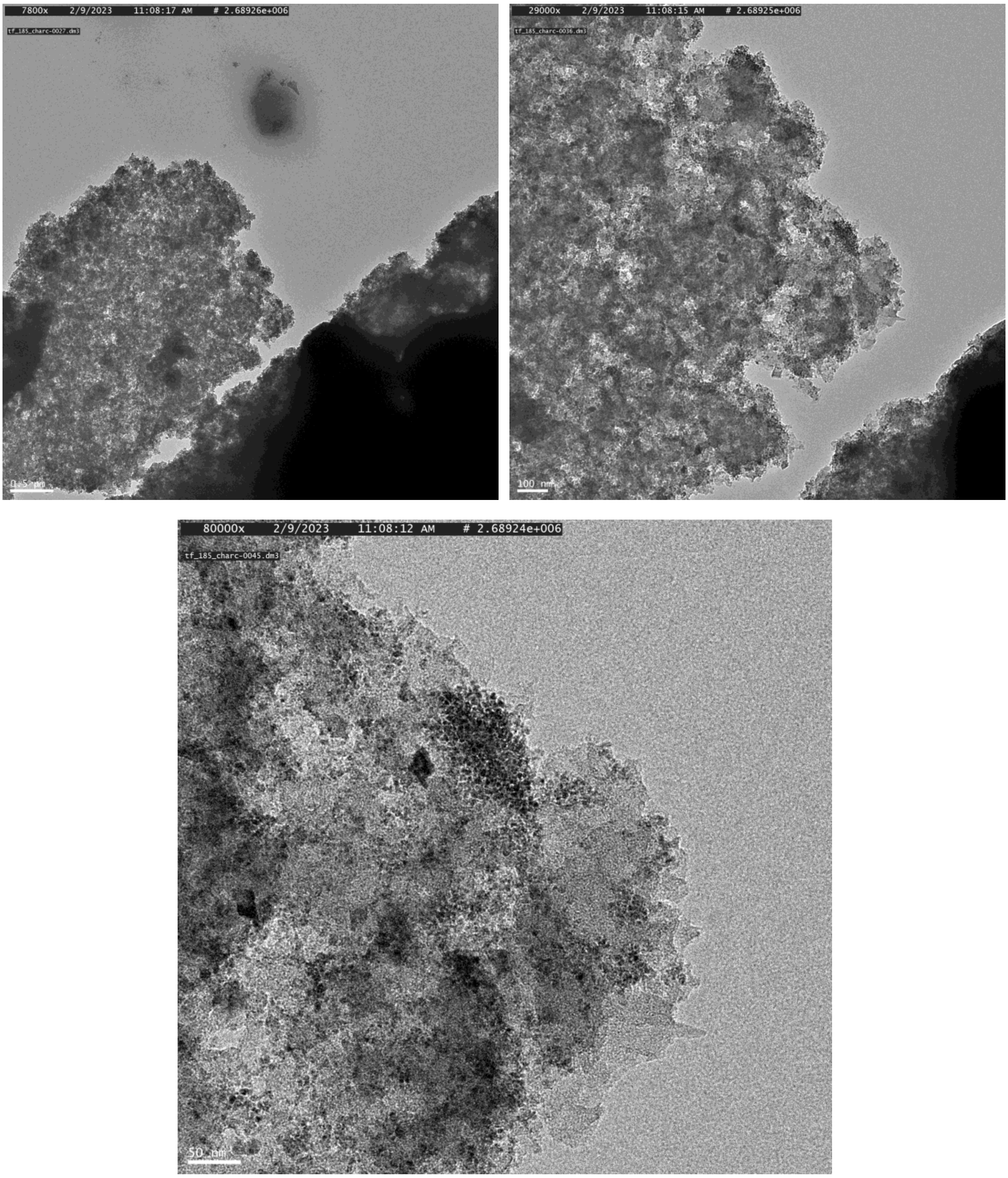

Figure S53: Transmission electron microscopy of FeO_x NPs immobilized on active charcoal (batch 183, oleic acid 2:1, active charcoal mesh 100, 1 mg mL^{-1} in DCM), at a magnification of 7800x (top left); at a magnification of 29000x (top right); at a magnification of 80000x (bottom).

4.2.1.4 FeO_x NPs with oleic acid immobilized on graphene nanoplatelets

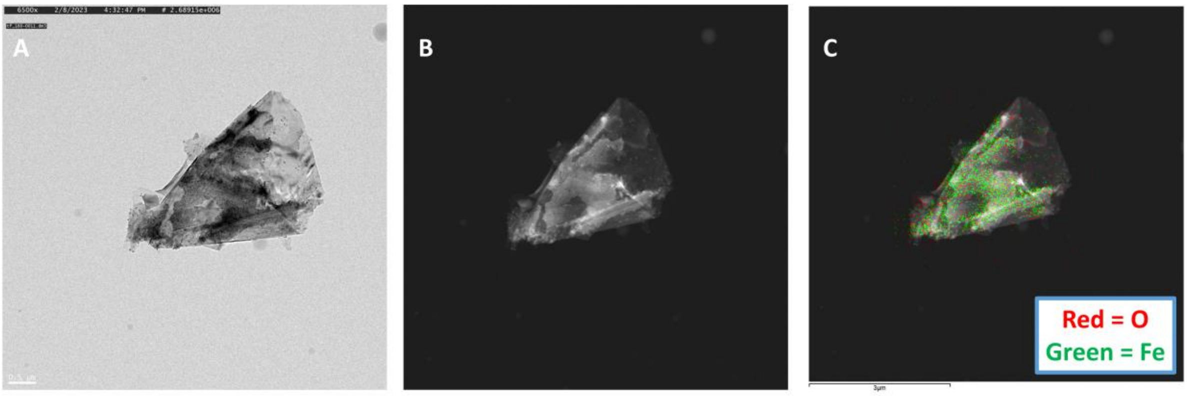

Figure S54: A) Transmission electron microscopy of FeO_x NPs immobilized on graphene nanoplatelets (batch 160, oleic acid 2:1, 1 mg mL^{-1} in DCM), at a magnification of 6500x. B) Scanning transmission electron microscopy FeO_x NPs immobilized on graphene nanoplatelets C) EDX of FeO_x NPs immobilized on nanoplatelets (batch 160), successful immobilization of FeO_x NPs on the support was achieved; oxygen is depicted in red – iron in green.

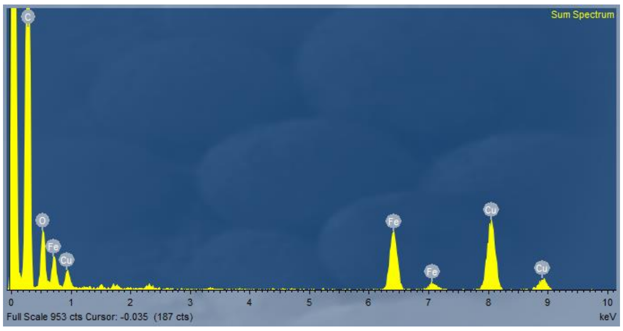

Figure S55: Full spectrum of the elemental analysis (EDX) of FeO_x NPs immobilized on graphene nanoplatelets (batch 160, oleic acid 2:1, 1 mg mL^{-1} in DCM).

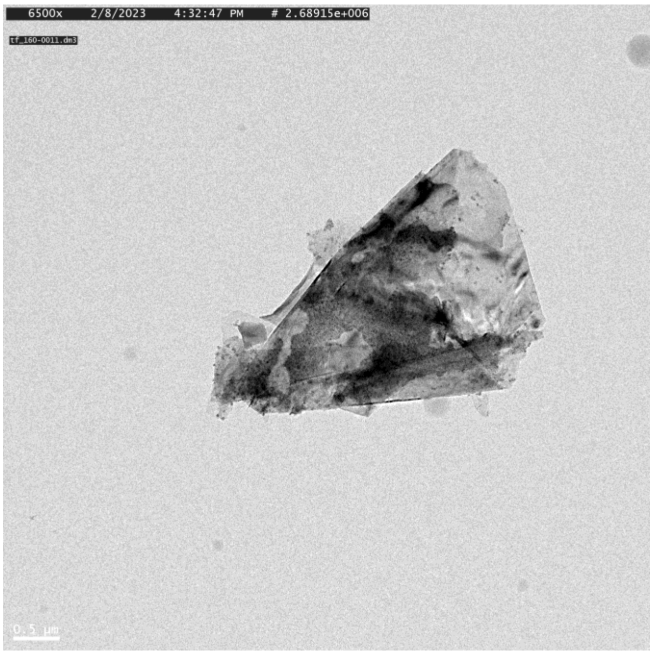

Figure S56: Transmission electron microscopy of FeO_x NPs immobilized on graphene nanoplatelets (batch 160, oleic acid 2:1, 1 mg mL^{-1} in DCM), at a magnification of 6500x.

4.2.5 FeO_x without surfactant

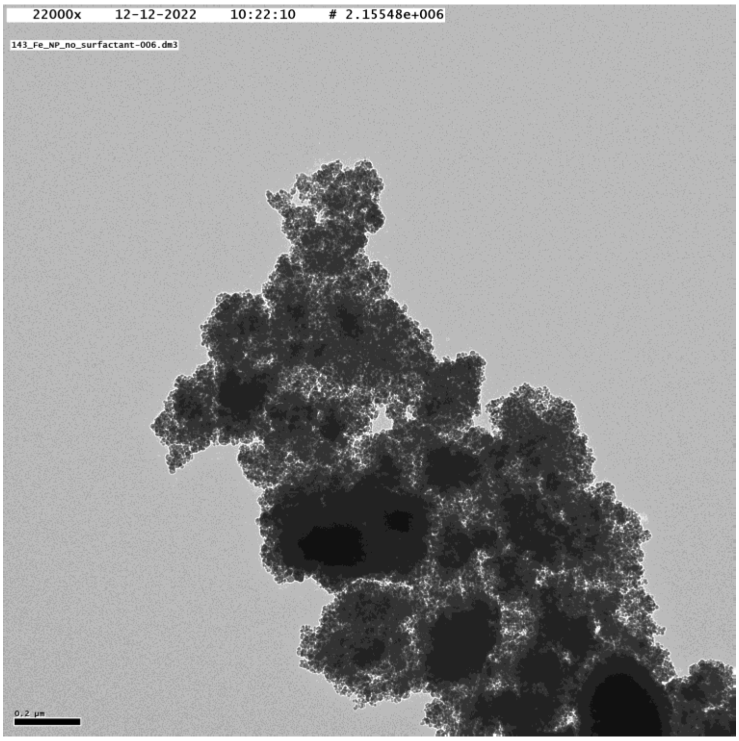

Figure S57: Transmission electron microscopy of FeO_x NP without surfactant (batch 143, no surfactant, 1 mg mL^{-1} in DCM); particle size by DLS of 1299±100 nm. Magnification of 22000x.

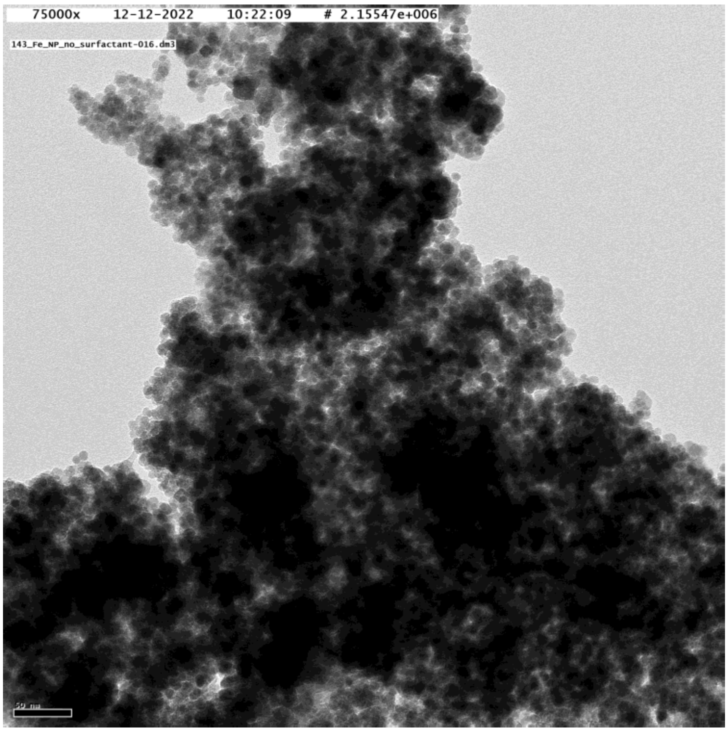

Figure S58: Transmission electron microscopy of FeO_x NP without surfactant (batch 143, no surfactant, 1 mg mL^{-1} in DCM); particle size by DLS of 1299±100 nm. Magnification of 75000x.

4.2.6 FeO_x NPs with octanoic acid

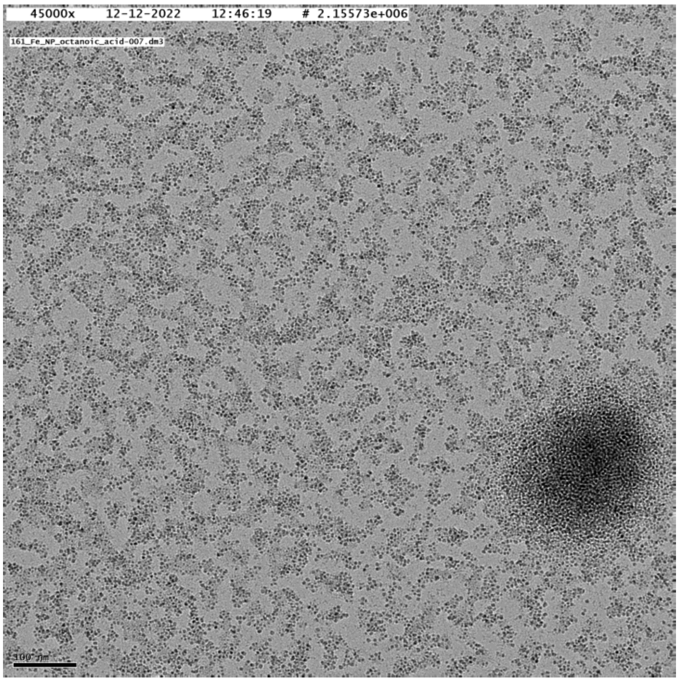

Figure S59: Transmission electron microscopy of FeO_x NP with octanoic acid (batch 161, octanoic acid 1:1, 1 mg mL^{-1} in DCM); particle size by DLS of 4.75±0.27 nm. Magnification of 45000x.

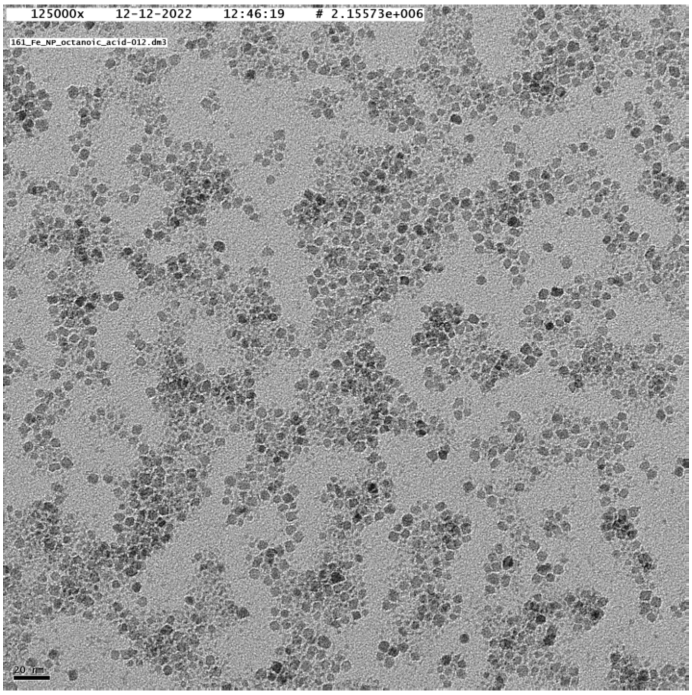

Figure S60: Transmission electron microscopy of FeO_x NP with octanoic acid (batch 161, octanoic acid 1:1, 1 mg mL^{-1} in DCM); particle size by DLS of 4.75±0.27 nm. Magnification of 125000x.

4.2.7 FeO_x NPs with nonanoic acid

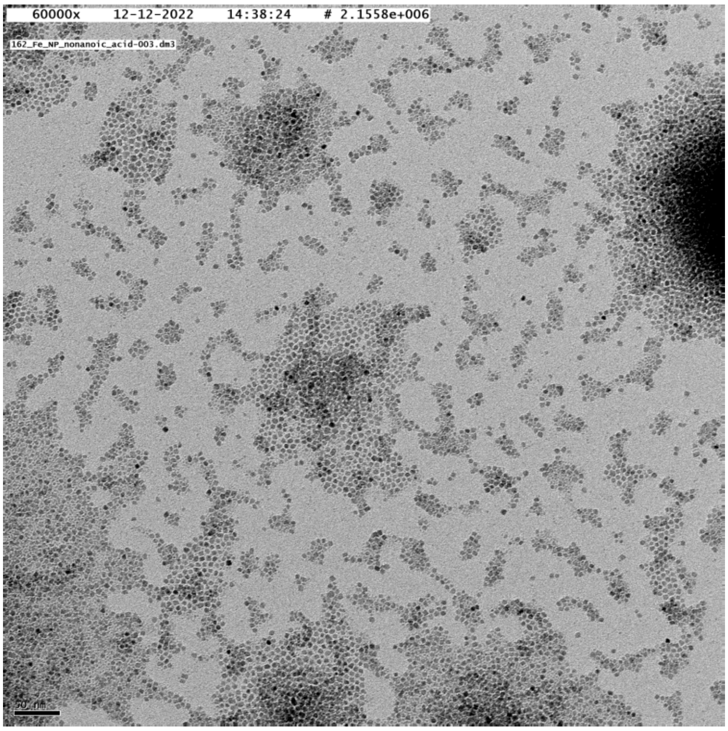

Figure S61: Transmission electron microscopy of FeO_x NP with nonanoic acid (batch 162, nonanoic acid 1:1, 1 mg mL^{-1} in DCM); particle size by DLS of 3.86±0.43 nm. Magnification of 60000x.

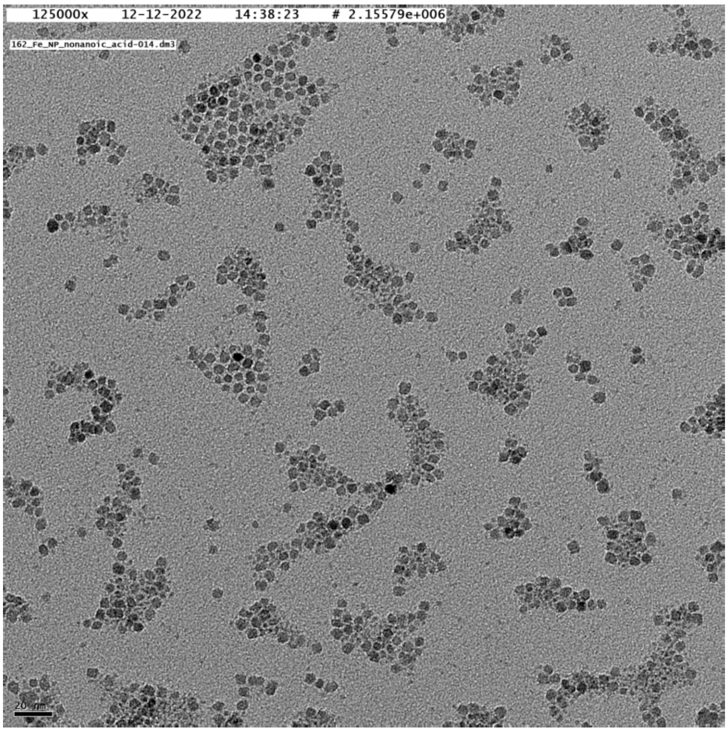

Figure S62: Transmission electron microscopy of FeO_x NP with nonanoic acid (batch 162, nonanoic acid 1:1, 1 mg mL^{-1} in DCM); particle size by DLS of 3.86±0.43 nm. Magnification of 125000x.

4.2.8 FeO_x NPs with lauric acid

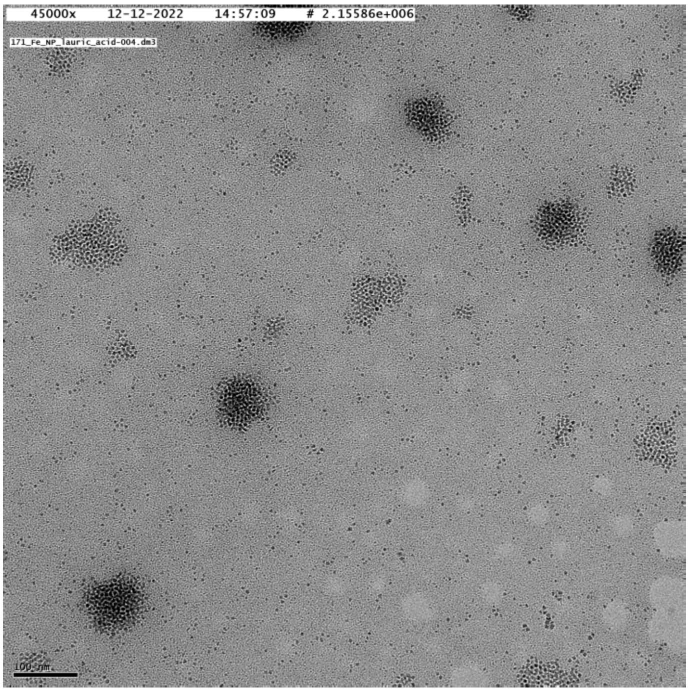

Figure S63: Transmission electron microscopy of FeO_x NP with lauric acid (batch 113, lauric acid 1.5:1, 1 mg mL^{-1} in DCM); particle size by DLS of 3.85±1.19 nm. Magnification of 45000x.

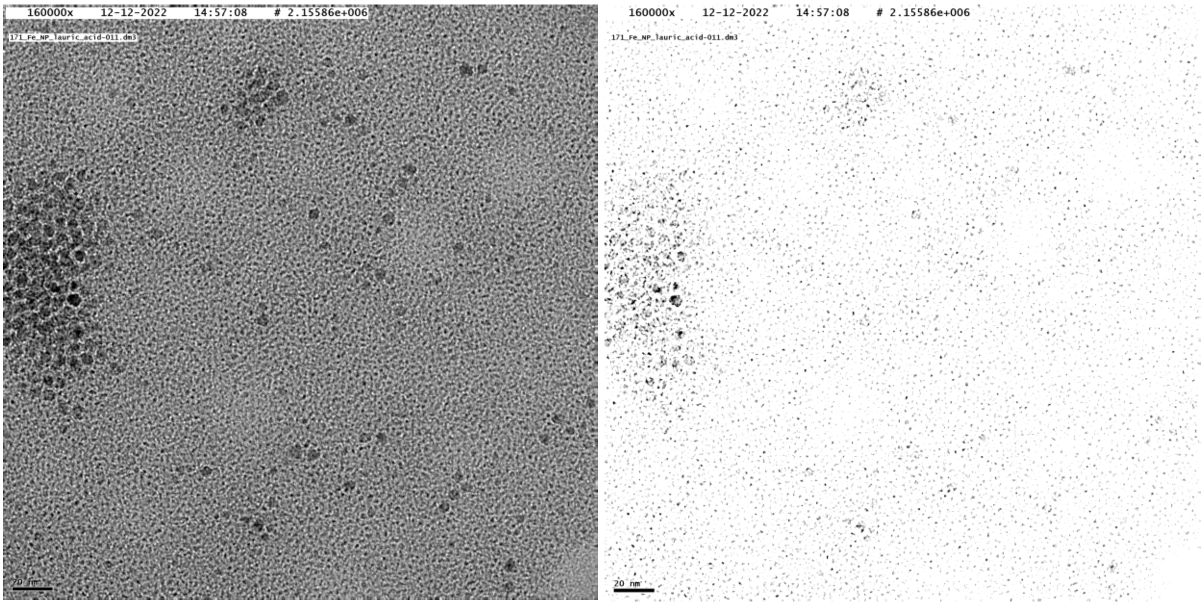

Figure S64: Transmission electron microscopy of FeO_x NP with lauric acid (batch 113, lauric acid 1.5:1, 1 mg mL^{-1} in DCM); particle size by DLS of 3.85±1.19 nm. Magnification of 160000x, higher contrast for better visibility (right).

4.2.9 FeOx NPs with elaidic acid (trans)

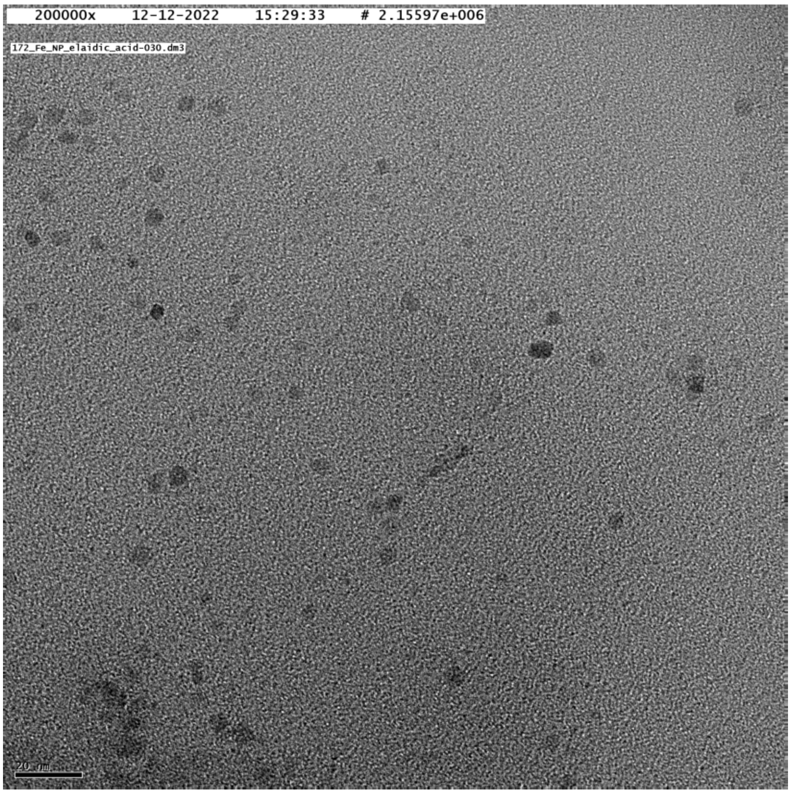

Figure S65: Transmission electron microscopy of FeO_x NP with elaidic acid (batch 172, elaidic acid 2:1, 1 mg mL^{-1} in DCM); particle size by DLS of 2.49±0.55 nm. Magnification of 200000x.

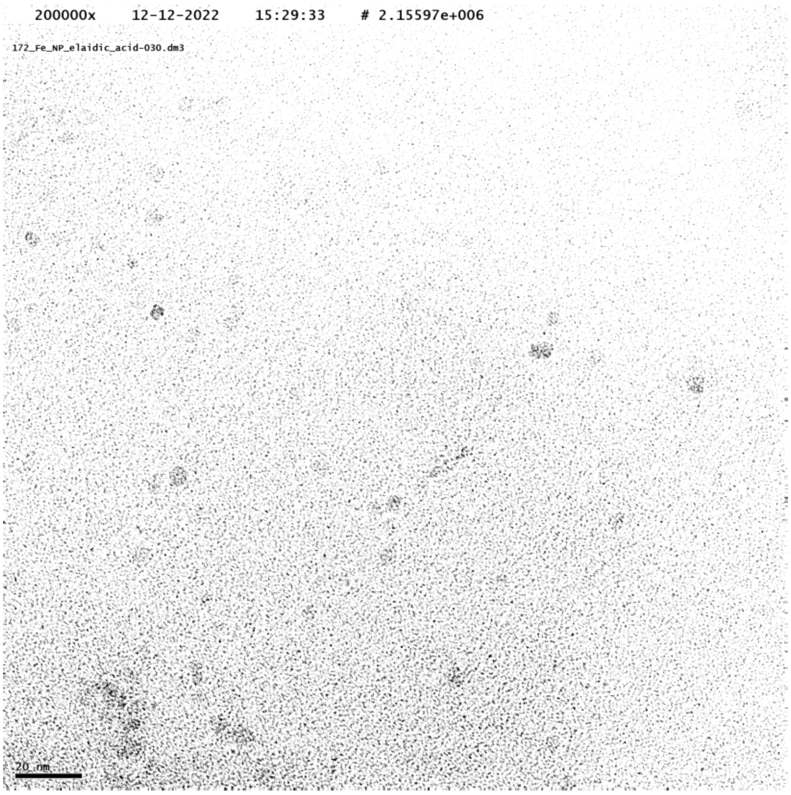

Figure S66: Transmission electron microscopy of FeO_x NP with elaidic acid (batch 172, elaidic acid 2:1, 1 mg mL^{-1} in DCM); particle size by DLS of 2.49±0.55 nm. Magnification of 200000x, higher contrast for better visibility.

4.2.10 FeO_x NPs with oleyl alcohol

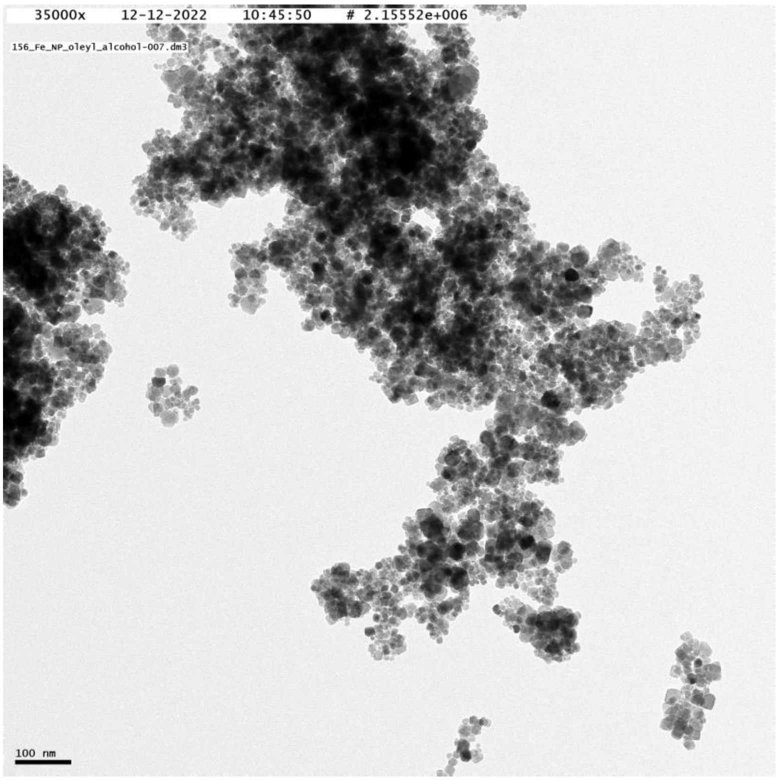

Figure S67: Transmission electron microscopy of FeO_x NP with oleyl alcohol (batch 156, oleyl alcohol 2:1, 1 mg mL^{-1} in DCM); particle size by DLS of 377±166 nm. Magnification of 35000x.

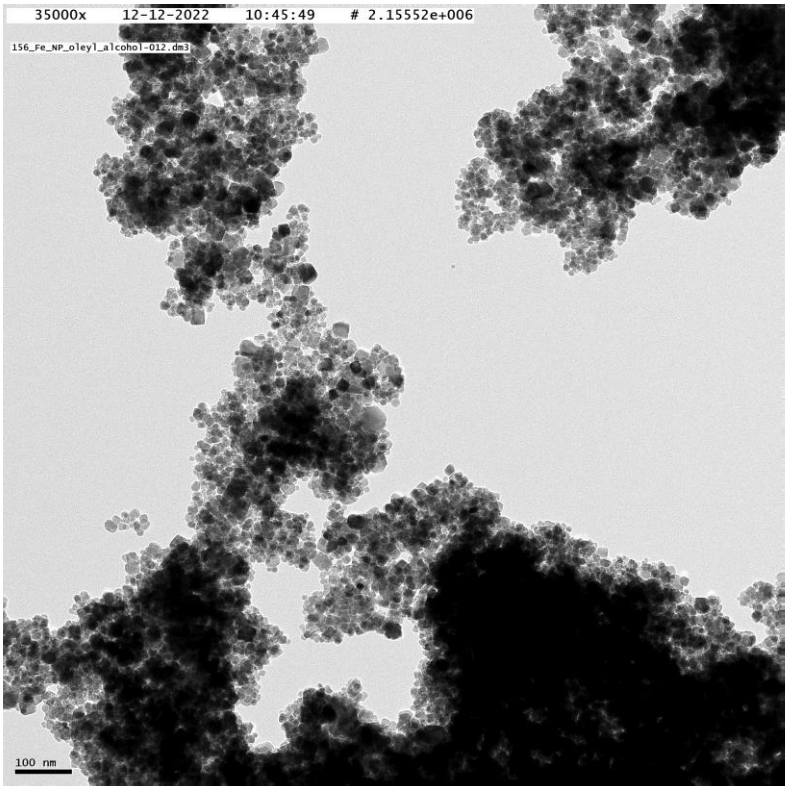

Figure S68: Transmission electron microscopy of FeO_x NP with oleyl alcohol (batch 156, oleyl alcohol 2:1, 1 mg mL^{-1} in DCM); particle size by DLS of 377±166 nm. Magnification of 35000x.

4.2.11 FeO_x NPs with oleylamine (1:1)

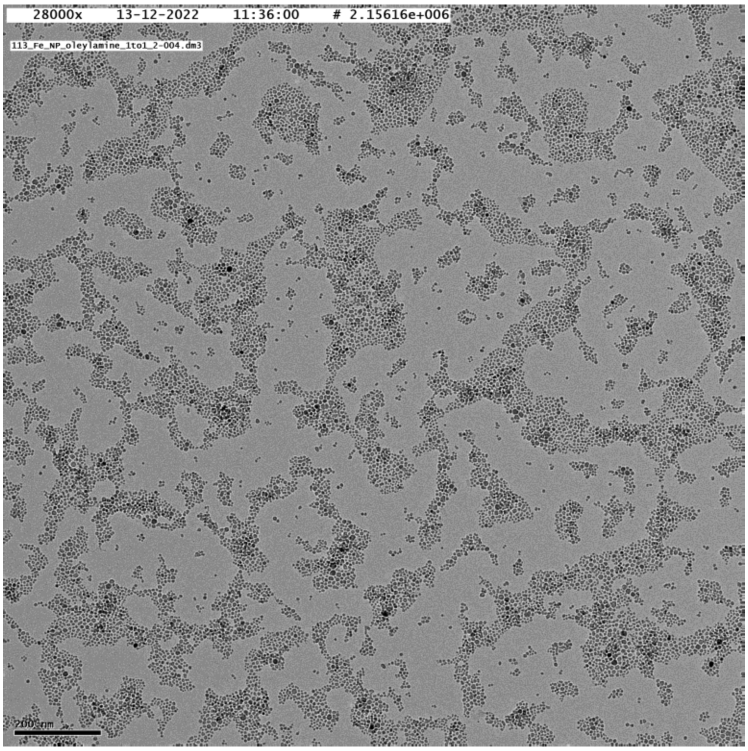

Figure S69: Transmission electron microscopy of FeO_x NP with oleylamine (batch 113, oleylamine 1:1, 1 mg mL^{-1} in DCM); particle size by DLS of 7.41±0.71 nm. Magnification of 28000x.

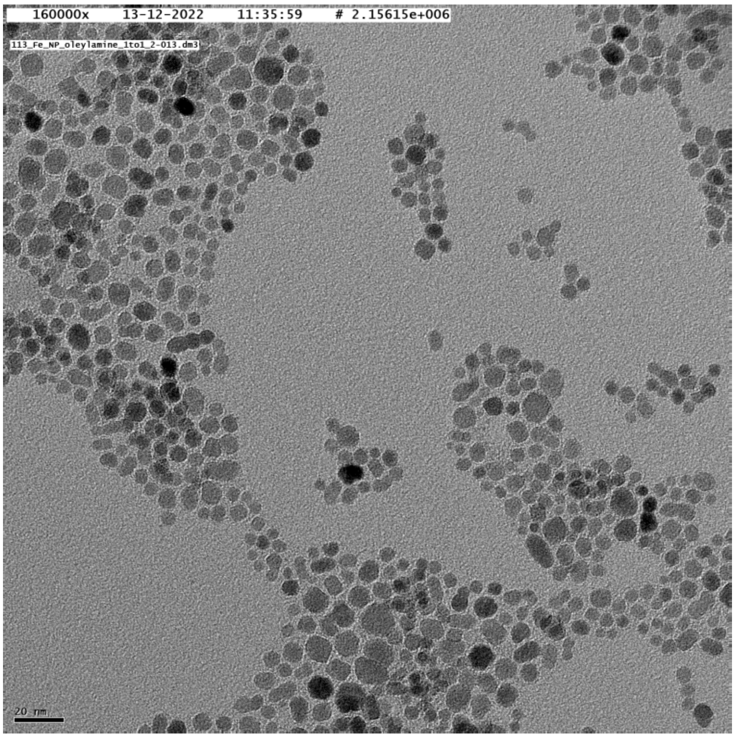

Figure S70: Transmission electron microscopy of FeO_x NP with oleylamine (batch 113, oleylamine 1:1, 1 mg mL^{-1} in DCM); particle size by DLS of 7.41±0.71 nm. Magnification of 160000x.

4.2.12 FeO_x NPs with oleylamine (2:1)

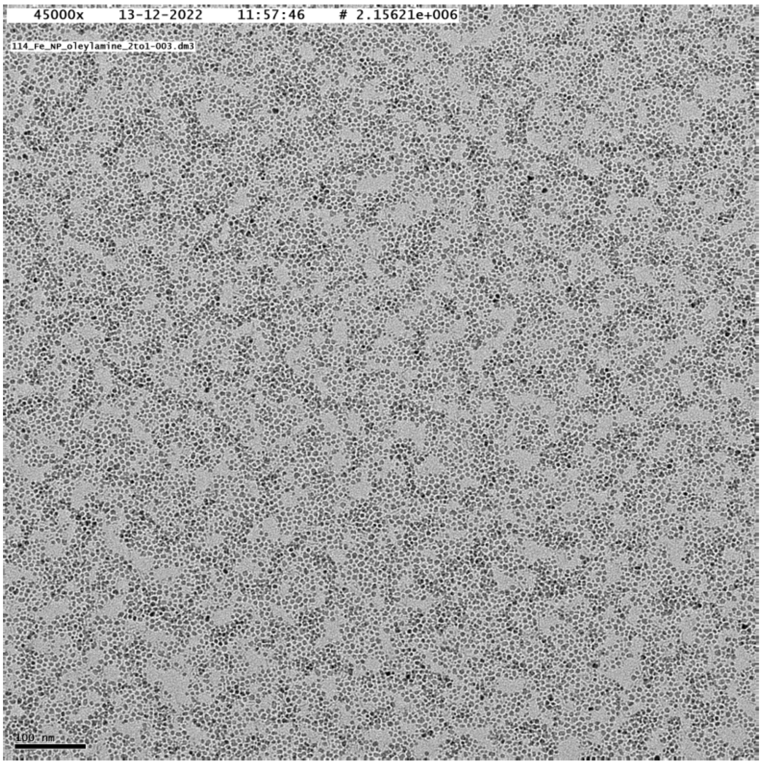

Figure S71: Transmission electron microscopy of FeO_x NP with oleylamine (batch 114, oleylamine 2:1, 1 mg mL^{-1} in DCM); particle size by DLS of 5.09±0.01 nm. Magnification of 45000x.

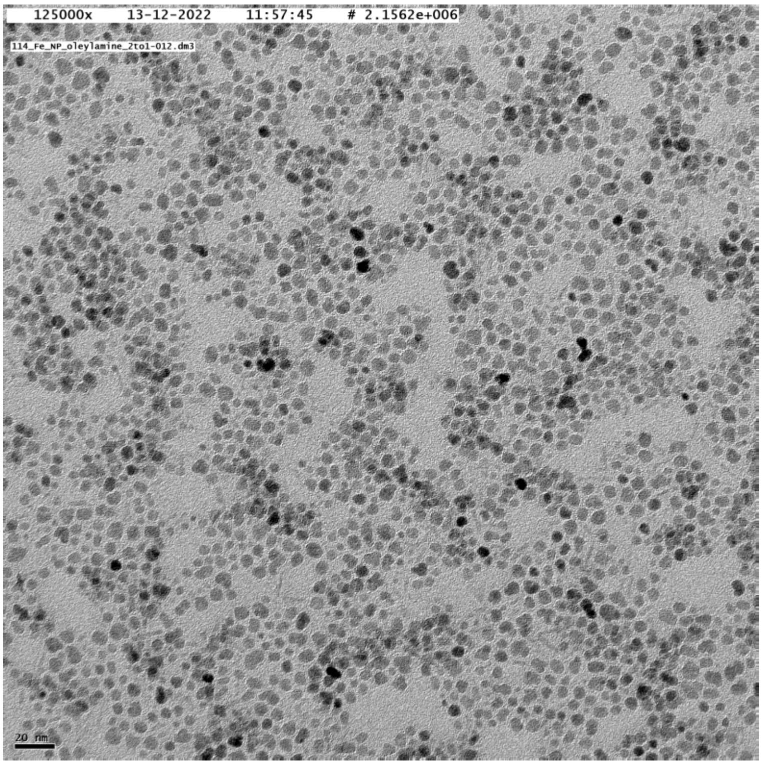

Figure S72: Transmission electron microscopy of FeO_x NP with oleylamine (batch 114, oleylamine 2:1, 1 mg mL^{-1} in DCM); particle size by DLS of 5.09±0.01 nm. Magnification of 125000x.

4.2.13 FeO_x NPs with oleylamine (3:1)

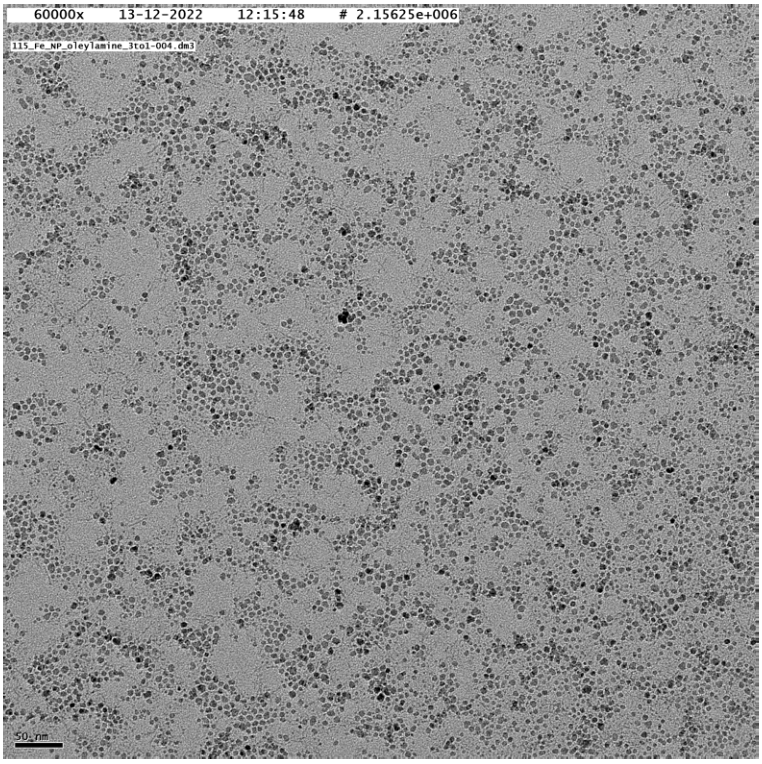

Figure S73: Transmission electron microscopy of FeO_x NP with oleylamine (batch 115, oleylamine 3:1, 1 mg mL^{-1} in DCM); particle size by DLS of 5.87±0.23 nm. Magnification of 60000x.

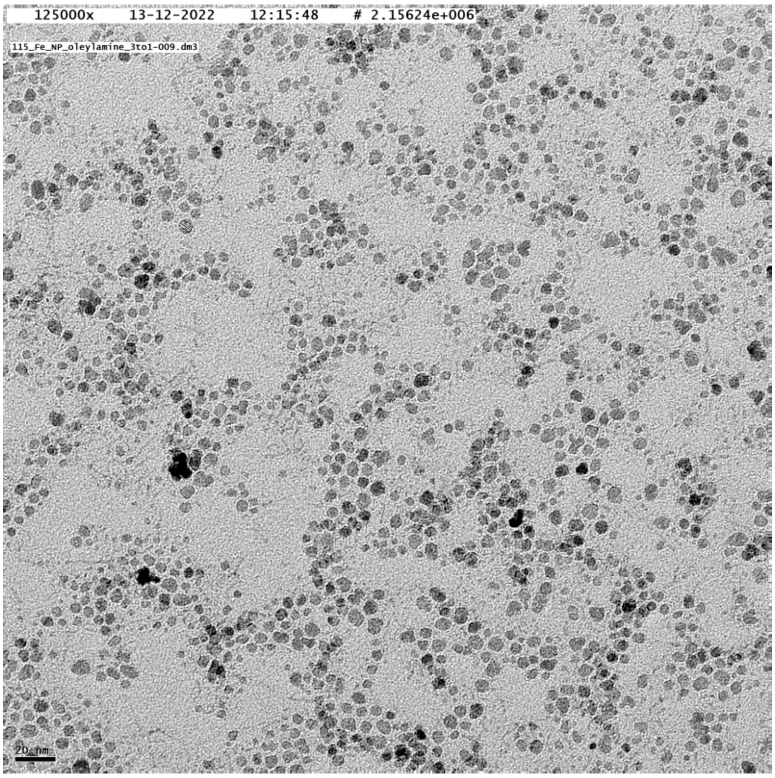

Figure S74: Transmission electron microscopy of FeO_x NP with oleylamine (batch 115, oleylamine 3:1, 1 mg mL^{-1} in DCM); particle size by DLS of 5.87±0.23 nm. Magnification of 125000x.

4.2.14 FeO_x NPs with N-oleyl sarcosine

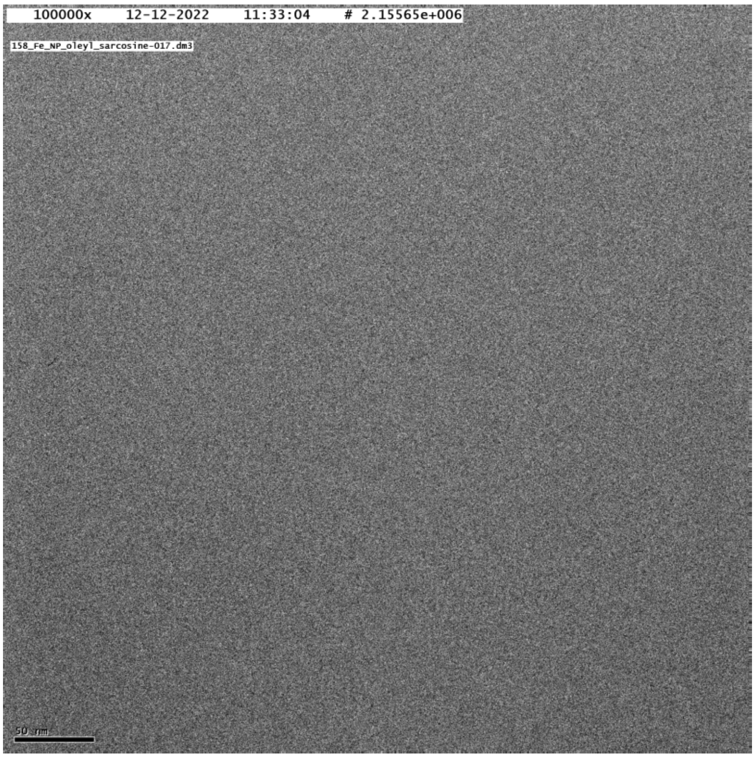

Figure S75: Transmission electron microscopy of FeO_x NP with N-oleyl sarcosine (batch 158, N-oleyl sarcosine 2:1, 1 mg mL^{-1} in DCM); particle size by DLS of 3.00±0.56 nm. Magnification of 100000x.

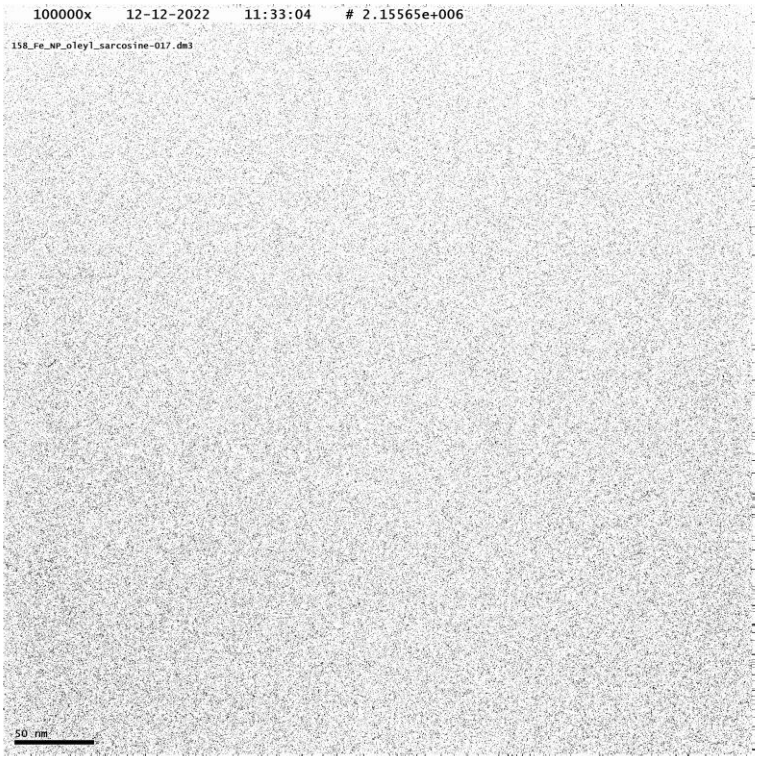

Figure S76: Transmission electron microscopy of FeO_x NP with N-oleyl sarcosine (batch 158, N-oleyl sarcosine 2:1, 1 mg mL^{-1} in DCM); particle size by DLS of 3.00±0.56 nm. Magnification of 100000x, higher contrast for better visibility.

4.3 XRD

XRD characterization: For analysis and comparison of the iron species present as FeO_x NPs an extensive powder X-ray diffraction study was conducted. XRD is a non-destructive analytical technique for evaluation of the crystal and atomic structure of the NPs. After dropcasting and drying the nanoparticles on the plate, an incident beam of X-rays with Cu Kα radiation (λ = 1.5418 Å) contacts the crystal/powder and the diffracted X-rays collected create a pattern which is characteristic of the crystal structure of the sample. A D8 Advance Bruker diffractometer with Cu Kα radiation (λ = 1.5418 Å) employing a 0.25° divergent slit and a 0.125° anti-scattering slit was utilized. The patterns were recorded in the 2θ range from 10° to 80° with a step of 0.017365 ° and a counting time of 10 s per step.

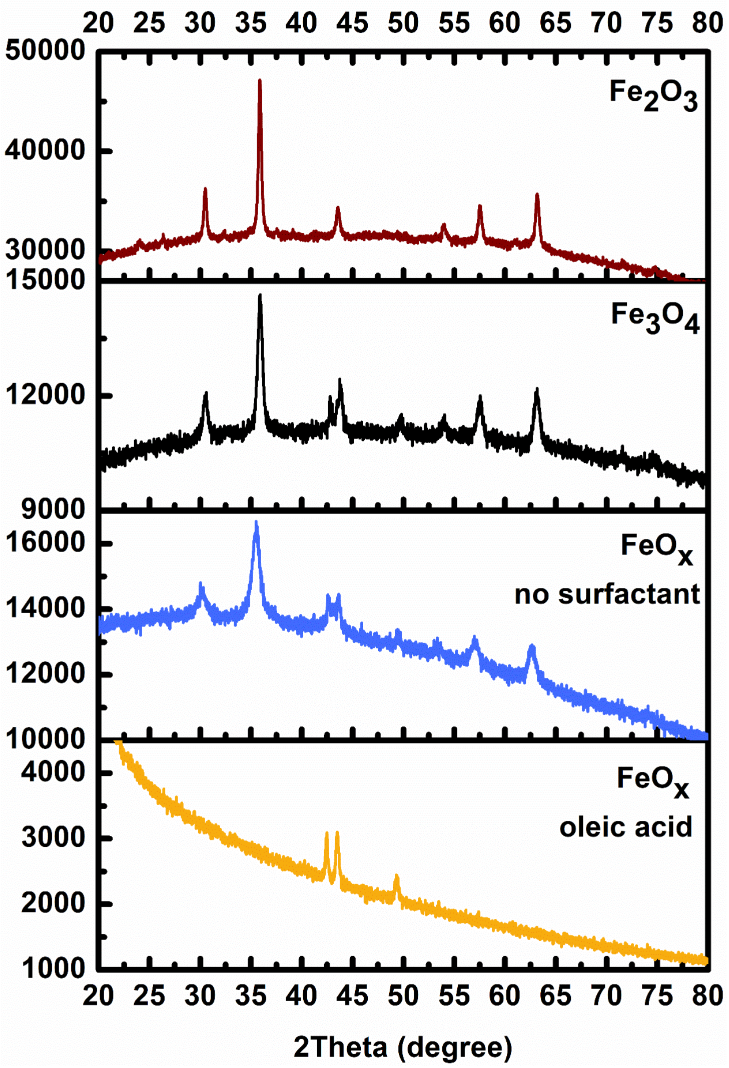

Figure S77: XRD comparison between FeO_x NPs with oleic acid (2:1, batch 135, Acros Organics) surfactant, without surfactant (batch 143, Acros Organics), Fe_2O_3 and Fe_3O_4. The FeO_x photocatalyst resembles Fe_3O_4 as indicated by the peaks in the area of 40-55 degrees; peak broadening is observed due to small size and/or amorphous properties of the FeO_x NP material.

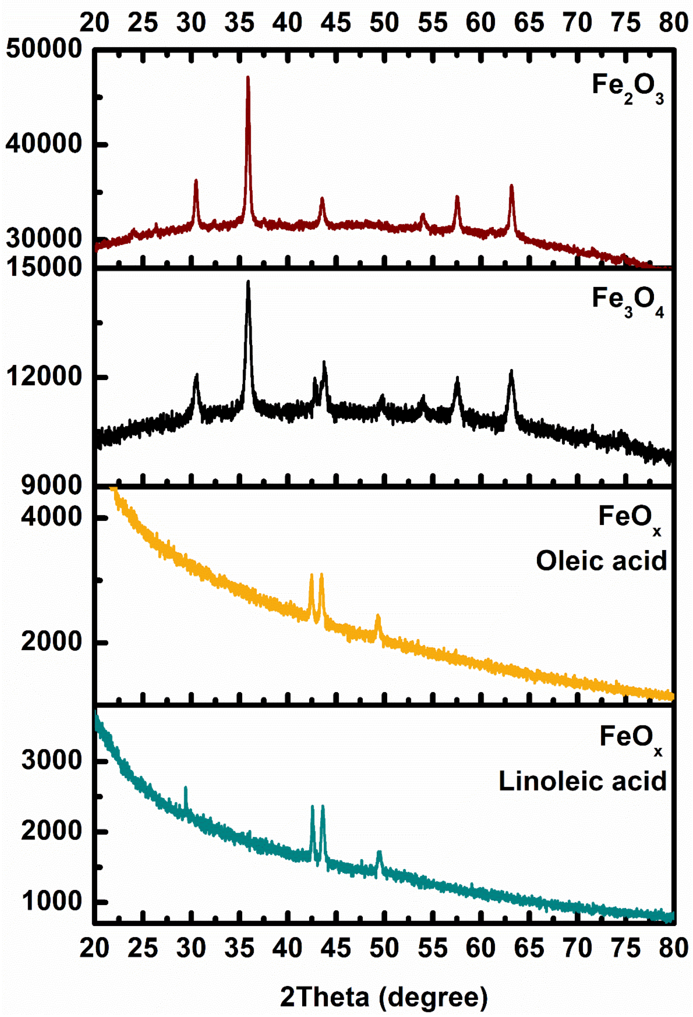

Figure S78: XRD comparison between FeO_x NPs with oleic acid (2:1, batch 135, Acros Organics, Ethanol), FeO_x NPs with linoleic acid (2:1, batch 144, Acros Organics, ethanol), Fe_2O_3 and Fe_3O_4. The FeO_x photocatalyst resembles Fe_3O_4 as indicated by the peaks in the area of 40-55 degrees. Peak broadening is observed due to small size and/or amorphous properties of the FeO_x NP material. Linoleic acid possesses the same properties (small size and/or amorphous Fe_3O_4-like material) as the standard oleic acid (FeO_x material).

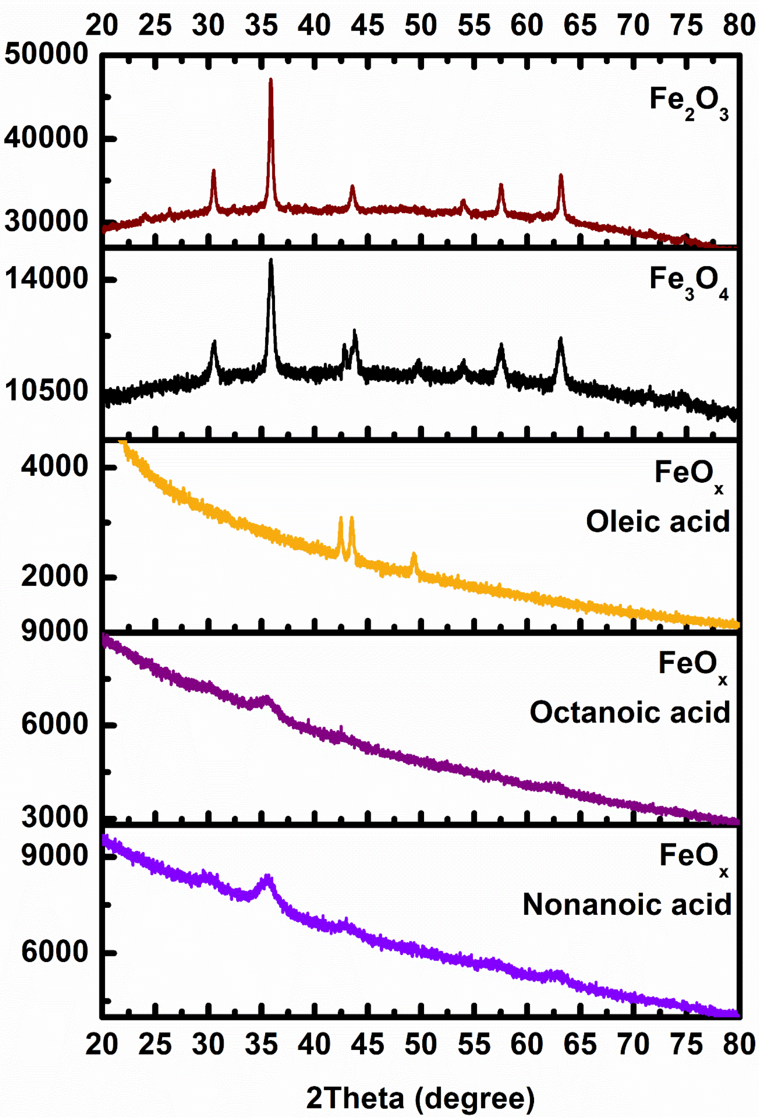

Figure S79: XRD comparison between FeO_x NPs with oleic acid (2:1, batch 135, Acros Organics, Ethanol), FeO_x NPs with octanoic acid (1:1, batch 121, Acros Organics, ethanol), FeO_x NPs with nonanoic acid (1:1, batch 120, Acros Organics, ethanol), Fe_2O_3 and Fe_3O_4. The FeO_x photocatalyst resembles Fe_3O_4 as indicated by the peaks in the area of 40-55 degrees. Peak broadening is observed due to small size and/or amorphous properties of the FeO_x NP material.

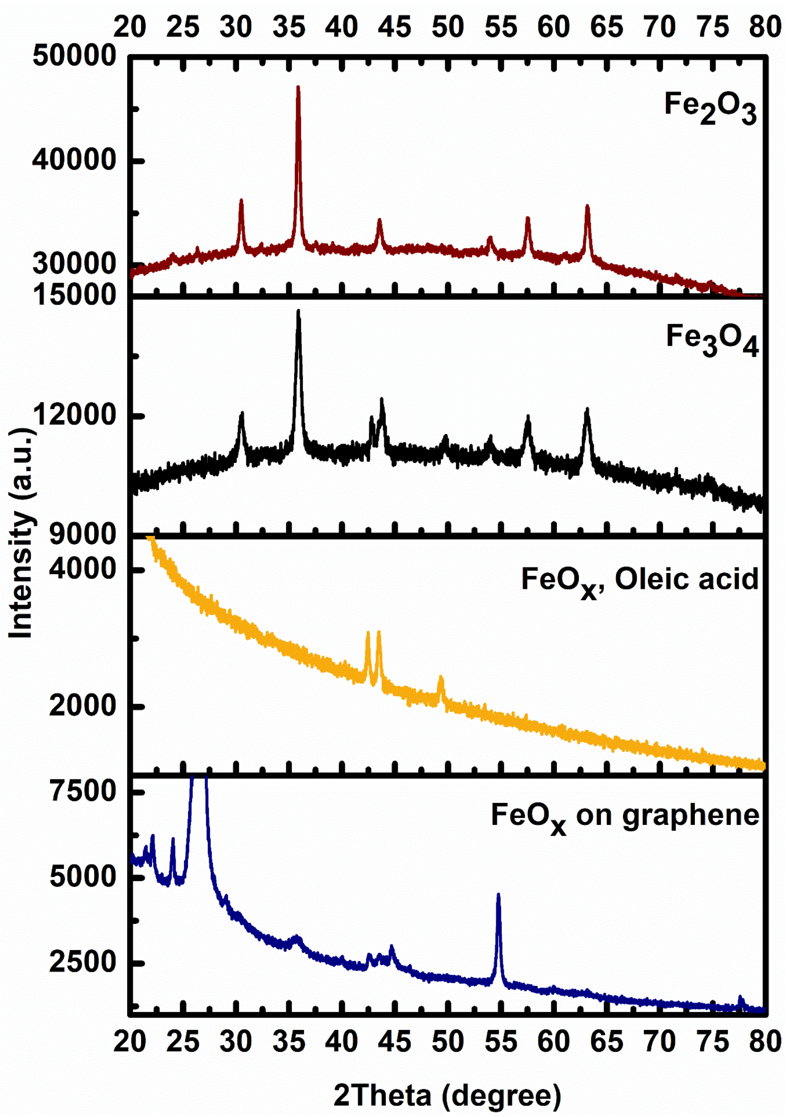

Figure S80: XRD comparison between FeO_x NPs with oleic acid (2:1, batch 135, Acros Organics, Ethanol), FeO_x NPs with octanoic acid (2:1, batch 160, Acros Organics, ethanol) immobilized on graphene nanoplatelets, Fe_2O_3 and Fe_3O_4. Also immobilized FeO_x photocatalyst on graphene nanoplatelets resembles Fe_3O_4 as indicated by the peaks in the area of 40-55 degrees. In addition to the carbon peaks the immobilization of the iron on the graphene surface can be confirmed *via* the distinct peaks.

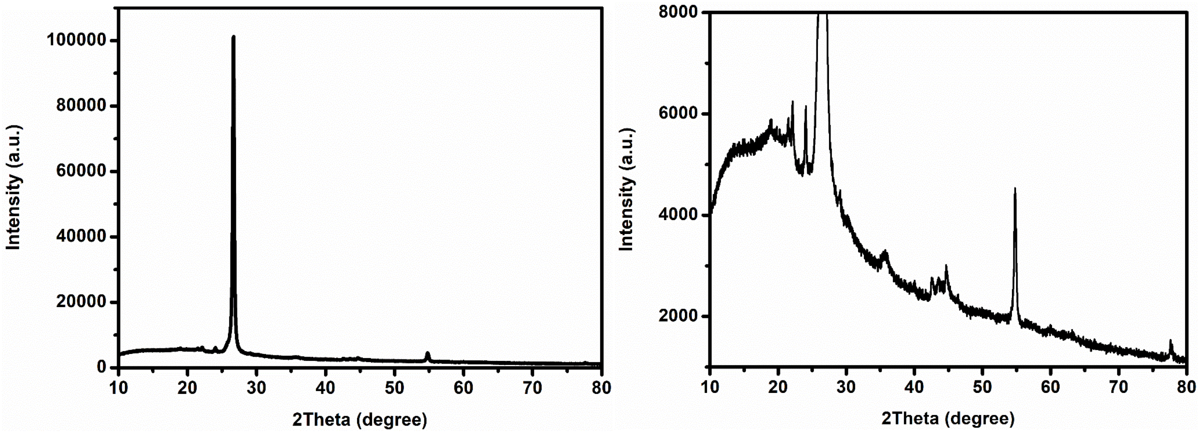

Figure S81: Powder XRD of FeO_x NPs with oleic acid immobilized on graphene (2:1, batch 160, Acros Organics, synthesized in ethanol). Top: original spectrum, bottom: zoom.

4.4 Elemental analysis

Table S2: Elemental analysis (C, H, Fe and calculated O value) of FeO_x nanoparticle catalyst with oleic acid as surfactant.

	w/w%	M (g/mol)	n (mol ratio)	Sum formula	Fe_3O_4/FeO species	Ratio
**Fe**	11.55	55.94	0.21	1.03	4	1
**O**	17.99	15.99	1.13	5.63	20	5
**C**	61.14	12	5.10	25.48	100	25
**H**	9.32	1	9.32	46.60	200	50
**Sum**	**100**			$FeO_5C_{25}H_{46}$	$(C_2H_5OH)_5/(C_{18}H_{34}O_2)_5@Fe_3O_4/FeO$	

The value for oxygen has been calculated by subtracting C, H and Fe from 100%. The final estimated species $(C_2H_5OH)_5/(C_{18}H_{34}O_2)_5@Fe_3O_4/FeO$ was calculated to fit the molar ratios of the original sum formula and serves as indication of the nanoparticle species.

4.5 UV-Vis

4.5.1 FeO_x scope comparison

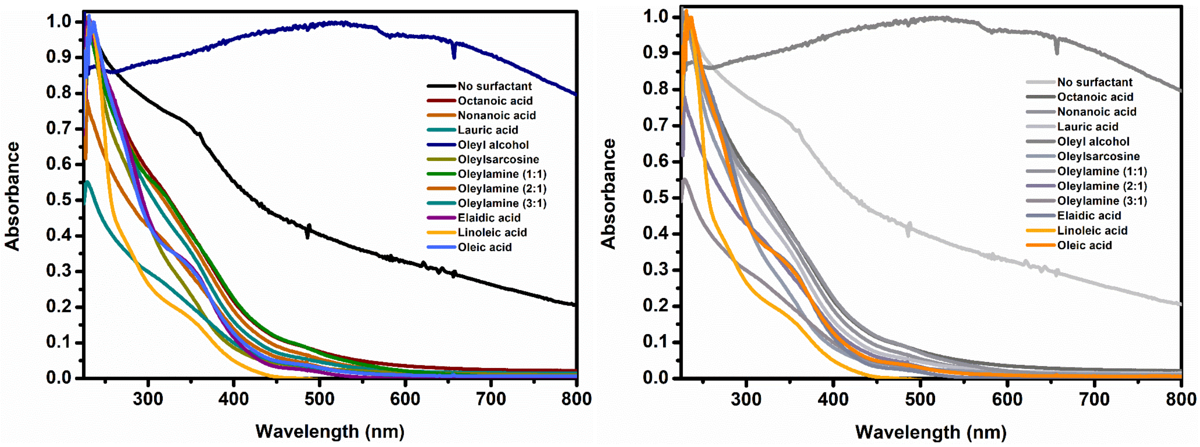

Figure S82: UV-Vis absorption spectra of the whole scope of FeO_x NPs normalized (left). Comparison of absorption spectra for the whole scope of the synthesized FeO_x NPs. Active catalyst materials (oleic acid and linoleic acid) have been depicted in orange/yellow, while others are depicted in greyscale (right).

4.5.2 Comparison FeO_x active materials: oleic acid (*cis*), linoleic acid (*cis*,*cis*) with inactive elaidic acid (*trans*)

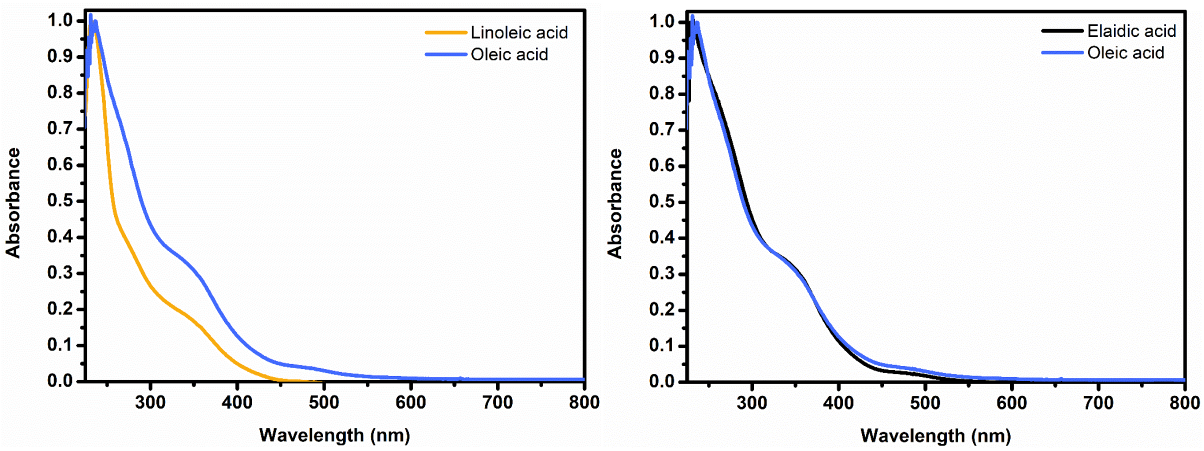

Figure S83: Comparison of absorption spectra for FeO_x species possessing photochemical activity for oxygen reduction towards H_2O_2. No significant difference in the electronic states/absorption between the surfactants is obtained (left). Comparison of absorption spectra between FeO_x NP with elaidic acid and oleic acid. No significant difference in the electronic states/absorption between the surfactants is obtained, which is remarkable as oleic acid (*cis*) promotes photochemical oxygen reduction and elaidic acid (*trans*) is not (right).

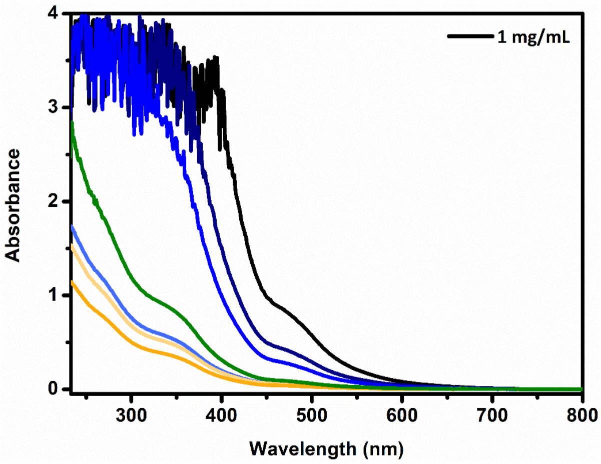

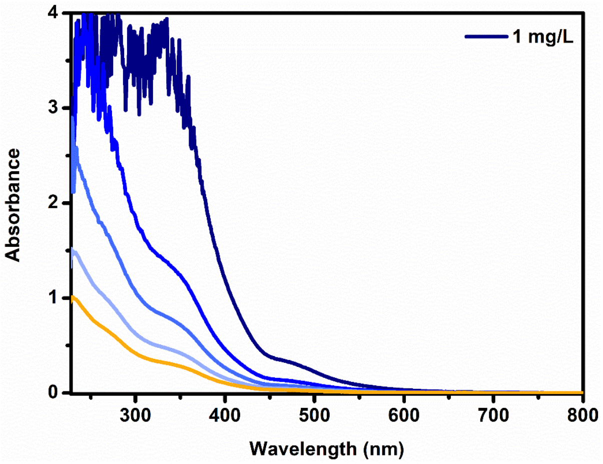

Figure S84: UV-Vis spectrum of FeO_x NPs with oleic acid (2:1, Acros Organics, batch 188) in DCM, dilutions from 1 mg mL^{-1} onwards (left). UV-Vis spectrum of FeO_x NPs with oleic acid (2:1, Sigma Aldrich, batch 189) in DCM, dilutions from 1 mg mL^{-1} onwards (right).

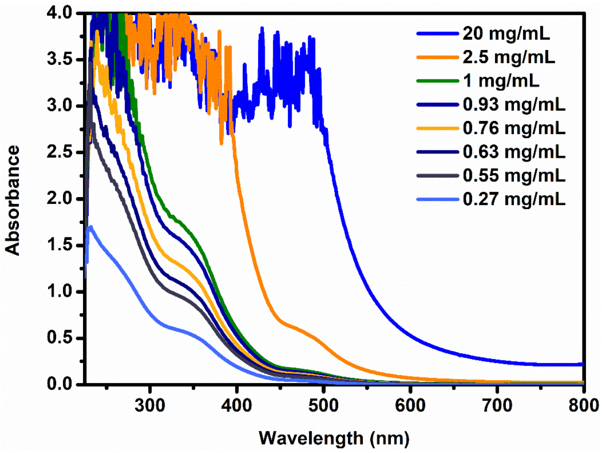

Figure S85: UV-Vis spectrum FeO_x NPs with elaidic acid (2:1, batch 172) in DCM.

4.5.3 FeO_x NP scope

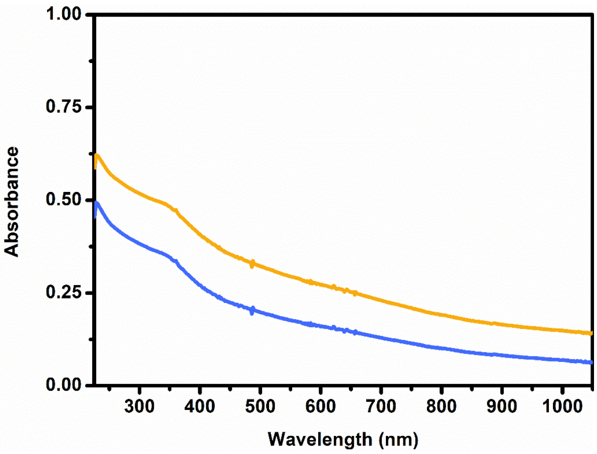

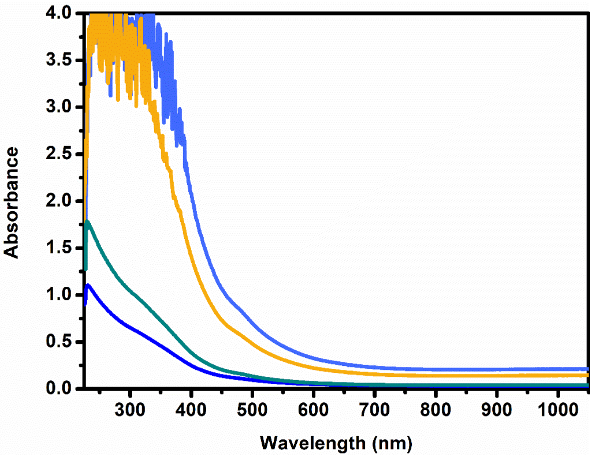

Figure S86: UV-Vis spectrum FeO_x NPs without surfactant (batch 143) in DCM (left). UV-Vis spectrum FeO_x NPs with octanoic acid (1:1, batch 161) in DCM (right)

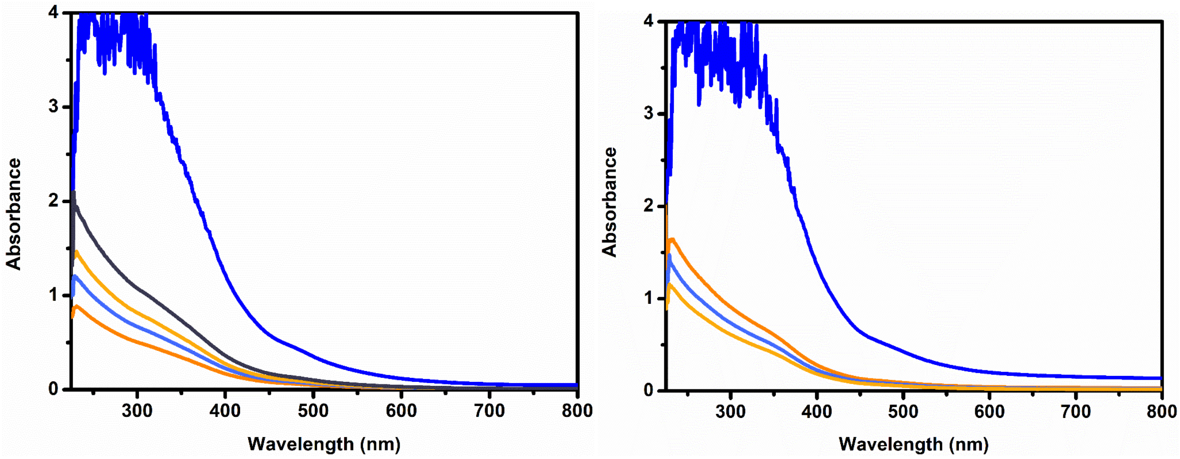

Figure S87: UV-Vis spectrum FeO_x NPs with nonanoic acid (1:1, batch 162) in DCM (left). UV-Vis spectrum FeO_x NPs with lauric acid (1.5:1, batch 171) in DCM (right).

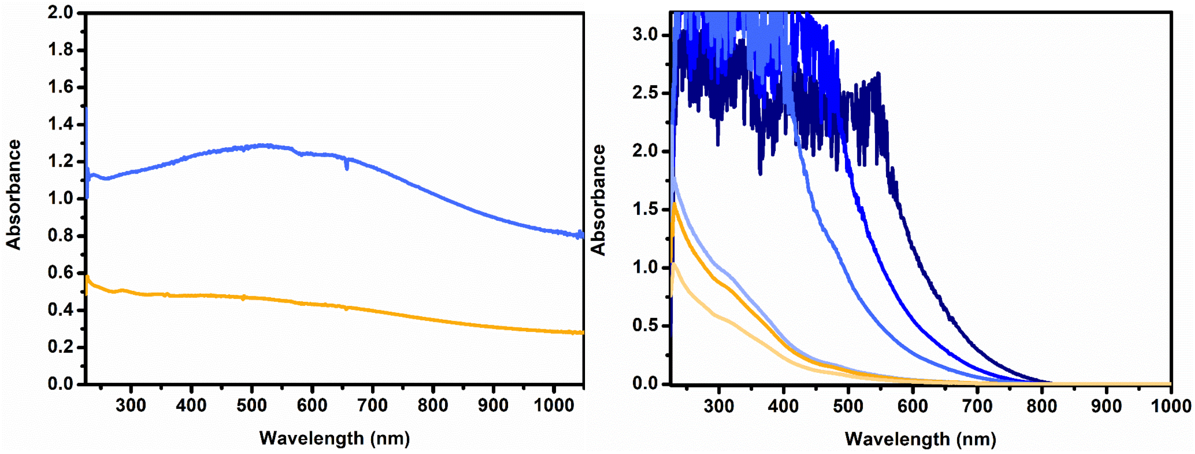

Figure S88: UV-Vis spectrum FeO_x NPs with oleyl alcohol (2:1, batch 156) in DCM (left). UV-Vis spectrum of FeO_x NPs with oleylamine (1:1, batch 113), dilutions from 1 mg mL^{-1} onwards in DCM (right).

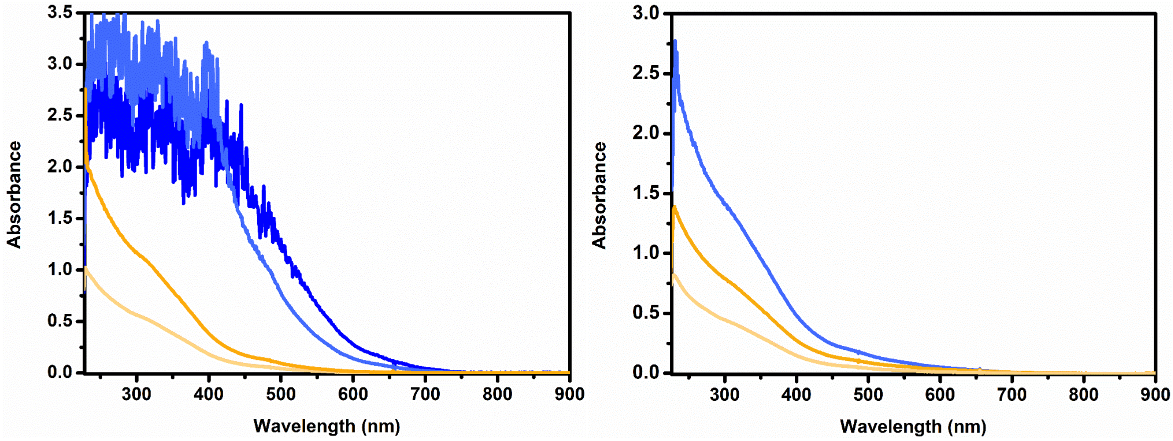

Figure S89: UV-Vis spectrum of FeO_x NPs with oleylamine (2:1, batch 114), dilutions from 1 mg mL^{-1} onwards in DCM (left). UV-Vis spectrum of FeO_x NPs with oleylamine (3:1, batch 115), dilutions from 1 mg mL^{-1} onwards in DCM (right).

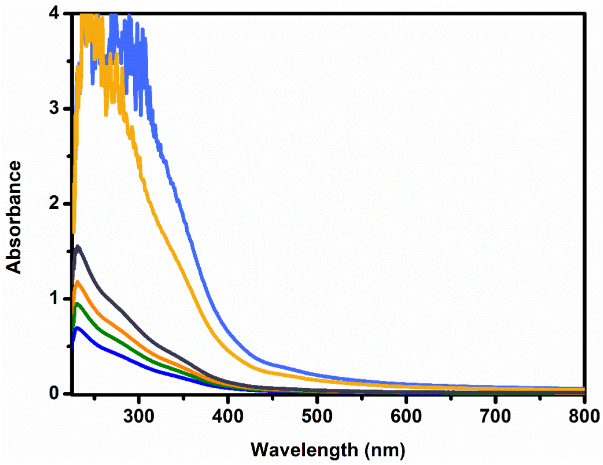

Figure S90: UV-Vis spectrum FeO_x NPs with N-oleylsarcosine (2:1, batch 158) in DCM.

4.6 Tauc plot

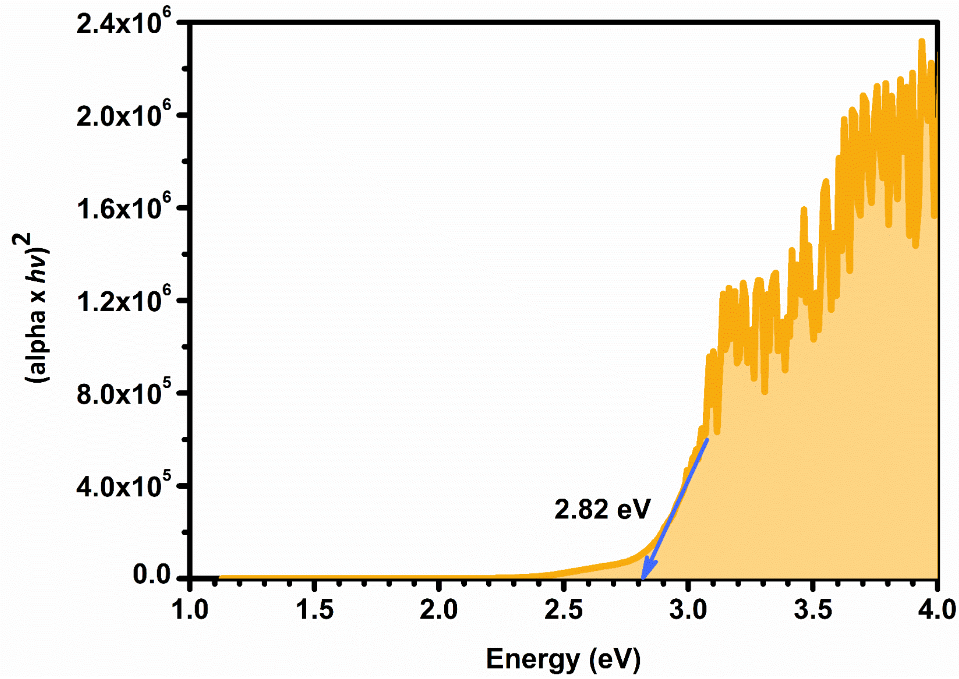

Figure S91: UV-Vis Tauc plot indicating the optical bandgap of the FeO_x catalyst, where h = Planck's constant, *v* = frequency of the radiation and α = absorption coefficient. The Tauc plots of different batches, solvents (THF and DCM) and heterogeneous deposition on the side of the cuvettes (AQY measurements) where compared. Conditions: batch 188 of FeO_x NP with oleic acid measured in DCM (c = 1 mg mL^{-1}).

4.7 Electrochemical Mott-Schottky measurements

The as-received fluorine-doped tin oxide glass slides (FTO, Sigma Aldrich, 30×30 cm, 2.3 mm thickness, 13 Ω/sq) were cut into smaller pieces of 2.5×5 cm. The conductive side was scrubbed with Deconex Forte 24 and warm tap water, followed by a thorough rinse with ethanol (absolute grade). After wiping the FTO surface with an ethanol-soaked medical wipe, the plate was left to air dry, after which it was cut into pieces of 1×2.5 cm. An area of 1 cm^2 was masked with Scotch® Magic Tape.

The FeO_x@FTO electrodes were prepared according to an adjusted literature procedure.[14] 324 µL of FeO_x solution (in DCM, 15.4 mg/mL) was added to isopropanol (750 µL, HPLC grade) and DCM was evaporated at reduced pressure at 20 °C, resulting in an orange-brown suspension. While sonicating, Milli-Q (18.2 MΩ cm, 250 µL) and Nafion (D-521 dispersion, 5% w/w in water and 1-propanol, ≥ 0.92 meq/g exchange capacity, Alfa Aesar) were added, and the mixture was sonicated for another ten minutes. The resulting suspension was drop-casted immediately after sonication on the exposed part of the FTO plate with a Gilson micropipette (15 µL per cm^2 FTO). The electrodes were left to airdry, after which the tape was removed, resulting in the FeO_x@FTO electrode depicted in **Figure S92 A**.

The Mott-Schottky plots were measured on an electrochemical workstation (Zahner Zennium & PP211, Germany) in a standard three-electrode system, with the FeO_x@FTO as working electrode (WE), a Pt wire (d = 0.5 mm) as counter electrode (CE), and a Ag/AgCl reference electrode (RE, in 3 M KCl). The three electrodes were assembled in a heart-shaped cell, as depicted in **Figure S92 B**, and placed in a light exclusion box (Zahner Elektrik, Germany). The Mott-Schottky measurements were conducted in the dark, at an AC amplitude of 10 mV, at 500, 700, 1000, 1500 and 2000 Hz frequencies. The step size of the voltage sweep was set at 10 mV, with a 5 s settling time for each data point.

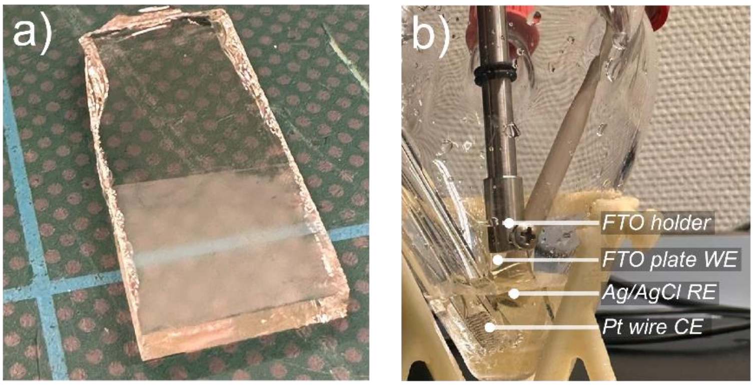

Figure S92: a) FeO_x@FTO electrode; b) setup for electrochemical measurements, consisting of a heart-shaped cell, containing FeO_x@FTO plate WE (in a stainless-steel holder), the Pt wire CE and Ag/AgCl RE.

The flat band potential (E_{fb}) of FeO_x was determined to be –0.074 V (**Figure S93**) *versus* the reversible hydrogen electrode (RHE), according to the equation $E_{RHE} = E_{Ag/AgCl} + 0.210 + (0.0591 \times pH)$.[14] Therefore, the E_{CB} of FeO_x lies at –0.074 V vs. RHE. Since the valence band position can be derived from the equation $E_{vb} = E_{cb} + E_g$, and the optical gap (E_g) was determined from the UV-vis spectrum with a value of 2.82 eV (**Figure S91**), the valence band lies at 2.75 V vs. RHE.

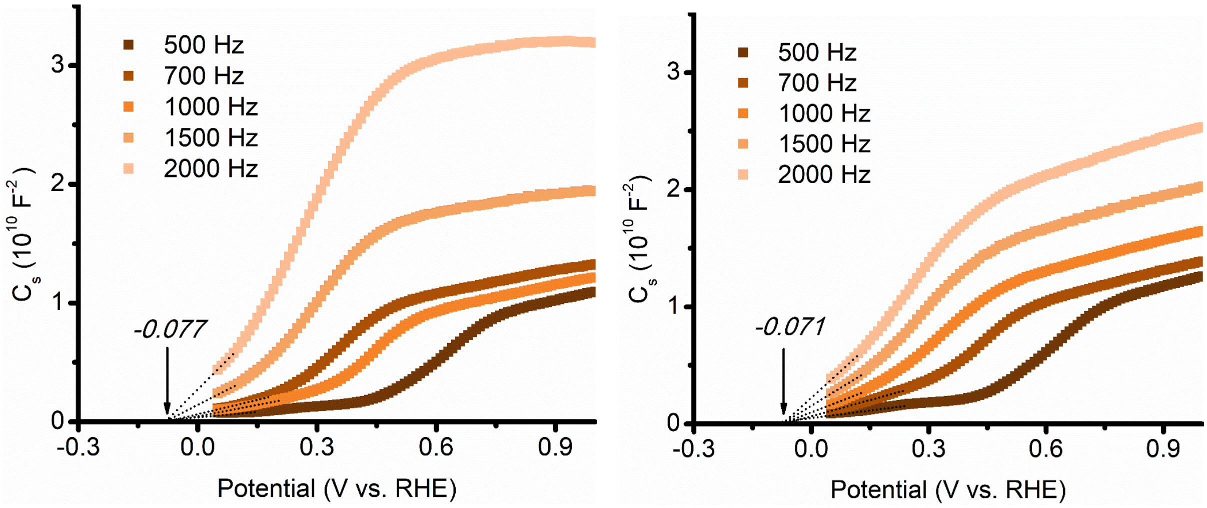

Figure S93: Electrochemical Mott-Schottky plots (duplo) of the FeO_x@FTO electrodes at varying frequencies, revealing their flat-band potential (as indicated by the arrow).

5. Irradiation studies

5.1 Batch photocatalytic oxygen reduction

Photocatalytic oxygen reduction to hydrogen peroxide (H_2O_2) *via* FeO_x NPs was carried out in 10 mL vials, in a block of 6 slots, with irradiation of 500 mW LED from the bottom (**Figure S94**). To allow for an oxygen atmosphere and saturation of the solution, oxygen was bubbled (30 min solvent + 5 min headspace) using a needle. The temperature of the LEDs was controlled at 20°C by a liquid circulator. The photooxygenation was carried out according to the **Experimental procedure.**

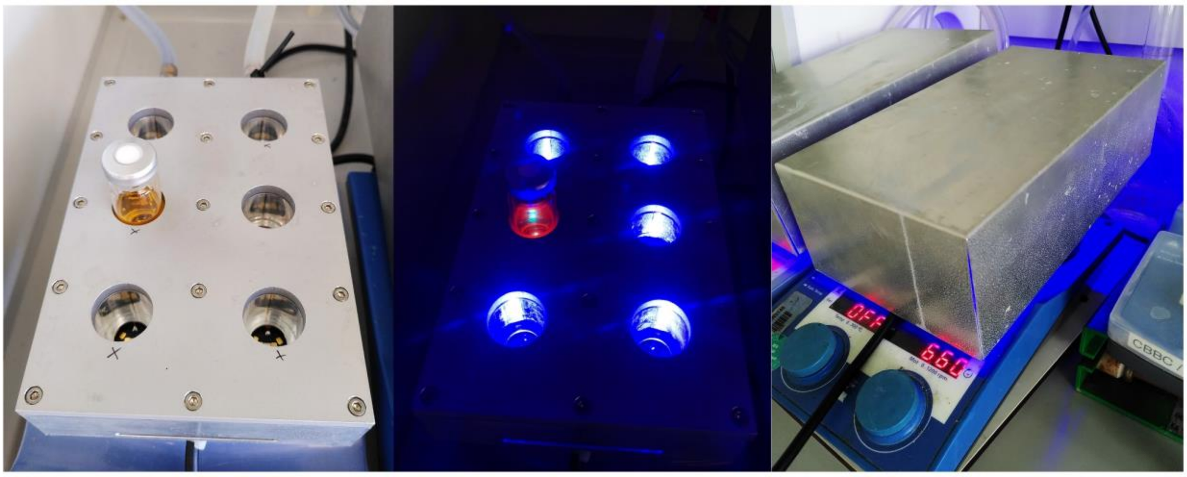

Figure S94: Batch irradiation setup for high-throughput screening.[15]

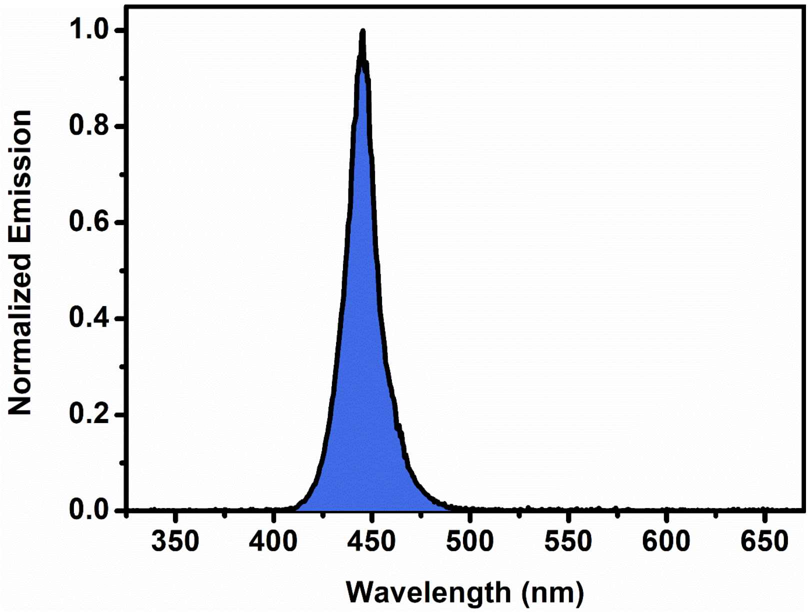

Figure S95: Normalized emission spectrum LED OSRAM Oslon SSL 80 royal blue (500 mW, λ = 445 nm, 180 mW/cm^2) as light source for batch photo-reactions.

5.2 Rotary photoreactor (PhotoVap)

The previously described rotary evaporator allows for efficient larger scale photooxygenation due the fast rotation creating a thin film, optimal for light penetration, and creating a high mass transfer of oxygen into the solution. To allow for an oxygen atmosphere in the rotary photoreactor, the photoreactor was purged with O_2 through vacuum/oxygen cycles using an O_2 filled balloon that was attached to the rotary evaporator. A home-built white light setup (575 lm, 8 W each; 5750 lm, 80 W total) consisting of standard white light LED bulbs was placed at a distance of approximately 5 cm around the 1 L flask, containing the reaction mixture (**Figure S96**). The photocatalytic transformations were carried out according to the **Experimental procedures**. The percentage of hydrogen peroxide produced by the described FeO_x photocatalysts was analyzed by performing a 5 h irradiation experiment (white light) on the rotavap as shown in **Figure S96**. Here the inside of a round bottom flask was covered/coated by a thin film of iron oxide nanoparticles with oleic acid (2:1) surfactant (batch 129). Oxygenated water (30 mL, 30 minutes oxygen bubbling) was added and an oxygen balloon attached ensuring constant oxygen atmosphere. Successful photochemical production of H_2O_2 was achieved.

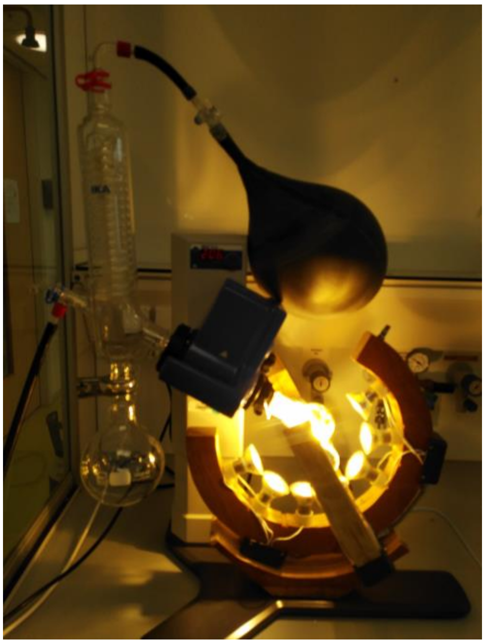

Figure S96: Setup used for hydrogen peroxide production *via* ORR for LC-MS experiment.[16]

5.3 Real sunlight

A long-term irradiation experiment in sunlight was conducted for 1 week by placing a sample in a window, where it had approximately 8h of sunlight daily (performed in September 2022 facing south in Groningen, NL). Conducting the same LC-MS experiment on this sample showed 92% selectivity towards hydrogen peroxide, where 7% could be assigned to the hydroperoxide. This increased selectivity towards the hydroperoxide could be caused by the longer irradiation time or UV content of sunlight, decomposing the catalyst.

5.4 Screening and batch irradiation of NPs synthesized in dibutyl ether

Iron oxide nanoparticles dissolved in DCM were added to a 10 mL vial to obtain 4 mg after evaporation of DCM. To the dried nanoparticles Milli-Q water (4 mL) was added to obtain a catalyst loading of 1 mg mL^{-1}. A Teflon stirring bar was added after which the vial was closed by capping. The sample was then extensively vortexed and sonicated for 5 min for better dispersion of the nanoparticles. Finally, 5 min of oxygen bubbling through the solution was conducted to ensure an oxygen atmosphere in the vial. Irradiation studies were conducted for 2.5 h at 20°C by 445 nm irradiation in triplicate. The photo-reactions were carried out in a block of 6 slots, with irradiation of 500 mW LED (180 mW/cm^2) from the bottom. Blanks in darkness were also performed as triplicate measurements for comparison.

5.5 Screening and batch irradiation of NPs synthesized in ethanol

Iron oxide nanoparticles dissolved in DCM were added to a 10 mL vial to obtain 4 mg after evaporation of DCM. Pre-oxygenated (30 minutes) Milli-Q water (4 mL) was added to the dried nanoparticles to obtain a catalyst loading of 1 mg mL^{-1}. A Teflon stirring bar was added after which the vial was closed by capping. The sample was then extensively vortexed and sonicated for 5 min for better dispersion of the nanoparticles. Finally, 5 min of oxygen bubbling through the solution was conducted to ensure an oxygen atmosphere in the vial. Irradiation studies were conducted for 5h at 20°C by 445 nm irradiation in triplicate. The photo-reactions were carried out in a block of 6 slots, with irradiation of 500 mW LED (180 mW/cm^2) from the bottom. Blanks in darkness were also performed as triplicate measurements for comparison.

Catalyst recycling was performed by drying the catalyst after each irradiation reaction. The dried catalyst (~4mg) was then redissolved in DCM (0.5 mL), and stored in nitrogen atmosphere and darkness at 5°C. The method as described above could then be followed after evaporation of DCM. These catalyst recycling reactions were conducted until catalyst activity was depleted.

The abovementioned procedure describes how a standard screening reaction was prepared and conducted. **Table S3** describes different catalyst screening conditions and how they varied from the standard method described above.

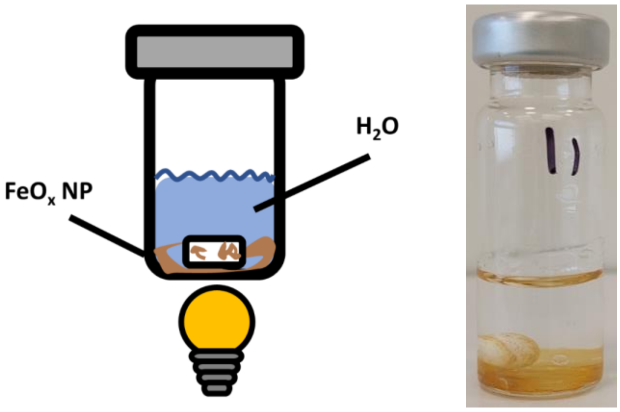

Figure S97: Left) Schematic representation of a prepared sample for irradiation studies. Right) FeO_x NPs with oleic acid (2:1) surfactant sticking to glass (batch 126).

Table S3: Different catalyst screening reactions performed and their deviation from the standard method. When suspensions and dissolved particles were obtained after 5h screening, then a filtration through a 0.2 µm syringe filter was conducted.

Screening	Amounts added	Comment
Kinetics	-	Irradiation time varied
Atmosphere (Air)	-	No pre-oxygenation of Milli-Q water and no oxygen bubbling
Atmosphere (nitrogen)	-	30 min of Milli-Q water pre-nitrogenation and 5 min of nitrogen bubbling
Atmosphere (open air)	-	No pre-oxygenation of Milli-Q water, no oxygen bubbling and no capping
Atmosphere (Oxygen flow)	-	Constant bubbling of oxygen through the vial during the reaction
Stirring bar	-	Glass or no stirring bar instead of Teflon stirring bar
Mechanism scavengers	12.5 mM	Added to vial before capping
pH	1 mM HCl (pH 3)<br>0.1 M phosphate buffer<br>1 mM NaOH (pH 11)	Pre-oxygenated solutions of Milli-Q water with HCl, phosphate buffer and NaOH were used
Ethanol (sacrificial agent)	0, 10, 20 and 50% ethanol in Milli-Q water	Pre-oxygenated (30 min) ethanol (100%)
Methanol (sacrificial agent)	0, 10, 20 and 50% ethanol in Milli-Q water	Pre-oxygenated (30 min) methanol (100%)
Cation exchangers	40 mM	Added to vial before capping
Sodium salts	40 mM	Added to vial before capping
Acetic acid	60 mM	Added to vial before capping
Lake and seawater	-	Utilized pre-oxygenated (30 min) instead of Milli-Q water
Biphasic systems	2 mL DCM/heptane with 2 mL Milli-Q water	Milli-Q water and heptane were pre- oxygenated and had oxygen bubbling, DCM was not oxygenated

5.6 Experimental procedure for blank reactions and results

Irradiation reactions of surfactants in Milli-Q water (4 mL) were conducted to confirm catalysis by the iron oxide nanoparticles. The chemicals were added (as described in **Table S4**) to a 10 mL vial with Milli-Q water (4 mL, 30 min pre-oxygenated). A Teflon stirring bar was added after which the vial was closed by capping. The sample was then extensively vortexed and sonicated for 5 min for better dispersion of the nanoparticles. Finally, 5 min of oxygen bubbling through the solution was conducted to ensure an oxygen atmosphere in the vial. Irradiation studies were conducted for 5h at 20°C by 445 nm irradiation in triplicate. Blanks in darkness were also performed as triplicate measurements for comparison.

From the results mentioned in **Table S4** the following conclusions can be made:

Entry 1,2: No photocatalytic production of H_2O_2 in darkness. Hydrogen peroxide production with light irradiation, where blanks in darkness were subtracted.

Entry 3-6: Blank reactions of starting materials/substrates used in catalyst synthesis. No production of H_2O_2.

Entry 8-11: Auto-photoxidation of sacrificial agents/electron donors when isopropanol or benzyl alcohol is utilized. No autoxidation for ethanol, methanol, heptane or dichloromethane. Allowing these solvents to be utilized in biphasic studies.

Entry 12-15: No production of hydrogen peroxide by pure autoxidation of oleic acid (4 mg). Trace amounts (less than during FeO_x catalysis) when large excess (50 mg) of oleic acid is utilized.

Entry 16-19: Addition of commercial iron oxides to oleic acid enhances cross-interference with redox titration (**ESI 6.2.3**), but shows a general trend to more peroxides/autoxidation.

Entry 20-25: Addition of FeOx NP to oleic acid, also limited by the redox interference (**ESI 6.2.3)**, significantly boosts the general production trend of peroxides and exceeds the commercial iron species (3x higher production, **Entry 23**), indicating the enhancement effect of iron NPs but also their own production behavior without addition of oleic acid.

Entry 26-35: Autoxidation observed for pure surfactants (elaidic acid, oleyl amine, oleyl sarcosine), but inactivity for H_2O_2 production when used as surfactants in FeOx NPs. Again, indicating a decoupled effect/mechanism than autoxidation of double bonds. Especially elaidic acid with a *trans* double bond possessing autoxidation activity while being inactive as FeO_x NPs highlights the iron oxide nanoparticle with *cis* oleic acid.

Entry 26-38: Only surfactants possessing double bonds showed autoxidation activity, while surfactants without double bond were inactive as FeO_x NPs and as blank surfactant for peroxide production.

Entry 39-41: Mechanism scavengers do not produce peroxides themselves, confirming the validity of the mechanism studies.

Entry 42-46: Lignin itself produces peroxides upon irradiation, as well as chitosan to a minor extend. Other carbon materials are inactive as blanks for peroxide production.

Entry 47-49: Pure water sources did not produce any peroxides upon irradiation.

Table S4: Blank reactions in darkness and light of reactants, surfactants, solvents and sacrificial agents.

Entry	Chemical 1 [mg]	Chemical 2 [mg]	Peroxide teststrip [mg L^{-1}]	Produced H_2O_2 [µmol L^{-1}]	Produced H_2O_2 [mmol g^{-1} L^{-1}]	Comment
**1**	FeO_x NP Standard (4 mg, darkness)	-	0	0	0	No production
**2**	FeO_x NP Standard (4 mg)	-	0.5 to 2	34±2	9.4±1.3	Hydrogen peroxide production, blanks in darkness always subtracted
**3**	$Fe(CO)_5$ (4 mg)	-	0	0	0	No production
**4**	Trimethylamine N-oxide (4 mg)	-	0	0	0	No production
**5**	Dibutyl ether (4 mL)	-	0.5	-	-	Ethers form peroxides upon irradiation
**6**	Dichloromethane (DCM, 4 mL)	-	0	0	0	No production
**7**	Heptane (4 mL)	-	0	0	0	No production
**8**	Methanol	-	0	0	0	No production
**9**	Ethanol	-	0	0	0	No production
**10**	Isopropanol	-	positive	-	-	non innocent
**11**	Benzyl alcohol	-	2 to 10	-	-	non innocent, even in darkness and air
**12**	Oleic acid (*cis;* 4 mg), darkness	-	0	0	0	No production
**13**	Oleic acid (*cis;* 4 mg)	-	0.5	0	-	No production
**14**	Oleic acid (cis; 50 mg), darkness		0	0	0	No production
**15**	Oleic acid (cis; 50 mg)	-	0.5 to 2	38±8	-	Autooxidation
**16**	Oleic acid (cis; 2 mg), darkness	Fe_3O_4 (2 mg)	0	42±12	19.4±6.3	Interference dissolved iron species
**17**	Oleic acid (cis; 2 mg), light	Fe_3O_4 (2 mg)	0.5 to 2	34±4	15.6±4.3	After subtraction of blank in darkness, trend visible, higher production, interference dissolved iron species

Entry	Chemical 1 [mg]	Chemical 2 [mg]	Peroxide teststrip [mg L^{-1}]	Produced H_2O_2 [µmol L^{-1}]	Produced H_2O_2 [mmol g^{-1} L^{-1}]	Comment
**18**	Oleic acid (cis; 4 mg), darkness	Fe_3O_4 (4 mg)	0 to 0.5	44±1	11.1±0.1	Interference dissolved iron species
**19**	Oleic acid (cis; 4 mg), light	Fe_3O_4 (4 mg)	2	29±4	7.2±0.6	Trend visible, higher production, interference dissolved iron species
**20**	Oleic acid (cis; 2 mg), darkness	FeO_x NPs (3.5 mg, batch 129)	0 to 0.5	184±16	56.7±6.1	Interference dissolved iron species
**21**	Oleic acid (cis; 2 mg)	FeO_x NPs (3.5 mg, batch 129)	2 to 5	66±22	12.6±9.7	Trend visible, higher production, interference dissolved iron species, more production *via* NPs than commercial Fe_3O_4
**22**	Oleic acid (cis; 4 mg), darkness	FeO_x NPs (6 mg, batch 129)	0 to 0.5	247±58	42.0±9.6	Interference dissolved iron species
**23**	Oleic acid (cis; 4 mg)	FeO_x NPs (6 mg, batch 129)	5	127±30	20.3±1.9	Trend visible, higher production, interference dissolved iron species, more production *via* NPs than commercial Fe_3O_4
**24**	Oleic acid (cis; 50 mg), darkness	FeO_x NPs (6 mg, batch 129)	0 to 0.5	410±12	69.9±2.5	Interference dissolved iron species
**25**	Oleic acid (cis; 50 mg)	FeO_x NPs (6 mg, batch 129)	5	20±6	2.4±0.5	Trend visible, higher production, interference dissolved iron species, more production *via* NPs than commercial Fe_3O_4

Entry	Chemical 1 [mg]	Chemical 2 [mg]	Peroxide teststrip [mg L^{-1}]	Produced H_2O_2 [µmol L^{-1}]	Produced H_2O_2 [mmol g^{-1} L^{-1}]	Comment
**26**	Linoleic acid (*cis,cis*; 4 mg)	-	0	0	-	Autooxidation, more than oleic acid
**27**	Linoleic acid (*cis,cis*; 4 mg)	-	2 to 5	35	-	Autooxidation, more than oleic acid
**28**	Elaidic acid (*trans*; 4 mg), darkness	-	0 to 0.5	39±1	-	Autooxidation, inactive in NP catalysis
**29**	Elaidic acid (*trans;* 4 mg), light	-	0.5	0	-	Autooxidation, inactive in NP catalysis
**30**	Elaidic acid (*trans;* 50 mg), darkness	-	0 to 0.5	41±2	0	Autooxidation, inactive in NP catalysis
**31**	Elaidic acid (*trans;* 50 mg), light	-	0.5 to 2	0	0	Autooxidation, inactive in NP catalysis
**32**	Oleylamine (4 mg)	-	0 to 0.5	39±0	-	Autooxidation in contrast to oleic acid (4 mg)
**33**	Oleylamine (50 mg)	-	2	26±8	-	Autooxidation
**34**	N-oleylsarcosine (4 mg)	-	0 to 0.5	22±4	-	Autooxidation in contrast to oleic acid (4 mg)
**35**	N-oleylsarcosine (50 mg)	-	2	85±11	-	Autooxidation
**36**	Octanoic acid (4 mg)	-	0	0	0	No production
**37**	Octanoic acid (50 mg)	-	0	0	0	No production
**38**	Stearic acid (50 mg)	-	0	0	0	No production

Entry	Chemical 1 [mg]	Chemical 2 [mg]	Peroxide teststrip [mg L^{-1}]	Produced H_2O_2 [µmol L^{-1}]	Produced H_2O_2 [mmol g^{-1} L^{-1}]	Comment
**39**	*p*-benzoquinone (6 mg)	-	0	0	0	No production, mechanism scavenger experiment is valuable
**40**	Silver nitrate (8 mg)	-	0	0	0	No production, mechanism scavenger experiment is valuable
**41**	Tert-butyl alcohol (5 mg)	-	0	0	0	No production, mechanism scavenger experiment is valuable
**42**	Lignin	-	30 to 100	-	-	non innocent
**43**	Chitosan	-	0 to 0.5	-	-	non innocent
**44**	Cellulose	-	0	0	0	No production
**45**	Graphene	-	0	0	0	No production
**46**	Active Charcoal	-	0	0	0	No production
**47**	Lake water	-	0	0	0	No production
**48**	Seawater	-	0	0	0	No production
**49**	Milli-Q water	-	0	0	0	No production

The surfactants were further studied for autoxidation behavior *via* ^{1}H-NMR. The surfactants (oleic acid, linoleic acid, linolenic acid, oleylamine, nonanoic acid) were each oxygenated in a closed vial *via* bubbling through the reactant for 30 minutes and stored in O_2 atmosphere over night. The ^{1}H-NMR spectra were measured after 20 h. Surfactants with no (nonanoic acid) or one double bond (oleic acid, oleylamine) did not possess significant autoxidation properties as the peroxide test strips did not change in colour and no changes *via* NMR was observed (**ESI 14.2**). However, surfactants with two (linoleic acid) or three (linolenic acid) double bonds showed increased amounts of peroxides through blue test strips. No changes in the NMR spectra were obtained while peroxide teststrips turned blue (autoxidation), confirming that the detection limit was not reached *via* blank autoxidation of the surfactants (linoleic acid, linolenic acid).

6. Quantification of hydrogen peroxide

6.1 Peroxide test strips

Peroxide test strips contain an organic redox indicator. Upon contact with peroxides the peroxide test strips produce a blue oxidation product. The peroxide concentration is measured **semiquantitatively** by visual comparison of the reaction zone of the test strip with the fields of a colour scale. For accurate measuring the pH of the samples should be within the range 2-12.

Measurements were performed by immersing the test strips for one second in the samples. Excess liquid was allowed to run off and after approximately 10-15 sec a **semiquantitative** comparison was made using the colour scale.

In case organic solvents were utilized, a slightly different procedure was necessary. The peroxide test strips were first immersed in the organic sample. Subsequently a few water drops were utilized to humidify the reaction zone. A **semiquantitative** comparison could then be made 10-15 sec after the water drops were added to the reaction zone.

Peroxide test strips are depicted in **Figure S98** indicating produced hydrogen peroxide in Milli-Q water without additives after 5 h irradiation (445 nm) and their comparison in darkness.

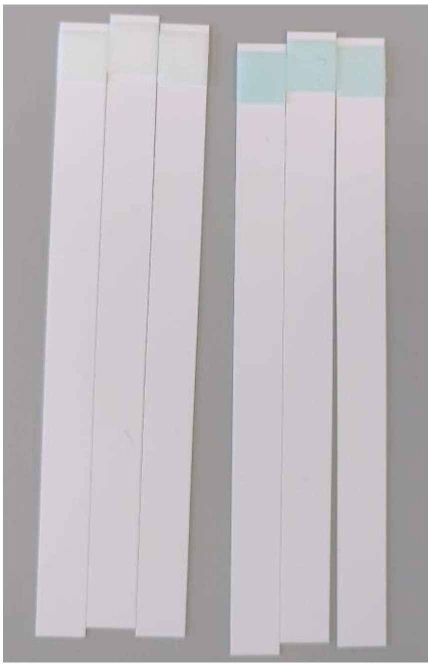

Figure S98: Semiquantitative analysis by peroxide test strips of sample irradiated (445 nm) for 5h (right), and their respective blanks in darkness (left).

6.2 Iodometric titration

Reaction S1: Iodide oxidation by hydrogen peroxide.

$$H_2O_2 + H_2SO_4 + 2\,KI \rightarrow I_2 + K_2SO_4 + 2\,H_2O$$

Reaction S2: Iodometric titration.

$$I_2 + 2\,Na_2S_2O_3 \rightarrow Na_2S_4O_6 + 2\,NaI$$

It was opted for iodometric titration, because titration by potassium permanganate ($KMnO_4$) led to interference with organic matter such as the surfactants of the FeO_x photocatalyst.

6.2.1 Stock solution preparation

Potassium iodide solution (2 wt%)

Potassium iodide (2 g, 12.04 mmol) was dissolved in demineralized water (100 mL). The solution was stored in a brown bottle to protect it from light irradiation. Properly stored, the solution is stable for six months, provided no change occurs in the colour of the solution.[17]

Ammonium molybdate solution

Ammonium molybdate tetrahydrate (9 g, 7.7 mmol) was dissolved in ammonium hydroxide (10 mL aqueous NH_3 (25%, 13.5M), of which 42% is converted to ammonium hydroxide 5.6M NH_4OH). Subsequently, ammonium nitrate (24 g, 0.3 mol) was added and the reaction mixture was diluted to 100 mL using Milli-Q water.

Sulfuric acid solution (3.5M)

One part of sulfuric acid (98%) was added carefully to four parts of demineralized water. This resulted in a solution that is stable indefinitely. Acid was always added to water, not water to acid, to avoid excess heat formation and spitting of acid. The solution was stirred while adding acid.[17]

Starch indicator solution

Reagent-grade NaCl was dissolved in double-distilled water (100 mL) while stirring, until no more dissolved. The beaker was subsequently heated until everything dissolved. NaCl crystals were observed on the sides of the beaker while cooling. This resulted in a solution that is stable for up to 12 months. Chemical starch (1 g) was dissolved in double-distilled water (10 mL) in a separate piece of glassware. The reaction mixture was heated until the contents were dissolved. The saturated NaCl solution was added to make 100 mL starch solution.[17]

Sodium thiosulphate solution (0.0001M)

Sodium thiosulphate (0.0316 g, 0.2 mmol) was dissolved in Milli-Q water (2 L). The resulting solution was stable for at least one month, if stored in the dark.

Standardization of sodium thiosulfate with a volumetric solution of potassium iodate is recommended. The concentration of the sodium thiosulfate could be adjusted to accommodate the analysis of larger sample weights.[17]

Normalization of sodium thiosulphate solution (0.0001M)

Every two weeks the sodium thiosulphate solution was standardized with a volumetric solution of potassium dichromate. Potassium dichromate (0.2 mg, 0.68 µmol) was dissolved in Milli-Q water (10 mL) in an Erlenmeyer flask. Subsequently, potassium iodide solution (5 mL, 2 wt%, 602 µmol),

sulfuric acid (2 mL, 3.5 M), ammonium molybdate solution (1 mL) and starch indicator (2 mL) were added to the potassium dichromate solution, resulting in a blue solution (**Reaction S3**). The blue colour was titrated away by dropwise addition of sodium thiosulphate solution (0.0001M) (**Reaction S4**).

Reaction S3: Oxidation of potassium iodide by potassium dichromate.

$$K_2Cr_2O_7 + 6\,KI + 7\,H_2SO_4 \rightarrow\ Cr_2(SO_4)_3 +\ 4\,K_2SO_4 + 7\,H_2O + 3\,I_2$$

Reaction S4: Reaction between sodium thiosulphate and iodine.

$$2\,Na_2S_2O_3 +\ I_2\ \rightarrow 2\,NaI +\ Na_2S_4O_6$$

6.2.2 Quantification procedure

Iodometric titration was utilized to accurately quantify the hydrogen peroxide amounts produced *via* the ORR. After uncapping the vials, they were first **semiquantitatively** analyzed *via* peroxide test strips. Subsequently, the Milli-Q water was decanted in a 20 mL vial while a magnet was held to the bottom of the initial 10 mL vial to ensure catalyst to stay in the vial. The inside of the 10 mL vial was washed once with Milli-Q water (1 mL), which was also decanted in the 20 mL vial for analysis.

Syringe filters (0.2 µm, Sartorius) were used in case solids were dispersed in the sample (e.g. iron oxides as additive) or when an suspension was obtained after reaction. The samples were then decanted into a syringe (5 mL) with a filter (0.2 µm) instead of decantation directly into the 20 mL vial.

For analysis potassium iodide solution (2 mL), sulfuric acid solution (1 mL) and ammonium molybdate solution (5 drops) were added to the sample. The vial was then immediately capped and stored in darkness for (exactly) 5 min. After these 5 min in darkness the solution had turned from colourless to slightly yellow. Upon addition of starch indicator, a blue/purple colour was obtained, which was titrated away with 0.0001 M sodium thiosulfate solution.

The amount of potassium iodide added to each sample should be in excess. 2 mL KI (2wt%) = 236 µmol, of which 118 µmol are available for reaction with H_2O_2 (**Reaction S1**). This amount is a few orders of magnitude higher (81 – 844 times) than typical produced amounts of hydrogen peroxide (0.14 - 1.45±0.07 µmol).

6.2.3 Troubleshooting iodometry

As mentioned in **ESI 6.2.2**, samples were capped and stored in darkness for exactly 5 minutes. It was found to be crucial that every sample stood in darkness for 5 min, as otherwise comparison was not possible anymore. Over time potassium iodide is oxidized by oxygen and carbon dioxide to form iodine and potassium carbonate, which interferes with the measurements.

Also, having iron ions in solution interfered with iodometric test results *via* **Reaction S5**. This effect was observed when iron oxides were added to the reaction mixtures, but also in the blank reactions (**ESI 5.6**) when surfactants dissolved the iron oxide nanoparticles. In standard screening reactions with iron oxide nanoparticles this effect was not observed, as successful heterogeneous immobilization of the nanoparticles on glass surfaces was achieved without considerable amounts of leaching.

Reaction S5: Interference by iron ions to form iodine from iodide.[18]

$$2\,Fe^{3+} + 2\,I^-\ \rightarrow 2\,Fe^{2+} +\ I_2$$

6.3 HPLC-MS

Method: Liquid-chromatography (LC) mass-spectrometry (MS) (LC-MS) measurements were performed on an Agilent InfinityLab LC/MSD (G6125C SG2215N102) with an Agilent 1290 Infinity II. A non-polar column by Waters (BEH-C4, 2.1x150, 1.7 micron) was utilized with 100.0% Water as eluent at a flow rate of 0.300 mL min^{-1} and 600.00 bar pressure (26 min acquisition time). The injection volume was set to 1.00 µL. The UV-DAD detector followed products at wavelengths of 200 nm, 210 nm and 250 nm and full spectra were recorded from 190 nm to 350 nm. The SQ Mass Spectrometer was set to follow mass values ranging from 40-600 m/z. Simultaneously SIM scans at masses of 312 m/z, 313 m/z and 281 m/z were conducted.

As unsaturated fatty acids are able to undergo autoxidation to hydroperoxides, it was important to identify whether hydrogen peroxide or organic peroxides were produced using iron oxide nanoparticles. Titration as previously mentioned is an accurate method to quantify peroxides, however no distinction can be made between different peroxides. A method was developed using high-performance liquid chromatography-mass spectrometry (HPLC-MS) allowing for separation of potentially formed different peroxides.
Hydrogen peroxide was found to leave the column at a retention time of ~1.5 min. This was confirmed and established by increased peak intensity of the UV-diode array detector (DAD), when calibrating with different concentrations of H_2O_2 in water ($1x10^{-9}$ mol/L, $1x10^{-6}$ mol/L, 30 ppm, 3%, 30%).
Standard irradiation studies for 5h at 445 nm with FeO_x NPs (oleic acid (2:1) surfactant (batch 131) were conducted for photochemical production of H_2O_2, see **ESI 5.5**. After the reaction was completed, the aqueous sample was analyzed by HPLC/DAD-MS. The chromatographic peak at 1.5 min was identified as hydrogen peroxide by comparing retention time and UV absorption spectra using an analytical standard solution. An external calibration curve was built to quantify the amount of hydrogen peroxide produced by our proposed reaction. Finally, by decoupling the outlet of the column from the MS and dropping the eluent directly on peroxide-specific test strips, the presence of hydrogen peroxide was confirmed by appearance of a blue colour at ~1.5 min as shown in **Figure S101**. Blanks were also tested (water and blank in darkness; also highlighted in **Figure S99**), where test strips did not show any alteration in color, serving as final proof for the presence of hydrogen peroxide.

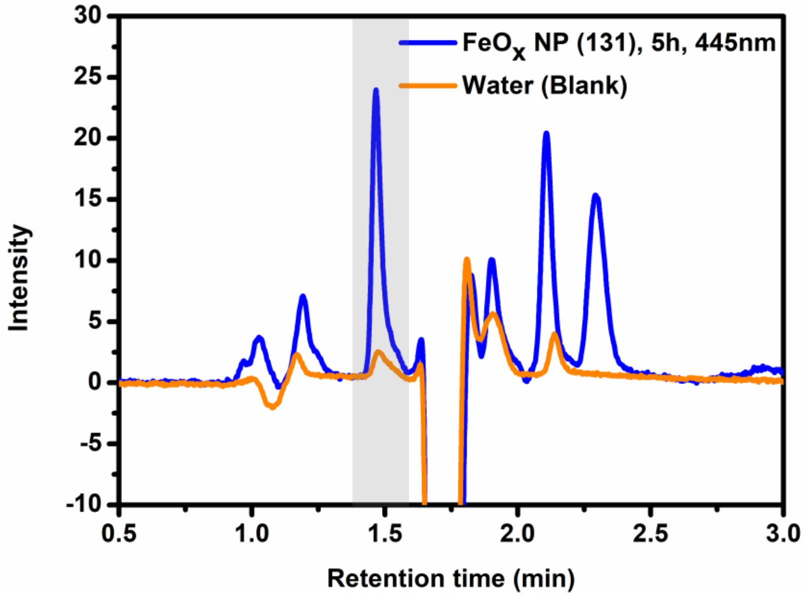

Figure S99: Hydrogen peroxide is confirmed *via* the UV-DAD detector at a retention time of 1.5 min using HPLC. Light vs. darkness hydrogen peroxide measured by LC-DAD at 240 nm. HPLC/DAD chromatogram at 240 nm of the sample (blue) and blank (orange line). Hydrogen peroxide elution time is 1.5 min.

An increased intensity of the peak at 1.5 min was obtained *via* UV-DAD compared to its blank (**Figure S99**). Correspondence to H_2O_2 was confirmed by calibration with different concentrations of H_2O_2 solutions. The absorbance spectrum of the compound leaving the column at 1.5 min retention time (as highlighted by **Figure S100**) perfectly resembled an absorbance spectrum of H_2O_2 (see **Figure S106**).

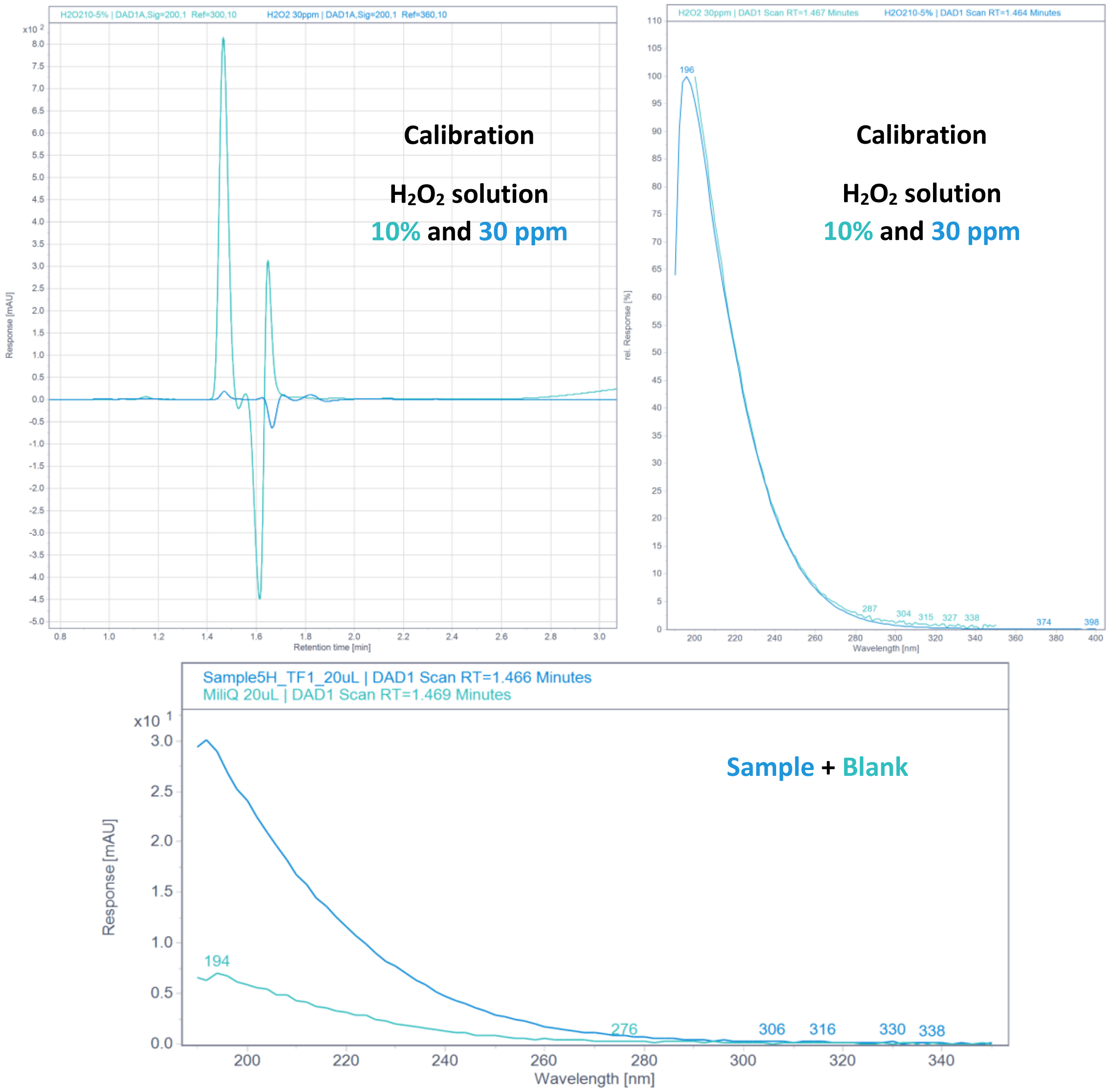

Figure S100: UV-Vis absorbance spectra of the peak at 1.5 min in comparison with an analytical standard solution of H_2O_2.

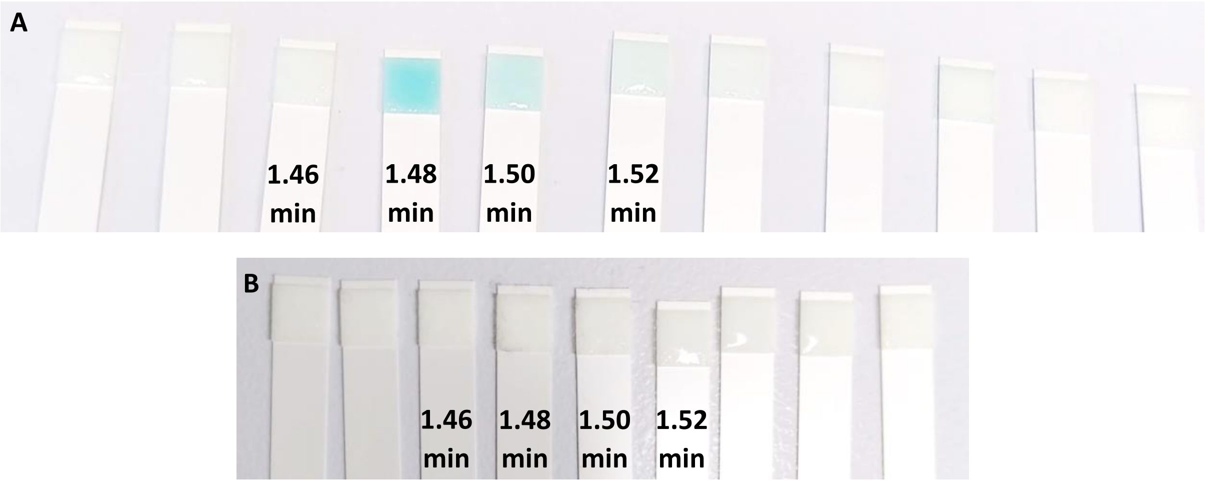

Figure S101: Peroxide test strip cross-validation for hydrogen peroxide identification at retention time ca. 1.5 min. a) FeO_x promoted production of H_2O_2 in water at 445 nm. b) Blank reaction in darkness as well as pure water eluent measurements.

Quantifying the present amounts of oleic acid and hydroperoxide was possible between 15-18 minutes (retention time, **Figure S102**). The presence of the hydroperoxide could be confirmed by the the negatively charged molecular ions m/z 312 and 313 depicted in **Figure S103**. Interestingly, the amount of hydroperoxide observed in the blank reaction (darkness) was significantly higher than for the irradiation experiments. A calibration curve was built for the target compounds, which facilitated the calculation of the ratio between the different peroxides (hydrogen peroxide and hydroperoxide) (**Table S5**). To our delight, reaction **selectivity greater than 99%** towards hydrogen peroxide was observed, with only trace amounts of oleic acid and hydroperoxide being detected.

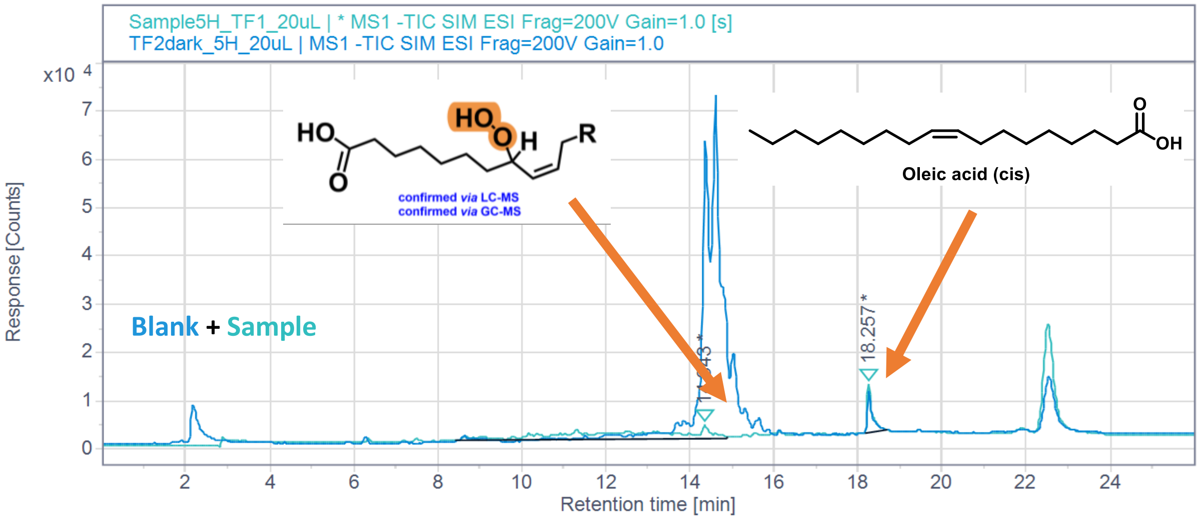

Figure S102: HPLC-MS total ion chromatogram (TIC) of sample (irradiated) and blank (darkness). Hydroperoxide elution time is 15 min and oleic acid is 18.2 min.

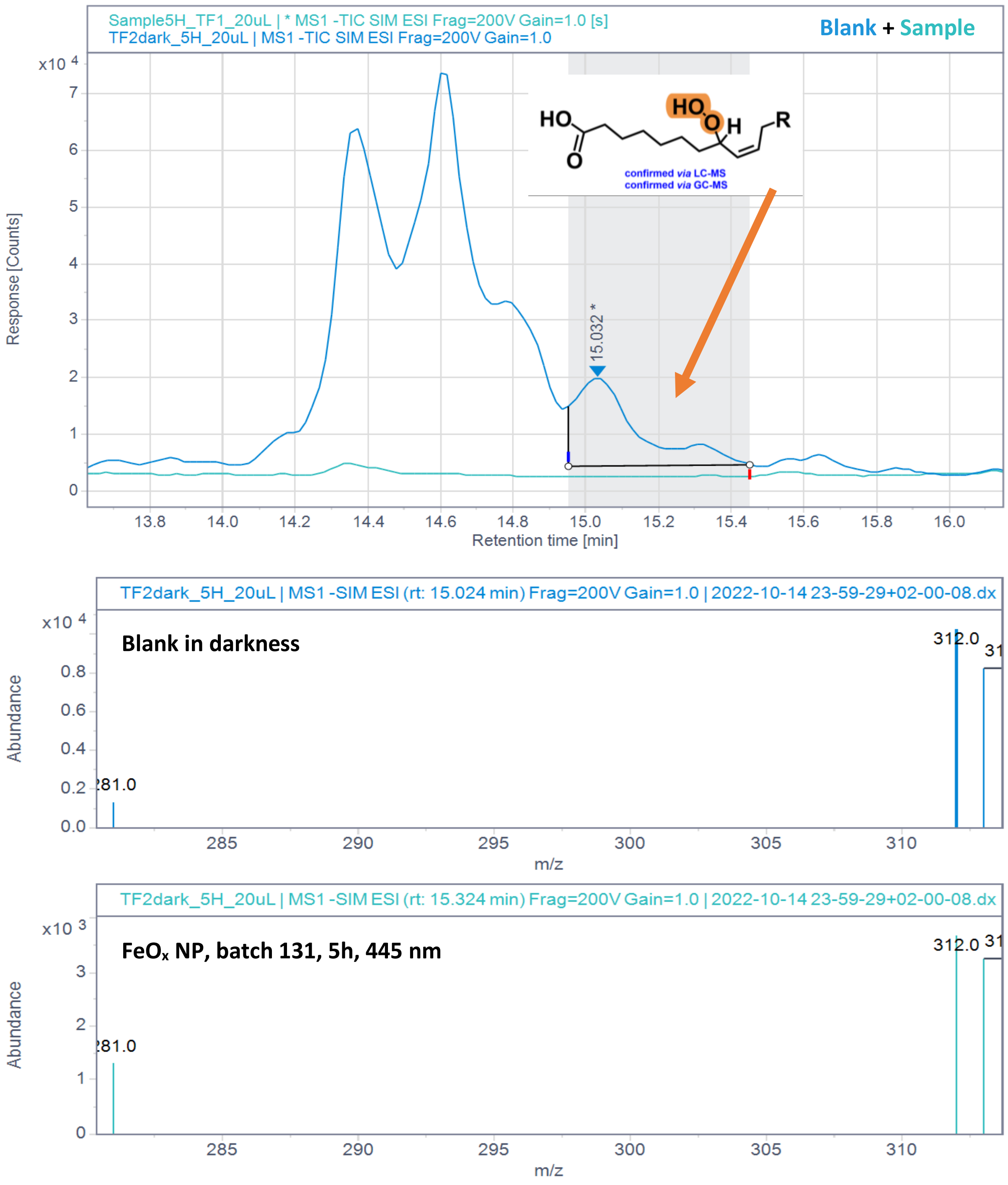

Figure S103: Light vs. dark zoomed in at retention time hydroperoxide; LC-MS SIM m/z 312 and 313 (hydroperoxide oleic acid). Zoomed in fraction of hydroperoxide retention time (top), and its mass spectra in light and blank in darkness (bottom), MS-SIM m/z 312 and 313.

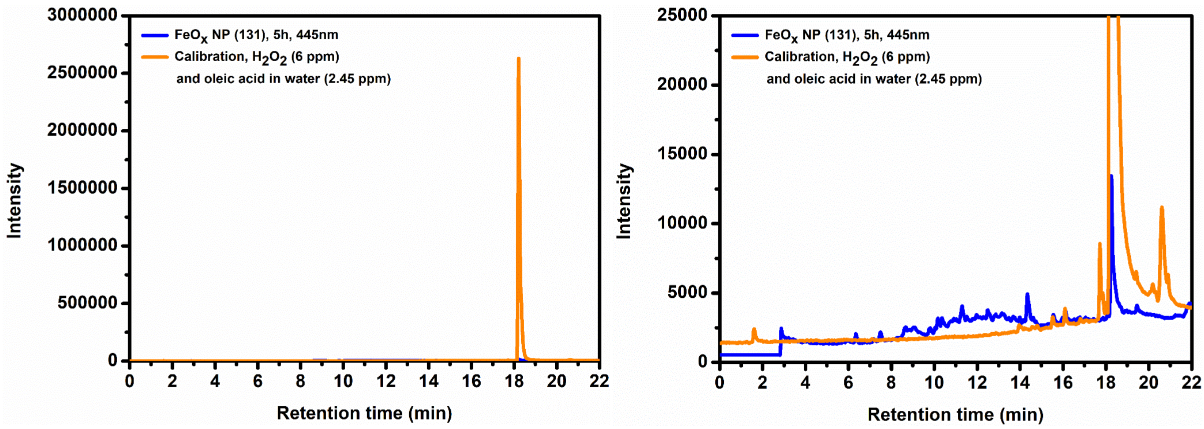

Figure S104: HPLC-MS total ion chromatogram of oleic acid analytical standard (orange line, 6ppm) and sample (blue line)

Table S5: Areas and calculation after calibration for selectivity for H_2O_2 production over hydroperoxide production.

Condition		Amount	Area	Concentration [mmol L^{-1}]	Molar ratio	Selectivity [%]
**Calibration Standard**	Hydroperoxide of oleic acid	0	0	0	0	-
	oleic acid	25 ppm	12091957	0.089	1	-
	H_2O_2	30 ppm	25	0.882	9.96	-
**Sample**	Hydroperoxide of oleic acid	-	25303.295	**0.00024**	1	**0.01**
	oleic acid	-	81894.266	**0.00054**	2.2	**0.03**
	H_2O_2	-	55.0125	**1.94084**	8006	**99.96**

6.4 Qualitative validation *via* UV-Vis (H_2O_2, resorufin and iodometric assay)

Produced amounts of hydrogen peroxide were cross validated by UV-Vis spectroscopy using iodometry and resorufin.

Hydrogen peroxide reacts with Amplifu red in the presence of horseradish peroxidase towards pink coloured resorufin with an absorption maximum of 571 nm depicted in **Figure S105A**. For this 2 mL of a produced H_2O_2 solution after 5h of irradiation (batch 135) was allowed to react with Amplifu red in the presence of horseradish peroxidase. Similarly, production of hydrogen peroxide was cross-validated by checking the absorbance of the formed triiodide anions at 352 nm. For this potassium iodide was added to a solution of H_2O_2 produced by ORR *via* FeO_x NPs, followed by addition of sulfuric acid and molybdenum catalyst for triiodide anion formation (**Chapter 6.2**). Increased absorbance was obtained at 352 nm for H_2O_2 solutions produced in light, where production in darkness gave zero absorbance (**Figure S105B**). This confirmed formation of hydrogen peroxide by photocatalysis. To obtain these results the potassium iodide (KI) blank had to be subtracted as it was found, that KI is oxidized by oxygen in air, stressing the importance of 5 minutes reaction time for iodometric titrations in future results.

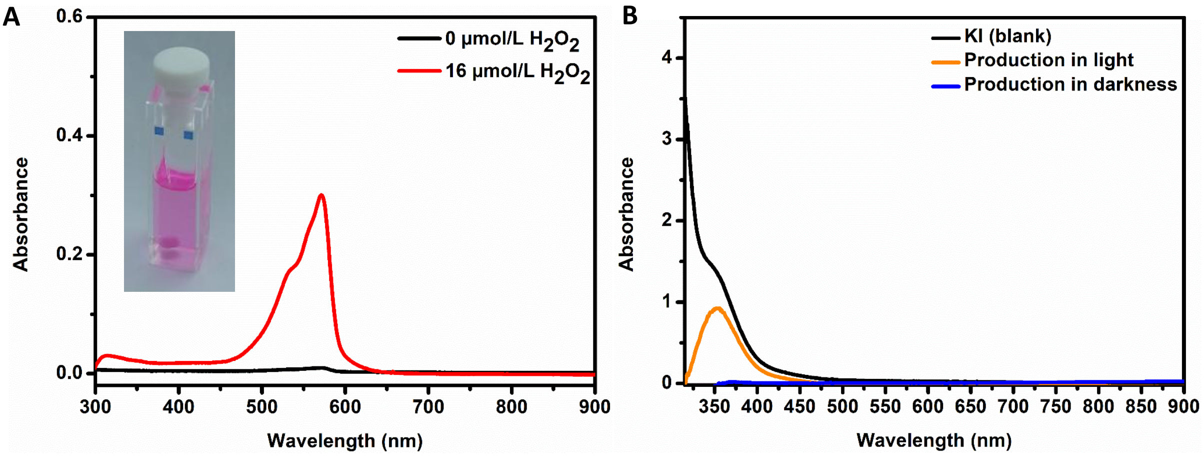

Figure S105: a) Calibration curve for resorufin absorbance at wavelength 571 nm (top), absorption resorufin produced by measured sample (bottom); insert: resorufin in a cuvette, produced by hydrogen peroxide from batch 135 (FeO_x with oleic acid 2:1, 5h irradiation in O_2 atmosphere, 445 nm). b) Dilution of iodide formed after 5 min by air (top) and produced hydrogen peroxide (FeO_x with oleic acid, batch 131) by nanoparticles *via* iodine (352 nm) formation (bottom).

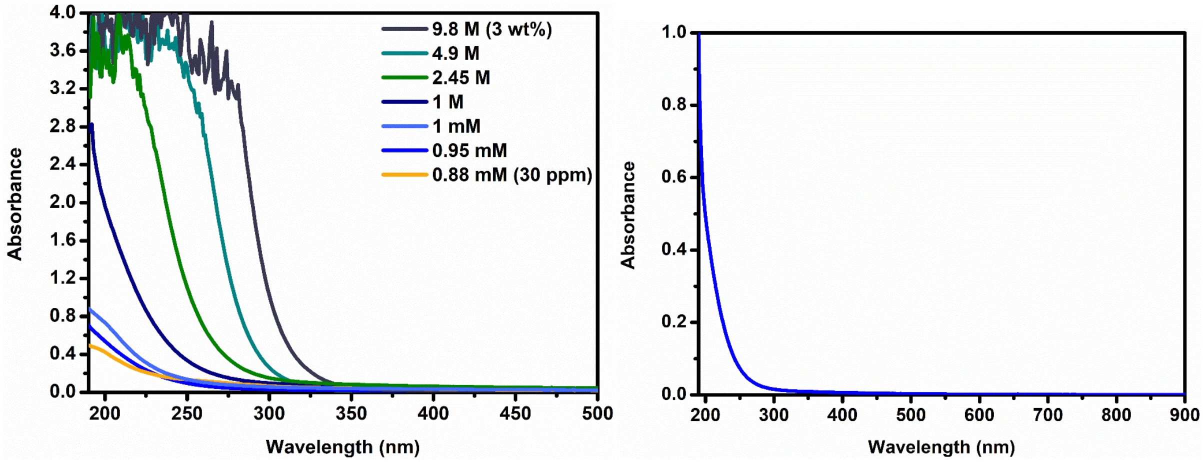

Figure S106: UV-Vis spectrum of H_2O_2 at different concentrations in Milli-Q water (top). Normalized absorption spectrum of H_2O_2 (1 mM) in Milli-Q water. Photochemical production or degradation of H_2O_2 can be followed at 200 nm or 240 nm.

6.5 ^{1}H-NMR spectroscopy

An *in-situ* oxidation of *o*-tolidine was attempted as a quantification method *via* ^{1}H-NMR. Production however could not be observed *via* ^{1}H-NMR as too less hydrogen peroxide was produced, not reaching the detection limit (**Figure S107**). For that *o-T*olidine (362 µmol) was irradiated (445 nm) for 60 h in 4 mL methanol with 4 mg FeO_x NPs (1 mg mL^{-1}). The NMR was taken in DMSO-d_6. Ultimately, produced amounts of hydrogen peroxide were not enough for more advanced quantification methods (e.g. ^{1}H-NMR).

o-Tolidine:
^{1}H NMR (400 MHz, DMSO-d_6) δ 6.95 (d, *J* = 2.2 Hz, 2H), 6.92 (dd, *J* = 8.0, 2.2 Hz, 2H), 6.46 (d, *J* = 8.0 Hz, 2H), 1.93 (s, 6H).

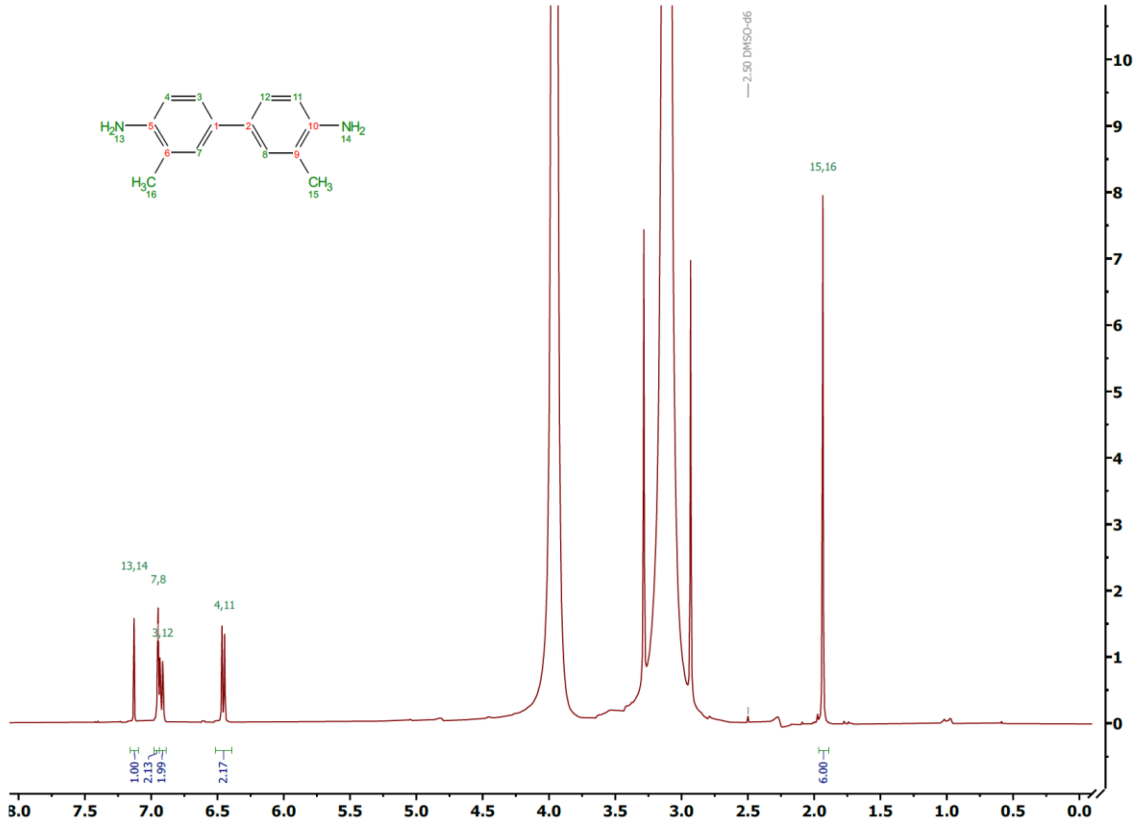

Figure S107: ^{1}H-NMR spectrum of *o*-Tolidine (362 µmol) in 4 mL methanol with 4 mg FeO_x NPs, irradiated with 445 for 60h. No reaction was observed as the detection limit of ^{1}H-NMR was not reached (solvent: DMSO-d_6).

6.6 GC-MS

Trace amounts of oleic acid hydroperoxide were also confirmed by GC-MS analysis. Hydroperoxide and oleic acid were extracted from an aqueous sample (5 h irradiated) and derivatized (silylation and methylation, respectively) prior to chromatographic analysis to facilitate their separation and detection.

6.6.1 Derivatization procedure (esterification and silylation)

Derivatization of the hydroperoxide of oleic acid for its detection by GC-MS *via* mild methanolysis/methylation and rapid methanolysis/methylation using conc. HCl. and subsequent silylation.

Before conducting the experiments on samples, the method was successfully validated on pure oleic acid (90%, Sigma-Aldrich) where a solution of 7.8 g in 16 mL toluene was prepared and 0.3 mL (146 mg oleic acid (90%) in 0.3 mL toluene) used for subsequent acidic methylation.

After reaction in light and darkness (H_2O_2 in 4 mL Milli-Q water) the water was separated from the heterogeneous FeO_x *via* decantation as it was sticking to the bottom of the glass and separated also *via* magnet. The samples were washed with 1 mL Milli-Q water and filtered through a 0.2 µm syringe filter.

For the LC-MS studies, 1 mL solution of the sample was used without pre-treatment. For the GC-MS studies on the other hand, the remaining 3 mL were extracted with toluene (3 x 2 mL) and subsequently evaporated under nitrogen flow until a volume of 0.3 mL. Reagent grade HCl (35%, w/w; 9.7 ml) was diluted with 41.5 ml of methanol to make 50 mL of 8.0% (w/v) HCl. This HCl reagent contained 85% (v/v) methanol and 15% (v/v) water that was derived from conc. HCl and was stored at 8 °C. To the sample solution, 1.50 mL of methanol and 0.30 mL of the 8.0% HCl solution were added in this order. The final HCl concentration was 1.2% (w/v) or 0.39 M, which corresponded to 0.06 mL of conc. HCl in a total volume of 2.1 mL. After addition of the acidified methanol to each sample, it was stored under nitrogen atmosphere and capped. The samples were heated at 60°C for 12h for mild methanolysis/methylation and then cooled down to room temperature. The methanol solution was transferred to a 20 mL vial containing 0.4-1 mL hexane. An organic free NaCl (5%, 5 mL) solution was added, the vial closed and the solution vortexed for 30s. The hexane fraction was separated *via* pipette into a 4 mL vial and the extraction repeated 3x 0.4 mL. The obtained solution was dried over $MgSO_4$, washed with 1 mL hexane and concentrated to ~0.5 mL *via* N_2 flow.

The solution of N,O-bis(trimethylsilyl)trifluoroacetamide (BSTFA/TMS) was added for silylation and capped in a 4 mL vial in N_2 atmosphere. The reaction was heated at 70°C for 2h. The product mixture was dried under N_2 flow and 1 mL of hexane added, which was transferred to a 1 mL GC-MS vial in N_2 atmosphere for analysis.[19]

7. Catalyst performance

Iodometric titration is a successful method for accurate quantification of H_2O_2 as confirmed and cross validated *via* several techniques (positive peroxide test strips, HPLC-MS, UV-Vis resorufin, UV-Vis iodometric essay), see **ESI 6.4**). Furthermore, the extensive work and method development *via* HPLC-MS confirmed H_2O_2 production with 99% selectivity. In all screenings it was opted to add 4 mg of catalyst to 4 mL of Milli-Q water, to obtain a catalyst loading of around 1 mg mL^{-1} and the highest production values (**Figure S108**). 7 mg of catalyst in 4 mL of Milli-Q water was found to have significantly lower production of hydrogen peroxide, presumably through stronger Fenton-decomposition as more iron is present. This also indicates that production is not limited by catalyst amount, since more catalyst resulted in less production. 4 mg catalyst in 8 mL Milli-Q water was found to be slightly lower in hydrogen peroxide production than 1 mg mL^{-1}. However, these were in each other's error bars, not indicating any dilution effect of the solvent on Fenton chemistry or H_2O_2 decomposition. In future screening results a catalyst loading of 1 mg mL^{-1} is maintained.

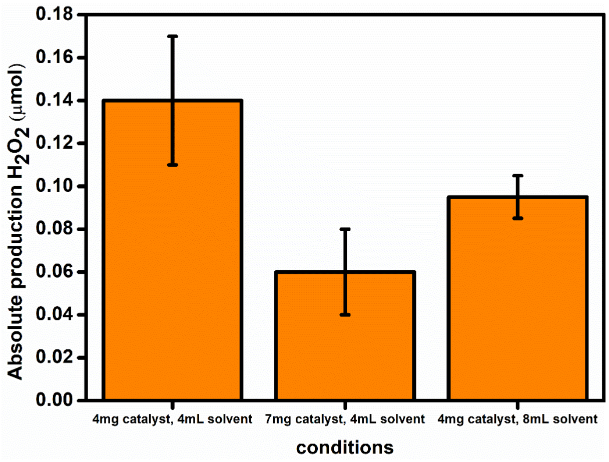

Figure S108: Screening results of catalyst loading for iron oxide batches with oleic acid (2:1) surfactant, obtained by 5h irradiation with 445 nm at 20°C in Milli-Q water at 20°C.

7.1 List of photochemical H_2O_2 production values

Table S6: List of production values for the photochemical production of H_2O_2 *via* FeO_x NPs.

Entry	Condition	Peroxide Teststrip [mg L^{-1}]	Produced H_2O_2 [µmol L^{-1}]	Produced H_2O_2 [mmol g^{-1} L^{-1}]	Productivity H_2O_2 [mmol g^{-1} L^{-1} h^{-1}]	Normalized production H_2O_2 [mmol g^{-1} L^{-1}]
**1**	FeO_x NP Standard (4 mg, darkness)	0	0	0	0	0
**2**	FeO_x NP Standard (4 mg)	0.5 to 2	34±2	9.4±1.3	1.7±0.3	9.4±1.3
**Recycling**						
**3**	Round 1	2	24±9	6.0±2.0	1.2±0.4	9.0±2.0
**4**	Round 2	2	24±2	6.3±0.9	1.3±0.2	9.6±0.9
**5**	Round 3	0.5 to 2	24±6	6.4±1.9	1.3±0.4	9.7±1.9
**6**	Round 4	2	30±1	7.8±0.4	1.6±0.1	11.8±0.4
**7**	Round 5	0.5 to 2	16±6	4.3+1.6	0.9±0.3	6.5±1.6
**8**	Round 6	0.5 to 2	19±3	4.9±1.1	1.0±0.2	7.4±1.1
**9**	Round 7	0.5	15±4	4.0±1.2	0.8±0.2	6.1±1.2
**10**	Round 8	0 to 0.5	1±1	0.5±0.5	0.1±0.1	0.7±0.5
**11**	Round 9	0 to 0.5	11±11	3.1±2.4	0.6±0.5	4.7±2.4
**12**	Round 10	0 to 0.5	2±1	0.6±0.3	0.1±0.1	0.9±0.3
**FeO_x NPs surfactant scope**						
**13**	No surfactant	0	0	0	0	0
**14**	Octanoic acid	0	0	0	0	0
**15**	Nonanoic acid	0	0	0	0	0
**16**	Lauric acid	0	0	0	0	0
**17**	Oleic acid	0.5 to 2	34±2	9.4±1.3	1.7±0.3	9.4±1.3
**18**	Elaidic acid	0	0	0	0	0
**19**	Linoleic acid	0.5 to 2	35±7	7.9±1.9	1.6±0.4	7.9±1.9
**20**	Oleyl alcohol	0	0	0	0	0
**21**	Oleylamine	0	0	0	0	0
**22**	N-Oleylsarcosine	0	0	0	0	0
**23**	OA@graphene	0	0	0	0	0
**24**	OA@charcoal	0 to 0.5	16±6	2.4±1.6	1.0±0.6	2.4±1.6
**Kinetics FeO_x NPs with oleic acid surfactant**						
**25**	1 h	0.5	10±2	2.6±1.4	2.6±1.4	2.6±1.4
**26**	2.5 h	0.5	13±2	3.9±1.0	1.6±0.4	3.9±1.0
**27**	5 h	0.5 to 2	29±2	9.0±0.4	1.8±0.1	9.0±0.4
**28**	20 h	2	36±1	14.2±1.1	0.7±0.1	14.2±1.1
**29**	67 h	2 to 5	63±13	12.8±2.5	0.2±0.1	12.8±2.5
**pH**						
**30**	pH = 3	0.5 to 2	48±8	11.2±1.8	2.2±0.4	14.8±1.8
**31**	pH = 7	0.5 to 2	32±6	6.8±0.5	1.4±0.1	9.0±0.5
**32**	pH = 7.24 (buffer)	0.5 to 2	20±3	4.8±0.7	1.0±0.1	6.3±0.7
**33**	pH = 11	2 to 5	26±5	6.1±1.0	1.2±0.2	8.0±1.0

Entry	Condition	Peroxide Teststrip [mg L^{-1}]	Produced H_2O_2 [µmol L^{-1}]	Produced H_2O_2 [mmol g^{-1} L^{-1}]	Productivity H_2O_2 [mmol g^{-1} L^{-1} h^{-1}]	Normalized production H_2O_2 [mmol g^{-1} L^{-1}]
**Atmosphere**						
**34**	Nitrogen (5h)	0	0	0	0	0
**35**	Air (5h)	0.5	18±4	4.5±1.0	0.9±0.2	4.5±1.0
**36**	Oxygen (5h)	0.5 to 2	29±2	9.0±2.0	1.8±0.1	9.0±2.0
**37**	Open air (5h)	0.5	8±2	1.6±1.5	0.3±0.3	2.9±1.5
**38**	Oxygen flow (5h)	0.5 to 2	26±3	7.0±2.7	1.4±0.5	12.6±2.7
**39**	Nitrogen (3d)	0	0	0	0	0
**40**	Oxygen (3d)	2 to 5	63±13	12.8±2.5	0.2±0.1	12.8±2.5
**Mechanism scavenger**						
**41**	No scavenger	0.5 to 2	44±10	9.8±2.3	2.0±0.5	9.0±2.3
**42**	Benzoquinone	0	0	0	0	0
**43**	Silver nitrate	-	12±3	2.7±0.8	0.5±0.2	3.0±0.8
**44**	*tert*-Butyl alcohol	0.5 to 2	4±3	1.1±0.7	0.2±0.1	0.4±0.7
**Electron donor (EtOH)**						
**45**	EtOH (0%)	0.5 to 2	38±12	7.2±2.2	1.4±0.4	9.0±2.2
**46**	EtOH (10%)	0.5 to 2	30±11	6.6±2.2	1.3±0.4	8.3±2.2
**47**	EtOH (20%)	0.5 to 2	13±6	2.0±0.3	0.4±0.1	2.5±0.3
**48**	EtOH (50%)	0.5	16±10	3.2±1.8	0.6±0.4	4.1±1.8
**Solvents and biphasic systems**						
**49**	Milli-Q (4 mL)	0.5 to 2	28±9	6.8±3.3	1.4±0.7	9.0±3.3
**50**	Lake water	0.5	11±4	3.1±1.0	0.6±0.2	4.1±1.0
**51**	Seawater	0.5 to 2	34±3	8.6±0.8	1.7±0.2	11.4±0.8
**52**	Biphasic Heptane	0.5 to 2	88±32	15.0±1.2	3.0±1.6	18.5±1.2
**53**	Biphasic DCM	2	66±14	15.9±2.7	3.2±0.5	19.5±2.7
**Temperature**						
**54**	10°C	0.5	20±7	5.3±1.4	1.1±0.3	4.8±1.4
**55**	20°C	0.5 to 2	44±10	9.8±2.3	2.0±0.5	9.0±2.2
**56**	35-40°C	2 to 5	53±2	12.4±2.3	2.5±0.5	11.5±2.3
**Stirring bars**						
**57**	Teflon	0.5 to 2	41±3	9.4±0.4	1.9±0.1	9.0±0.4
**58**	Glass	0.5	29±6	6.7±2.1	1.3±0.4	6.4±2.1
**59**	No stirring	0.5 to 2	40±10	10.1±2.7	2.0±0.5	9.7±2.7
**Catalyst and solvent amount**						**[µmol]**
**60**	4 mg catalyst, 4 mL solvent	0.5 to 2	34±2	9.4±1.3	1.7±0.3	0.14±0.03
**61**	7 mg catalyst, 4 mL solvent	0.5 to 2	16±1	-	-	0.06±0.02
**62**	4 mg catalyst, 8 mL solvent	0.5	12±1	-	-	0.09±0.01

Entry	Condition	Peroxide Teststrip [mg L^{-1}]	Produced H_2O_2 [µmol L^{-1}]	Produced H_2O_2 [mmol g^{-1} L^{-1}]	Productivity H_2O_2 [mmol g^{-1} L^{-1} h^{-1}]	Normalized production H_2O_2 [mmol g^{-1} L^{-1}]
**Cation exchangers**						
**63**	No additive	0.5 to 2	28±4	7.9±1.0	1.6±0.2	9.0±1.0
**64**	Fe_2O_3	0	0	0	0	0
**65**	Fe_3O_4	0	0	0	0	0
**66**	$FeSO_4$	0	0	0	0	0
**67**	$FeCl_2$	0	0	0	0	0
**68**	Al_2O_3	0.5 to 2	49±16	13.6±4.3	2.7±0.9	15.5±4.3
**69**	$Zn(NO_3)_2$	0.5 to 2	21±7	4.5±1.7	0.9±0.3	5.2±1.7
**70**	$Al(NO_3)_3$	0 to 0.5	25±20	5.5±4.6	1.1±0.9	6.3±4.6
**71**	$NiCl_2$	0.5 to 2	18±2	2.7±0.3	0.5±0.1	8.3±0.3
**Salts and acids**						
**72**	No additive	0.5 to 2	35±4	8.0±0.8	1.6±0.2	9.0±0.8
**73**	NaCl	0.5 to 2	0	0	0	0
**74**	NaBr	0.5	0	0	0	0
**75**	Acetic acid	0.5 to 2	9±17	6.2±1.9	1.2±1.0	6.9±1.9

Providing productivity (i.e. production rates) and production of H_2O_2 already provides comparable results to other literature. As the AQY at 445 nm (5h) was 0.11%, this value can theoretically be multiplied with the productivity to combine production values with photonflux and its yield. This way a value is obtained ("quantum productivity"), which contains all the information for photocatalytic production of hydrogen peroxide and makes comparison to other research efficient.

$$\text{Productivity} \cdot \text{AQY} = \left(\frac{\text{Moles of hydrogen peroxide produced} * 2}{\text{Moles of photons supplied}} * 100\%\right) \cdot \text{Productivity } H_2O_2 \text{ [mmol g}^{-1}\text{ L}^{-1}\text{ h}^{-1}]$$

Table S7: Calculation of comparable metrics for the production of H_2O_2. AQY was multiplied with production rate values.

Entry	Condition	Produced H_2O_2 [µmol L^{-1}]	Produced H_2O_2 [mmol g^{-1} L^{-1}]	Productivity H_2O_2 [mmol g^{-1} L^{-1} h^{-1}]	Normalized production H_2O_2 [mmol g^{-1} L^{-1}]	Quantum productivity<br>Productivity · AQY (0.11% at 445 nm, 5h)
**1**	FeO_x NP Standard (4 mg, darkness)	0	0	0	0	**0**
**2**	FeO_x NP Standard (4 mg)	34±2	9.4±1.3	1.7±0.3	9.4±1.3	**0.19**
**3**	pH = 3	48±8	11.2±1.8	2.2±0.4	14.8±1.8	**0.24**
**4**	Biphasic DCM	66±14	15.9±2.7	3.2±0.5	19.5±2.7	**0.35**
**5**	35-40°C	53±2	12.4±2.3	2.5±0.5	11.5±2.3	**0.28**
**6**	Al_2O_3	49±16	13.6±4.3	2.7±0.9	15.5±4.3	**0.30**

7.2 Catalyst recycling

Catalyst stability was tested by recycling for multiple rounds of irradiation (**Figure S109**). After each round of irradiation, the product solution was titrated for quantification of hydrogen peroxide. The catalyst was dried *via* constant air flow, and stored in nitrogen atmosphere in DCM at 5°C. The next day the samples were prepared for another round of irradiation, until hydrogen production stagnated because of catalyst depletion/degradation. The iron oxide catalyst with oleic acid (2:1) surfactant produced hydrogen peroxide for seven consecutive rounds, after which the catalyst was depleted. Thus, the catalyst was stable and could produce hydrogen peroxide for 35h. From round seven onwards the catalyst is deactivated and production is close to zero (including error bars). Reactivation was attempted by heating the FeO_x NPs at reflux in ethanol and oleic acid (as described in **Chapter 8.3**). After reaction and workup, the particles looked drier than before and were not able to catalyze the ORR towards hydrogen peroxide.

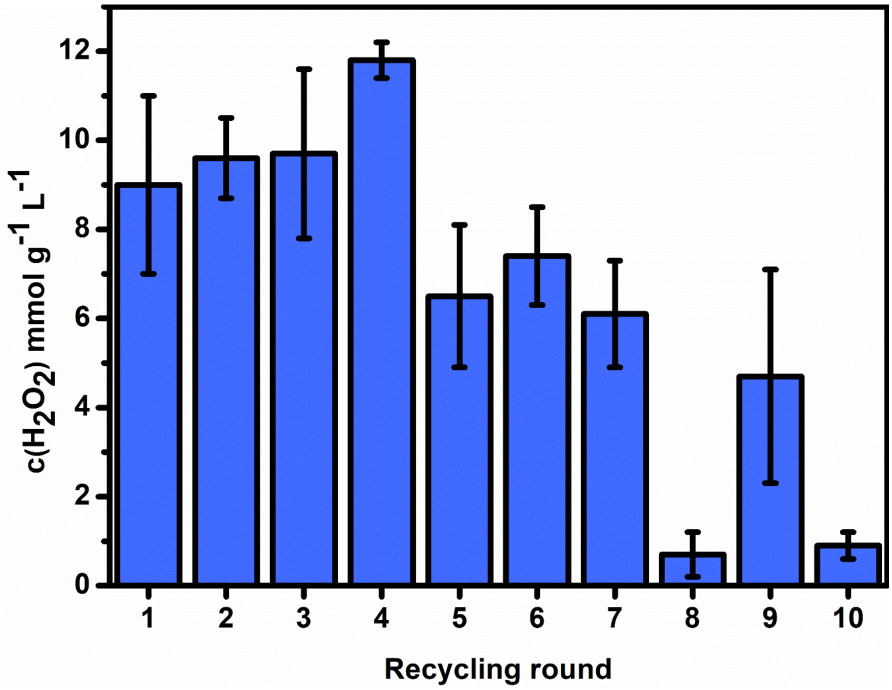

Figure S109: Recycling of iron oxide batches with oleic acid (2:1) surfactant (1 mg mL^{-1}), obtained by irradiation for 5h at 20°C repeatedly with 445 nm in new Milli-Q water every round.

7.3 Synthesis and production consistency

The optimized synthesis method in ethanol as solvent resulted in consistently active iron oxide nanoparticles with oleic acid (2:1) surfactant. In **Figure S110** production of hydrogen peroxide of several produced batches is depicted. All production values of hydrogen peroxide were found to be approximately in the same range (6.0-9.8 mmol g^{-1} L^{-1}). The upcoming screening results were largely conducted using a different batch of nanoparticles for each screening. In order to have good comparison, every batch was normalized to the production of batch 126 shown below (9.0±0.4 mmol g^{-1} L^{-1}). Also mixing of batches was found to preserve activity for several nanoparticle batches (e.g. 177 and 178). This is a huge advantage of the synthesis in ethanol as a solvent over dibutyl ether besides the sustainability aspect. Additionally, it was found that batches of FeO_x NPs (including mixing of different batches) were stable for at least six months upon storing in nitrogen atmosphere in darkness in the fridge, without loss of activity.

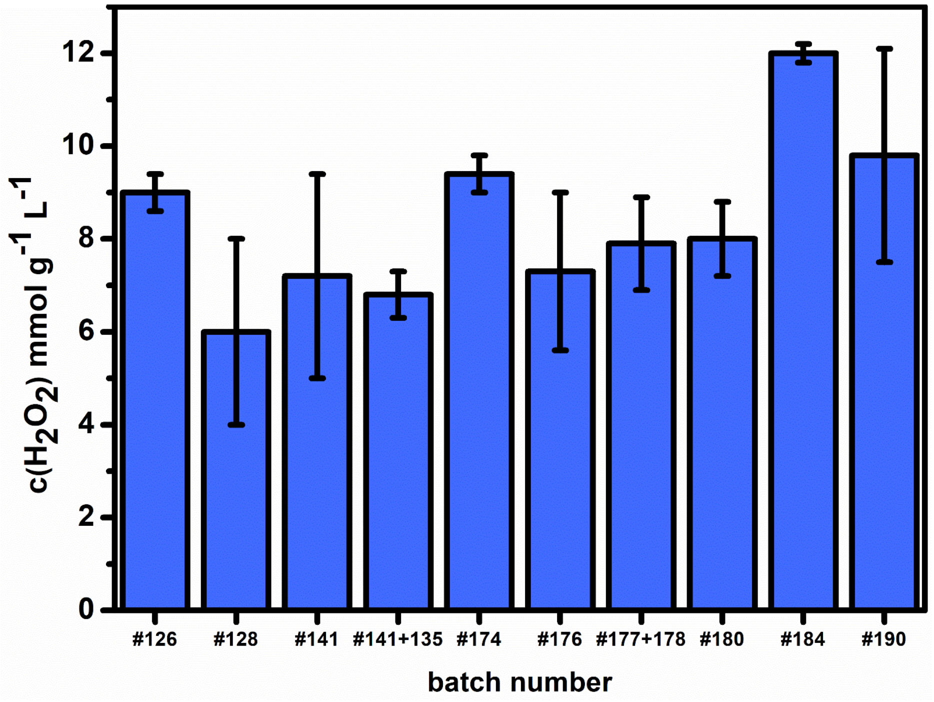

Figure S110: Production of different iron oxide batches with oleic acid (2:1) surfactant (1 mg mL^{-1}), obtained by irradiation for 5h with 445 nm in Milli-Q water.

7.4 Apparent quantum yield (AQY) measurements

The apparent quantum yield (AQY) of the nanoparticles was measured *via* UV-Vis. The catalyst was immobilized on the side of a cuvette (**Figure S111**) and 2 mL of oxygenated Milli-Q water was added. A known number of photons was subsequently shone on and through that side of the cuvette. Over time (~5h) absorption at 240 nm was followed, corresponding to formed hydrogen peroxide. Absorption kept rising upon irradiation with blue light (445 nm) and UV light (365 nm) as depicted in **Figure 3C**.

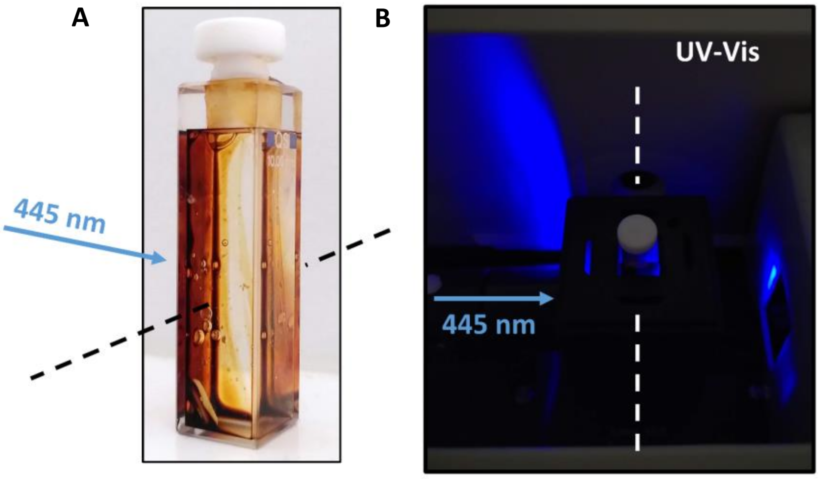

Figure S111: a) Cuvette with catalyst immobilized on the side of the glass, depicting hydrophobic oxygen affinity; b): UV-Vis irradiation setup, photonflux 365 nm: $3.70588 \cdot 10^{-5}$ mmol s^{-1}, 445 nm: $2.60028 \cdot 10^{-5}$ mmol s^{-1}.

After irradiation was stopped, the produced hydrogen peroxide solution was titrated to quantify the amount of moles produced. The UV-Vis apparatus irradiated a known number of photons over time, as the photon flux was measured *via* actinometry and correlated to the percentage of absorbance of the catalyst (i.e. catalyst concentration) at the irradiation wavelength. With these results the AQY of the nanoparticles could be calculated *via* **Eq. 1**. Here the 2 resembles the two electrons needed for the two-electron ORR pathway our nanoparticles need for hydrogen peroxide production. The calculated AQY values are depicted in **Table S8**. The absorption at 240 nm was followed over time (5h), corresponding to formed hydrogen peroxide (**Figure 3C**) with an AQY_{365} = 0.10% and AQY_{445} = 0.11%.

Equation 1: Apparent quantum yield of hydrogen peroxide.[20]

$$\mathrm{AQY} = \frac{\text{Moles of hydrogen peroxide produced} * 2}{\text{Moles of photons supplied}} * 100\% \quad (1)$$

Table S8: Apparent quantum yield results.

Irradiated wavelength (time)	Photonflux (absorbed)	AQY
365 nm (5h)	$3.70588 \cdot 10^{-5}$ mmol s^{-1} ($2.23763 \cdot 10^{-5}$ mmol s^{-1})	0.10%
445 nm (5h)	$2.60028 \cdot 10^{-5}$ mmol s^{-1} ($8.19909 \cdot 10^{-6}$ mmol s^{-1})	0.11%

7.5 Catalyst stability

Catalyst stability towards hydrogen peroxide was also evaluated (**Figure S112**). After 5h of being subjected to a 1 mM hydrogen peroxide solution (30 min nitrogenated) and light irradiation (445 nm) no effect in the absorbance spectrum was visible. Hence the photocatalytic production of hydrogen peroxide or stronger oxidizing conditions do not have an effect on the photochemical catalyst properties. This is in line with previous reports, where catalyst stability should be tested for over 24h to evaluate practical applications.[21] Catalyst recycling (**Figure S109**) showed production for over seven rounds of 5h, indicating catalyst depletion/decomposition to start happening after 35h. The catalyst material was stable for at least 6 months (including mixing of different batches) without loss of activity.

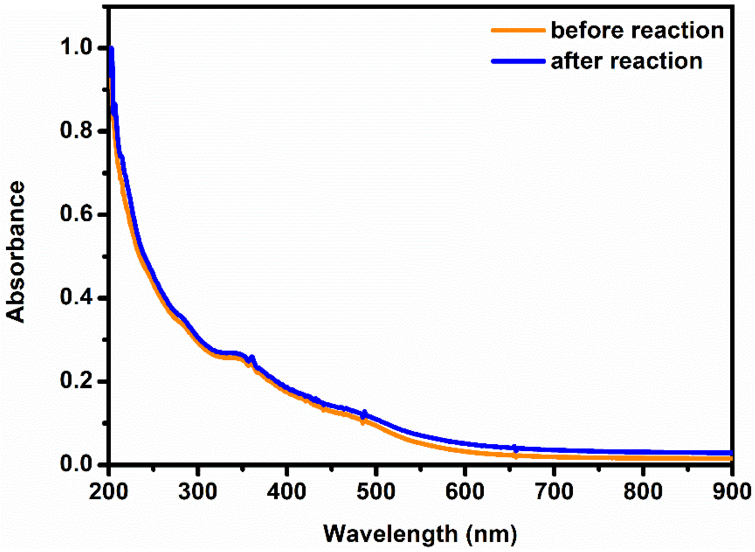

Figure S112: Catalyst stability of batch 141 after subjection to 5h of light irradiation (445 nm) in nitrogen atmosphere and a 1 mM solution (30 min nitrogenated) of hydrogen peroxide.[21]

7.6 Temperature dependency

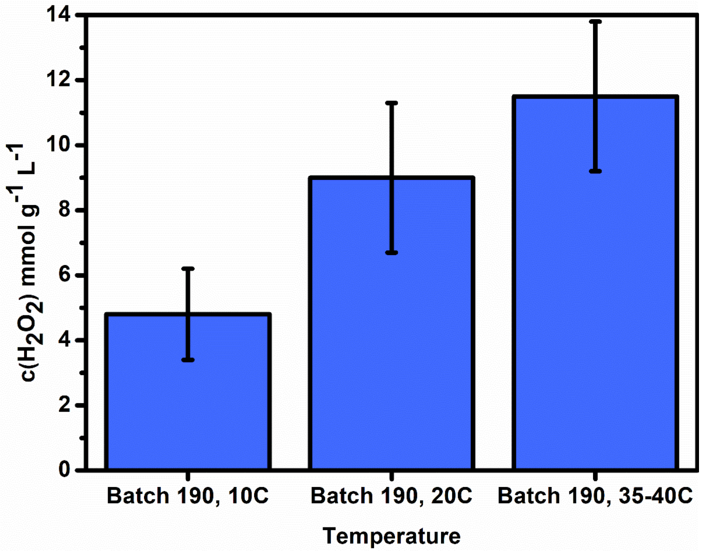

Figure S113: Screening of temperature dependency at 10°C, 20°C and 35-40°C (increasing over the course of 5h irradiation *via* heating of the LEDs). Higher temperatures lead to higher production of H_2O_2. Conditions: 5h, 445 nm, O_2 atmosphere, batch 190, FeO_x NPs (oleic acid 2:1, 1 mg mL^{-1}), 4 mL Milli-Q water in a 10 mL vial.

8. Mechanism studies

This section covers the different experiments that were performed to propose the mechanism depicted in **Figure 5B**. Iron oxide particles with oleic acid (2:1) surfactant were already found to be active for hydrogen peroxide production, therefore a broad surfactant scope (**Figure S3**) was synthesized of different surfactants to analyze their effect on activity towards hydrogen peroxide. As shown in **Figure 4A**, only oleic acid and linoleic acid particles were found to produce hydrogen peroxide photochemically. Also, immobilization on active charcoal resulted in slightly active photocatalysts, however less active than without immobilization. This lowered activity by immobilization could be explained by enhanced conductivity from the carbon support, which accelerates recombination of charge carriers. When no surfactant or oleyl alcohol were incorporated it was already found from DLS that microparticles were synthesized. These microparticles were not photoactive for hydrogen peroxide production, suggesting a role of the surfactant in the mechanism for hydrogen peroxide production as well as particle size. Nanoparticles with amines and alcohols were also found not to produce hydrogen peroxide. It is assumed that the nanoparticles with amines or alcohols as surfactant have different connectivity to the iron oxide surface than carboxylic acid (see **Figure S114**). This fact seems to be crucial for proper electron transfer to the active site. Saturated fatty acids were all found to be inactive, suggesting a direct role of the double bond(s) in the mechanism. Unfortunately, nanoparticles with stearic acid as surfactant were not possible to be synthesized, as this would have offered a direct comparison of an equally long unsaturated fatty acid compared to oleic acid (**Supporting Information 3.3**). Interestingly, elaidic acid has the exact same molecular structure as oleic acid, however its double bond is *trans* where oleic acid contains a *cis* double bond (**Figure 4A**). No differences in the UV-Vis absorption spectrum between nanoparticles with elaidic and oleic acid could be observed, indicating no differences in its photochemical behavior. However, elaidic acid nanoparticles where not able to promote photocatalytic production towards hydrogen peroxide. The active site therefore seems to consist of iron oxide connected to a carboxylic acid, which in the proximity of the *cis* double bond forms a hydrophobic pocket favorable for oxygen affinity. Here protons could possibly be supplied by (carboxylic) acids or water, while electron transfer could be possible by iron and its connectivity with the surfactant. From XRD and DLS measurements (**Table S1**) it was found that particle sizes smaller than/around 2 nm with a certain crystallinity were necessary for activity, while also TEM confirmed these small and round particles. This interplay between factors seems to be crucial for photochemical oxygen reduction activity towards hydrogen peroxide *via* FeO_x.

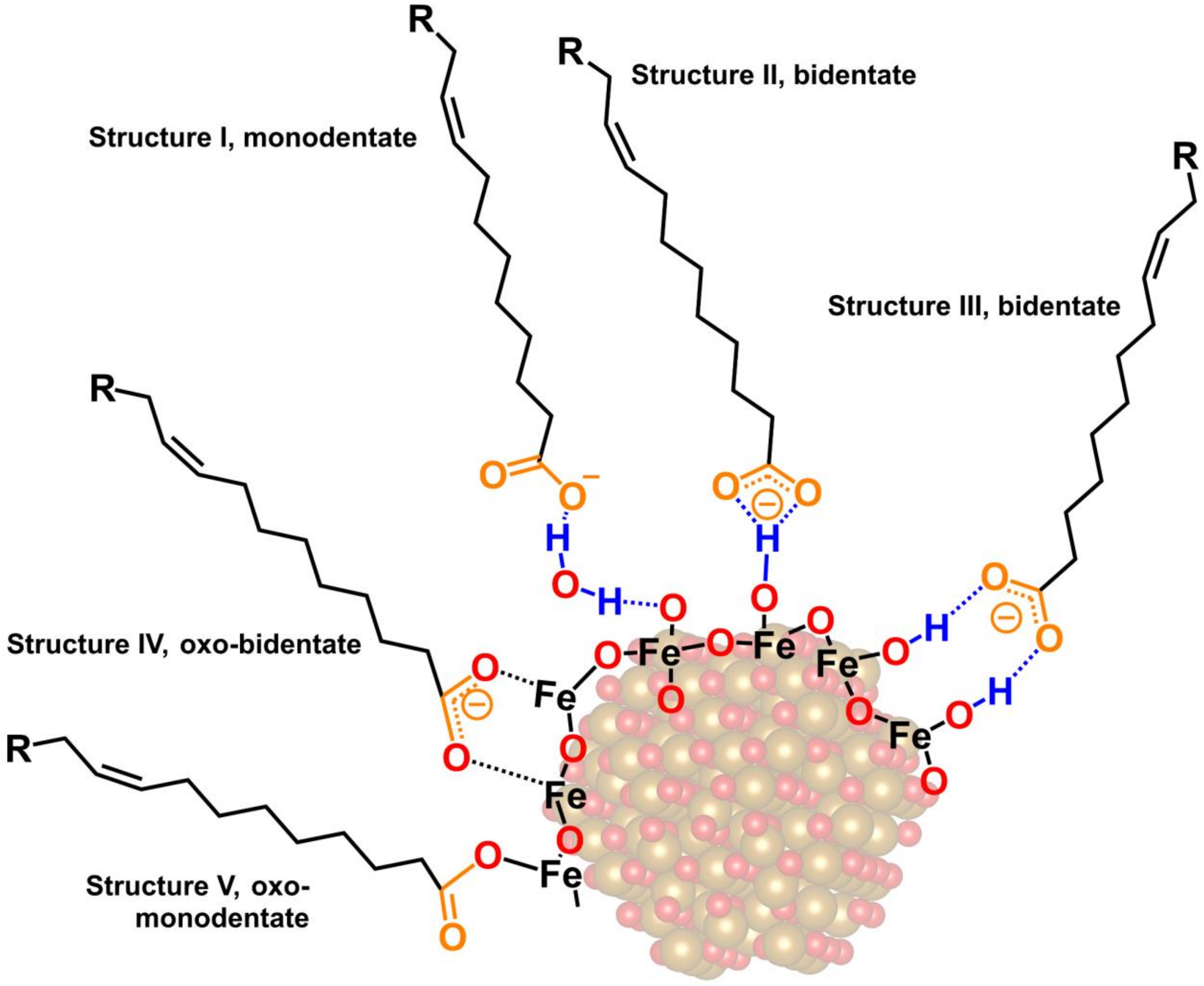

Figure S114: Different ways (un)saturated fatty acids could bind/coordinate towards the iron oxide core.

The influence of the stirring bar and remaining trace metals on its surface were examined by addition of a Teflon and glass stirring bar, as well as testing for no stirring bar. Teflon can indeed act as cocatalyst, where PTFE increased oxygen affinity to the active site.[5] **Figure S115** depicts no significant difference upon addition of no stirring bar, a Teflon or a glass stirring bar. Teflon is hydrophobic and therefore the iron oxide nanoparticles and oxygen coordinated to it, however this did not increase production as no stirring bar resulted in similar production due to the sufficient hydrophobicity of the particles themselves.

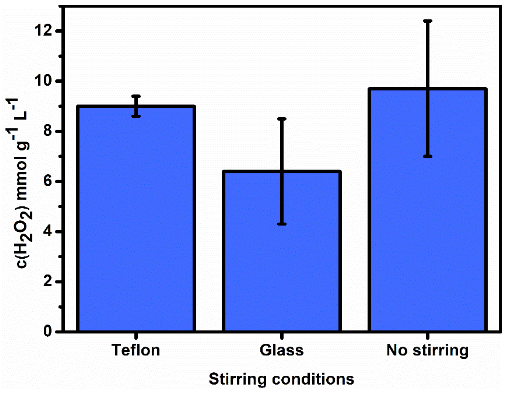

Figure S115: Effect of stirring bar on production of hydrogen peroxide. Irradiation (445 nm) for 5h at 20°C, catalyst loading 1 mg mL^{-1}.

Active species trapping experiments of superoxide radicals, electrons and hydroxyl radicals by *p*-benzoquinone, silver nitrate and *tert*-butyl alcohol (TBA) respectively were conducted (**Scheme S2**, **Scheme S3**). These experiments should always be performed to check which active intermediates are participating in the hydrogen peroxide production mechanism. In our reaction system, only when silver nitrate or *tert*-butyl alcohol were added hydrogen peroxide was still produced, however significantly less than when no scavenger was added (**Figure 4D**). Electrons and hydroxyl radicals are thus present in large amounts, as the amount of silver nitrate/TBA added were not able to scavenge them all. In general, it can be concluded from the peroxide test strips and iodometric titration (**Scheme S2A**), that less hydrogen peroxide is produced as soon as the abovementioned mechanism scavengers were added. These results indicate that superoxide radicals, electrons and hydroxyl radicals are all actively taking part in the mechanism of hydrogen peroxide production.

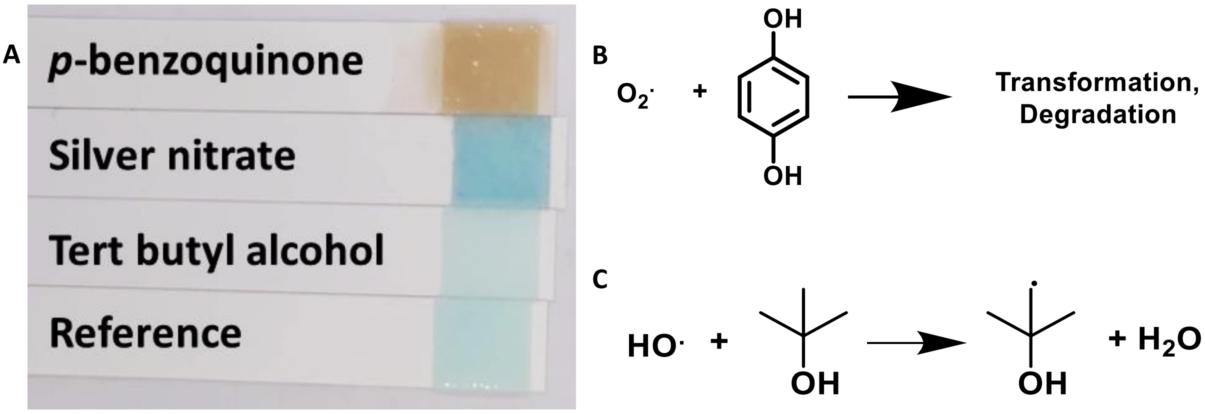

Scheme S2: a) Mechanism scavenger peroxide test strips after light irradiation (445 nm) for 5 hours. b) Mechanism of superoxide scavenging by *p*-benzoquinone.[22] c) Mechanism of hydroxyl radical scavenging by *tert*-butanol.[23]

$$NO_3^- + 2e^- + 2H^+ \rightarrow NO_2^- + H_2O \qquad (E^0 = 0.84\ V)$$

$$NO_3^- + 8e^- + 10H^+ \rightarrow NH_4^+ + 3H_2O \qquad (E^0 = 0.88\ V)$$

$$2NO^- + 10e^- + 12H^+ \rightarrow N_2 + 6H_2O \qquad (E^0 = 1.25\ V)$$

$$NO_3^- + e^- \rightarrow NO_3^{2-} \qquad (E^0 = -1.1\ V)$$

$$NO_3^- + 2e^- \rightarrow NO_3^{2-} \qquad (E^0 = 0.94\ V,\ pK_a = 3.3)$$

$$2NO_3^- + 8e^- + 10H^+ \rightarrow N_2O + 5H_2O \qquad (E^0 = 1.12\ V)$$

$$2HNO_2 \rightleftharpoons NO + NO_2 + H_2O$$

Scheme S3: Mechanism of electron scavenging by nitrate.[24]

A pH screening was conducted at pH 3, 7 and 11, as well as in phosphate buffer (pH 7.24, **Figure 4B**). Production in Milli-Q water and 1 mM NaOH resulted in similar amounts of hydrogen peroxide production, showing that basic conditions do not enhance or lower production. Phosphate buffer solutions do not change pH over time, where in Milli-Q water and 1 mM NaOH the pH of the solution decreased (**Table S9**). This pH restriction could be the reason that production of hydrogen peroxide slightly decreased, however phosphate could also interfere with/block the active site. An increased production of hydrogen peroxide production was obtained *via* acidic environment. The low pH ensures more protons in solution, which are necessary for the two electron ORR (**Scheme S*4***). The general decrease in pH during the reaction (**Table S9**) could be explained by formation of hydrogen peroxide (slightly acidic).[25]

Table S9: Changes in pH after 5h of light irradiation (445 nm).

Sample	pH before	pH after
pH 3 (light)	2.9	3
pH 3 (darkness)	2.9	3
pH 7 (light)	8.0	6.3
pH 7 (darkness)	8.0	7.6
pH 11 (light)	11.0	10.3
pH 11 (darkness)	11.0	10.5

$$O_2 + 2H^+ + 2\,e^- \longrightarrow H_2O_2 \qquad E^0 = +0.68\ V_{SHE} \qquad (2)$$

Scheme S4: Two electron ORR towards hydrogen peroxide.

8.1 Headspace GC analysis

Samples were taken from the headspace of the 10-mL pressure vial using a VICI Precision Sampling PRESSURE-LOK® syringe equipped with a push-button valve. 5-mL samples of the headspace were injected in the 50 µL sample loop of a SHIMADZU Nexis GC-2030 (Japan) gas chromatograph, equipped with two columns connected to a 6-way valve. The injected gases were first introduced over a fused silica porous polymer PLOT column (30 m × 0.32 mm i.d., film thickness 10 µm, SH-Rt-Q-BOND), with a fused silica molecular sieve PLOT column (30 m × 0.32 mm inner diameter × film thickness 10 µm, SH-Rt-Msieve 5A) positioned in series. After 2.20 min, the valve switched from the molsieve column to an empty chamber, allowing the gases retained on the polymer column to bypass the molsieve column. After 2.60 min, the valve turned back to the molsieve column, allowing the gases retained on this column to elute. A thermal conductivity detector (TCD) was used for the analysis of H_2, N_2 and O_2, and data acquisition and processing were carried out with LabSolutions Lite Software 5.93 (Shimadzu Corporation). The experiments were conducted under the following conditions: oven temperature program at 40 °C, detector temperature of 200 °C, detector current of 40 mA, carrier gas flow rate 16.0 mL/min Ar and split ratio of 1. The system was purged with 2 × 10 mL Ar prior to injection of a 5 mL sample.

Hydrogen, being the least retained species, was detected by the TCD after 3.79 min, followed by oxygen (4.23 min) and nitrogen (5.12 min) (Figures SI–A and SI–B). Hydrogen could be observed down to 0.17 ppm of the headspace, corresponding to $4.2\cdot10^{-8}$ mol (using a molar volume of 24 mol·L^{-1} at 20 °C). As observed in the chromatograms, hydrogen (H_2) was not formed under neither O_2 nor Ar atmosphere, indicating the absence of proton reduction as side reaction. As the oxygen to nitrogen ratio under Ar atmosphere remained constant, i.e. no O_2 formation, the absence of proton reduction as well as water oxidation can be concluded for the FeO_x catalyst system.

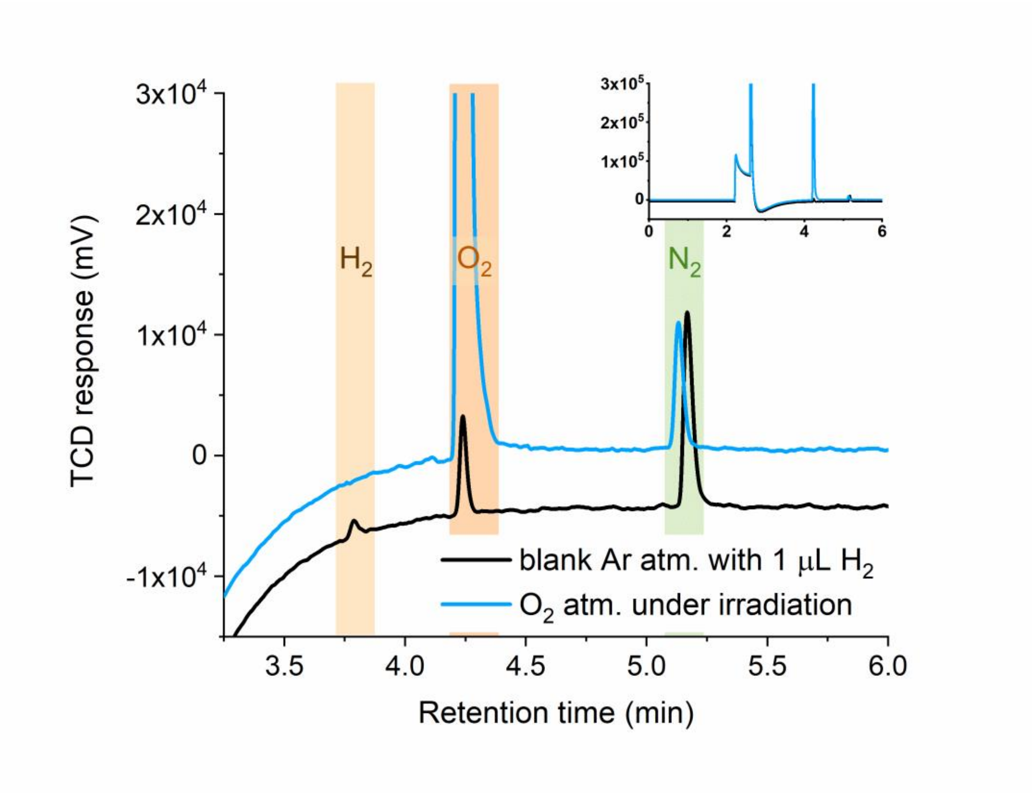

Figure S116: Typical chromatograms obtained from the headspace of a reaction under O_2 atmosphere (in blue) and from a blank Ar headspace filled with 1 µL of H_2 added (in black). The inset shows the full chromatograms.

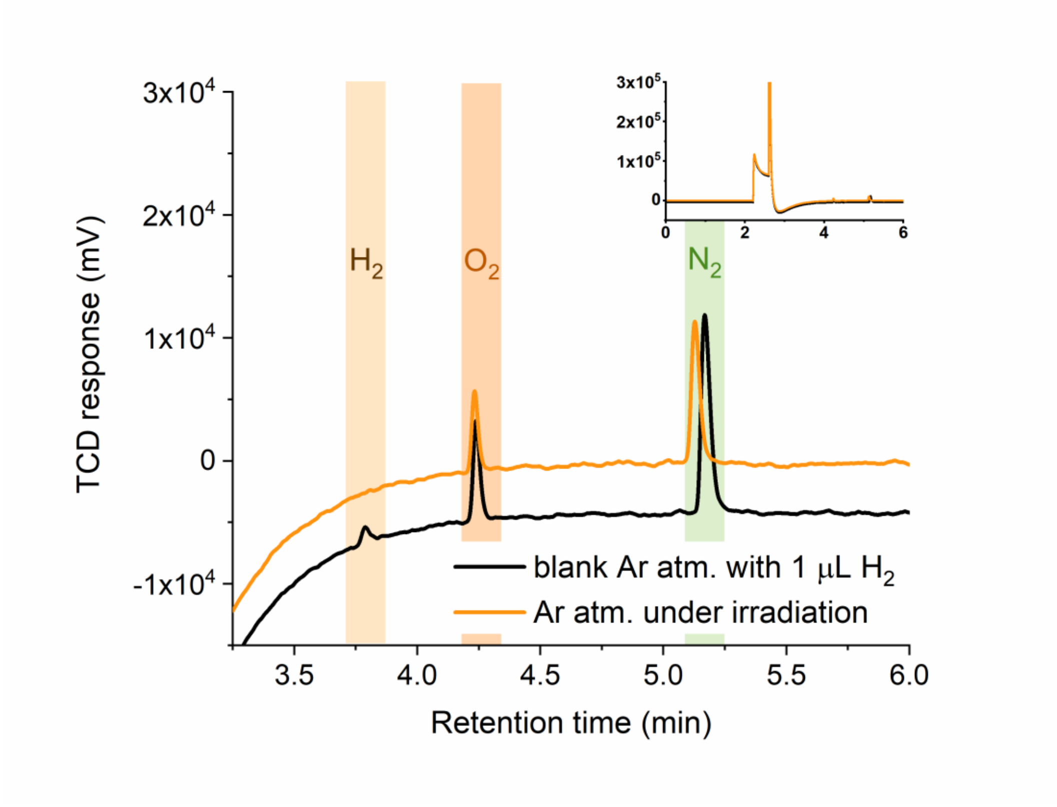

Figure S117: Typical chromatograms obtained from the headspace of a reaction under Ar atmosphere (in orange) and from a blank Ar headspace filled with 1 µL of H_2 added (in black). The inset shows the full chromatograms.

8.2 Sacrificial electron donors (hole scavengers)

In literature it is well described that the non-innocent auto-photocatalytic oxidation of benzyl alcohol to benzaldehyde produces large quantities of H_2O_2 upon irradiation.[44-47] Thus, an extensive evaluation of sacrificial agents was conducted by exposing sacrificial agents to air overnight. Confirming latest research, benzyl alcohol and isopropanol produced peroxides leading to blue peroxide test strips. Therefore, it was decided against the usage of benzyl alcohol and isopropanol, whereas only methanol and ethanol were found to be trustworthy as sacrificial agent. Methanol and ethanol did not inherently produce hydrogen peroxide in contact with oxygen by autoxidation. Ethanol and methanol were however miscible with Milli-Q water.

The addition of ethanol as sacrificial agent/electron donor at different concentration has been analyzed (**Figure 4E**). Mixtures of ethanol and Milli-Q water did not dissolve the nanoparticles, where mixtures of methanol and Milli-Q water did. In literature higher productions/productivities could be obtained by the addition of sacrificial agents through filling of excess holes, produced by a mismatch in the WOR and ORR. Since the FeO_x NPs nanoparticles were lacking the WOR, we opted for addition of an electron donor (EtOH) to improve production and mismatch. However, as depicted in **Figure 4E**, no significant increase was obtained.

8.3 Catalyst reactivation

Catalyst recycling was performed by drying the catalyst after each irradiation reaction. The dried catalyst (~4 mg) was then redissolved in DCM (0.5 mL), and stored in nitrogen atmosphere and darkness at 5°C. The method as described above could then be followed after evaporation of DCM. These catalyst recycling reactions were conducted until catalyst activity was depleted after 10 rounds of 5h.

Deactivated nanoparticles (21.6 mg) and oleic acid (7.58 mg) were added to ethanol (2 mL) in a 10 mL vial. A Teflon stirring bar was added after which the vial was closed by capping. The mixture was heated to 87°C for 1h, after which temperature was increased to 109°C for 2h. The mixture was cooled and partly soluble in ethanol as a light brown solution. Particles also stuck to the glass. Workup was conducted by magnetic precipitation from 5 mL ethanol for 1h. After decantation 2 mL of ethanol was added and the particles were placed on top a magnet for additional 15 min. The solution was decanted off and particles were dried and stored in DCM (3 mL) in nitrogen atmosphere at 5°C. The procedure described did not lead to reactivation of the iron oxide nanoparticles for hydrogen peroxide production.

8.4 XPS Analysis

X-ray photoelectron spectroscopy (XPS) was performed using a Surface Science Instruments SSX-100 ESCA spectrometer, equipped with a monochromatic Al Kα X-ray source (hν =1486.6 eV). The pressure in the measurement chamber was maintained below $5*x10^{-9}$ mbar during data acquisition. The photoelectron take-off angle was 37° with respect to the surface normal. The diameter of the analyzed area was 1000 µm; the energy resolution was 1.26 eV (or 1.67 eV for a broad survey scan). XPS spectra were analyzed using the least-squares curve fitting program Winspec developed at the LISE, University of Namur, Belgium and included a Shirley baseline subtraction and a peak deconvolution using a linear combination of Gaussian and Lorentzian functions, taking into account the experimental resolution. The spectra were fitted with a minimum number of peaks consistent with the structure of the surface. Binding energies of isolated peaks are given ± 0.05 eV; when more than one component was needed to reproduce the raw data, the error in peak position was ± 0.1 eV. Binding energies were referenced to the C 1s photoemission peak originating from adventitious carbon (C-C/C=C) at a binding energy of 284.8 eV. FeO_x NPs with oleic acid surfactant (before and after 8 cycles of catalysis) or FeO_x reference NPs without surfactant were dispersed in dichloromethane (DCM) and drop-casted onto a 200 nm thick thermally evaporated gold (99.99% granulate, Umicore) layer on mica substrates.[26] Charge neutralization was achieved with a flood gun set to 0 eV and a gold grid placed 3 mm above the sample. Oleic acid was found to be instable for measurements longer than 2 hours. Therefore, measurements were taken at up to 6 different sample spots and summed up to achieve a sufficient signal to noise ratio.

Measurements of Fe 2p core levels of the FeO_x reference, the FeO_x NPs and the FeO_x NPs after catalysis can be found in **Figure S118**. While the signal of the reference and nanoparticle look very alike with only variations of noise level due to the oleic acid coverage, the samples after catalysis show a wider spectrum towards 714 eV. The Fe 2p 3/2 part of the doublet was fit with 3 contributions at 712 eV, 714 eV and 719 eV, that can be attributed to Fe^{2+}, Fe^{3+} and an Fe^{2+} satellite, respectively.[27,28] The results indicate a higher amount of Fe^{3+} and hence oxidization after catalysis. Area ratios deducted from the fit are shown in **Table S10**.

Figure S119 depicts O 1s core level spectra of the 3 sample types. While the FeO_x reference shows a pronounced peak at 530.3 eV, the other FeO_x NPs show a peak maximum at 532.3 eV. A pronounced shoulder around 534 eV and a higher O 1s intensity can be observed after catalysis. O 1s spectra were fit with 3 contributions at 530.5 eV, 532 eV and 533.5 eV, which can be attributed to O-Fe, O-C / O=C and O-C=O species, respectively.[29,30] The total intensity of the O-Fe peak was not found to change significantly after catalysis but the increase in total O 1s intensity and the higher intense O-C=O species after catalysis indicate an oxidization of the oleic acid surfactant.

Measurements of C 1s core levels are given in **Figure S120**. Spectra were fit with tree peaks at 284.8 eV, 286.2 eV and 288.5 eV which can be assigned to the aliphatic C, C-O / C-OH / C=O bonds and bidentate carboxylate carbon (O-C=O).[31,32] After catalysis a significant decrease in aliphatic C is observed, while the C-O / C-OH / C=O peak shows an increased intensity, indicating an oxidization of the aliphatic C.

Elemental contributions and fit parameters are given in **Table S10**.

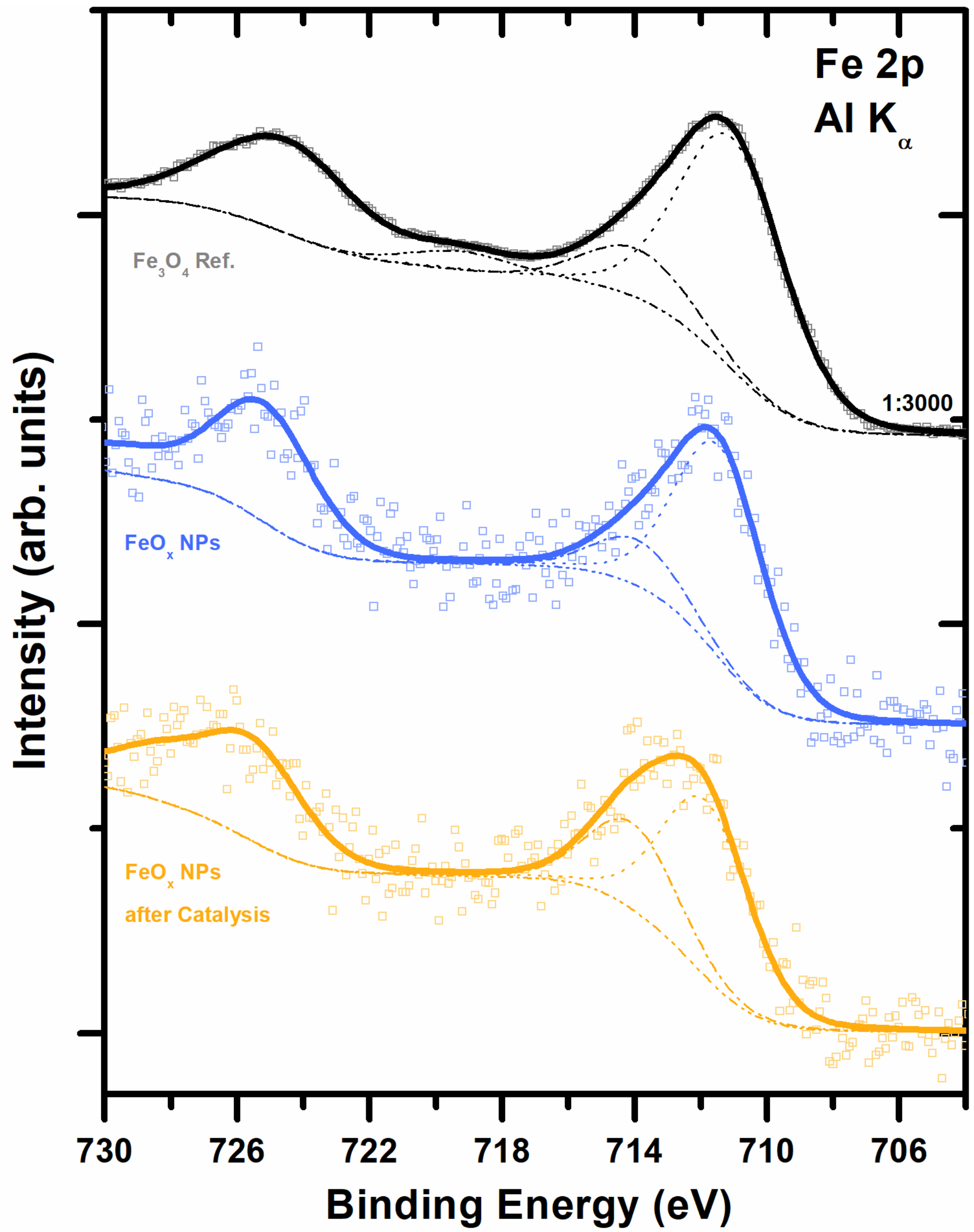

Figure S118: Fe 2p XPS region for the as-synthesized nanoparticles, black: FeO_x without surfactant, blue: FeO_x NPs before photochemical production of H_2O_2, orange: inactive FeO_x NPs after 8 consecutive rounds of catalysis. Conditions: Batch 184, FeO_x NPs with oleic acid surfactant (2:1), 5h of irradiation at 445 nm for 8 consecutive rounds, O_2 atmosphere, 1 mg mL^{-1} of catalyst in 4 mL of Milli-Q water in a 10 mL vial.

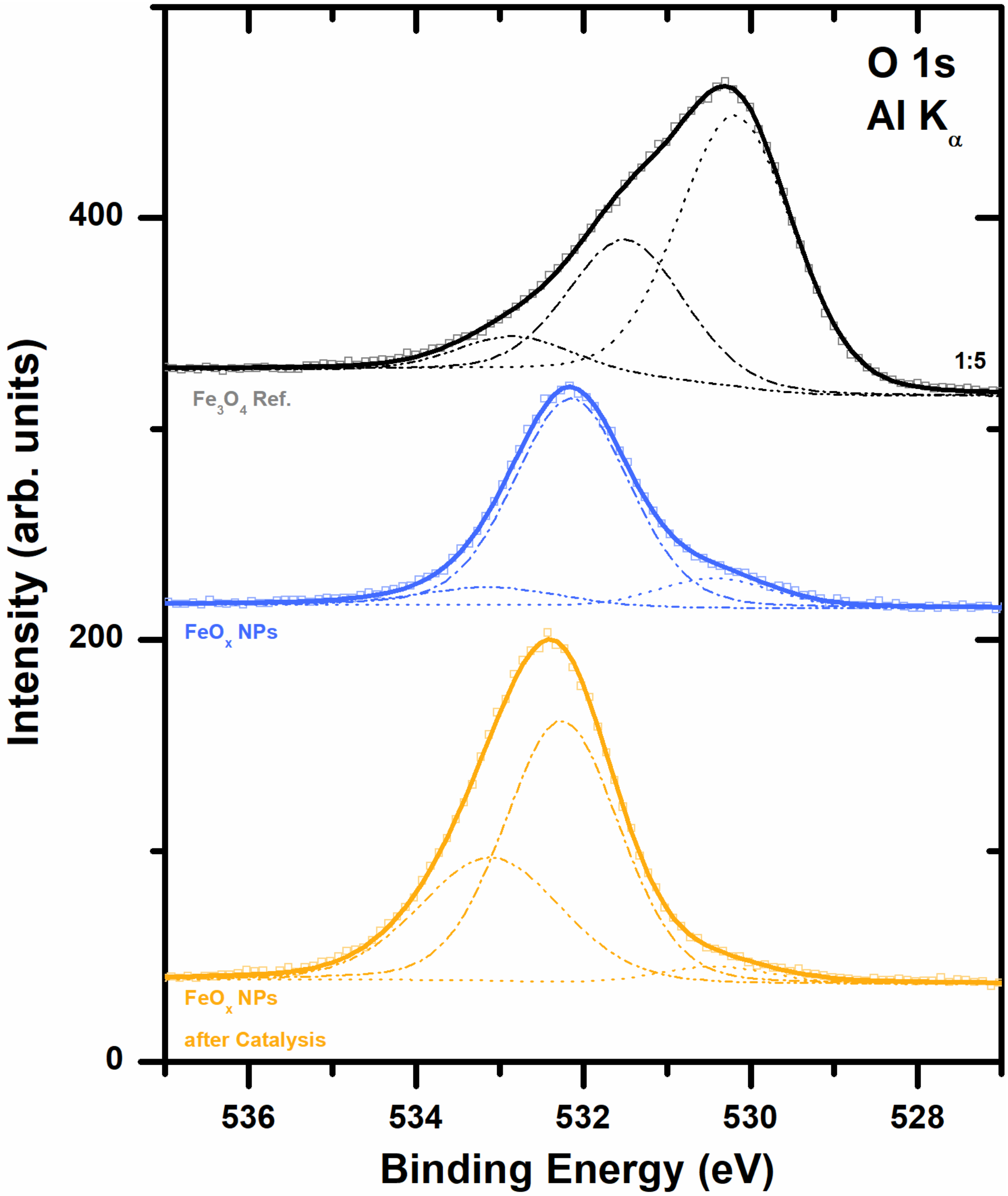

Figure S119: O 1s XPS region for the as-synthesized nanoparticles, black: FeO_x without surfactant, blue: FeO_x NPs before photochemical production of H_2O_2, orange: inactive FeO_x NPs after 8 consecutive rounds of catalysis. Conditions: Batch 184, FeO_x NPs with oleic acid surfactant (2:1), 5h of irradiation at 445 nm for 8 consecutive rounds, O_2 atmosphere, 1 mg mL^{-1} of catalyst in 4 mL of Milli-Q water in a 10 mL vial.

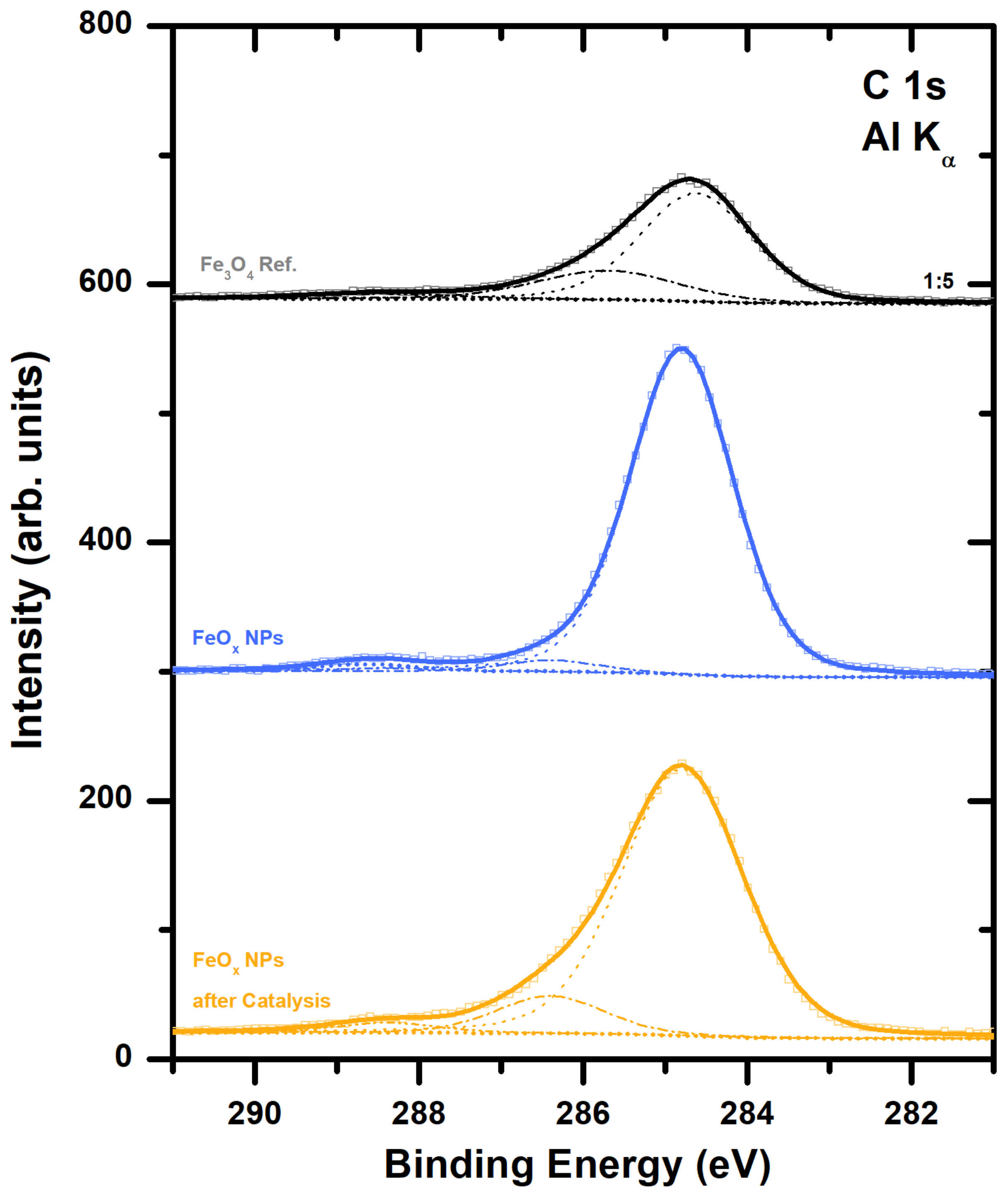

Figure S120: C 1s XPS region for the as-synthesized nanoparticles, black: FeO_x without surfactant, blue: FeO_x NPs before photochemical production of H_2O_2, orange: inactive FeO_x NPs after 8 consecutive rounds of catalysis. Conditions: Batch 184, FeO_x NPs with oleic acid surfactant (2:1), 5h of irradiation at 445 nm for 8 consecutive rounds, O_2 atmosphere, 1 mg mL^{-1} of catalyst in 4 mL of Milli-Q water in a 10 mL vial.

Table S10: XPS analysis results

Elemental contribution	184 Before	184 After	FeO_x / Fe_3O_4 Ref
Carbon	79.7%	71.5%	52.0%
Oxygen	19.8%	27.8%	37.4%
Iron	0.5%	0.7%	10.6%
**Fe 2p fit values**			
Fe^{2+} (711 eV)	83%	54%	83%
Fe^{3+} (714 eV)	17%	46%	17%
**C 1s fit values**			
C-C / C=C / C-H (284.8 eV)	94%	86%	72%
C-O / C-OH / C=O (287 eV)	3%	11%	24%
O-C=O (288 eV)	1%	3%	4%
**O 1s fit values**			
O-Fe (530 eV)	9%	1%	61%
O-C, O=C (531 eV)	91%	81%	31%
O-C=O (533 eV)	0%	17%	8%

The XPS analysis reveals that the electrons for the photochemical oxygen reduction are provided by the FeO_x catalyst material, as depicted in **Table S10**. Fe^{2+} (711.7 eV) is oxidized to Fe^{3+} (714.3 eV) over time, transforming the Fe_3O_4 (709.9 eV) resembling material into Fe_2O_3 (711.1 eV) over time (general shift of the spectra towards higher energies, **Figure S118**).[27,29] These observations can also be observed by loss of magnetism over the consecutive rounds of catalyst deactivation. The *cis* double bond, playing a role as cocatalyst due to its hydrophobic pocket formation and thus oxygen affinity, is also oxidized over time (286.5 eV, **Figure S120**). The general oxygen ratio is increasing due to oxidative conditions (more oxygen and again general shift to higher energies, **Table S10**, **Figure S119**). Thus, the catalyst becomes inactive after seven rounds of catalysis i.e. ORR, as holes generated are not filled by WOR or sacrificial agents. Catalyst reactivation by attempting to reattach new surfactant was unsuccessful (**ESI 8.3**). Future research will focus on implementation as photoelectrodic material to achieve not only higher oxygen reduction rates towards H_2O_2 but also avoid catalyst oxidation.[33,34]

8.5 DFT calculations

Computational methods.

All computational input files were prepared in GaussView 6.0 on a local Windows 10 terminal. Input files were then transferred to the Rijksuniversiteit Groningen Peregrine HPC cluster where DFT or TD-DFT calculations were carried out using the Gaussian 16 (g16) suite of programs.

The DFT thermochemistry of various fatty acid oxidation processes were examined at the MN15/Def2TZVP/SMD=H_2O level. Geometry optimization of the fatty acids (in solution, not bound to FeO_x) was done using the g16 opt command at the MN15 functional and Def2TZVP basis set level of theory with implicit solvation using the Solvation Model based on Density (SMD = water).[35-37] After optimization, frequency DFT calculations of all obtained optimized structures were carried out using the g16 freq command at the MN15/Def2TZVP/SMD= H_2O level, to confirm that minima structures had zero imaginary frequencies and that transition states had a single imaginary frequency. All shown free enthalpy differences (ΔH) are ZPE and thermally corrected and were obtained from the frequency calculations and are reported in kcal/mol, at 298.15 K and 1 atm.

8.6 Proposed mechanism

DFT calculations and all beforementioned results lead us to the proposed mechanism depicted in **Figure 5B**, which exhibits similar characteristics as the peroxidase and cyclooxygenase reaction of arachidonic acid (AA, *cis*) to Prostaglandin G_2.[38-40] These findings allow for more insights into the role of the surfactant as cocatalyst in contrast to the earlier proposed mechanism.[41]

It was found by performing the photocatalytic reaction in nitrogen that the WOR was missing, therefore the described nanoparticles produce hydrogen peroxide from oxygen. From active species trapping experiments with silver nitrate, it was known that electrons are present in the mixture, and actively participating in the mechanism. These electrons are formed by photoexcitation of the photocatalyst. Excitation of oxygen with electrons yields superoxides, also confirmed by active species trapping experiments with *p*-benzoquinone. The superoxide is able to attack the surfactant at the double bond forming first an allylic radical, followed by trapping of this allylic radical with molecular triplet oxygen to form an allylic peroxyl radical. A i) subsequent single electron transfer (SET) event with either the FeO_x core or with the solvated electrons; or ii) subsequent hydrogen abstraction reaction of this peroxyl radical with water, both yield the hydroperoxide (confirmed by LC-MS and GC-MS) and hydroxyl radicals whose presence was confirmed by active species trapping with *tert*-butyl alcohol.

The final cleavage of the hydroperoxide from the surfactant and regeneration of the catalytically active fatty acid allyl radical is also though to proceed *via* either i) SET event; or ii) hydrogen abstraction from the water solvent. Direct radical scission of the hydroperoxide to regenerate the fatty acid allyl radical was calculated to be energetically unfavorable (>30 kcal/mol).

Overall, the active site of this heterogeneous catalyst seems to consist of iron oxide connected to a carboxylic acid, which in the proximity of the *cis* double bond forms a hydrophobic pocket favorable for oxygen affinity (**Figure 5B insert**). Here protons could possibly be supplied by (carboxylic) acids or water, while electron transfer could be possible by iron and its connectivity with the surfactant. The presence of iron was not taken into account for the performed DFT calculations, but is crucial for the catalytic cycle proposed in **Figure 5B**. The presence of the active pocket containing iron, carboxylic acid, *cis* double bond, linked with particle size < 2 nm (facilitating exciton transfer and quantum dot behavior), amorphous and round nanoparticles are crucial to photocatalytically produce hydrogen peroxide.

9. Additives (cation exchangers, salts, acids, biphasic systems)

The addition of aluminum oxide resulted in significantly increased production. Aluminum(III) thus facilitated the ORR at the catalyst surface, probably by facilitating enhanced electron transfer from iron core to the surface to oxygen. Hereby reducing the recombination of charge carriers in the photocatalyst.

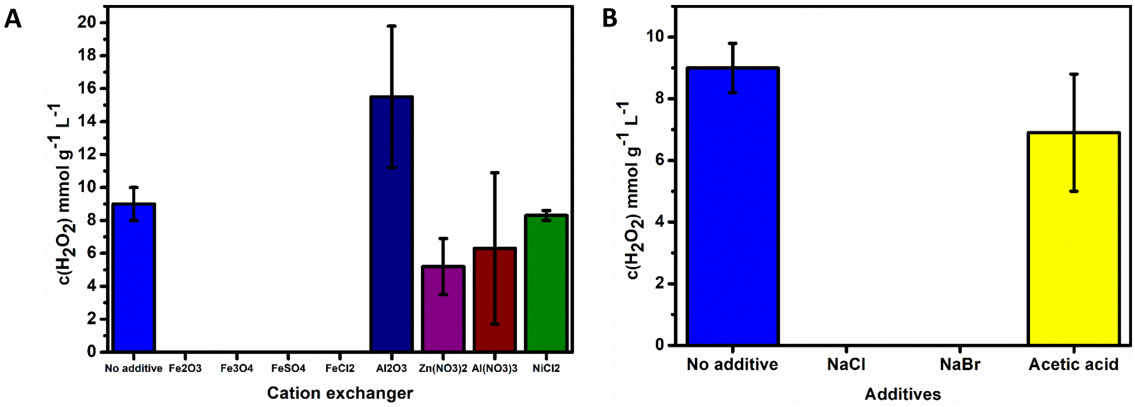

Figure S121: a) Cation exchangers screening at catalyst loading of 1 mg mL^{-1} irradiated for 5h (445 nm) at 20°C; additives added to obtain 40 mmol L^{-1}. b) Salts and acids screening at catalyst loading of 1 mg mL^{-1} irradiated for 5h (445 nm) at 20°C; additives added to obtain 40 mmol L^{-1}, Acetic acid 60 mmol L^{-1}.

Addition of acetic acid reduced production, where it was found that lowered pH by HCl resulted in significant enhancement (**Figure S121B**). This was explained by dissolving the nanoparticles through organic acetic acid addition, whereas aq. HCl solutions did not dissolve the nanoparticles. Dissolved nanoparticles presumably increased Fenton decomposition by enhanced mass transfer between produced hydrogen peroxide and iron. Additionally, the dissolved nanoparticles interfered with iodometric titration by redox reaction between iron ions and potassium iodide (**Reaction S5**).

9.1 Solvents and biphasic systems

Heptane and DCM were chosen for biphasic systems as these were not able to undergo autoxidation and are immiscible with Milli-Q water. As depicted in **Figure S122**, heptane forms a biphasic layer on top of, while DCM forms a biphasic layer below Milli-Q water. DCM was not oxygenated, yet still able to improve production. Significantly higher production was achieved (18.5±1.2 mmol g^{-1} L^{-1} for heptane, 19.5±2.7 mmol g^{-1} L^{-1} for DCM), the reason being circumvention of Fenton degradation trough separation of produced H_2O_2 and $^{\cdot}O_2^{-}$ from the catalyst surface.

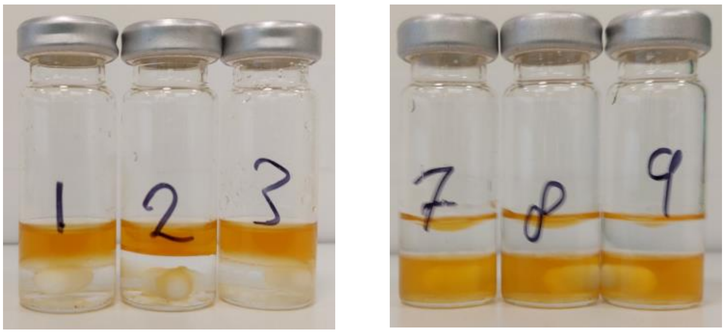

Figure S122: Biphasic system of FeO_x NPs (4 mg, batch 180) in 2 mL heptane and 2 mL Milli-Q water (left). Biphasic system of FeO_x NPs (4 mg, batch 180) in 2 mL DCM and 2 mL Milli-Q water (right).

10. Applications

The iron oxide nanoparticles were found to be able to photochemically produce hydrogen peroxide *via* ORR. The performance of the nanoparticles was extensively tested and reported in **previous sections**. Besides its ability to produce hydrogen peroxide also other applications were tested like wastewater treatment, polymerizations and *in-situ* oxidations. *In-situ* oxidation of *o*-tolidine was also attempted as a quantification method *via* ^{1}H-NMR. Production however could not be observed *via* ^{1}H-NMR as too less hydrogen peroxide was produced, not reaching the detection limit (**Figure S107**).

10.1 Styrene polymerization and oxidation

Styrene is normally polymerized *via* free radical polymerization techniques initiated by addition of a radical initiator (e.g. benzoyloxy peroxide).[42] The O-O bond is cleaved at elevated temperatures when a peroxide initiator is used to form radicals for initiation (**Scheme S5**). *In-situ* polymerization of styrene (0.4 g, distilled) in methanol (2 mL) with FeO_x NPs (4 mg) was possible after three days of irradiation by blue light (445 nm, **Scheme 1A**) as indicated by the formation of a polystyrene solid in the reaction vial (**Scheme S5, image**). This observation shows that hydrogen peroxide was formed and subsequently decomposed *via* photo-Fenton reactions towards radical species. These formed radical species were ultimately able to polymerize styrene. The polymerization reaction was slow as polymer was only observed after three days. Oxygen present in the vial is known to inhibit free radical polymerizations by reaction with active radicals.[43] Therefore, reason for the slow reaction could be explained by the necessity for oxygen to be depleted first. Besides styrene polymer also other products were found in the liquid phase (MeOH). These included unreacted styrene, benzaldehyde, styrene oxide, benzene acetaldehyde and a dimethyl acetal as confirmed by GC-MS. Interestingly, there was no polymer formed when the same reaction was conducted in ethyl acetate (**Scheme S6**). Moreover, almost no styrene reacted during these 60h as 92% of styrene was still unreacted. This indicates a direct role of the solvent in the polymerization and oxidation products of styrene.

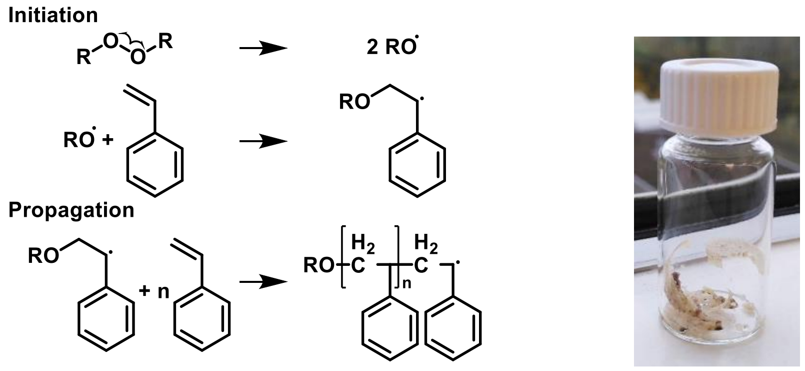

Scheme S5: Initiation by peroxide initiator and propagation of styrene polymerization (left). Polymerized styrene, initiated by hydroxyl radicals formed *via* Fenton degradation on FeO_x NPs (right).

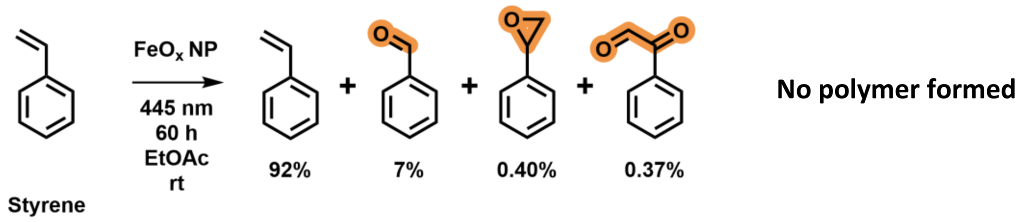

Scheme S6: Oxidation of styrene (0.4 g, distilled) in ethyl acetate (2 mL) after 60h of light irradiation (445 nm) in the presence of FeO_x NPs (4 mg).

10.1.1 Experimental procedure styrene oxidation

Styrene (0.4 g, distilled) and iron oxide nanoparticles (4 mg) were added to pre-oxygenated (30 min) methanol (2 mL) in a 10 mL vial. A Teflon stirring bar was added after which the vial was closed by capping. Finally, 5 min of oxygen bubbling through the solution was conducted to ensure an oxygen atmosphere in the vial. After 60h of irradiation (445 nm) a solid had formed (**Scheme S5, image**). Both solid and liquid were analyzed *via* GC-MS.

Styrene (0.4 g, distilled) and iron oxide nanoparticles (4 mg) were added to pre-oxygenated (30 min) ethyl acetate (2 mL) in a 10 mL vial. A Teflon stirring bar was added after which the vial was closed by capping. Finally, 5 min of oxygen bubbling through the solution was conducted to ensure an oxygen atmosphere in the vial. After 60h of irradiation (445 nm) the liquid was analyzed *via* GC-MS (**Scheme S6**).

10.2 Furfural – nucleophilic addition

A nucleophilic addition on furfural (30 µL, 362 µmol) was conducted in oxygenated methanol (4 mL) by irradiation with 445 nm light catalyzed by FeO_x NPs (4 mg) (**Scheme 1B**). After 60h quantitative production of the dimethyl acetal of furfural was observed *via* ^{1}H-NMR (**Figure S124**). This reaction confirmed the absence of singlet oxygen in the mechanism as this would lead to the formation of hydroxybutenolide *via* [4+2] cycloaddition.[16] 17045 mmol g^{-1} L^{-1} of dimethyl acetal was formed, which compared to previously investigated hydrogen peroxide production (5h, 9.4±1.3 mmol g^{-1} L^{-1}) is a 1812x increase concentration wise. Performing *in-situ* oxidation reactions thus results in higher yields through circumvention of Fenton-decomposition by direct reaction with the organic substrate.

10.2.1 Experimental procedure furfural oxidation

Furfural (30 µL, 362 µmol) and iron oxide nanoparticles (4 mg) were added to pre-oxygenated (30 min) methanol (4 mL) in a 10 mL vial (**Figure S*97***). A Teflon stirring bar was added after which the vial was closed by capping. Finally, 5 min of oxygen bubbling through the solution was conducted to ensure an oxygen atmosphere in the vial. After 60h of irradiation (445 nm) quantitative production of the dimethyl acetal of furfural was observed *via* ^{1}H-NMR (**Figure S124**).

^{1}H-NMR (400 MHz, $CDCl_3$) δ 8.00 (d, J = 7.3 Hz, 1H), 7.40 – 7.30 (m, 1H), 6.31 (dd, J = 21.0, 3.0 Hz, 2H), 3.53 (s, 6H).

10.3 α-terpinene oxidation

An *in-situ* oxidation reaction of α-terpinene (0.15 mL) in oxygenated isopropanol (5 mL) was catalyzed by FeO_x NPs (5 mg) *via* irradiation with 445 nm light. This reaction resulted in a number of products as depicted in **Scheme 1C**. Also here, no singlet oxygen was formed, as there were no [4+2] cycloaddition products obtained.[15] After reaction 65% of initial α-terpinene was still present. The products of the *in-situ* oxidations catalyzed by FeO_x NPs consisted of epoxides, ketones, ethers and alcohols. In total 7377 mmol g^{-1} L^{-1} of products were formed, which compared to previously investigated hydrogen peroxide production (5h, 9.4±1.3 mmol g^{-1} L^{-1}) is a 784x increase concentration wise.

10.3.1 Experimental procedure α-terpinene oxidation

α-terpinene (0.15 mL, 85%) and iron oxide nanoparticles (5 mg) were added to pre-oxygenated (30 min) isopropanol (5 mL) in a 10 mL vial. A Teflon stirring bar was added after which the vial was closed by capping. Finally, 5 min of oxygen bubbling through the solution was conducted to ensure an oxygen atmosphere in the vial. After 60h of irradiation (445 nm) the reaction mixture was analyzed *via* GC-MS (**Scheme 1C**).

10.4 Wastewater treatment for degradation of methylene blue (MB)

The ability to degrade methylene blue (20 mg L^{-1}) by the FeO_x NPs was tested by irradiation of prepared samples (4 mL) with 445 nm light for ~25h. Results of these reactions and its blanks (without addition of nanoparticles) are depicted in **Figure S123**. Here every vial on the left was irradiated while the one on the right was not (blank in darkness). After ~25h methylene blue solutions were found to be partly photobleached (**Figure S123 A**) without any additives. Similarly, methylene blue solutions with hydrogen peroxide (17.5 mM) added (**Figure S123 B**) and methylene blue solutions with FeO_x NPs (1 mg mL^{-1}) added (**Figure S123 C**) also showed this photobleaching effect. Addition of both FeO_x NPs (1 mg mL^{-1}) and hydrogen peroxide (17.5 mM) to methylene blue solutions (20 mg L^{-1}) induced photo-Fenton decomposition of H_2O_2 towards hydroxyl radicals. These hydroxyl radicals were subsequently able to degrade methylene blue towards a colorless solution (**Figure S123 D)**. The as synthesized FeO_x NPs were thus successful for wastewater treatment applications. These experiments however also give rise to possible inability of the nanoparticles to produce (enough) hydrogen peroxide for methylene blue degradation at this concentration (20 mg) without further addition of H_2O_2, leaving space for further optimization.

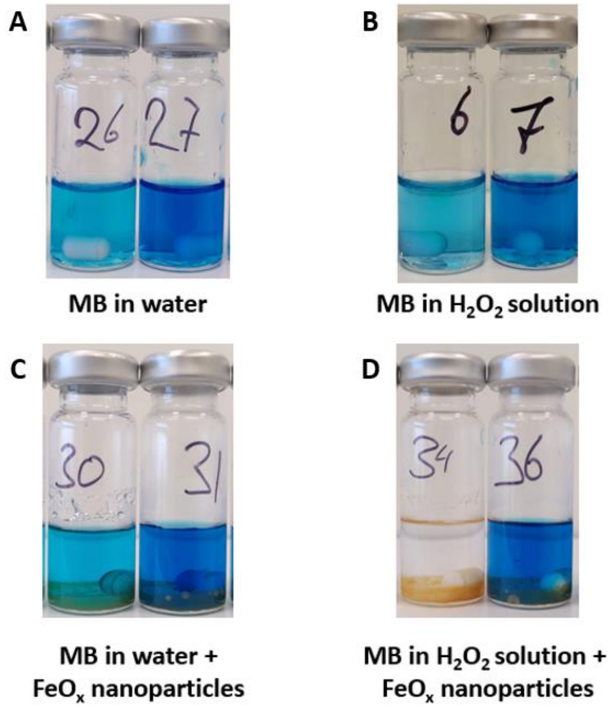

Figure S123: Wastewater treatment application experiments. Conditions: irradiated by 445 nm light for ~25h: [MB] = 20 mg L^{-1}; [H_2O_2] = 17.5 mM; FeO_x NPs, batch 157, 1 mg mL^{-1}. From **a-d** each left vial was irradiated, while the right vial depicts the blank in darkness.

10.5.1 Experimental procedure wastewater treatment

Milli-Q water was pre-oxygenated for 30 min. An aqueous solution was then prepared with methylene blue (20 mg L^{-1}) and hydrogen peroxide (17.5 mmol L^{-1}). To some samples 4 mg of FeO_x NPs was added after evaporation of DCM. A Teflon stirring bar was added after which the vial was closed by capping. After capping no oxygen bubbling was conducted. The samples were irradiated (445 nm) for 25h at 20°C (**Figure S94**).

11. DFT Data

Oleic Acid optimized geometry [# opt freq scrf=(smd,solvent=water) def2tzvp mn15]

EE + Thermal Free Energy Correction: -855.667418 Ha

0 1

C	7.62350900	-2.14679000	0.14124400
O	7.58941300	-2.37144600	1.33491400
O	8.59498800	-2.63383000	-0.64154300
H	9.21205100	-3.15575300	-0.09808200
C	6.63012400	-1.32502200	-0.61675100
H	7.18764200	-0.54612400	-1.14515500
H	6.20416900	-1.96756800	-1.39333100
C	5.54588800	-0.72709700	0.25935300
H	5.00739300	-1.52818100	0.77428800
H	6.00517500	-0.10878600	1.03651100
C	4.56811200	0.11194200	-0.54857300
H	5.11626400	0.90287800	-1.07362200
H	4.10645400	-0.51111100	-1.32331500
C	3.48231200	0.73830800	0.31227900
H	2.93095200	-0.05224200	0.83473400
H	3.94844800	1.35540100	1.08952700
C	2.50935000	1.58959400	-0.48873300
H	3.06425900	2.37269800	-1.01907200
H	2.03384800	0.97190500	-1.25974300
C	1.43663000	2.23527200	0.37410300
H	0.87326900	1.45889300	0.90421100
H	1.91200800	2.85421700	1.14382500
C	0.46872500	3.09524100	-0.43719800
H	1.05399300	3.83868000	-0.99131000
H	-0.03449400	2.47286900	-1.18062600
C	-0.51639000	3.80069300	0.44215400
C	-1.84418700	3.67139600	0.45182100
H	-0.07085200	4.47798600	1.16891700
H	-2.40129100	4.24974200	1.18694200
C	-2.68601000	2.78650100	-0.41407900
H	-3.43645100	3.39656600	-0.93041100
H	-2.08792900	2.30096000	-1.18854100
C	-3.41599400	1.72279200	0.40483100
H	-3.98437000	2.20960100	1.20578700
H	-2.67877100	1.07713700	0.89567300
C	-4.35502400	0.87565100	-0.43949800
H	-3.78665600	0.39562500	-1.24500400
H	-5.08956900	1.52822700	-0.92639800
C	-5.08280500	-0.18818700	0.36788500

H	-5.64437600	0.29305100	1.17759400
H	-4.34813900	-0.84421000	0.84990500
C	-6.03270600	-1.02779800	-0.47256900
H	-5.47175500	-1.50910900	-1.28270600
H	-6.76683200	-0.37077000	-0.95431600
C	-6.76253800	-2.09040800	0.33435400
H	-7.32149100	-1.60972100	1.14649500
H	-6.02948900	-2.75020700	0.81427500
C	-7.71704800	-2.92723800	-0.50432600
H	-7.15672200	-3.40554200	-1.31450400
H	-8.44698800	-2.26571800	-0.98258100
C	-8.43746300	-3.98273200	0.31975000
H	-9.01981900	-3.51834300	1.11955600
H	-9.12008500	-4.57857700	-0.28849000
H	-7.72197000	-4.66497700	0.78571000

Oleic Acid Allyl Radical optimized geometry [# opt freq scrf=(smd,solvent=water) def2tzvp mn15]

EE + Thermal Free Energy Correction: -855.044286 Ha

0 2

C	8.68124500	-1.61534600	0.12791700
O	8.35371800	-2.52686400	0.86149300
O	9.93383700	-1.49145000	-0.33016400
H	10.47753500	-2.22093300	0.01734500
C	7.77970300	-0.52842600	-0.36398400
H	8.22154600	0.42352800	-0.05452100
H	7.82608700	-0.53834100	-1.45700700
C	6.35048100	-0.65716400	0.12691700
H	5.94185100	-1.62238700	-0.18681100
H	6.33672600	-0.65164800	1.22086200
C	5.47347000	0.46664800	-0.40289400
H	5.88699400	1.43091500	-0.08544400
H	5.50219400	0.46373300	-1.49872100
C	4.03042700	0.35745500	0.06356600
H	3.61719200	-0.60645100	-0.25622500
H	4.00082700	0.35803700	1.15958500
C	3.15483400	1.48153900	-0.46535100
H	3.56182800	2.44565400	-0.14004400
H	3.18840300	1.48576900	-1.56077200
C	1.70713900	1.36857700	-0.00898100
H	1.29998000	0.39788100	-0.32449400
H	1.67147100	1.34724300	1.08929900
C	0.84674100	2.47367500	-0.52500300
H	1.32242300	3.23072100	-1.13978900
C	-0.51214300	2.58514000	-0.26374200
C	-1.25065900	1.70338400	0.49900100
H	-1.04030800	3.43490100	-0.69184900
H	-0.75630700	0.83984400	0.93736600
C	-2.71330000	1.83904500	0.74402700
H	-2.90889300	1.90922900	1.82195900
H	-3.07901500	2.76732500	0.29332800
C	-3.51456000	0.65530400	0.19838300
H	-3.12423300	-0.27366600	0.62899700
H	-3.36287100	0.58519900	-0.88434900
C	-5.00070200	0.77085400	0.49958300
H	-5.38372400	1.70896100	0.08052300
H	-5.14550300	0.83551900	1.58458000
C	-5.80995500	-0.39388900	-0.04959700
H	-5.42468800	-1.33199600	0.36756800
H	-5.66490100	-0.45653600	-1.13471700
C	-7.29625800	-0.28109400	0.25353100

H	-7.68031200	0.65884000	-0.16087500
H	-7.44137000	-0.22113400	1.33888700
C	-8.10758800	-1.44273100	-0.29890700
H	-7.72389800	-2.38347900	0.11440100
H	-7.96370400	-1.50274100	-1.38460000
C	-9.59429200	-1.33113600	0.00440800
H	-9.97403000	-0.39037100	-0.40781500
H	-9.73495800	-1.27189000	1.08877000
C	-10.38834400	-2.49996600	-0.55721700
H	-10.03514600	-3.44525200	-0.13746600
H	-11.45339500	-2.41286600	-0.33577700
H	-10.27566300	-2.55831800	-1.64275900

Oleic Acid Hydroperoxide optimized geometry [# opt freq scrf=(smd,solvent=water) def2tzvp mn15]

EE + Thermal Free Energy Correction: -1005.945797 Ha

0 1

C	8.74051300	-1.53771000	-0.27716700
O	9.10184200	-0.96196000	-1.28426600
O	9.55237400	-2.36476400	0.39425200
H	10.41566800	-2.40311000	-0.05506200
C	7.38903200	-1.42220200	0.35254500
H	6.97849500	-2.43344600	0.42807600
H	7.53985400	-1.08294900	1.38181400
C	6.44742900	-0.49987400	-0.39787500
H	6.89138000	0.49752500	-0.46881600
H	6.32190600	-0.86061900	-1.42310400
C	5.09082100	-0.40899000	0.28329800
H	4.65328000	-1.41122600	0.35835400
H	5.22339600	-0.05142500	1.31093800
C	4.12646000	0.50934700	-0.45004100
H	4.56188400	1.51290200	-0.52185700
H	3.99458300	0.15429100	-1.47851900
C	2.76706900	0.59797600	0.22790600
H	2.31510700	-0.39958400	0.27482900
H	2.91900400	0.92987700	1.26219900
C	1.83124100	1.55736000	-0.49076200
H	2.21277800	2.58128800	-0.40921100
H	1.81195500	1.31611300	-1.55971300
C	0.39173400	1.54066100	-0.01065900
H	-0.03758100	0.54109300	-0.13269400
C	-0.43806900	2.56795400	-0.73227900
C	-1.76286800	2.65871100	-0.62660600
H	0.10398200	3.27473700	-1.35375600
H	-2.27246600	3.44720500	-1.17683200
C	-2.64667400	1.76778700	0.18579600
H	-2.08936100	0.90546600	0.56356900
H	-2.99579200	2.32073300	1.06661400
C	-3.86527100	1.29608800	-0.60269600
H	-3.53508900	0.73731100	-1.48534300
H	-4.41355600	2.16917600	-0.97425600
C	-4.79521400	0.42827900	0.23033800
H	-5.11013100	0.98771400	1.11932600
H	-4.24504600	-0.44698900	0.59543200
C	-6.02449900	-0.03243200	-0.53750900
H	-5.70927800	-0.58604100	-1.43002700
H	-6.57630100	0.84424400	-0.89728200
C	-6.95251200	-0.90444900	0.29435100

H	-7.25998300	-0.35331500	1.19124200
H	-6.40263900	-1.78510100	0.64748600
C	-8.18945300	-1.35542700	-0.46685000
H	-7.88372100	-1.90423000	-1.36595000
H	-8.74163500	-0.47482600	-0.81708900
C	-9.11620500	-2.23048800	0.36393100
H	-9.41597600	-1.68161900	1.26285000
H	-8.56326700	-3.11021500	0.70955800
C	-10.34949700	-2.66619400	-0.41148900
H	-10.06675300	-3.23439400	-1.30127300
H	-11.00844700	-3.29394200	0.19075400
H	-10.92479200	-1.79812300	-0.74310400
O	0.29131000	1.84928200	1.39747200
O	0.32695000	0.64536200	2.14735100
H	1.26451800	0.54973400	2.38697800

Oleic Acid Hydroperoxyl Radical optimized geometry [# opt freq scrf=(smd,solvent=water) def2tzvp mn15]

EE + Thermal Free Energy Correction: -1005.318925 Ha

0 2

C	-8.31923100	-2.26518600	0.04374700
O	-7.85777900	-3.33796600	-0.29167500
O	-9.62380600	-2.10635600	0.30253400
H	-10.08332000	-2.95601100	0.17766500
C	-7.54153700	-0.99947800	0.21548300
H	-7.99641200	-0.24857400	-0.43742300
H	-7.71297100	-0.65021700	1.23804600
C	-6.06027600	-1.15365600	-0.07197500
H	-5.63648800	-1.91568000	0.58883900
H	-5.92177000	-1.51414200	-1.09561400
C	-5.31445200	0.15846800	0.11426500
H	-5.74825600	0.91972000	-0.54437900
H	-5.45964700	0.51728700	1.13970800
C	-3.82630900	0.03344900	-0.17107000
H	-3.39165700	-0.72631700	0.48870300
H	-3.68102500	-0.32630900	-1.19629800
C	-3.08241700	1.34700500	0.01275600
H	-3.51237200	2.10629100	-0.65009800
H	-3.22972600	1.70541700	1.03637800
C	-1.59560600	1.20988600	-0.28183400
H	-1.13841000	0.46949200	0.38240700
H	-1.45899800	0.84717200	-1.30403600
C	-0.84776600	2.51646500	-0.16036500
H	-1.35395100	3.30675800	-0.72172600
C	0.61359700	2.48988900	-0.48165600
C	1.27887100	1.44209200	-0.95772700
H	1.13692500	3.42708500	-0.30326700
H	0.75611500	0.50435200	-1.13478300
C	2.74122900	1.44269800	-1.26346100
H	2.89033000	1.22064500	-2.32634200
H	3.15824500	2.43720100	-1.07885400
C	3.49776300	0.39977900	-0.44341100
H	3.05226800	-0.58695200	-0.61263400
H	3.37493100	0.61878700	0.62293100
C	4.97832800	0.35473800	-0.78745800
H	5.41840900	1.34593500	-0.62627000
H	5.09445100	0.13515200	-1.85541200
C	5.74507400	-0.67597500	0.02659500
H	5.30382800	-1.66679800	-0.13477300
H	5.62684000	-0.45606800	1.09433300
C	7.22614400	-0.72241900	-0.31676800

H	7.66683200	0.26874000	-0.15530300
H	7.34388600	-0.94132500	-1.38489600
C	7.99489700	-1.75300000	0.49545700
H	7.55527600	-2.74482900	0.33405300
H	7.87752700	-1.53513700	1.56400700
C	9.47655300	-1.79944900	0.15293400
H	9.91258500	-0.80808800	0.31491300
H	9.59053600	-2.01582100	-0.91447700
C	10.22778100	-2.83557300	0.97401600
H	9.81815300	-3.83465600	0.80507900
H	11.28949600	-2.86254900	0.72305300
H	10.14160200	-2.61990400	2.04199300
O	-0.97274000	2.92687400	1.24791000
O	-0.77556200	4.19756500	1.40714500

Triplet Dioxygen optimized geometry [# opt freq scrf=(smd,solvent=water) def2tzvp mn15]

EE + Thermal Free Energy Correction: -150.251633 Ha

0 3

O	0.00000000	0.00000000	0.59692300
O	0.00000000	0.00000000	-0.59692300

Dioxygen Superoxide optimized geometry [# opt freq scrf=(smd,solvent=water) def2tzvp mn15]

EE + Thermal Free Energy Correction: -150.392704 Ha

-1 2

O	0.00000000	0.00000000	0.66048600
O	0.00000000	0.00000000	-0.66048600

12. ^{1}H-NMR spectra

14.1 Furfural-dimethyl-acetal

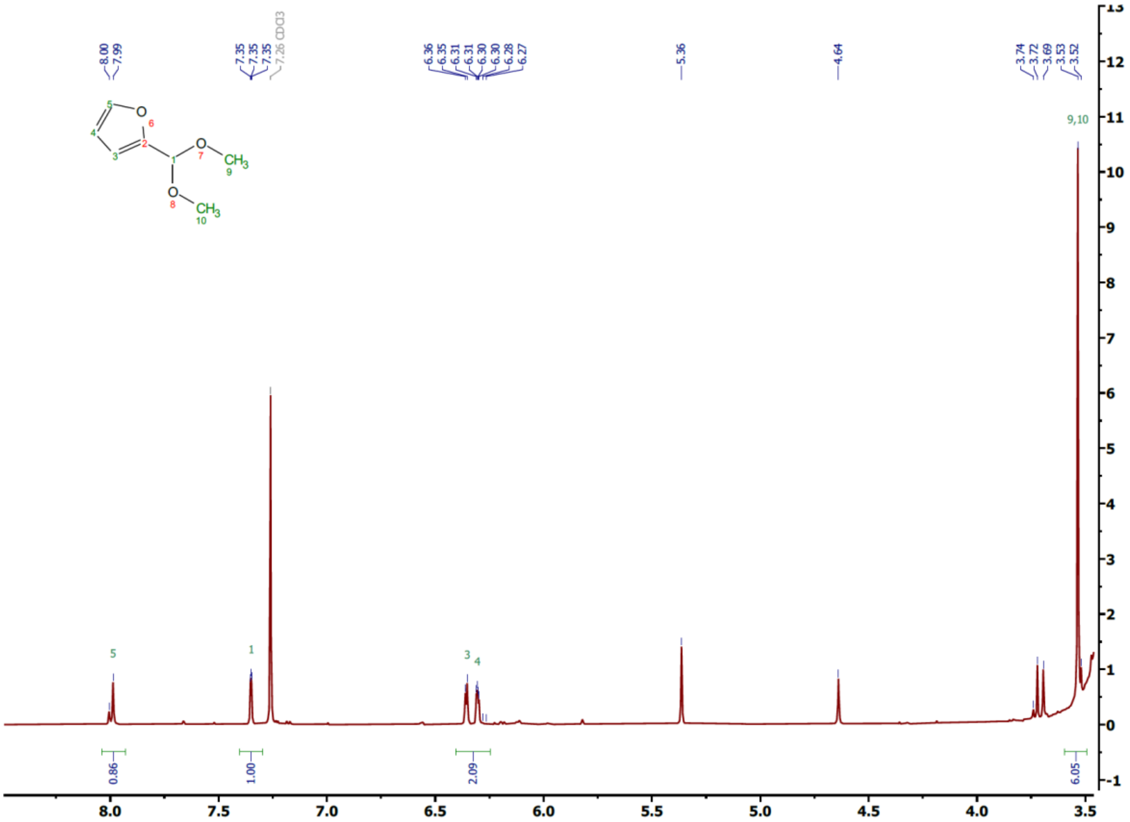

Figure S124: ^{1}H-NMR spectrum of quantitatively formed furfural-dimethyl-acetal measured in $CDCl_3$. ^{1}H-NMR (400 MHz, $CDCl_3$) δ 8.00 (d, J = 7.3 Hz, 1H), 7.40 – 7.30 (m, 1H), 6.31 (dd, J = 21.0, 3.0 Hz, 2H), 3.53 (s, 6H).

14.2 Surfactants and reactivity with oxygen

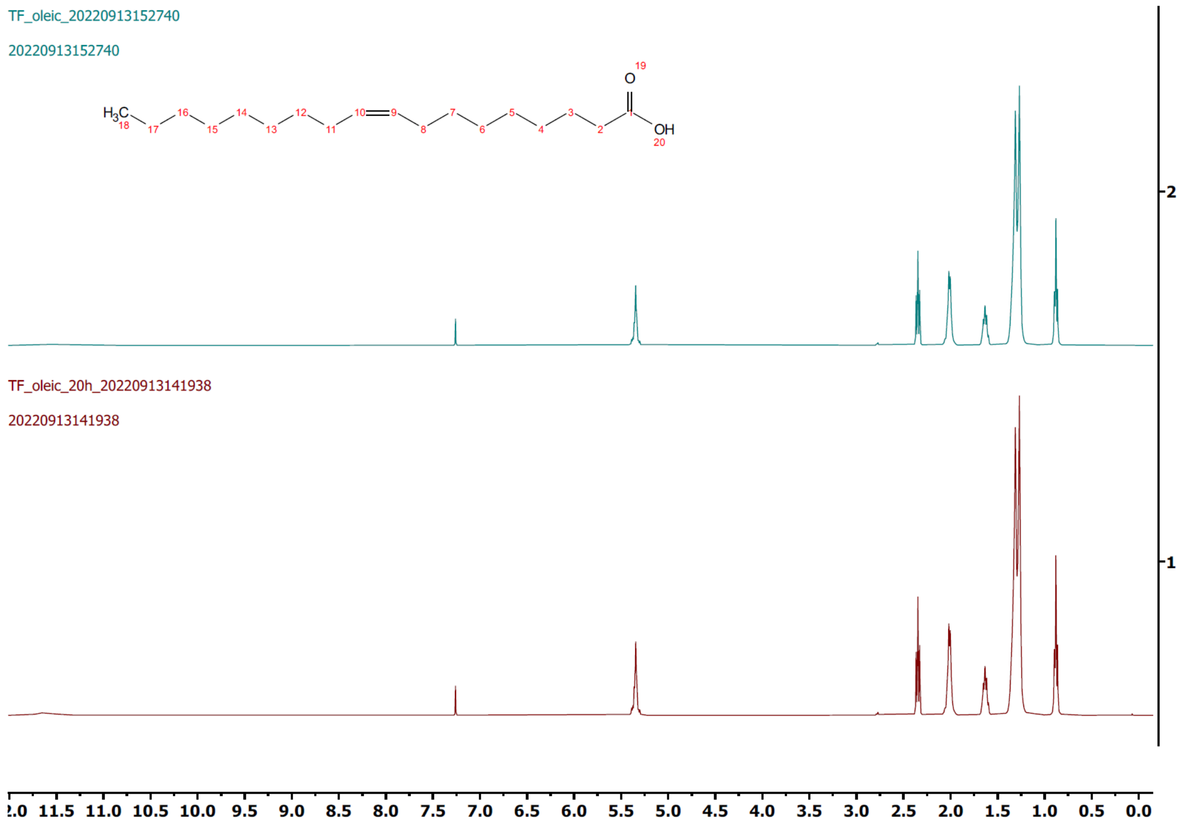

Figure S125: Stacked ^{1}H-NMR of oleic acid measured in $CDCl_3$. Reaction conditions: pure oleic acid was oxygenated in a closed vial *via* bubbling through the reactant for 30 minutes and stored in O_2 atmosphere over night. The reaction mixture was measured after 20 h. Peroxide teststrip before reaction: 0-0.5 mg L^{-1}; after reaction: 0-0.5 mg L^{-1} (no change observed, i.e. no significant autoxidation). Also, no changes in the NMR spectrum are obtained, confirming that the detection limit is not reached *via* blank autoxidation of the surfactant.

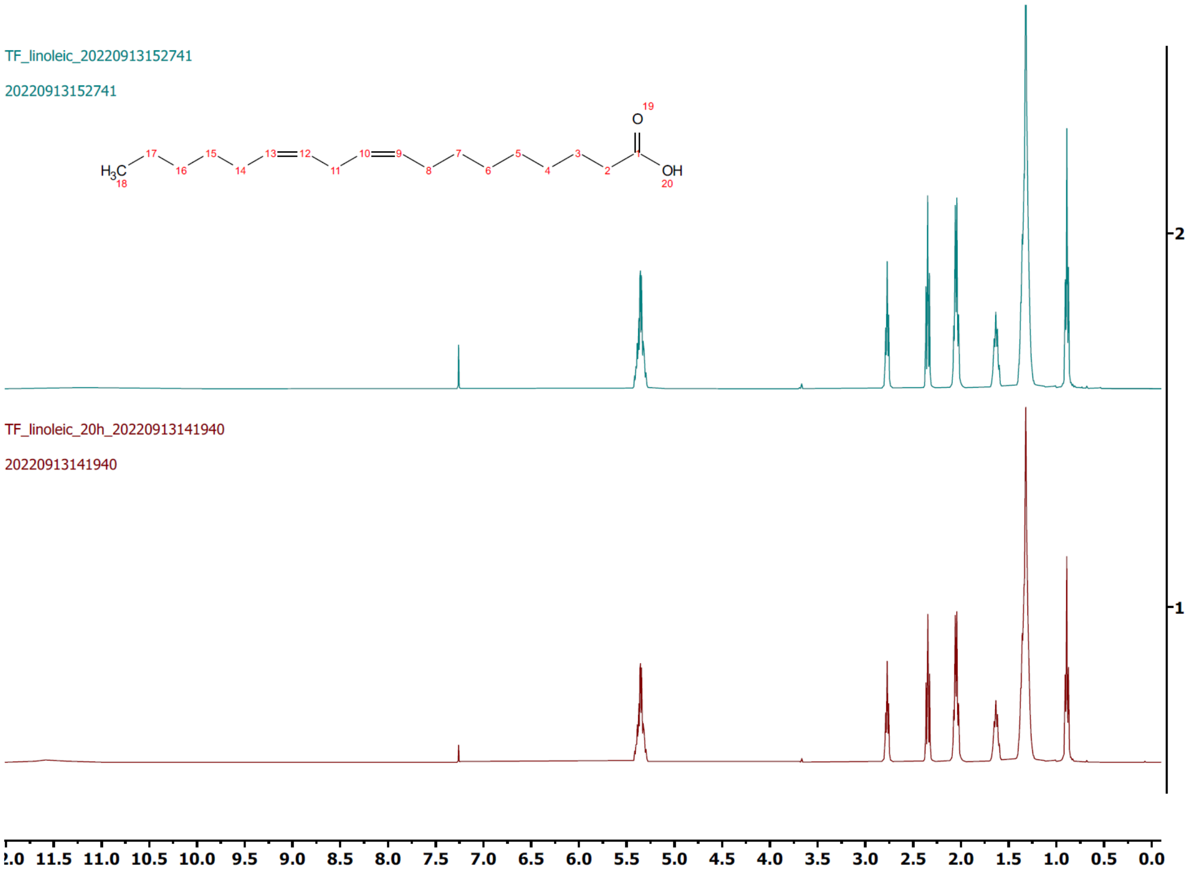

Figure S126: Stacked ^{1}H-NMR of linoleic acid measured in $CDCl_3$. Reaction conditions: pure linoleic acid was oxygenated in a closed vial *via* bubbling through the reactant for 30 minutes and stored in O_2 atmosphere over night. The reaction mixture was measured after 20 h. Peroxide teststrip before reaction: 0 mg L^{-1}; after reaction: 0.5 mg L^{-1}. No changes in the NMR spectrum are obtained while peroxide teststrips turned blue (autoxidation), confirming that the detection limit is not reached *via* blank autoxidation of the surfactant.

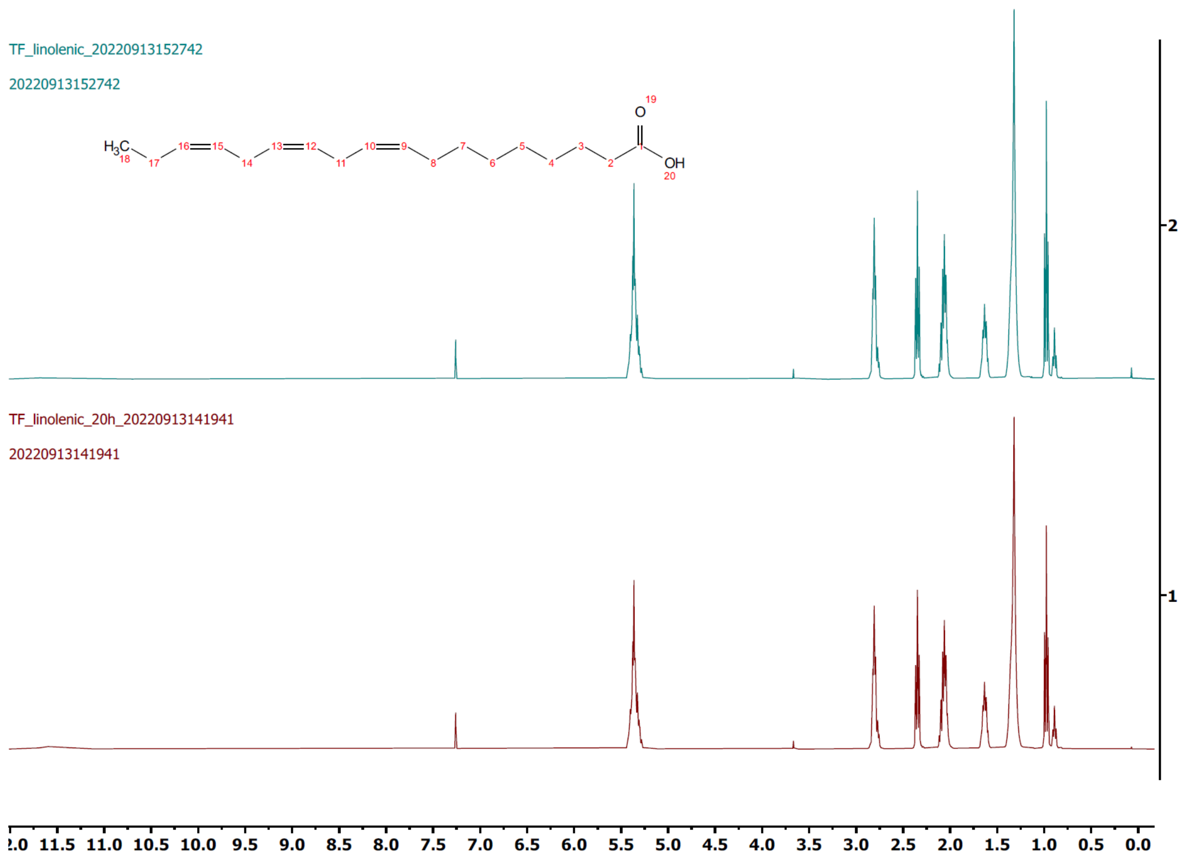

Figure S127: Stacked ^{1}H-NMR of linolenic acid measured in $CDCl_3$. Reaction conditions: pure linolenic acid was oxygenated in a closed vial *via* bubbling through the reactant for 30 minutes and stored in O_2 atmosphere over night. The reaction mixture was measured after 20 h. Peroxide teststrip before reaction: 0 mg L^{-1}; after reaction: 0.5-2 mg L^{-1}. No changes in the NMR spectrum are obtained while peroxide teststrips turned blue (autoxidation), confirming that the detection limit is not reached *via* blank autoxidation of the surfactant.

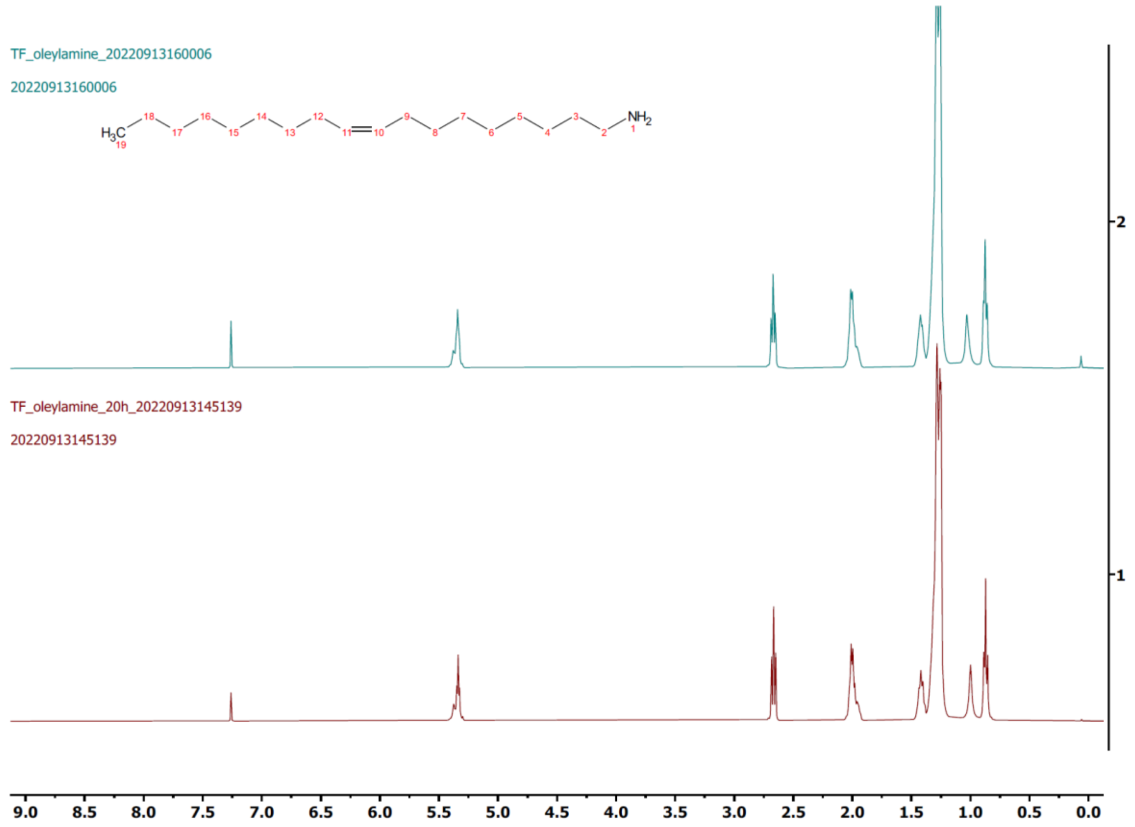

Figure S128: Stacked ^{1}H-NMR of oleylamine measured in $CDCl_3$. Reaction conditions: pure oleylamine was oxygenated in a closed vial *via* bubbling through the reactant for 30 minutes and stored in O_2 atmosphere over night. The reaction mixture was measured after 20 h. Peroxide teststrip before reaction: 0 mg L^{-1}; after reaction: 0 mg L^{-1} (no change observed, i.e. no significant autoxidation). Also, no changes in the NMR spectrum are obtained, confirming that the detection limit is not reached *via* blank autoxidation of the surfactant.

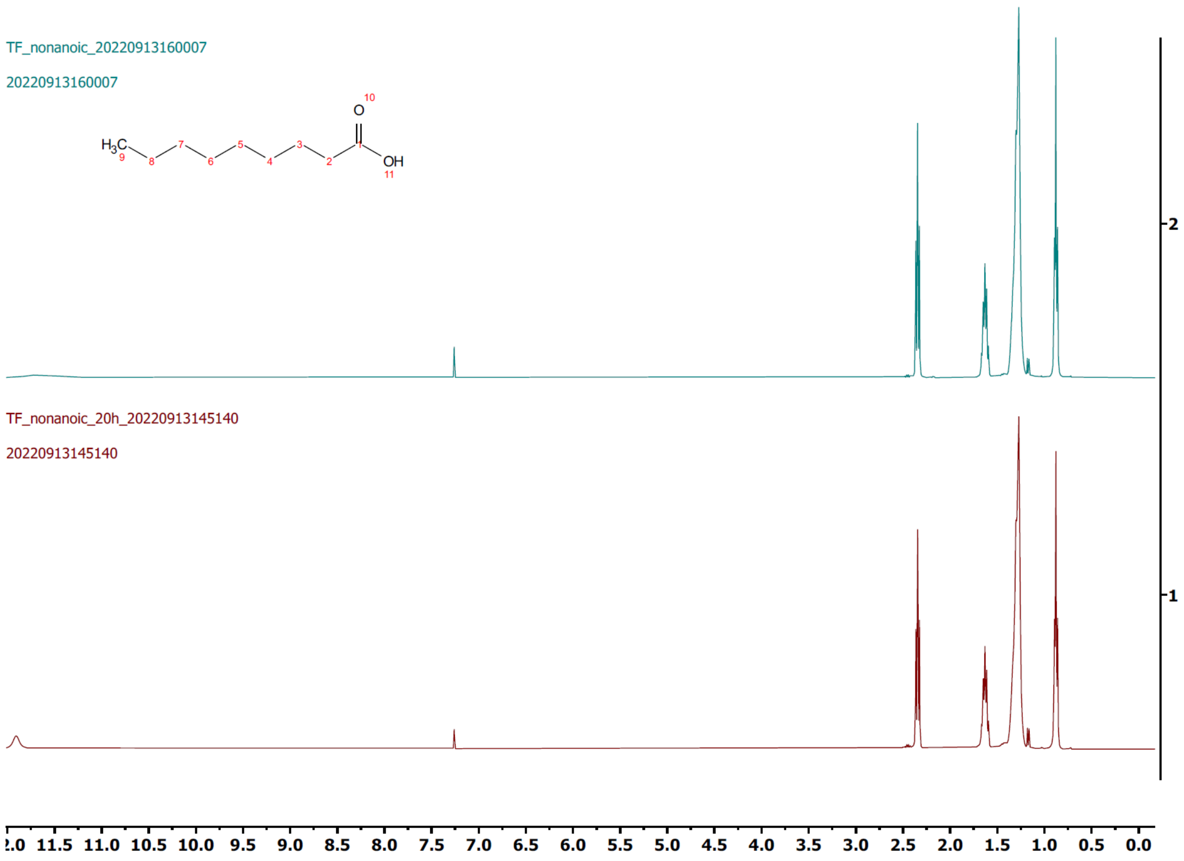

Figure S129: Stacked ^{1}H-NMR of nonanoic acid measured in $CDCl_3$. Reaction conditions: pure nonanoic acid was oxygenated in a closed vial *via* bubbling through the reactant for 30 minutes and stored in O_2 atmosphere over night. The reaction mixture was measured after 20 h. Peroxide teststrip before reaction: 0 mg L^{-1}; after reaction: 0 mg L^{-1} (no change observed, i.e. no significant autoxidation). Also, no changes in the NMR spectrum are obtained, confirming that the detection limit is not reached *via* blank autoxidation of the surfactant.

13. GC-MS

15.1 Styrene oxidation in ethyl acetate

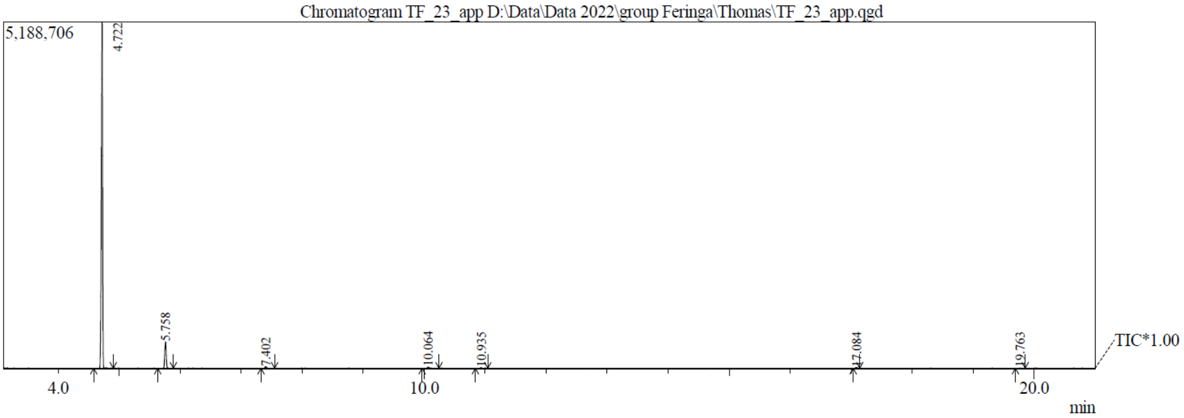

Peak Report TIC

Peak#	R.Time	Area	Area%	Height	Height%	Name	Base m/z	Base Int.
1	4.722	7810653	92.18	5173342	91.82	Styrene	104.10	1572564
2	5.758	564038	6.66	393401	6.98	Benzaldehyde	77.10	78025
3	7.402	33967	0.40	23634	0.42	Benzene, (epoxyethyl)-, (R)-	91.10	3207
4	10.064	31620	0.37	17437	0.31	Benzeneacetic acid, .alpha.-oxo-, methyl ester	105.10	5904
5	10.935	10665	0.13	8324	0.15		107.05	1587
6	17.084	16645	0.20	11006	0.20		104.10	2418
7	19.763	5768	0.07	6771	0.12		105.10	2668
		8473356	100.00	5633915	100.00			

Figure S130: GC-MS of styrene oxidation in ethyl acetate.

<< Target >>
Line#:1 R.Time:4.720(Scan#:325) MassPeaks:269
RawMode:Averaged 4.715-4.725(324-326) BasePeak:104.10(1572564)
BG Mode:Calc. from Peak Group 1 - Event 1 Scan

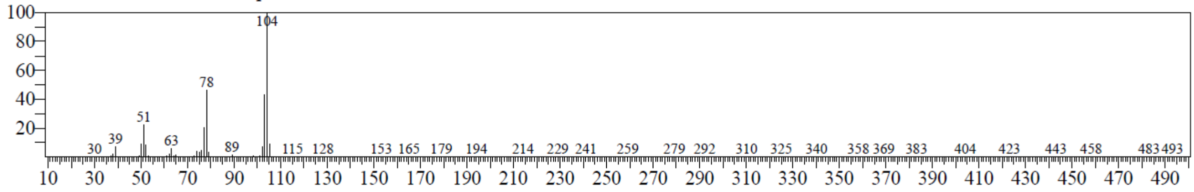

Hit#:1 Entry:2558 Library:NIST11.lib
SI:98 Formula:C8H8 CAS:100-42-5 MolWeight:104 RetIndex:883
CompName:Styrene $$ Benzene, ethenyl- $$ Bulstren K-525-19 $$ Cinnamene $$ Phenethylene $$ Phenylethene $$ Phenylethylene $$ Styrol (German) $$

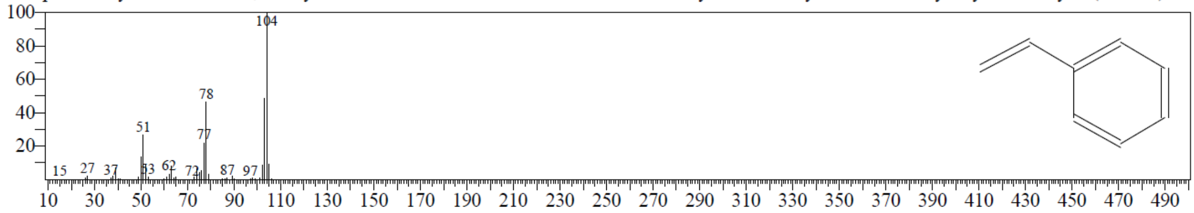

Figure S131: Mass spectrum of unreacted styrene (retention time 4.720).

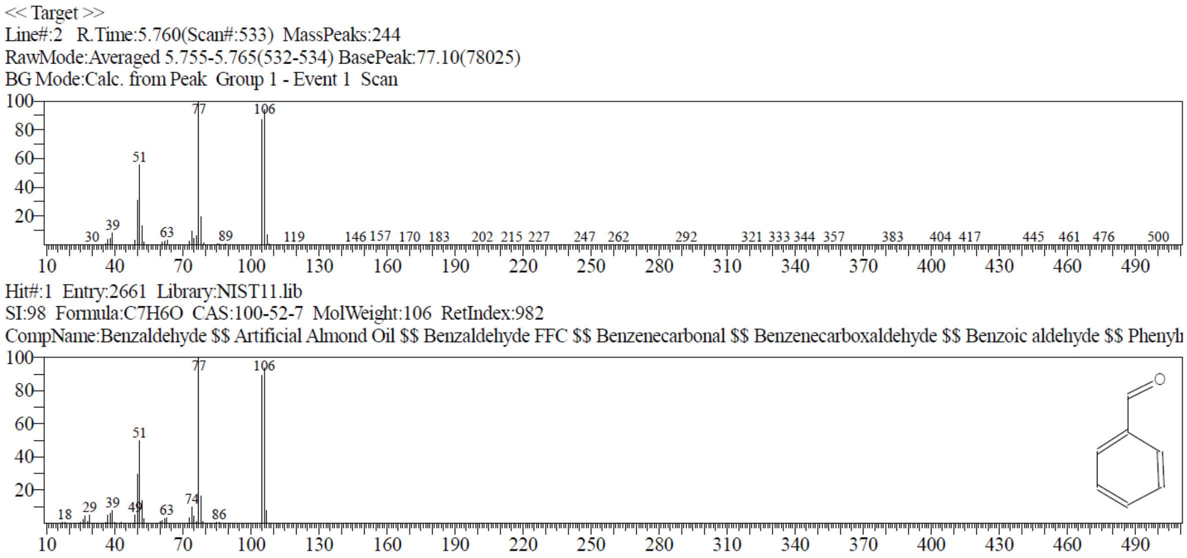

Figure S132: Mass spectrum of formed benzaldehyde (retention time 5.760).

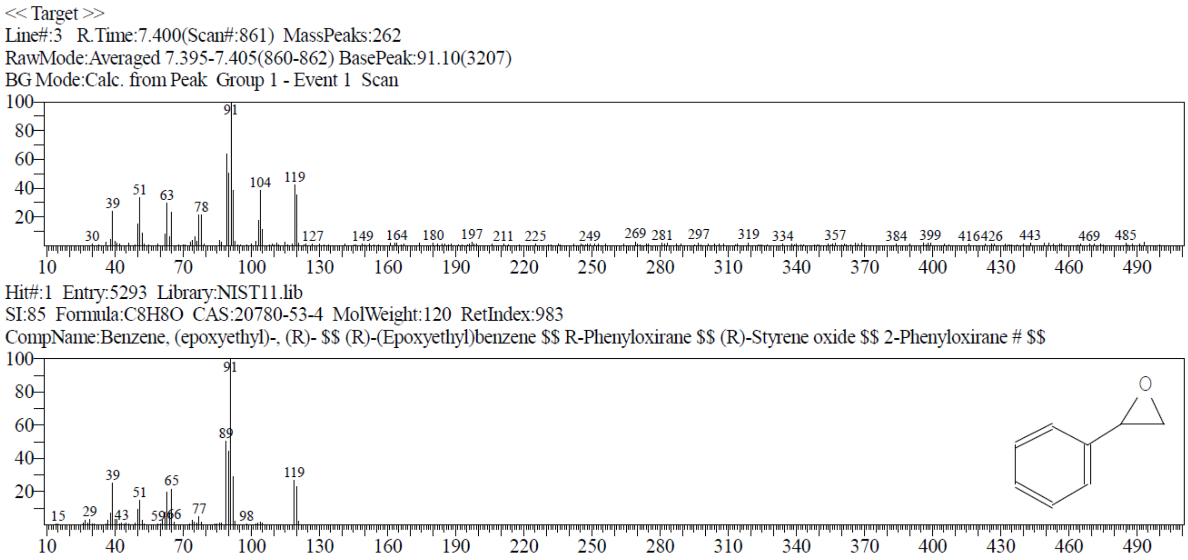

Figure S133: Mass spectrum of formed benzepoxy (retention time 7.400).

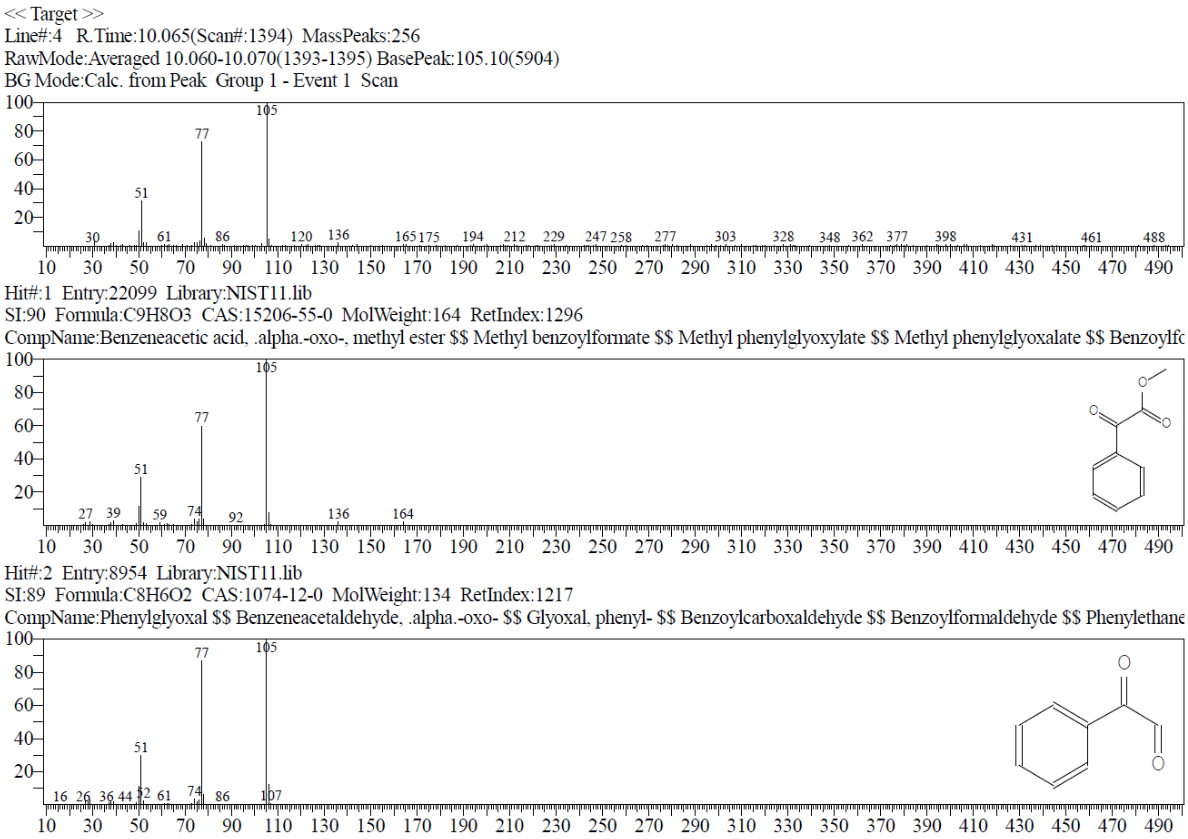

Figure S134: Mass spectrum of formed benzeneacetaldehyde (retention time 10.065).

15.2 Styrene oxidation in methanol

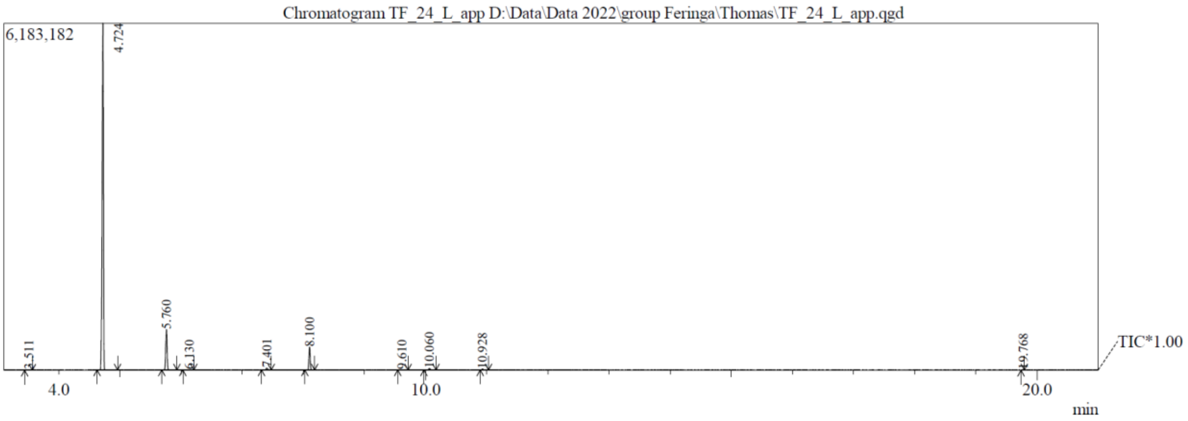

Peak Report TIC

Peak#	R.Time	Area	Area%	Height	Height%	Name	Base m/z	Base Int.
1	3.511	22963	0.20	7906	0.11		43.00	1771
2	4.724	9526300	84.00	6171694	83.35	Styrene	104.10	1824334
3	5.760	1005317	8.86	719225	9.71	Benzaldehyde	77.05	138709
4	6.130	21848	0.19	8569	0.12		43.00	3536
5	7.401	56596	0.50	32482	0.44	Benzene, (epoxyethyl)-, (R)-	91.05	4416
6	8.100	595708	5.25	406744	5.49	Benzaldehyde dimethyl acetal	121.10	150915
7	9.610	8307	0.07	6002	0.08		121.10	1932
8	10.060	61801	0.54	27293	0.37	Phenylglyoxal	105.10	8637
9	10.928	27454	0.24	17539	0.24	Propionic acid, 2,3-dihydroxy-3-phenyl-	79.05	2935
10	19.768	14957	0.13	7150	0.10		105.05	2110
		11341251	100.00	7404604	100.00			

Figure S135: GC-MS of styrene oxidation in methanol, liquid fraction.

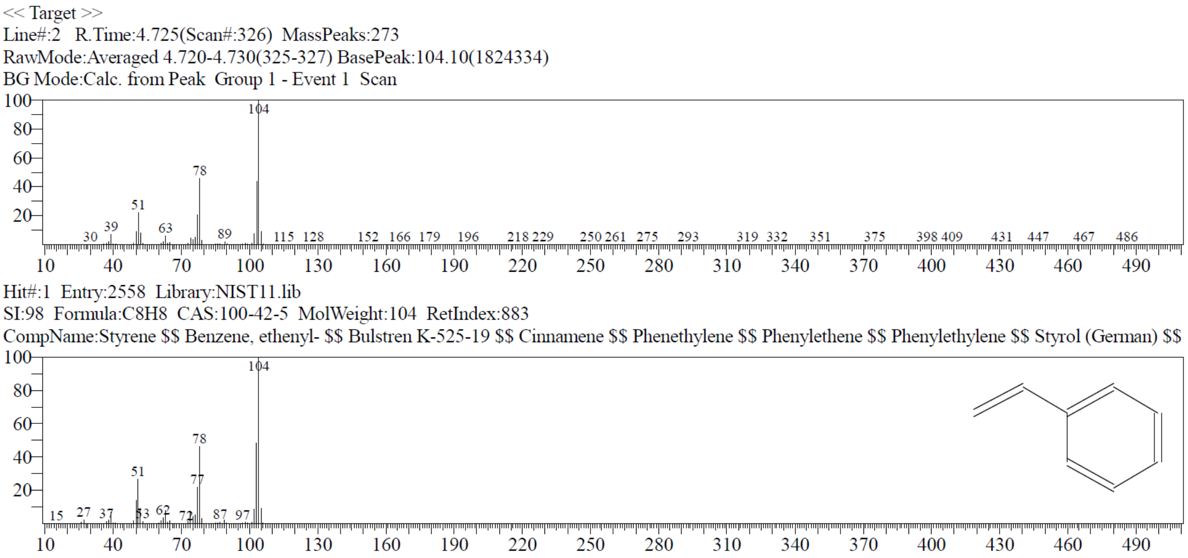

Figure S136: Mass spectrum of unreacted styrene from liquid fraction (retention time 4.725).

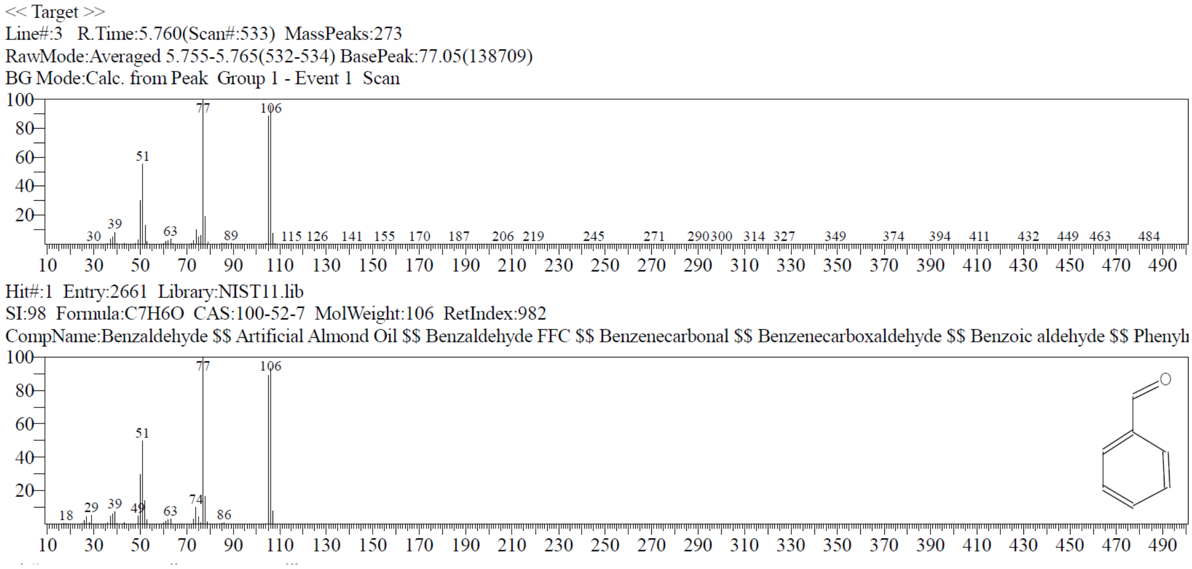

Figure S137: Mass spectrum of formed benzaldehyde from liquid fraction (retention time 5.760).

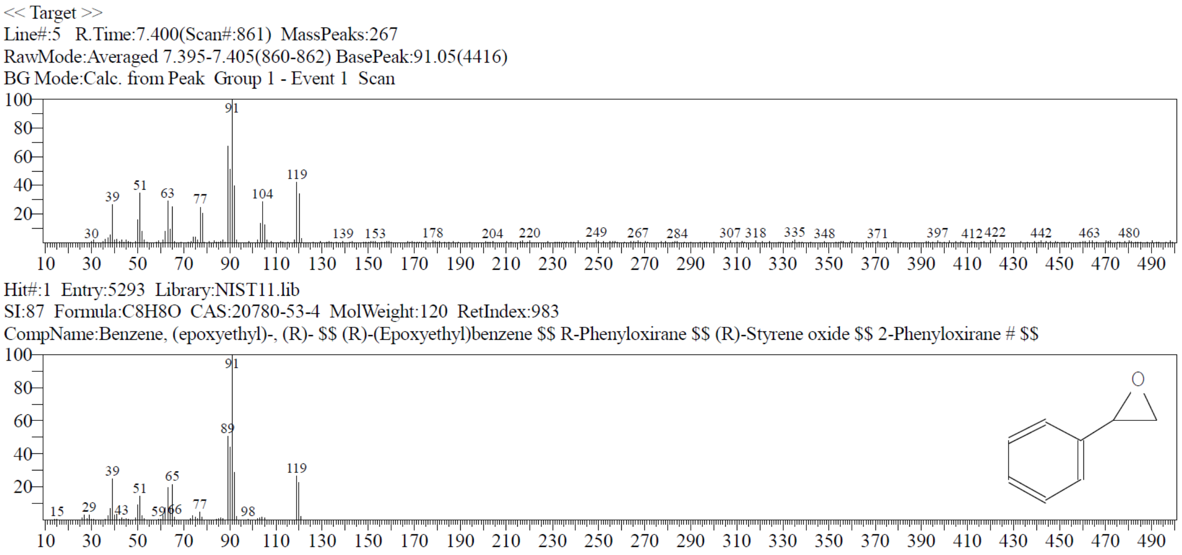

Figure S138: Mass spectrum of formed benzepoxy from liquid fraction (retention time 7.400).

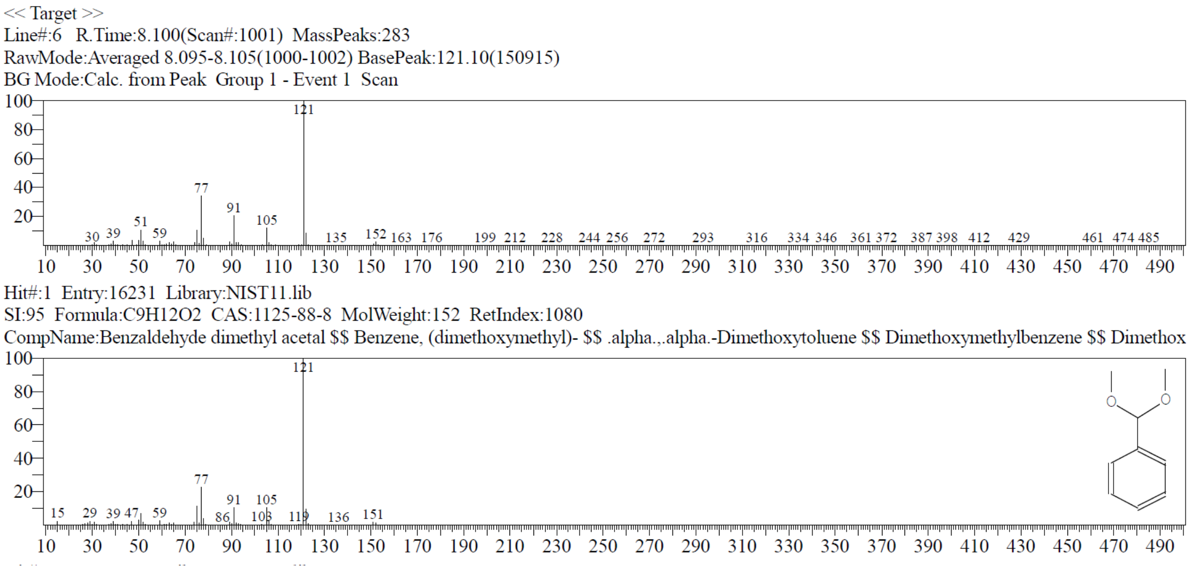

Figure S139: Mass spectrum of formed dimethyl acetal from liquid fraction (retention time 8.100).

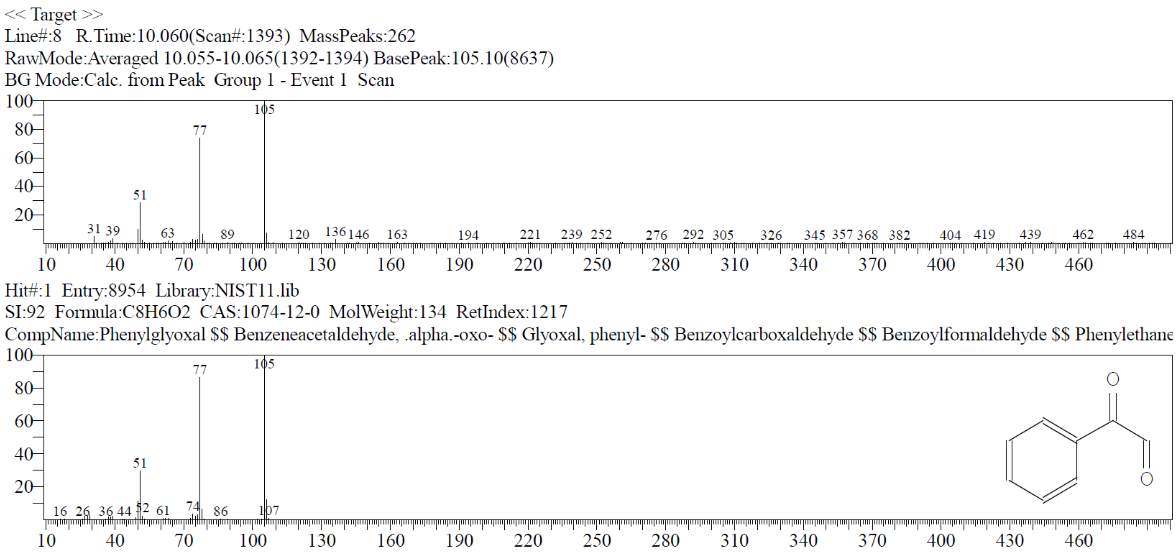

Figure S140: Mass spectrum of formed benzeneacetaldehyde (retention time 10.060).

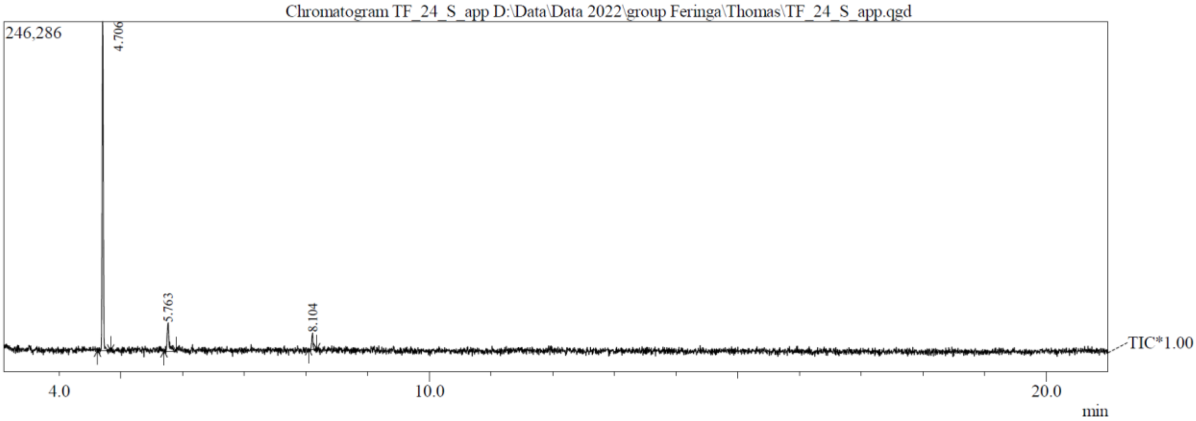

Peak Report TIC

Peak#	R.Time	Area	Area%	Height	Height%	Name	Base m/z	Base Int.
1	4.706	316620	84.62	231150	88.06	Styrene	104.10	60979
2	5.763	45309	12.11	19985	7.61	Benzaldehyde	77.05	3498
3	8.104	12238	3.27	11349	4.32		121.10	3541
		374167	100.00	262484	100.00			

Figure S141: GC-MS of styrene oxidation in methanol, solid fraction. Peaks from left to right: styrene, benzaldehyde and the dimethyl acetal.

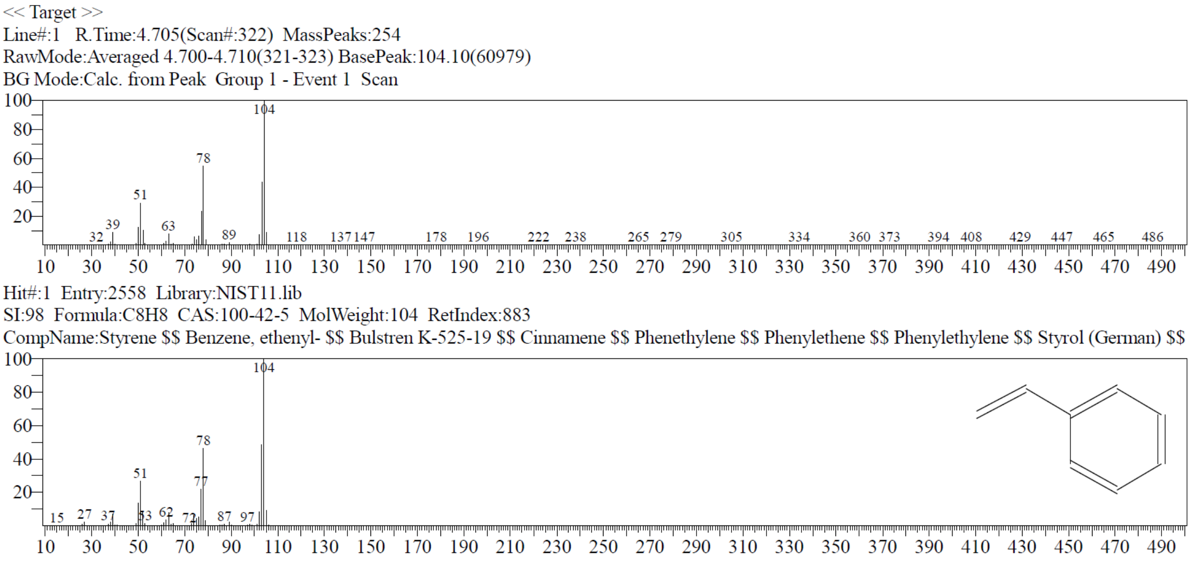

Figure S142: Mass spectrum of unreacted styrene from solid fraction (retention time 4.705).

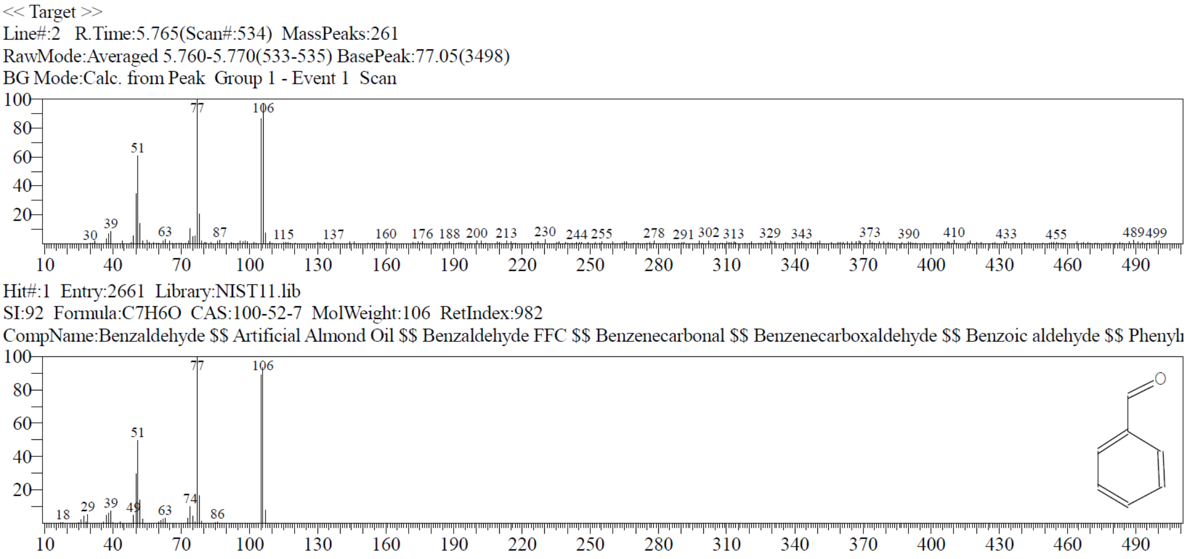

Figure S143: Mass spectrum of formed benzaldehyde from solid fraction (retention time 5.765)

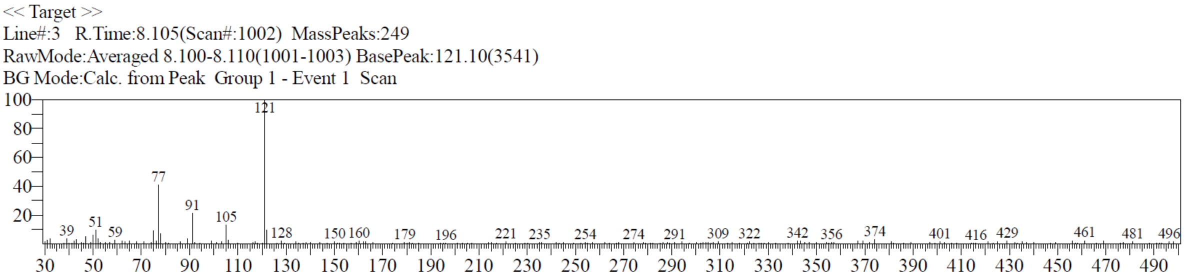

Figure S144: Mass spectrum of formed dimethyl acetal from solid fraction (retention time 8.105)

15.3 Furfural nucleophilic addition

Chromatogram TF_app1 D:\Data\Data 2022\group Feringa\Thomas\TF_app2.qgd

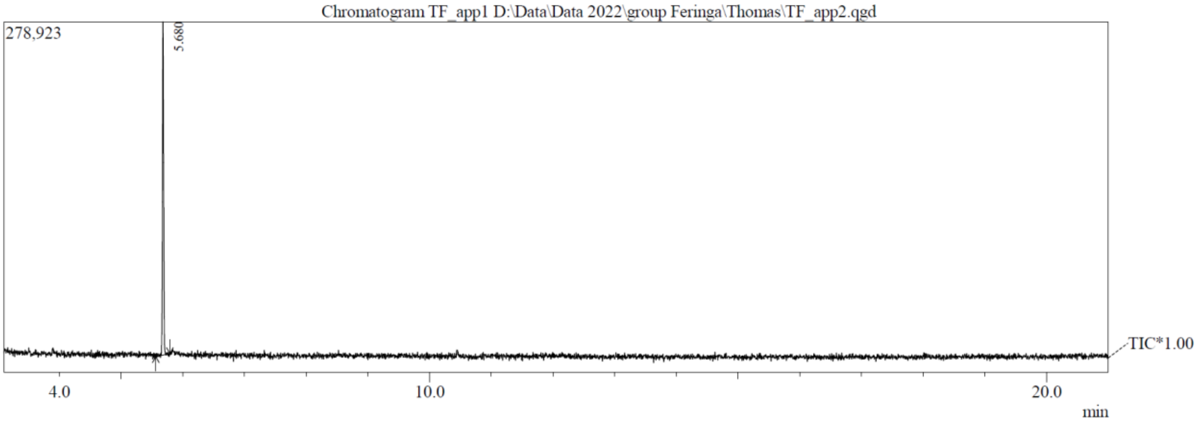

Peak Report TIC

Peak#	R.Time	Area	Area%	Height	Height%	Name	Base m/z	Base Int.
1	5.680	359302	100.00	264914	100.00	2(1H)-Pyridinone, 3-hydroxy-	111.10	103503
		359302	100.00	264914	100.00			

Figure S145: GC-MS of quantitatively formed furfural-dimethyl-acetal.

<< Target >>
Line#:1 R.Time:5.680(Scan#:517) MassPeaks:277
RawMode:Averaged 5.675-5.685(516-518) BasePeak:111.10(103503)
BG Mode:Calc. from Peak Group 1 - Event 1 Scan

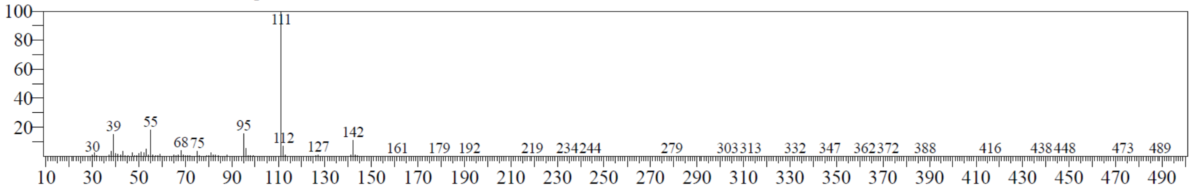

Hit#:4 Entry:25715 Library:NIST11.lib
SI:81 Formula:C9H14O3 CAS:72403-07-7 MolWeight:170 RetIndex:1128
CompName:Furan, 2-(2-ethoxy-1-methoxyethyl)- $$ 2-(2-Ethoxy-1-methoxyethyl)furan # $$

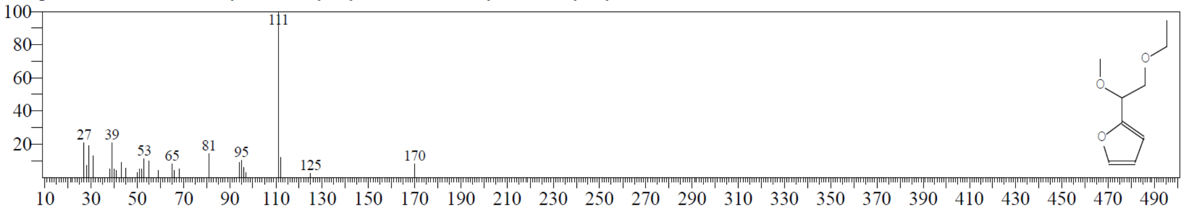

Figure S146: Mass spectrum of quantitatively formed furfural-dimethyl-acetal and its similarity to a model library compound (retention time 5.680)

15.4 α-terpinene oxidation

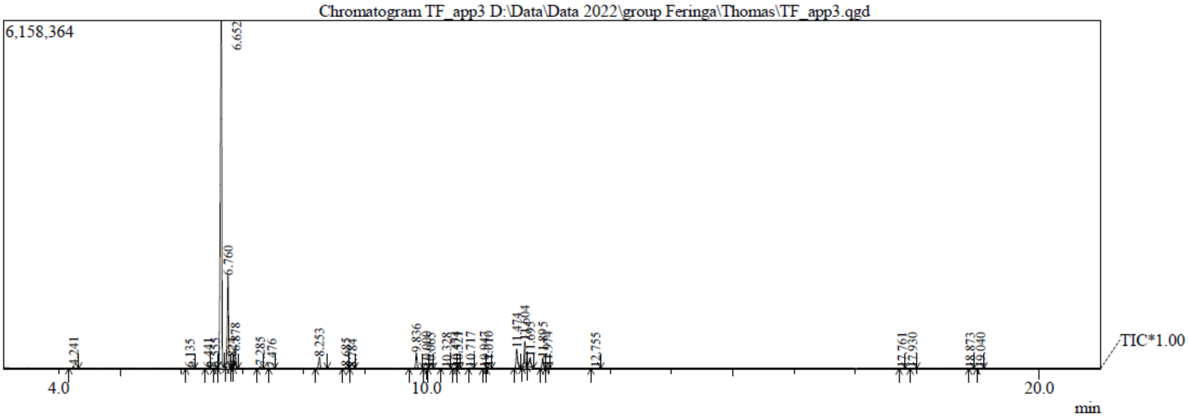

Peak Report TIC

Peak#	R.Time	Area	Area%	Height	Height%	Name	Base m/z	Base Int.
1	4.241	50405	0.30	39390	0.39	Boric acid (H3BO3), tris(1-methylethyl) ester	87.10	5180
2	6.135	16500	0.10	9170	0.09		43.00	3533
3	6.441	16874	0.10	13539	0.14	Bicyclo[3.1.0]hex-2-ene, 2-methyl-5-(1-methy	93.10	3335
4	6.555	14078	0.08	11597	0.12	1,4-Cyclohex-2-enedione	54.00	3128
5	6.652	10859740	65.04	6130121	61.33	(+)-4-Carene	121.15	1021139
6	6.760	2201310	13.18	1622544	16.23	Benzene, 1-methyl-3-(1-methylethyl)-	119.15	534756
7	6.824	27556	0.17	17647	0.18	D-Limonene	68.10	2614
8	6.878	413953	2.48	285477	2.86	Eucalyptol	43.05	33485
9	7.285	41579	0.25	32412	0.32	.gamma.-Terpinene	93.10	6337
10	7.476	6340	0.04	6300	0.06	n-Caproic acid vinyl ester	43.00	2304
11	8.253	334388	2.00	197043	1.97	Cyclohexanone, 2-ethyl-	55.05	26975
12	8.685	34578	0.21	23258	0.23		109.10	2722
13	8.784	8816	0.05	9569	0.10		109.10	867
14	9.836	464026	2.78	273689	2.74	(1R,2R,3R,5S)-(-)-Isopinocampheol	43.05	48862
15	10.000	21674	0.13	14812	0.15		137.20	1885
16	10.065	8924	0.05	8840	0.09		81.10	907
17	10.328	11176	0.07	8577	0.09		43.05	764
18	10.474	19759	0.12	14513	0.15	1,4-dihydroxy-p-menth-2-ene	109.10	2768
19	10.521	32328	0.19	23590	0.24		81.10	4264
20	10.717	28868	0.17	13751	0.14	1,4-dihydroxy-p-menth-2-ene	109.15	1996
21	10.947	26521	0.16	14711	0.15		95.10	2266
22	11.010	39548	0.24	24385	0.24		43.05	3754
23	11.474	626610	3.75	335740	3.36	1,4-dihydroxy-p-menth-2-ene	109.10	62196
24	11.604	653410	3.91	445271	4.45		112.10	67394
25	11.695	345868	2.07	165445	1.66	5-Isopropyl-6-methyl-hepta-3,5-dien-2-ol	43.05	28880
26	11.895	287227	1.72	182768	1.83	1,4-dihydroxy-p-menth-2-ene	109.10	37187
27	11.974	22825	0.14	18344	0.18		109.10	2597
28	12.755	14274	0.09	8898	0.09		43.05	1046
29	17.761	15812	0.09	12632	0.13		43.05	1128
30	17.930	16645	0.10	12831	0.13		43.05	982
31	18.873	10777	0.06	6945	0.07		43.05	598
32	19.040	25516	0.15	11253	0.11		136.15	1147

Figure S147: GC-MS of α-terpinene oxidation by iron oxide nanoparticles in isopropanol.

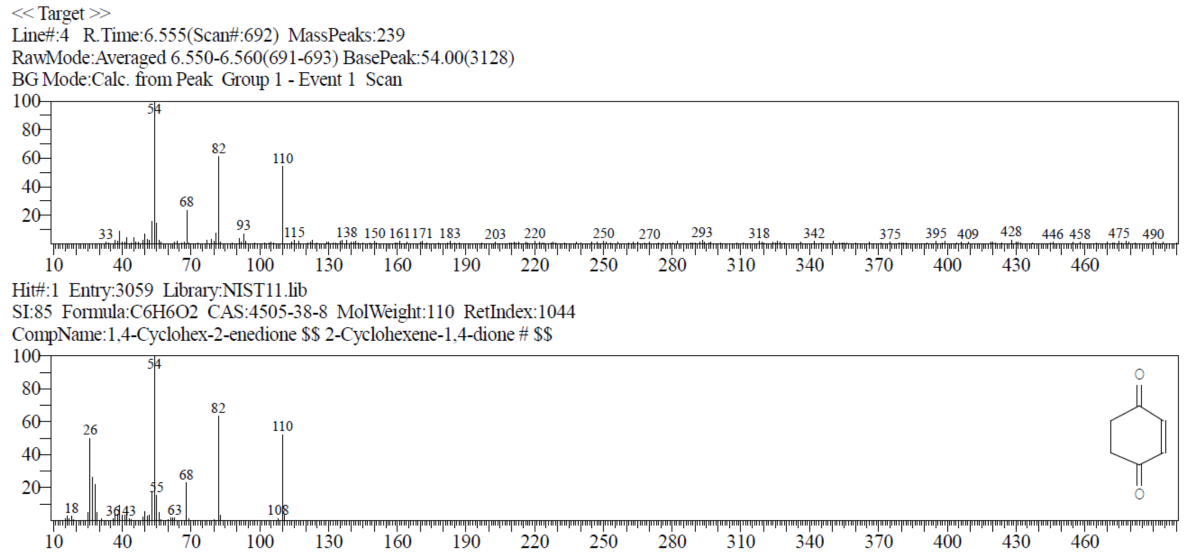

Figure S148: Mass spectrum of 1,4-Cyclohex-2-enedione (retention time 6.555).

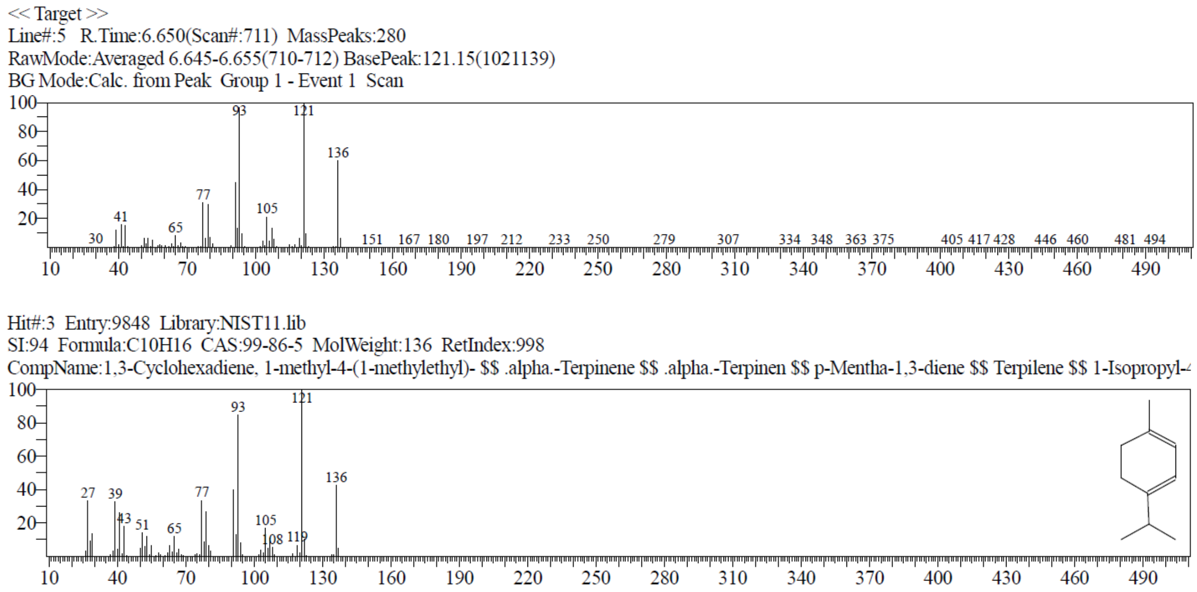

Figure S149: Mass spectrum of unreacted α-terpinene (retention time 6.650).

<< Target >>
Line#:26 R.Time:11.895(Scan#:1760) MassPeaks:293
RawMode:Averaged 11.890-11.900(1759-1761) BasePeak:109.10(37187)
BG Mode:Calc. from Peak Group 1 - Event 1 Scan

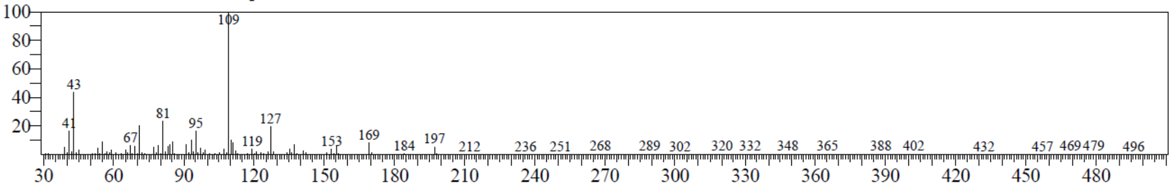

Hit#:1 Entry:25960 Library:NIST11.lib
SI:81 Formula:C10H18O2 CAS:0-00-0 MolWeight:170 RetIndex:1259
CompName:1,4-dihydroxy-p-menth-2-ene

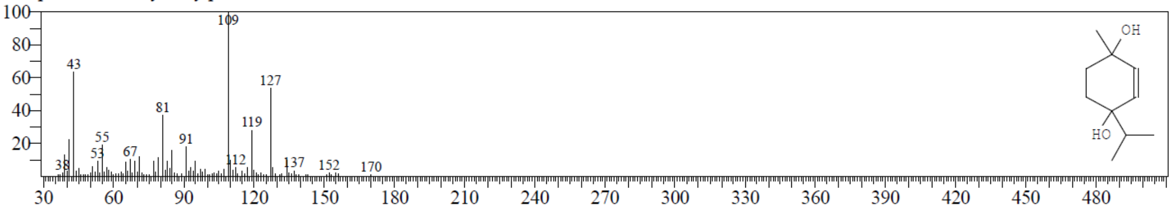

<< Target >>
Line#:8 R.Time:6.880(Scan#:757) MassPeaks:282
RawMode:Averaged 6.875-6.885(756-758) BasePeak:43.05(33485)
BG Mode:Calc. from Peak Group 1 - Event 1 Scan

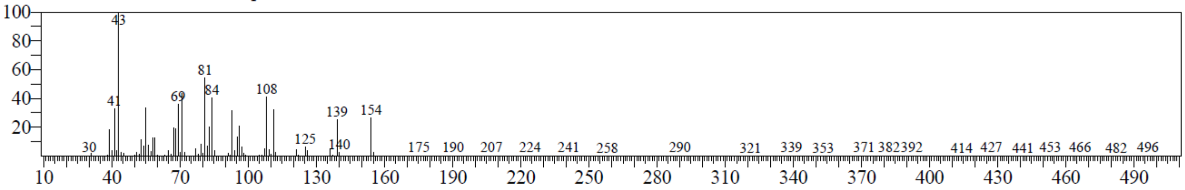

Hit#:1 Entry:17478 Library:NIST11.lib
SI:95 Formula:C10H18O CAS:470-82-6 MolWeight:154 RetIndex:1059
CompName:Eucalyptol $$ Cineole $$ 2-Oxabicyclo[2.2.2]octane, 1,3,3-trimethyl- $$ p-Menthane, 1,8-epoxy- $$ p-Cineole $$ Cajeputol $$ Cucalyptol $$ I

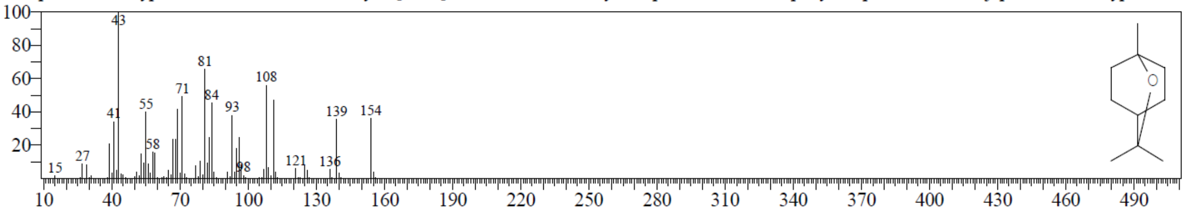

Figure S150: Mass spectrum of eucalyptol (retention time 6.880).

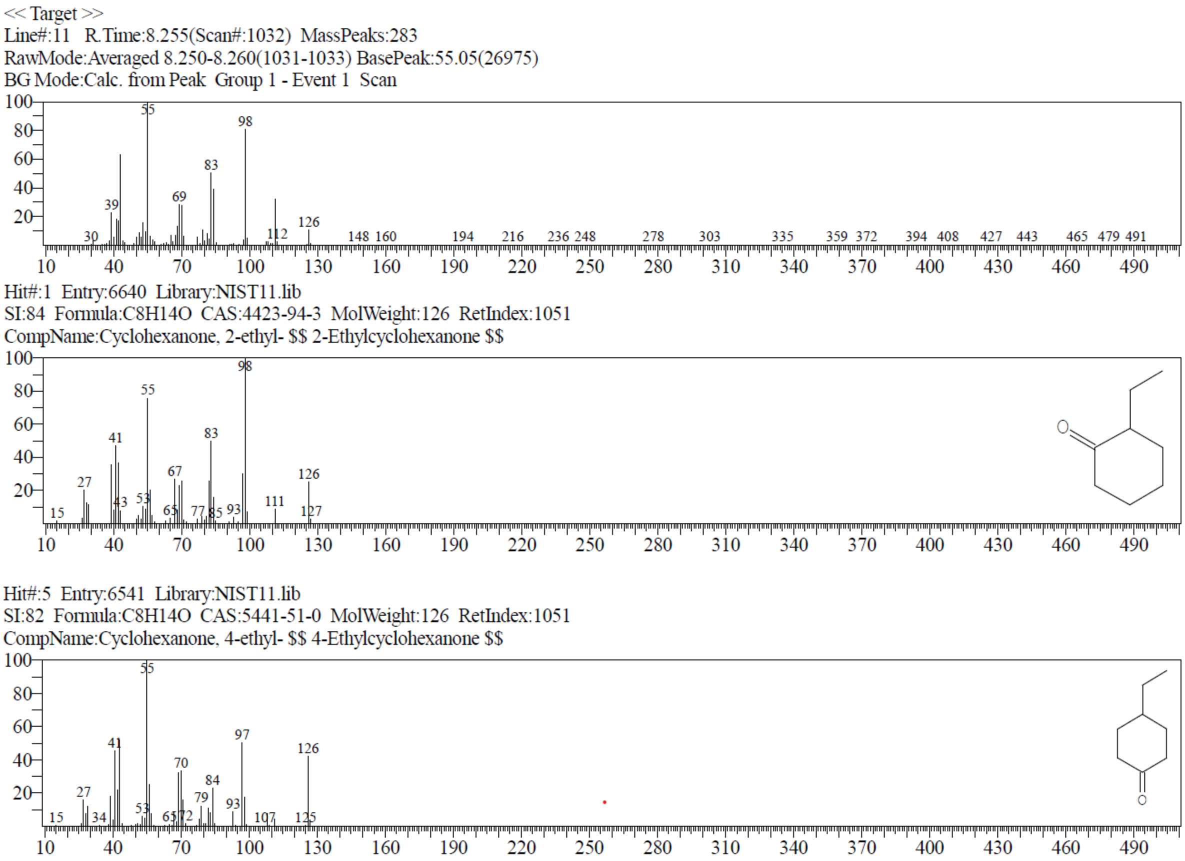

Figure S151: Mass spectrum of cyclohexanone (retention time 8.255).

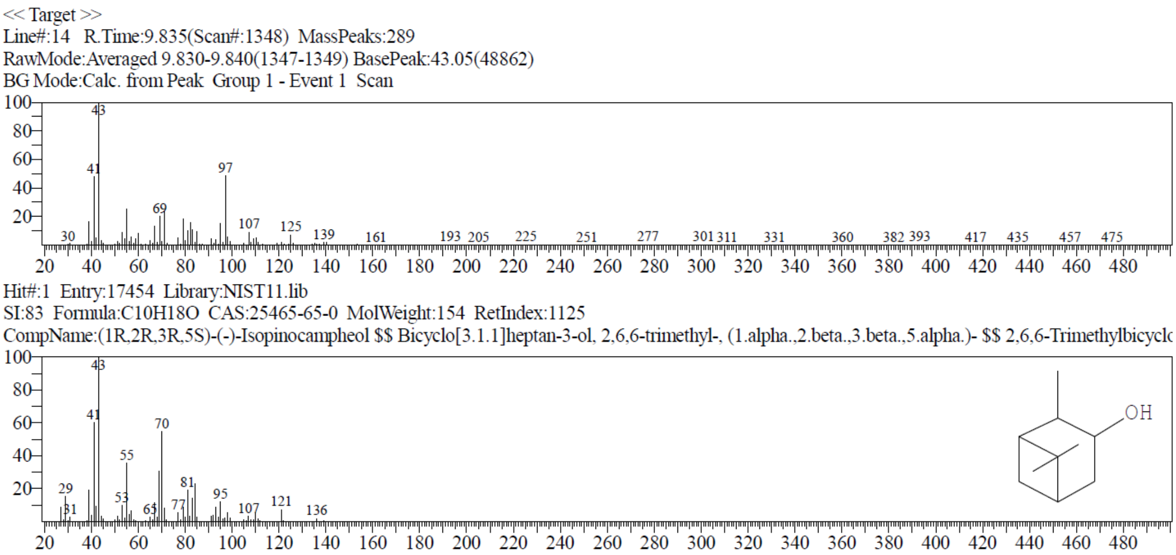

Figure S152: Mass spectrum of (+)-Isopinocampheol (retention time 9.835).

<< Target >>
Line#:18 R.Time:10.475(Scan#:1476) MassPeaks:267
RawMode:Averaged 10.470-10.480(1475-1477) BasePeak:109.10(2768)
BG Mode:Calc. from Peak Group 1 - Event 1 Scan

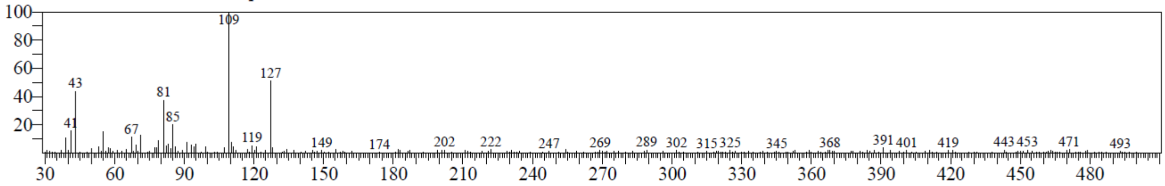

Hit#:1 Entry:25960 Library:NIST11.lib
SI:84 Formula:C10H18O2 CAS:0-00-0 MolWeight:170 RetIndex:1259
CompName:1,4-dihydroxy-p-menth-2-ene

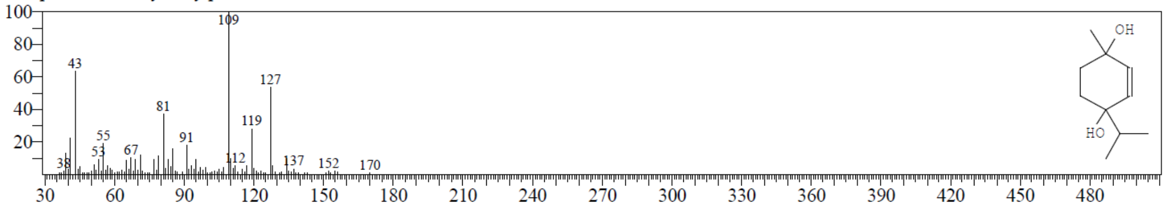

Figure S153: Mass spectrum of 1,4-dihydroxy-p-menth-2-ene (retention time 10.475).

<< Target >>
Line#:20 R.Time:10.715(Scan#:1524) MassPeaks:272
RawMode:Averaged 10.710-10.720(1523-1525) BasePeak:109.15(1996)
BG Mode:Calc. from Peak Group 1 - Event 1 Scan

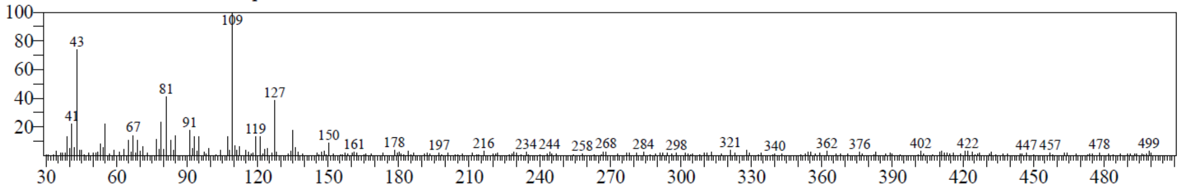

Hit#:1 Entry:25960 Library:NIST11.lib
SI:82 Formula:C10H18O2 CAS:0-00-0 MolWeight:170 RetIndex:1259
CompName:1,4-dihydroxy-p-menth-2-ene

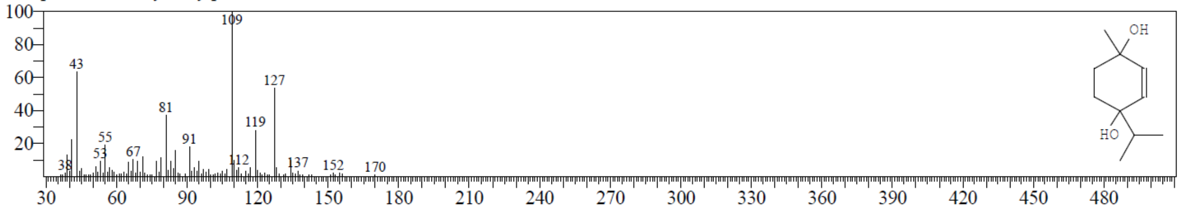

Figure S154: Mass spectrum of 1,4-dihydroxy-p-menth-2-ene (retention time 10.715).

<< Target >>
Line#:23 R.Time:11.475(Scan#:1676) MassPeaks:301
RawMode:Averaged 11.470-11.480(1675-1677) BasePeak:109.10(62196)
BG Mode:Calc. from Peak Group 1 - Event 1 Scan

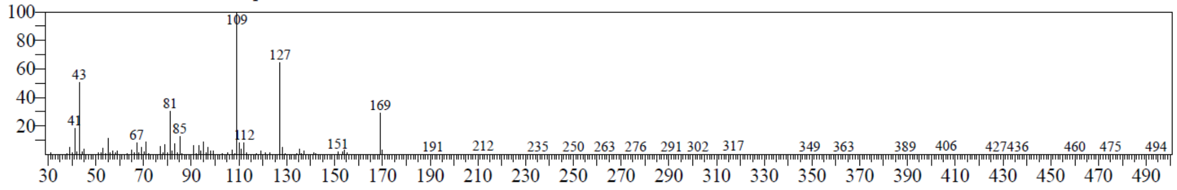

Hit#:1 Entry:25960 Library:NIST11.lib
SI:85 Formula:C10H18O2 CAS:0-00-0 MolWeight:170 RetIndex:1259
CompName:1,4-dihydroxy-p-menth-2-ene

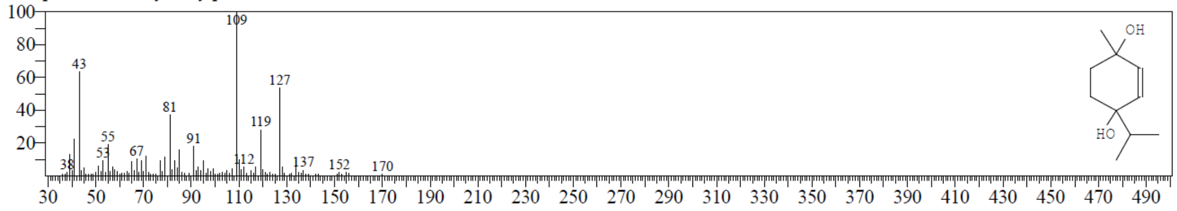

Figure S155: Mass spectrum of 1,4-dihydroxy-p-menth-2-ene (retention time 11.475).

<< Target >>
Line#:25 R.Time:11.695(Scan#:1720) MassPeaks:278
RawMode:Averaged 11.690-11.700(1719-1721) BasePeak:43.05(28880)
BG Mode:Calc. from Peak Group 1 - Event 1 Scan

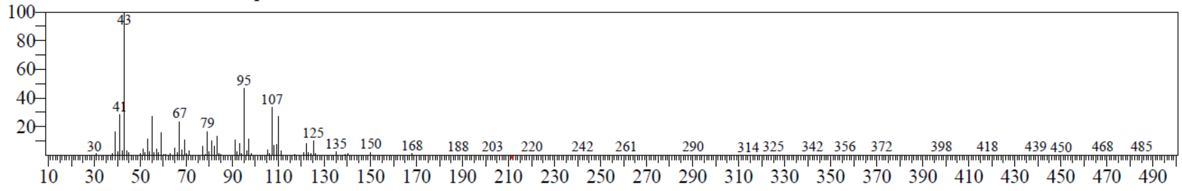

Hit#:2 Entry:24485 Library:NIST11.lib
SI:85 Formula:C10H16O2 CAS:96-08-2 MolWeight:168 RetIndex:1128
CompName:7-Oxabicyclo[4.1.0]heptane, 1-methyl-4-(2-methyloxiranyl)- $$ p-Menthane, 1,2:8,9-diepoxy- $$ .alpha.-Limonene diepoxide $$ Dipentene die[illegible]

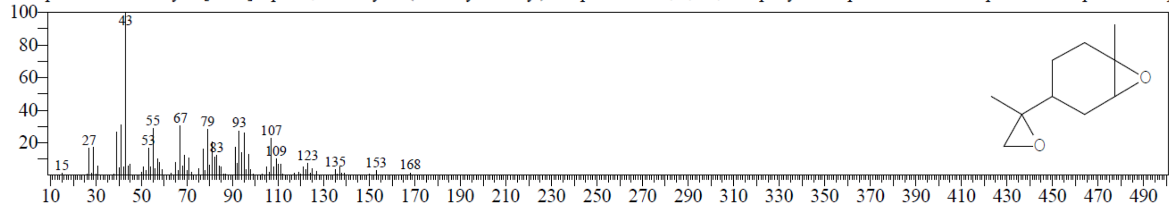

Hit#:4 Entry:24606 Library:NIST11.lib
SI:82 Formula:C10H16O2 CAS:0-00-0 MolWeight:168 RetIndex:1169
CompName:5-Isopropenyl-2-methyl-7-oxabicyclo[4.1.0]heptan-2-ol

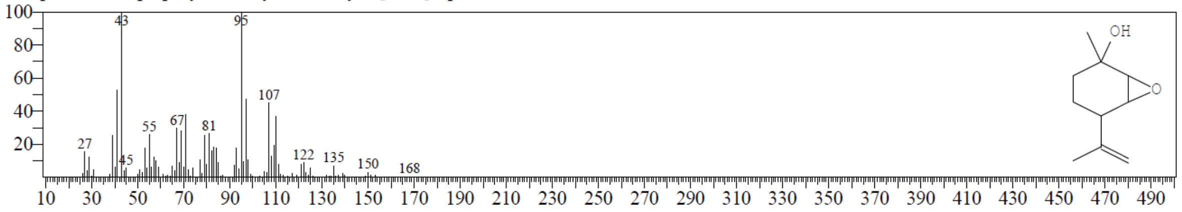

Figure S156: Mass spectrum of α-Limonene diepoxide (retention time 11.695).

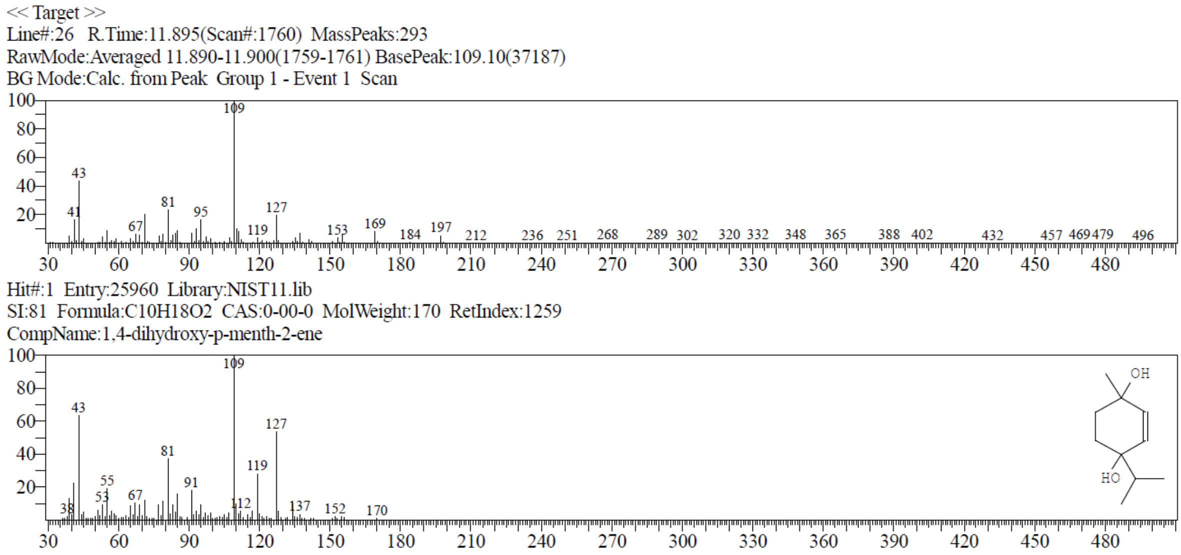

Figure S157: Mass spectrum of 1,4-dihydroxy-p-menth-2-ene (retention time 11.895).

15.5 GC-MS analysis for confirmation of oleic acid hydroperoxide

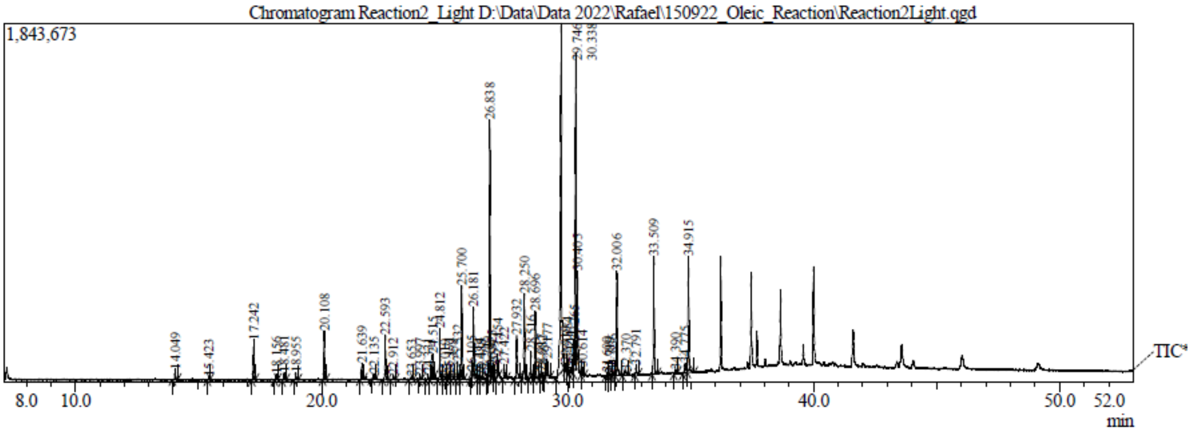

Peak Report TIC

Peak#	R.Time	Area	Area%	Height	Height%	Name	Base m/z	Base Int.
1	14.049	91914	0.34	53351	0.47	Cyclohexasiloxane, dodecamethyl-	73.05	13505
2	15.423	11816	0.04	14115	0.12	undecane	57.10	4923
3	17.242	391087	1.45	204853	1.80	Cycloheptasiloxane, tetradecamethyl-	73.05	41218
4	18.156	53810	0.20	30044	0.26	Trimethyl(2,6 ditert.-butylphenoxy)silane	263.10	9252
5	18.481	83716	0.31	37034	0.33	Silane, (dodecyloxy)trimethyl-	243.15	6506
6	18.955	72211	0.27	35782	0.31	Hexadecane	57.10	8902
7	20.108	479034	1.78	251575	2.21	Cyclooctasiloxane, hexadecamethyl-	73.05	53911
8	21.639	161496	0.60	83798	0.74	1-Trimethylsilyloxytetradecane	271.15	16573
9	22.135	63602	0.24	27492	0.24	Tricosane, 2-methyl-	57.10	5861
10	22.593	468412	1.74	231797	2.04	Cyclononasiloxane, octadecamethyl-	73.05	56126
11	22.912	53628	0.20	25665	0.23	Tetradecanoic acid, trimethylsilyl ester	73.05	2969
12	23.653	24639	0.09	14826	0.13	Tetradecanenitrile	97.10	1122
13	23.997	26639	0.10	22062	0.19	Hexadecanoic acid, methyl ester	74.05	4253
14	24.331	56936	0.21	26390	0.23	n-Pentadecanoic acid, trimethylsilyl ester	117.05	2641
15	24.515	184759	0.69	122751	1.08	Silane, (hexadecyloxy)trimethyl-	299.20	25496
16	24.812	507836	1.89	258628	2.27	Cyclodecasiloxane, eicosamethyl-	73.05	50415
17	25.019	29585	0.11	15733	0.14	Docosane, 11-butyl-	57.10	3122
18	25.171	55274	0.21	29462	0.26	1H-Indole-2,3-dione, 1-(tert-butyldimethylsily	201.00	2142
19	25.358	25665	0.10	16182	0.14	Palmitelaidic acid, trimethylsilyl ester	117.05	1452
20	25.532	177111	0.66	89567	0.79	Adipic acid, heptyl methyl ester	143.00	15187
21	25.700	928447	3.45	482167	4.24	Hexadecanoic acid, trimethylsilyl ester	117.05	57151
22	26.105	22826	0.08	26266	0.23	1,E-8,Z-10-Pentadecatriene	67.05	3712
23	26.181	631348	2.34	345851	3.04	Oleanitrile	55.10	33397
24	26.414	41560	0.15	18759	0.16	1,37-Octatriacontadiene	55.05	1515
25	26.488	38934	0.14	17030	0.15	1,37-Octatriacontadiene	57.10	1402
26	26.740	34562	0.13	22667	0.20	Diethylmalonic acid, dodecyl 2-isopropoxyphe	74.05	4320
27	26.838	2842265	10.55	1315939	11.57	Octadec-9Z-enol trimethylsilyl ether	75.05	123858
28	26.925	31279	0.12	41308	0.36	Octadec-9Z-enol trimethylsilyl ether	75.00	5671
29	26.997	61930	0.23	39478	0.35	Heptadecanoic acid, trimethylsilyl ester	117.05	4861
30	27.154	313864	1.17	115477	1.02	Silane, trimethyl(octadecyloxy)-	327.20	19201
31	27.422	171886	0.64	74130	0.65	Hexadecanamide	59.05	27078
32	27.932	653811	2.43	221116	1.94	Oleic acid, trimethylsilyl ester	73.05	18174
33	28.250	1160320	4.31	436026	3.83	Octadecanoic acid, trimethylsilyl ester	73.05	46541
34	28.516	285457	1.06	136382	1.20	Tributyl acetylcitrate	185.05	30661
35	28.696	721973	2.68	347759	3.06	Cyclononasiloxane, octadecamethyl-	73.05	57117
36	28.917	62885	0.23	34220	0.30	10-Undecenoic acid, tert-butyldimethylsilyl est	241.15	7365
37	28.982	36977	0.14	21424	0.19	12-Hydroxylauric acid, Di(trimethylsiloxyl-)	73.05	2243
38	29.177	311007	1.15	89760	0.79	1-Trimethylsilyloxy-2-undecene (E)	73.05	10228
39	29.746	6452932	23.96	1792649	15.76	9-Octadecenamide, (Z)-	59.05	329793
40	29.919	48773	0.18	30700	0.27	9-Octadecenoic acid, 2-[(trimethylsilyl)oxy]-1-	73.05	1964
41	29.984	191893	0.71	91024	0.80	Octadecanamide	59.05	32251
42	30.111	107660	0.40	30189	0.27	Diisooctyl adipate	129.05	5489
43	30.265	153211	0.57	87656	0.77	9,12-Octadecadienoic acid (Z,Z)-, trimethylsil	75.05	6551
44	30.338	3161870	11.74	1544761	13.58	Oleamide, N-trimethylsilyl-	131.10	149695
45	30.405	736074	2.73	449515	3.95	Cyclononasiloxane, octadecamethyl-	73.05	65911
46	30.614	85648	0.32	43445	0.38	Eicosanoic acid, trimethylsilyl ester	131.10	5535
47	31.609	22679	0.08	12950	0.11	Palmitic acid, 2-(trimethylsiloxy)ethyl ester	57.10	1198
48	31.719	32643	0.12	12486	0.11	Tetradecanoic acid, trimethylsilyl ester	73.00	1754
49	31.836	87369	0.32	26117	0.23	Diisooctyl phthalate	149.00	4294
50	32.006	1269296	4.71	533825	4.69	Cyclononasiloxane, octadecamethyl-	73.05	73377

Figure S158: GC-MS of oleic acid hydroperoxide (after esterification and silylation).

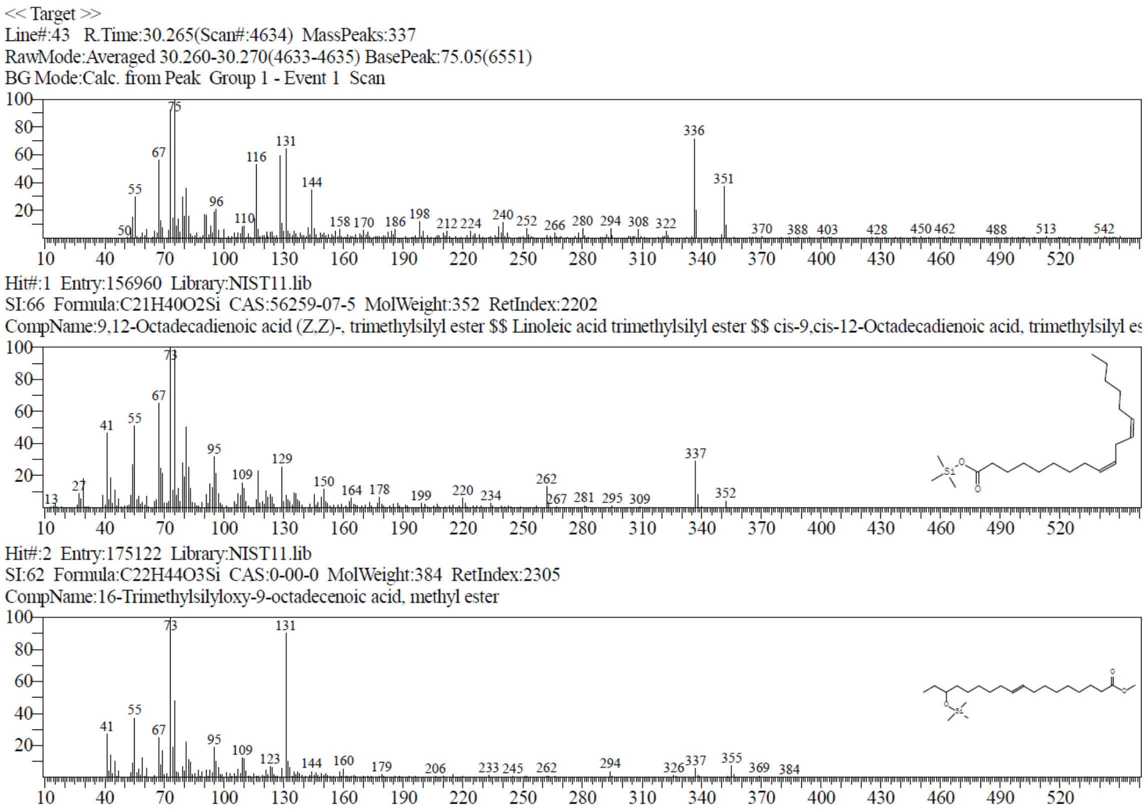

Figure S159: Mass spectrum of oleic acid hydroperoxide (after esterification and silylation).

14. References

1. L. N. Lameijer, S. Budzak, N. A. Simeth, M. J. Hansen, B. L. Feringa, D. Jacquemin, W. Szymanski, *Angew. Chem. Int. Ed.* **59**, 21663–21670 (2020).
2. Scott, S. L., Gunnoe, T. B., Fornasiero, P. & Crudden, C. M. To Err is Human; To Reproduce Takes Time. *ACS Catalysis* **12**, 3644–3650 (2022).
3. Manochio, C., Andrade, B. R., Rodriguez, R. P. & Moraes, B. S. Ethanol from biomass: A comparative overview. *Renewable and Sustainable Energy Reviews* **80**, 743–755 (2017).
4. Terés, S. et al. Oleic acid content is responsible for the reduction in blood pressure induced by olive oil. *Proceedings of the National Academy of Sciences of the United States of America* **105**, 13811–13816 (2008).
5. Duan, H., Wang, D. & Li, Y. Green chemistry for nanoparticle synthesis. *Chemical Society Reviews* **44**, 5778–5792 (2015).
6. Tang, S. L. Y., Smith, R. L. & Poliakoff, M. Principles of green chemistry: PRODUCTIVELY. *Green Chem.* **7**, 761–762 (2005).
7. Pylypchuk, I. & Sipponen, M. H. Organic solvent-free production of colloidally stable spherical lignin nanoparticles at high mass concentrations. *Green Chemistry* **24**, 8705–8715 (2022).
8. Poison Facts: Low Chemicals: Iron Pentacarbonyl.
9. Tsukamoto, D. et al. Photocatalytic H_2O_2 production from ethanol/O_2 system using TiO_2 loaded with Au-Ag bimetallic alloy nanoparticles. *ACS Catalysis* **2**, 599–603 (2012).
10. Miglbauer, E., Gryszel, M. & Głowacki, E. D. Photochemical evolution of hydrogen peroxide on lignins. *Green Chem.* **22**, 673–677 (2020).
11. Fenton, H. J. H. LXXIII.—Oxidation of tartaric acid in presence of iron. *Journal of the Chemical Society, Transactions* **65**, 899–910 (1894).
12. Babuponnusami, A. & Muthukumar, K. A review on Fenton and improvements to the Fenton process for wastewater treatment. *Journal of Environmental Chemical Engineering* **2**, 557–572 (2014).
13. Fu Y., Zhu Y., Shi S. Q., Goodell B. Formaldehyde emission from wood promoted by lignin in the presence of iron residues, *Green Chem.* **24**, 6631 (2022).
14. Gopakumar, A. et al. Lignin-Supported Heterogeneous Photocatalyst for the Direct Generation of H_2O_2 from Seawater. *Journal of the American Chemical Society* **144**, 2603–2613 (2022).
15. Hermens, J. G. H. et al. A sustainable polymer and coating system based on renewable raw materials. *Green Chem.* **24**, 9772–9780 (2022).
16. Hermens, J. G. H., Freese, T., Van den Berg, K. J., Van Gemert, R. & Feringa, B. L. A coating from nature. *Science Advances* **6**, 26–42 (2020).
17. Organization, W. H., UNICEF & ICCIDD. Assessment of Iodine Deficiency Disorders and Monitoring their Elimination. WHO/NHD/01.1 Geneva, Switzerland. World Health Organization, Geneva 1–107 (2001).
18. Chen, W. M. et al. Iodometric titration for determining the oxygen content of samples doped with Fe and Co. *Physica C: Superconductivity* **270**, 349–353 (1996).
19. Ichihara, K. & Fukubayashi, Y. Preparation of fatty acid methyl esters for gas-liquid chromatography. *Journal of lipid research* **51**, 635–640 (2010).
20. Shiraishi, Y. et al. Resorcinol–formaldehyde resins as metal-free semiconductor photocatalysts for solar-to-hydrogen peroxide energy conversion. *Nature Materials* 18:9 **18**, 985–993 (2019).
21. Liu, L. et al. Linear Conjugated Polymers for Solar-Driven Hydrogen Peroxide Production: The Importance of Catalyst Stability. *Journal of the American Chemical Society* **143**, 19287–19293 (2021).
22. Fónagy, O., Szabó-Bárdos, E. & Horváth, O. 1,4-Benzoquinone and 1,4-hydroquinone based determination of electron and superoxide radical formed in heterogeneous photocatalytic systems. *Journal of Photochemistry and Photobiology A: Chemistry* **407**, 113057 (2021).
23. Von Piechowski, M., Thelen, M.-A., Hoignc, J., Biihler, R. E. & Kinetics, C. tert-Butanol as an OH-Scavenger in the Pulse Radiolysis of Oxygenated Aqueous Systems. *Berichte der Bunsengesellschaft für physikalische Chemie* **96**, 1448–1454 (1992).

24. Doudrick, K., Yang, T., Hristovski, K. & Westerhoff, P. Photocatalytic nitrate reduction in water: Managing the hole scavenger and reaction by-product selectivity. *Applied Catalysis B: Environmental* 136–137, 40–47 (2013).
25. Goor, G., Glenneberg, J., Jacobi, S., Dadabhoy, J. & Candido, E. Hydrogen Peroxide. *Ullmann's Encyclopedia of Industrial Chemistry* 1–40 (Wiley-VCH Verlag GmbH & Co. KGaA, 2019).
26. Sandra M. Mendoza, Imad Arfaoui, Simone Zanarini, Francesco Paolucci, and Petra Rudolf, *Langmuir* **23** (2), 582-588 (2007).
27. Taufiq, A. et al. Synthesis of Fe_3O_4/Ag nanohybrid ferrofluids and their applications as antimicrobial and antifibrotic agents. *Heliyon* **6**, (2020).
28. T. Radu, A. Petran, D. Olteanu, I. Baldea, M. Potara, R. Turcu, Evaluation of physico-chemical properties and biocompatibility of new surface functionalized Fe_3O_4 clusters of nanoparticles, *Appl Surf Sci*, **501**, 144267 (2020).
29. NIST X-ray Photoelectron Spectroscopy (XPS) Database, Version 3.5, https://srdata.nist.gov/xps/, (accessed 2 June 2023).
30. D. Tahir, S. Ilyas, B. Abdullah, B. Armynah and H. J. Kang, J *Electron Spectros Relat Phenomena*, **229**, 47–51 (2018).
31. D. Wilson and M. A. Langell, *Appl Surf Sci*, **303**, 6–13 (2014).
32. M. C. Biesinger, Accessing the robustness of adventitious carbon for charge referencing (correction) purposes in XPS analysis: Insights from a multi-user facility data review, *Appl Surf Sci*, **597**, 153681 (2022).
33. J. W. Jang, C. Du, Y. Ye, Y. Lin, X. Yao, J. Thorne, E. Liu, G. McMahon, J. Zhu, A. Javey, J. Guo and D. Wang, Enabling unassisted solar water splitting by iron oxide and silicon, *Nat Commun*, **6**, Article number: 7447 (2015).
34. W. Zhu, Y. Wei, Z. Liu, Y. Zhang, H. He, S. Yang, Z. Li, Z. Zou and Y. Zhou, *Catal Sci Technol*, **12**, 4372–4379 (2022).
35. H. S. Yu, X. He, S. L. Li, D. G. Truhlar, *Chem. Sci.* **7**, 5032–5051 (2016).
36. J. Zheng, X. Xu, D. G. Truhlar, Theor. *Chem. Acc.* **128**, 295–305 (2011).
37. A. V. Marenich, C. J. Cramer, D. G. Truhlar, *J. Phys. Chem. B* **113**, 6378–6396 (2009).
38. Skill, N. J. et al. Role of cyclooxygenase isoforms in prostacyclin biosynthesis and murine prehepatic portal hypertension. *Am J Physiol Gastrointest Liver Physiol* **295**, 953–964 (2008).
39. Chandrasekharan, N. V & Simmons, D. L. The cyclooxygenases. *Genome biology* **5**, 1-7 (2004).
40. Van der Donk, W. A., Tsai, A. L. & Kulmacz, R. J. The cyclooxygenase reaction mechanism. *Biochemistry* **41** 15451–15458 (2002).
41. Xiao, Y. et al. Revealing Kinetics of Two-Electron Oxygen Reduction Reaction at Single-Molecule Level. *Journal of the American Chemical Society* **142**, 13201–13209 (2020).
42. Priddy, D. B. Recent advances in styrene polymerization. *Advances in Polymer Science* **111**, 66–114 (1994).
43. Simič, R., Mandal, J., Zhang, K. & Spencer, N. D. Oxygen inhibition of free-radical polymerization is the dominant mechanism behind the "mold effect" on hydrogels. *Soft Matter* **17**, 6394–6403 (2021).
44. Krivtsov, I., Vazirani, A., Mitoraj, D. & Beranek, R. Benzaldehyde-Promoted (Auto)Photocatalysis under Visible Light: Pitfalls and Opportunities in Photocatalytic H_2O_2 Production, *ChemCatChem* **15**, e202201215 (2023).
45. Freese, T., Meijer, J.T., Feringa, B.L. *et al.* An organic perspective on photocatalytic production of hydrogen peroxide. *Nat Catal* **6**, 553–558 (2023). https://doi.org/10.1038/s41929-023-00980-x.
46. Moon, B. C., Bayarkhuu, B., Zhang, K. A. I., Lee, D. K. & Byun, J. Solar-driven H2O2 production via cooperative auto- and photocatalytic oxidation in fine-tuned reaction media, *Energy Environ. Sci.* **15**, 5082–5092 (2022).
47. Bhattacharjee, A., LaVigne, A. R., Frazee, S. M., Herrera, T. L. & McCormick, T. M. Photocatalytic aerobic oxidation of benzylic alcohols and concomitant hydrogen peroxide production. *Chem. Commun.* **59**, 1090-1093 (2023)
